# Phosphonoalamides Reveal the Biosynthetic Origin of Phosphonoalanine Natural Products and a Convergent Pathway for Their Diversification

**DOI:** 10.1002/anie.202405052

**Published:** 2024-07-09

**Authors:** Jerry J. Cui, Yeying Zhang, Kou-San Ju

**Affiliations:** Department of Microbiology, The Ohio State University, 318W. 12th Ave, Columbus, OH-43210 (USA); Department of Microbiology, The Ohio State University, 318W. 12th Ave, Columbus, OH-43210 (USA); Department of Microbiology, The Ohio State University, 318W. 12th Ave, Columbus, OH-43210 (USA); Division of Medicinal Chemistry and Pharmacognosy, Center for Applied Plant Sciences, Infectious Disease Institute, The Ohio State University, 318W. 12th Ave, Columbus, OH-43210 (USA)

**Keywords:** Biosynthesis, Ligases, Natural Products, Peptides, Phosphonates

## Abstract

Phosphonate natural products, with their potent inhibitory activity, have found widespread use across multiple industries. Their success has inspired development of genome mining approaches that continue to reveal previously unknown bioactive scaffolds and biosynthetic insights. However, a greater understanding of phosphonate metabolism is required to enable prediction of compounds and their bioactivities from sequence information alone. Here, we expand our knowledge of this natural product class by reporting the complete biosynthesis of the phosphonoalamides, antimicrobial tripeptides with a conserved *N*-terminal l-phosphonoalanine (PnAla) residue produced by *Streptomyces.* The phosphonoalamides result from the convergence of PnAla biosynthesis and peptide ligation pathways. We elucidate the biochemistry underlying the transamination of phosphonopyruvate to PnAla, a new early branchpoint in phosphonate biosynthesis catalyzed by an aminotransferase with evolved specificity for phosphonate metabolism. Peptide formation is catalyzed by two ATP-grasp ligases, the first of which produces dipeptides, and a second which ligates dipeptides to PnAla to produce phosphonoalamides. Substrate specificity profiling revealed a dramatic expansion of dipeptide and tripeptide products, while finding PnaC to be the most promiscuous dipeptide ligase reported thus far. Our findings highlight previously unknown transformations in natural product biosynthesis, promising enzyme biocatalysts, and unveil insights into the diversity of phosphonopeptide natural products.

## Graphical Abstract

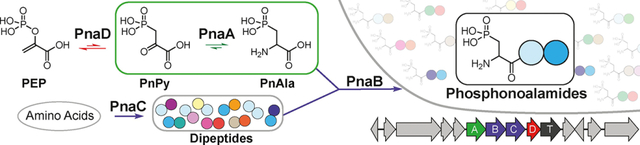

The *Streptomyces* phosphonoalamides result from the convergence of phosphonoalanine (PnAla) biosynthesis and peptide ligation pathways. The transamination of phosphonopyruvate (PnPy) to PnAla drives unfavorable C–P bond formation despite its reversibility, establishing this early branch of phosphonate biosynthesis, while the promiscuity of the dipeptide and tripeptide ligases result in a dramatic expansion of observed di- and tripeptide products.

## Introduction

Phosphonate and phosphinate (Pn) compounds are characterized by their direct C–P bonds. This moiety, which enables chemical mimicry of phosphate esters and carboxylic acids, is responsible for the bioactivity of these compounds.^[1]^ The majority of Pn natural products (NPs) are potent metabolic inhibitors, with a historical commercialization rate over two order of magnitudes greater than NPs as a whole.^[2]^ These compounds have found broad success, with examples including the antibiotic fosfomycin (Monurol), the antiviral phosphonoformate (Foscarnet), and the antimalarial fosmidomycin. Fosfomycin mimics phosphoenolpyruvate (PEP) to covalently inhibit MurA, blocking peptidoglycan biosynthesis.^[3]^ Foscarnet is a pyrophosphate analog which inhibits viral polymerases.^[4]^ Lastly, fosmidomycin blocks the non-mevalonate pathway of isoprenoid biosynthesis by inhibiting 1-deoxy-d-xylulose 5-phosphate reductoisomerase, and has shown promising results against malaria in human trials.^[5]^

**Figure 1.**
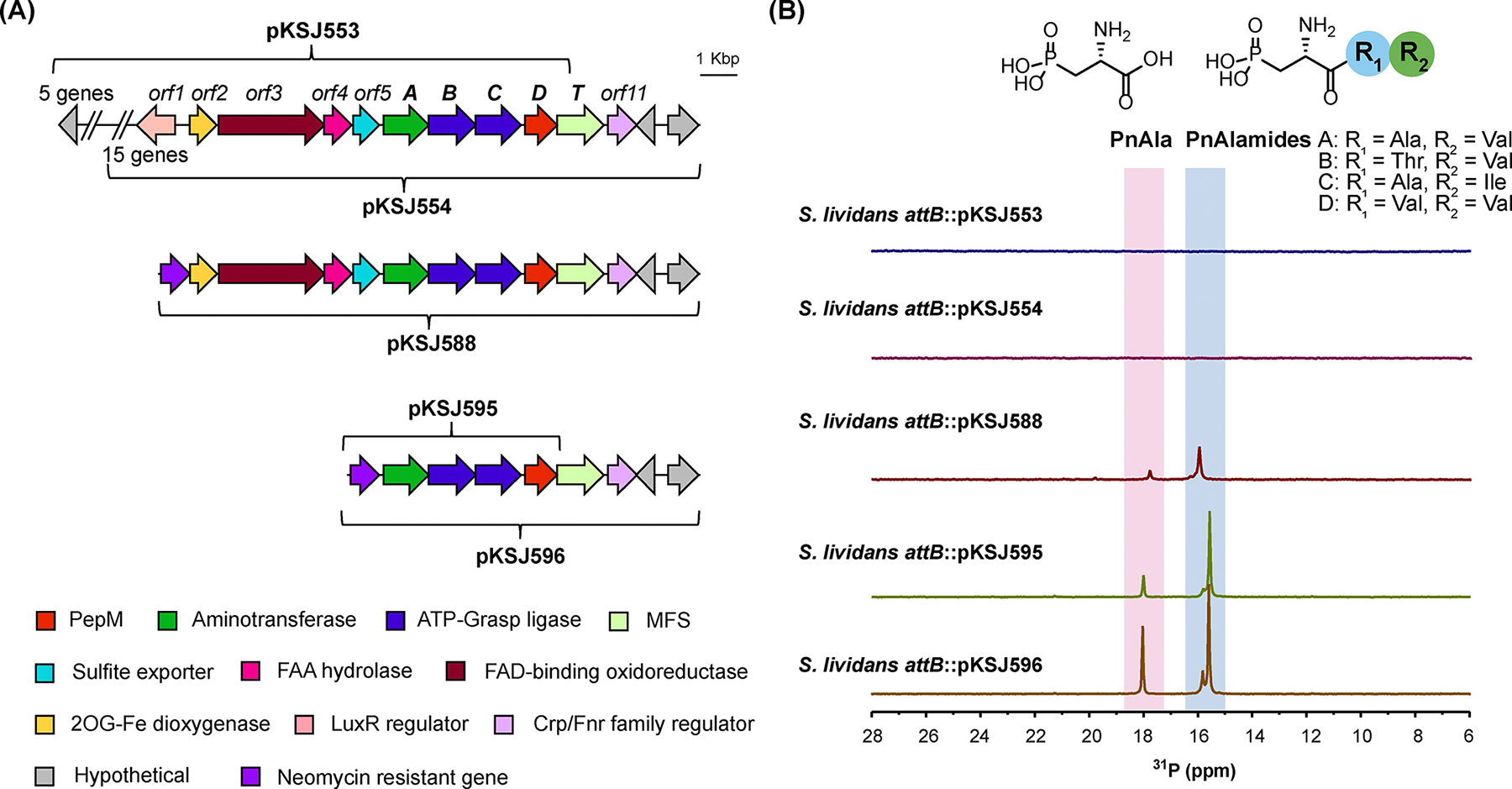
A) The genome neighborhood of the phosphonoalamide biosynthetic gene cluster, with cloned cosmid inserts indicated by brackets. Annotation of *orf1–11* is provided in Table S1. B) ^31^P NMR analysis of heterologous expression strain extracts. Slight variation in chemical shifts is due to minor differences in pH and salt content between samples.

This established track record for Pn NPs has inspired genomics-driven methods that have resulted in the discovery of multiple, diverse, antimicrobial and herbicidal Pn scaffolds.^[2,6]^ Even with these findings, genomic data suggest a tremendous wealth of unexplored chemical diversity remains, as nearly 7% of all bacteria are predicted to encode biosynthetic pathways for Pn NPs.^[7]^ In spite of this, a greater understanding of these biosynthetic gene clusters (BGCs) is required to improve the discovery process, facilitating accurate prediction and classification of their pathways, products, and biological activities. While all known Pn biosynthesis begins with the isomerization of PEP to phosphonopyruvate (PnPy) by phosphoenolpyruvate mutase (PepM), this reaction is highly unfavorable, and requires a coupled enzymatic reaction to provide the thermodynamic energy to drive it forward.^[8]^ This principle was leveraged to identify a family of BGCs encoding a previously unknown coupling enzyme, resulting in the isolation of L-phosphonoalanine (PnAla) and four tripeptides with an *N-*terminal PnAla (phosphonoalamides A–D) from *Streptomyces* sp. NRRL B-2790 (Figure 1B).^[6b]^

Although PnAla was the second Pn compound ever isolated from biological material,^[9]^ the role it plays in Nature still remains unclear six decades later. In experimental physiology, synthetic PnAla is used within cerebral tissues as a selective antagonist of metabotropic glutamate receptors and an inhibitor of phosphoserine phosphatases.^[10]^ PnAla has been found within multiple tissues including the human liver, intestine, and spleen,^[11]^ and genes implicated in PnAla degradation are found across several classes of bacteria.^[12]^ The isolation of strains capable of using free PnAla as a sole carbon, nitrogen, and phosphorus source suggest it, like other Pn compounds, may play a role in global nutrient cycles.^[12–13]^ However, PnAla had not been observed as a component of a small molecule or free amino acid until our isolation of PnAla and its peptide conjugants, the phosphonoalamides.^[6b,c]^ These studies also revealed their biological activity as potent broad-spectrum antibacterials, suggesting roles in microbial chemical ecology beyond that of a mere nutrient.^[6b,c]^

Despite its frequent isolation from biological macromolecules and widespread use in research, several aspects of PnAla metabolism remain uncharacterized, including the nature of the transamination resulting in its biosynthesis and rationale for how this reaction thermodynamically drives unfavorable C–P bond formation.^[1,6b]^ The mechanism of PnAla incorporation into peptides also remains unknown, though conservation of biosynthetic genes between the *Streptomyces* (*N*-terminal PnAla) and *Bacillus* (*C-*terminal PnAla) BGCs suggest ATP-grasp ligases are responsible for amide bond formation, with their differential specificities driving diversification of these peptides.^[6b,c]^ The existence of additional related BGCs among terrestrial, marine, and plant-associated bacteria, as well as the differing spectrums of activity between phosphonoalamide A (PnAla-Ala-Val) and phosphonoalamide F (Ala-Ala-PnAla), further underscore the diversity and potential of PnAla-containing NPs.^[6c]^ Here we advance our knowledge of Pn metabolism by establishing the genetic and biochemical logic for phosphonoalamide biosynthesis in *Streptomyces.* We defined the BGC using heterologous expression and deletion analyses, with biochemical reconstitution demonstrating phosphonoalamide A to be the product of four enzymatic reactions. Among these steps, the pyridoxal-5’-phosphate (PLP)-dependent transamination of PnPy to PnAla by PnaA represents a new early branchpoint in Pn metabolism, analogous to the reaction catalyzed by aspartate aminotransferase (AAT). Tripeptide formation was revealed to be a convergent process catalyzed by two distinct ATP-grasp l-amino acid ligases. PnaC produced a diverse array of dipeptides, which were then ligated to PnAla by PnaB. The natural specificity of these ligases was leveraged to generate a total of 181 dipeptides and 97 PnAla-tripeptides, of which 93 had not been previously observed in Nature.

## Results and Discussion

### Delineation of the Phosphonoalamide Biosynthetic Gene Cluster

All characterized pathways for Pn biosynthesis begin with the isomerization of PEP to PnPy by PepM. PepMs with at least 80% sequence identity have been shown to yield similar Pn NPs,^[6a]^ a fact which was previously used to identify PnAla and the phosphonoalamides from a group of *Streptomyces.*^[6b]^ As these strains produced the same compounds, we reasoned that the BGC would consist of genes within the *pepM* neighborhood which were conserved between them. To provide contigs of sufficient length for synteny analysis, the genomes of *Streptomyces* sp. NRRL B-2790 and S-488 were re-sequenced. In addition to *pepM* (designated as *pnaD*), clear conservation was observed for genes encoding a putative transcriptional regulator (*orf1*), two oxidoreductases (*orf2* and *orf3*), a hydrolase (*orf4*), a transaminase (*pnaA*), two ligases (*pnaB* and *pnaC*), and two transporters (*orf5* and *pnaT*) (Figure S1; Table S1). Except for *orf1* (putative FAA hydrolase) and *orf11* (Crp/Fnr transcriptional regulator), gene arrangement was largely conserved across all 10 strains. Breaks in synteny beyond *orf1/2* and *orf11* suggested these as the boundaries of the BGC. However, *orf1–5* were present in several genomic neighborhoods of *Streptomyces* unrelated to Pn biosynthesis, suggesting they were not involved (Figure S2).

To clearly establish the BGC boundaries, we performed heterologous expression and deletion analysis of the gene neighborhood. We first constructed a fosmid library of *Streptomyces* sp. NRRL S-515, selected two clones encoding overlapping regions of the *pnaD* neighborhood (pKSJ553 and pKSJ554), and integrated them into the ΦC31 *attB* site of *S. lividans* 66. Metabolites produced by the resulting strains (*S. lividans* 66 *attB*::pKSJ553, *S. lividans* 66 *attB*::pKSJ554) were analyzed by ^31^P NMR and LC-HRMS and compared to a negative integration control strain (*S. lividans* 66 *attB*::pAE4). Even after growth on multiple media types, Pns were not observed from either heterologous expression strain. We reasoned the lack of production may have been due to inadequate gene expression, possibly stemming from regulation afforded by the adjacently encoded LuxR (*orf1*) and FNR-type (*orf11*) transcriptional regulators. To test this hypothesis, we used λ-Red mediated recombination to replace *orf1* and upstream genes from S-515 on clone pKSJ554 with an *aph* resistance cassette, resulting in pKSJ588 (Figure 1A). This significantly improved Pn production, as evidenced by the multiple Pn signals observed within *S. lividans* 66 *attB*::pKSJ588 extracts (Figure 1B). ^31^P and ^1^H-^31^P HMBC spectra matched established literature values for PnAla and phosphonoalamides A–D (Figure 1B, Figure S3)^[6b]^ while LC-HRMS and LC-HRMS/MS fragmentation verified the production of these compounds (Figure S4).

We further truncated the BGC to determine if *orf2–5*, *pnaT*, and *orf11* are required for Pn biosynthesis. All 6 genes were deleted to create pKSJ595, while only *orf2–5* were deleted to create pKSJ596. Interestingly, analysis of both *S. lividans* 66 *attB*::pKSJ595 and *S. lividans* 66 *attB*::pKSJ596 by ^31^P NMR and LC-HRMS revealed an identical profile of Pn species produced, but with greater abundance than in *S. lividans* 66 *attB*::pKSJ588. These results were consistent with our synteny analyses and clearly demonstrate *pnaABCD* as the only genes required for phosphonoalamide biosynthesis.

### A Dedicated Phosphonopyruvate Transaminase for L-PnAla Biosynthesis

Having established the minimal BGC, we focused our attention on the biosynthesis of PnAla and its peptide derivatives. Our previous co-expression of *pnaD* and *pnaA* in *S. albus* J1074 resulted in production of PnAla,^[6b]^ suggesting that PnaA functions as a coupling enzyme for PepM to drive the formation of PnPy. PnaA was identified as a PLP-dependent aminotransferase belonging to the AAT superfamily (Table S1). Canonical AATs catalyze the interconversion of l-Asp and α-ketoglutarate (αKG) to oxaloacetate (OAA) and L-Glu using PLP reaction chemistry.^[14]^ This, along with clear structural similarities between PnPy and PnAla to OAA and l-Asp, suggested PnaA may have originated as an AAT that subsequently evolved reaction specificity for Pn substrates.

**Figure 2.**
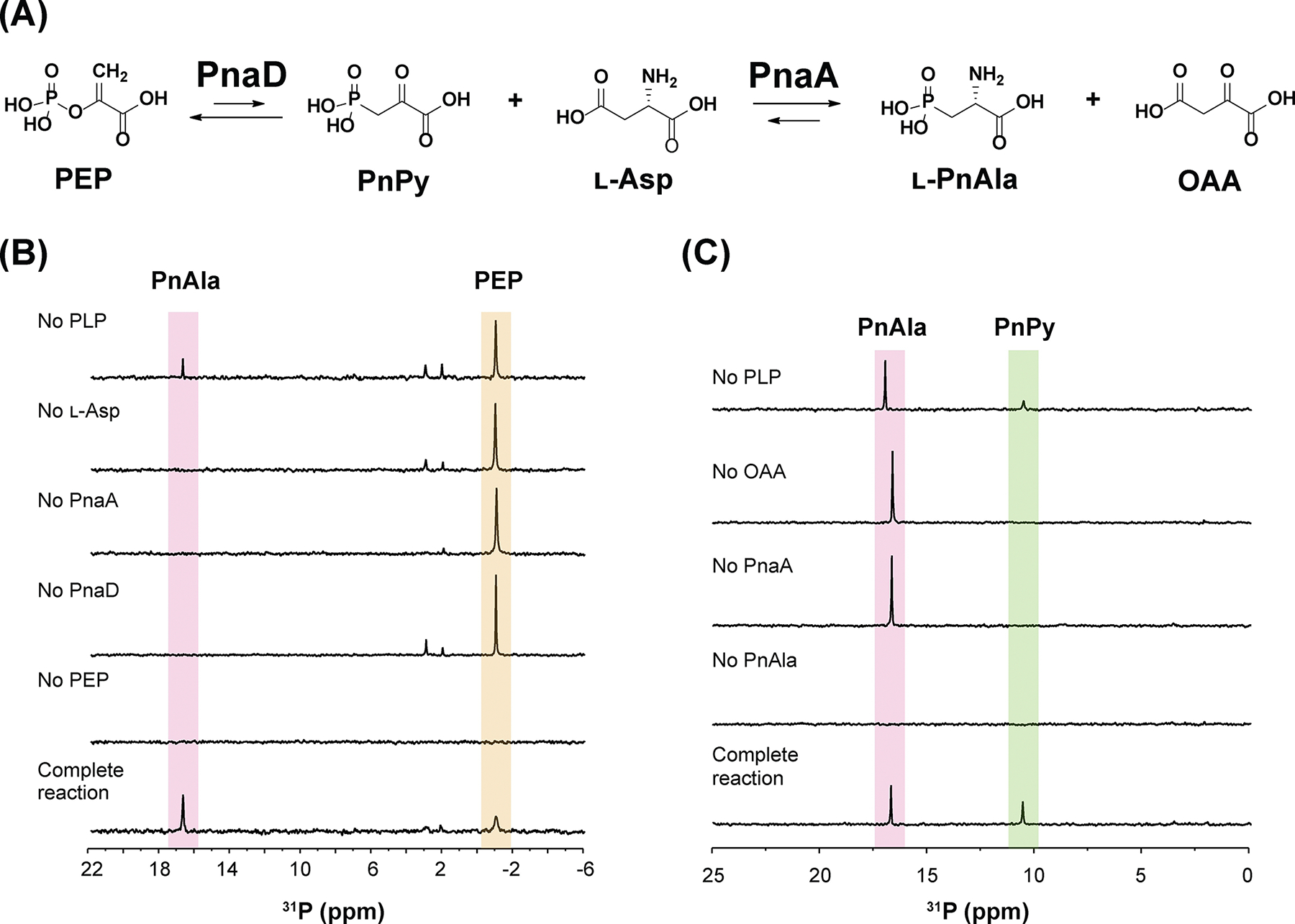
A) Biosynthetic scheme for l-PnAla formation. ^31^P NMR analysis of B) PnaAD reactions demonstrating conversion of PEP to PnAla and C) PnaA reverse reactions demonstrating conversion of PnAla to PnPy. Slight variation in chemical shifts is due to minor differences in pH and salt content between reactions.

To test this hypothesis and investigate the biochemical nature of PnAla formation, we first overexpressed and purified recombinant PnaD and PnaA from *E. coli* for in vitro assays (Figure S5). We proposed that following isomerization of PEP to PnPy by PnaD, the amine of l-Asp would be transferred onto the keto moiety of PnPy by PnaA to yield PnAla and OAA (Figure 2A). Indeed, incubating PnaA with PnaD, PEP, Asp, and PLP resulted in consumption of PEP (−1.5 ppm) and emergence of a new species (16.5 ppm) in the ^31^P NMR spectrum (Figure 2B). ^1^H-^31^P HMBC experiments identified the new signal as PnAla (Figure S6). Excluding any reaction component other than PLP eliminated PnAla production. Without addition of PLP, enzyme activity was observed at significantly reduced amounts, likely due to endogenous cofactor that co-purified with PnaA. Unlike other aminotransferases, PnAla formation by PnaA was strictly dependent on l-Asp as the amino donor and substitution with other proteinogenic amino acids was not tolerated (Figure S7).

Having established general reaction conditions for PnaA, we examined its ability to catalyze the reverse reaction, transamination of OAA to l-Asp using l-PnAla as an amino donor. Incubating PnaA with PnAla, OAA, and PLP resulted in a new species in ^31^P NMR (11.0 ppm) with ^1^H-^31^P HMBC spectra showing correlating protons at 3.17 ppm (Figure 2C; Figure S8).^[8]^ These properties were consistent with PnPy. To validate its identity, we derivatized the new species with phenylhydrazine to improve its stability and detectability by LC-HRMS. Indeed, both monomeric [M–H]^−^ (257.0332 *m/z*) and dimeric [2M–H]^−^ (515.0738 *m/z*) forms of the phenylhydrazine-PnPy conjugate were readily observed, with MS/MS fragmentation confirming their identities (Figure S9–10). The reverse reaction was likewise dependent on all components and significantly greater PnPy was observed when additional PLP was provided (Figure 2C; Figure S10).

These results demonstrated PnaA as a PLP-dependent transaminase that functions as a coupling enzyme with PepM. While other PepM coupling enzymes transform PnPy using reactions that are either irreversible (decarboxylation^[15]^ and acetylation^[16]^) or utilize the co-oxidization of NAD(P)H to drive reduction forward,^[6d]^ many transamination reactions are naturally reversible under physiological conditions, with equilibrium constants close to one.^[17]^ Indeed, the PepM-coupled forward reaction of PnaA yielded ~50% PnAla, while the reverse reaction of PnaA yielded ~50% PnPy (Figures 2B&C). Nonetheless, we reasoned there must be physiologic reaction conditions and/or inherent characteristics of PnaA which allow it to drive PnAla biosynthesis forward.

Since all enzymatic transaminations require an amino donor and keto-acid acceptor, we started by examining the effects of substrate concentration on the directionality of the PnaA reaction. First, we monitored forward and reverse transamination reactions using ^31^P NMR spectroscopy to understand the general dynamics of PnaA activity over time. Rapid conversion was observed when PnaA was provided a large excess (10 mM) of amino donor (Asp) or keto-acid acceptor (OAA), reaching an apparent equilibrium within 2 h (Figure S11–12). In *S. griseus* and *S. californicus*, Asp was measured to be ~20 μmol/g cell dry weight under basal conditions.^[18]^ As bacterial cells contain 0.12–0.33 g dry weight per mL of cell volume,^[19]^ we estimate intracellular Asp concentrations to within 3–10 mM. Therefore, we varied the concentration of Asp (1.5, 3, 5, or 10 mM) in forward reactions while maintaining PEP at 1.5 mM. To examine the reverse reaction under corresponding conditions, we utilized 1.5 mM PnAla while varying the concentration of OAA (1.5, 3, 5, or 10 mM). Product formation for both forward and reverse reactions was measured by ^31^P NMR after 2 h (Figure S13). Interestingly, product formation reflected the availability of Asp and OAA co-substrates, accumulating upwards of 70% PnAla or PnPy in their respective reactions. These results indicated that the initial concentration of available amino donors and acceptors strongly influences the degree of product formation at apparent equilibrium, and favors PnAla formation under physiologic Asp concentrations.

Next, we performed kinetic analyses to derive insight on the substrate specificity and reaction selectivity of PnaA transamination. Apparent steady state parameters were calculated in spectrophotometric assays by coupling the formation of OAA, PnPy, and αKG with NADH oxidation in the presence of malate dehydrogenase (MDH), PnPy reductase (VlpB), and 3-phosphoglycerate dehydrogenase (SerA), respectively (Figures S14–20). The high catalytic efficiency observed with the conversion of Asp and PnPy into OAA and PnAla (*k*_cat_ = 2.33 ± 0.08 s^−1^, *K*_m_ = 0.0048 ± 0.0004 mM, *k*_cat_/*K*_m_ = (4.850 ± 0.44)×10^5^ M^−1^ s^−1^) further support that this conversion is the physiological reaction catalyzed by PnaA (Figure S14). The micromolar *K*_m_ value observed for PnaA with PnPy is similar to that of other PepM coupling enzymes such as PnPy decarboxylase.^[15a,20]^ In comparison, the reverse reaction (transamination of PnAla and OAA to PnPy and Asp) exhibited a 100-fold higher *K*_m_ and 100-fold lower catalytic efficiency (*k*_cat_ = 2.43 ± 0.10 s^−1^, *K*_m_ = 0.327 ± 0.040 mM, *k*_cat_/*K*_m_ = (7.43 ± 0.19)×10^3^ M^−1^ s^−1^) (Figure S15).

We also derived apparent steady state parameters for the conversion of PEP to l-PnAla in reactions with PnaD and PnaA (Figure S16). While we attempted to use equimolar amounts of enzyme, a 5:1 ratio of PnaD to PnaA was required for quantifiable NADH oxidation. PnAla formation from PEP using both biosynthetic enzymes exhibited a catalytic efficiency 1,000-fold lower (*k*_cat_ = 0.0840 ± 0.001 s^−1^, *K*_m_ = 0.546 ± 0.060 mM, *k*_cat_/*K*_m_ = (1.53 ± 0.19)×10^2^ M^−1^ s^−1^) than that of PnaA directly provided with PnPy. The lower activity in the two-enzyme system is consistent with the slower rate of l-PnAla accumulation observed in the time course experiments (Figure S12), reflects the unfavourability of PnPy formation,^[20]^ and supports the general hypothesis that PepM activity is the limiting step in Pn biosynthetic pathways.

In addition to the transamination of Pn substrates, PnaA was also capable of catalyzing the reversible conversion of Glu and OAA into αKG and Asp. However, the catalytic efficiencies observed for Glu (*k*_cat_ = 0.31 ± 0.01 s^−1^, *K*_m_ = 7.5 ± 0.7 mM, *k*_cat_/*K*_m_ = 42.0 ± 0.004 M^−1^ s^−1^, Figure S19) and αKG transamination reactions (*k*_cat_ = 1.80 ± 0.11 s^−1^, *K*_m_ = 0.899 ± 0.097 mM, *k*_cat_/*K*_m_ = (2.00 ± 0.25)×10^3^ M^−1^ s^−1^, Figure S20) were manyfold lower than the AAT dedicated for amino acid biosynthesis,^[21]^ and the above reactions with PnPy and PnAla.

Our collective results demonstrate PnaA functions as a reversible PLP-dependent transaminase with significant preference for Pn substrates while retaining minor AAT activity. While the transamination of PnPy by PnaA is a reversible reaction and its directionality is influenced by substrate concentration, our kinetic analyses of PnaA’s inherent catalytic properties and reported intracellular metabolite concentrations indicate that PnAla is likely to be efficiently produced in vivo, validating its function as a PepM coupling enzyme. Aspartate, the preferred amino donor for PnAla formation, is the second most abundant amino acid in *E. coli*^*[*22]^ and several *Streptomyces spp.*^[18,23]^ In *E. coli*, the intracellular concentration of Asp is 220 times greater than that of OAA, such that metabolic flux may further favor the transamination of PnPy to PnAla and concurrent conversion of aspartate to OAA.^[24]^ The ability to drive the PnaA reaction in reverse also offers a route for biocatalytic generation of PnPy, which is valuable as both a chemical synthon and substrate for biochemical studies of Pn metabolism.

### Phosphonoalamides are Formed by Convergent Biosynthesis

Having established the biochemistry behind PnAla formation, we redirected our efforts towards the biosynthetic reactions resulting in oligopeptide formation. The two remaining genes in the BGC encoded putative ATP-grasp ligases (*pnaB* and *pnaC*), a superfamily of enzymes that catalyze diverse reactions including those within the biosynthesis of amino acids, essential macromolecules, and peptide natural products.^[25]^ We therefore hypothesized that PnaB and PnaC may catalyze the final steps in phosphonoloamide biosynthesis by ligating amino acids to PnAla.

**Figure 3.**
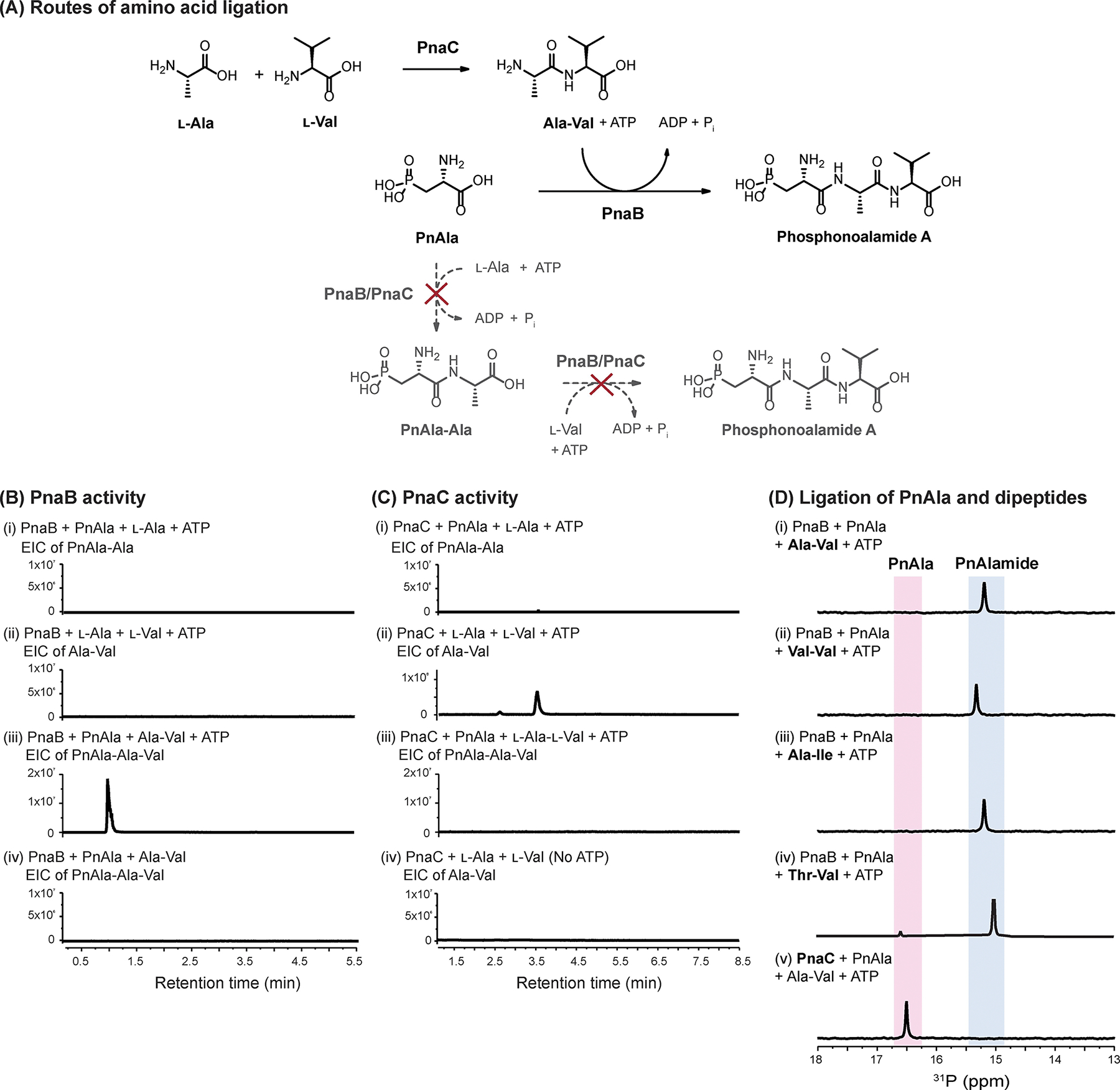
A) Potential biosynthetic routes to the phosphonoalamide A. B) LC-HRMS analyses of PnaB reactions. PnaB ligates PnAla to Ala-Val (iii) but only in the presence of ATP (iv). PnAla-Ala (i) and Ala-Val (ii) are not formed by PnaB. C) LC-HRMS analyses of PnaC reactions. PnaC ligates Ala to Val (ii) but only when provided ATP (iv). PnAla-Ala (i) and PnAla-Ala-Val (iii) are not formed by PnaC. D) ^31^P NMR analysis of PnaB ligation reactions of PnAla and chemically synthesized Ala-Val (i), Val-Val (ii), and Ala-Ile (iii). The reaction containing PnaC, PnAla, and Ala-Val served as a negative control (iv).

We envisioned two possible routes by which PnaB and PnaC could function. As phosphonoalamide A (PnAla-Ala-Val) was the most abundant phosphonopeptide from the heterologous expression and native production strains, we used it as the representative product for establishing the pathway. In the first scenario, Ala and Val may be sequentially ligated onto PnAla, with one enzyme responsible for each of the reactions. Invoking the canonical mechanism for peptide bond formation by ATP-grasp ligases, one ligase would activate the carboxylate of PnAla into an acylphosphate intermediate via ATP hydrolysis, priming it for nucleophilic attack by the amine of Ala to form PnAla-Ala. This dipeptide would then be ligated to Val by the remaining ligase, yielding phosphonoalamide A (Figure 3A). Alternatively, the pathway could begin with one ligase activating Ala to form Ala-Val. Then, PnAla would be activated by the second enzyme and ligated to the dipeptide to produce phosphonoalamide A.

We tested these hypotheses by overproducing and purifying recombinant PnaB and PnaC from *E. coli* for use in a series of biochemical assays. We incubated PnAla, Ala, and ATP with PnaB or PnaC and monitored the reactions by ^31^P NMR and LC-HRMS. Even with extended incubation up to 16 h, PnAla remained unmodified, indicating neither enzyme forms the PnAla-Ala dipeptide (Figure 3Bi and 3Ci). After substituting PnAla with Val, LC-HRMS identified Ala-Val as a product of the PnaC reaction (Figure 3Cii), with no product observed with PnaB (Figure 3Bii). To determine if either enzyme could convert Ala-Val into phosphonoalamide A, protein was removed from the PnaC reaction by ultrafiltration and PnAla, ATP, and fresh PnaC or PnaB was added to the filtrate. LC-HRMS identified PnAla-Ala-Val as a product following the addition of PnaB, but not PnaC (Figure S21). As very little phosphonoalamide A was observed, we repeated the assays with individual enzymes, ATP, PnAla, and chemically synthesized Ala-Val to eliminate the effect of competing substrates. ^31^P NMR analysis revealed complete conversion of PnAla into phosphonoalamide A by PnaB, while PnAla remained unmodified by PnaC (Figure 3Dv). Incubating PnaB with ATP, PnAla, and chemically synthesized Thr-Val, Ala-Ile, and Val-Val resulted in their respective conversion into phosphonoalamides B, C, and D (Figures 3D and S21). ATP was strictly required for reactions with PnaB and PnaC, as its omission abolished all activity (Figures 3Biv and 3Civ).

Thus, PnaB and PnaC are essential ATP-grasp ligases with distinct roles in phosphonoalamide formation. Our results also demonstrate phosphonoalamide biosynthesis in *Streptomyces* occurs via a convergent pathway. PnAla is derived from PEP by PnaD and PnaA while PnaC functions as an l-amino acid ligase (LAL) to produce dipeptides. PnaB then serves to ligate PnAla and a dipeptide to form the phosphonoalamides (Figure 3A).

### An Overlooked Diversity of L-PnAla Oligopeptides

Biosynthetic pathways which utilize ATP-grasp ligases for peptide biosynthesis are often capable of producing a mixture of products, including other phosphonopeptide NPs composed of the same Pn headgroup adorned with different amino acids.^[6b,d,26]^ These observations, combined with the isolation of four unique phosphonoalamides from *Streptomyces*, suggested PnaB and PnaC may exhibit relaxed substrate specificity, synthesizing more PnAla-oligopeptides than previously identified. With 2 variable positions and 20 proteinogenic amino acids, there exist 400 possible tripeptides with PnAla at the N-terminus. To determine whether additional variants are produced and provide physiological insight into the specificity of these ligases, we analyzed our heterologous expression data for additional tripeptides.

In addition to peptides which incorporated the amino acids comprising the isolated phosphonoalamides A–D (PnAla-Ala-Val, PnAla-Thr-Val, PnAla-Ala-Ile, and PnAla-Val-Val respectively), we detected signals corresponding to additional PnAla tripeptides containing residues which had not been seen before (Gly, Met, Gln, and Ser) (Figure S23). While these new signals were less abundant than those whose composition resembled known phosphonoalamides, these data suggested the dipeptide and tripeptide ligases PnaC and PnaB may naturally accommodate a broader range of substrates than previously realized.

### The ATP-Grasp Ligases PnaC and PnaB Underlie Diversification of L-PnAla-Containing Oligopeptides

**Figure 4.**
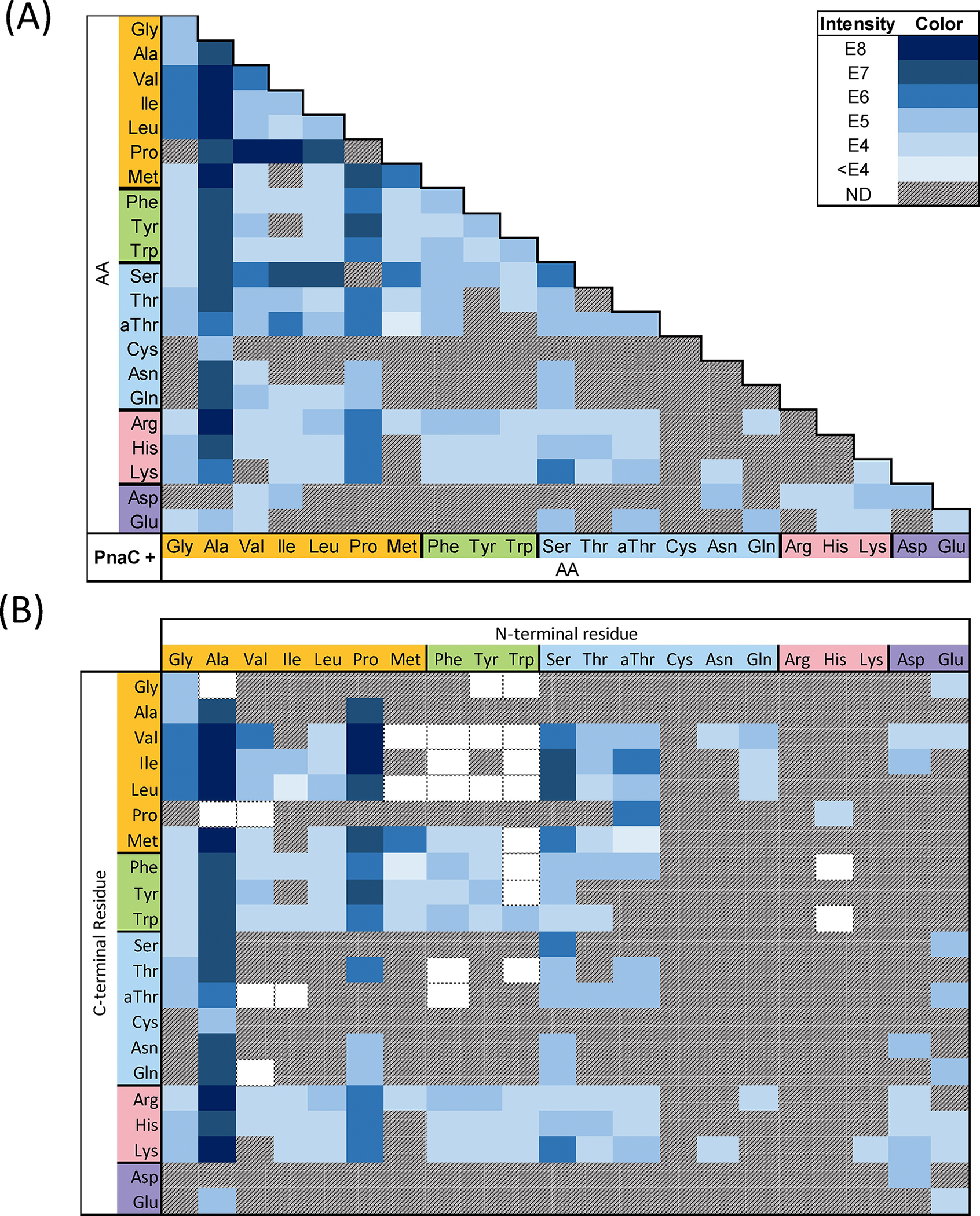
A) Summary of PnaC reactions yielding product. Each cell indicates a single reaction incorporating the corresponding amino acids. Cells are shaded based on the peak intensity of the LC-HRMS EIC for the respective product *m/z* (or not detected, ND). A key is provided in the top right indicating the ion intensity represented by each colour. B) Subsequent LC-HRMS/MS fragmentation analysis was used to determine the arrangement of each dipeptide. Co-elution of dipeptide isomers complicated their individual quantification, such that the relative intensities of their y_1_ ions was used to calculate their contribution to the overall ion intensity. White cells with a dotted border represent dipeptides inferred from the observation of an *N*-terminal L-PnAla tripeptide containing the indicated dipeptide.

To understand the biochemical nature of this potential phosphonopeptide diversity, we first delineated the substrate specificity of the dipeptide ligase PnaC. We performed microscale biochemical reactions containing purified PnaC with every combination of the 20 canonical amino acids and l-*allo*-threonine (231 total pairs of substrates). Reactions were analyzed by LC-HRMS to reveal putative dipeptides from 148 out of the 231 combinations (Figure 4A). To distinguish residues in reactions containing isomeric pairs (e.g. Ile and Leu, Thr and *allo*-Thr) new reactions were prepared using ^15^N-Ile and 4-^13^C-2,3-D_2_-Thr as substrates. While some products were only present in trace amounts, LC-HRMS/MS fragmentation analysis was performed to unambiguously verify all products and determine their structures.

Structure assignment was facilitated by identification of conserved peptide fragment ions, with the y_1_ ion enabling assignment of the *N-*to-*C* orientation for each dipeptide (Figure 4B). For example, a dipeptide composed of Ala and Ile would be either Ala-Ile or Ile-Ala. However, the y_1_ fragment would include either protonated Ile or Ala. As these are mutually exclusive, the presence of protonated Ile (Table S9 ion *b*) within the fragmentation pattern of this dipeptide unambiguously assigned it as Ala-Ile (Figure S67). Indeed, all X–Ile dipeptides shared this diagnostic y_1_ ion (Tables S9–11). As the isomeric dipeptides coeluted, the relative ratio of y_1_ ion intensities was used to approximate their individual contribution to the overall [M+H]^+^ EIC intensity (Figure 4B).

A total of 155 unique dipeptides were identified from the 148 positive reactions (Figures 4B&S63–S108, Tables S5–S50). These included all constitutive dipeptides for the 11 phosphonoalamide signals observed within crude cell extracts (Figure S23). To the best of our knowledge, PnaC exhibited the broadest substrate specificity of any biochemically characterized l-amino acid dipeptide ligase (Figures 4, S21–S47).^[27]^ All 20 proteinogenic amino acids were accepted as nucleophiles, while all but arginine and cysteine were accepted as carboxylates. Grouping amino acids based on their side-chain properties (nonpolar, aromatic, polar uncharged, basic, and acidic) revealed patterns of substrate specificity. PnaC synthesized dipeptides by pairing combinations of amino acids from within and between each group (e.g. aromatic + aromatic, nonpolar + polar, and acidic + basic) with the sole exception of aromatic + acidic products. Notably, PnaC was not restricted by dipeptide size (forming Gly-Gly and Trp-Trp), polarity (forming Phe-Phe and Lys-Lys), or charge (forming doubly positive Lys-Lys, doubly negative Asp-Asp, and mixed charge Glu-Lys dipeptides).

Our data suggest that properties of the activated carboxylate residue influence specificity of the selected nucleophilic amino acid. This is in agreement with the model for the ATP-grasp ligase MurD, where ligand binding induces conformational changes which alter enzyme affinity for subsequent ligands.^[28]^ The apoenzyme has high affinity for ATP and Mg^2+^, but low affinity for its substrates. Binding of ATP-Mg^2+^ triggers a conformational shift which increases affinity for UDP-*N-*acetylmuramoyl-l-Ala (UMA), the carboxylate substrate, but not d-Glu. Once UMA binds, ATP-hydrolysis allows for a final conformational shift, enhancing affinity for the d-Glu nucleophile.

Even small differences between carboxylates, such as the single stereocenter differentiating Thr and *allo*-Thr, resulted in altered product profiles. Notably, activation of Thr led to acceptance of Trp as a nucleophile, but not Thr or Pro. In contrast, activation of *allo*-Thr resulted in both Thr and Pro as nucleophiles, to the exclusion of Trp. These results suggest the likely sensitivity of PnaC to small conformational changes upon substrate binding.

For PnaC, activation of small nonpolar amino acids resulted in the greatest proportion of reactions which yielded product (78 of 147; 53.1%). Structurally, this suggests that the most permissive nucleophilic binding pockets result from activation of small, nonpolar carboxylate substrates. In comparison, activation of a basic amino acids resulted in the smallest proportion of reactions (2 of 63; 3.2%). Activation of aromatic (18 of 63; 28.6%), uncharged polar (42 of 126; 33.3%), and acidic (15 of 42; 35.7%) amino acids demonstrated intermediate success (Figure 4B). These preferences were also reflected in overall product yield. The most abundant dipeptides contained small nonpolar amino acids. Ala was clearly the most preferred substrate, yielding dipeptides with an average ion intensity nearly a magnitude larger than any other group (Figure 4).

Having established the diversity of dipeptides produced by PnaC, we probed the reaction specificity of PnaB. We used PnaC to generate substrates for ligation to PnAla by PnaB. Microscale reactions contained PnaB, PnaC, PnAla, and all pairs of amino acids shown to form dipeptides. LC-HRMS was used to identify potential PnAla-containing tripeptides from these 80 reactions (Figures 5A, S48–S62) and subsequent fragmentation analysis was used to verify these products and identify their orientation (Figures 5B, S109–S146; Tables S51–S88). For tripeptides, the b_1_ and b_2_ ions enabled identification of the residue forming an amide bond with PnAla. Again, coelution of isomers complicated their individual quantification, such that they were approximated using the relative ratio of b_2_ ions (Figure 5B). Altogether, our combined enzyme system produced 97 distinct PnAla-tripeptides, 93 of which had not been previously described (Figures 4&5).

**Figure 5.**
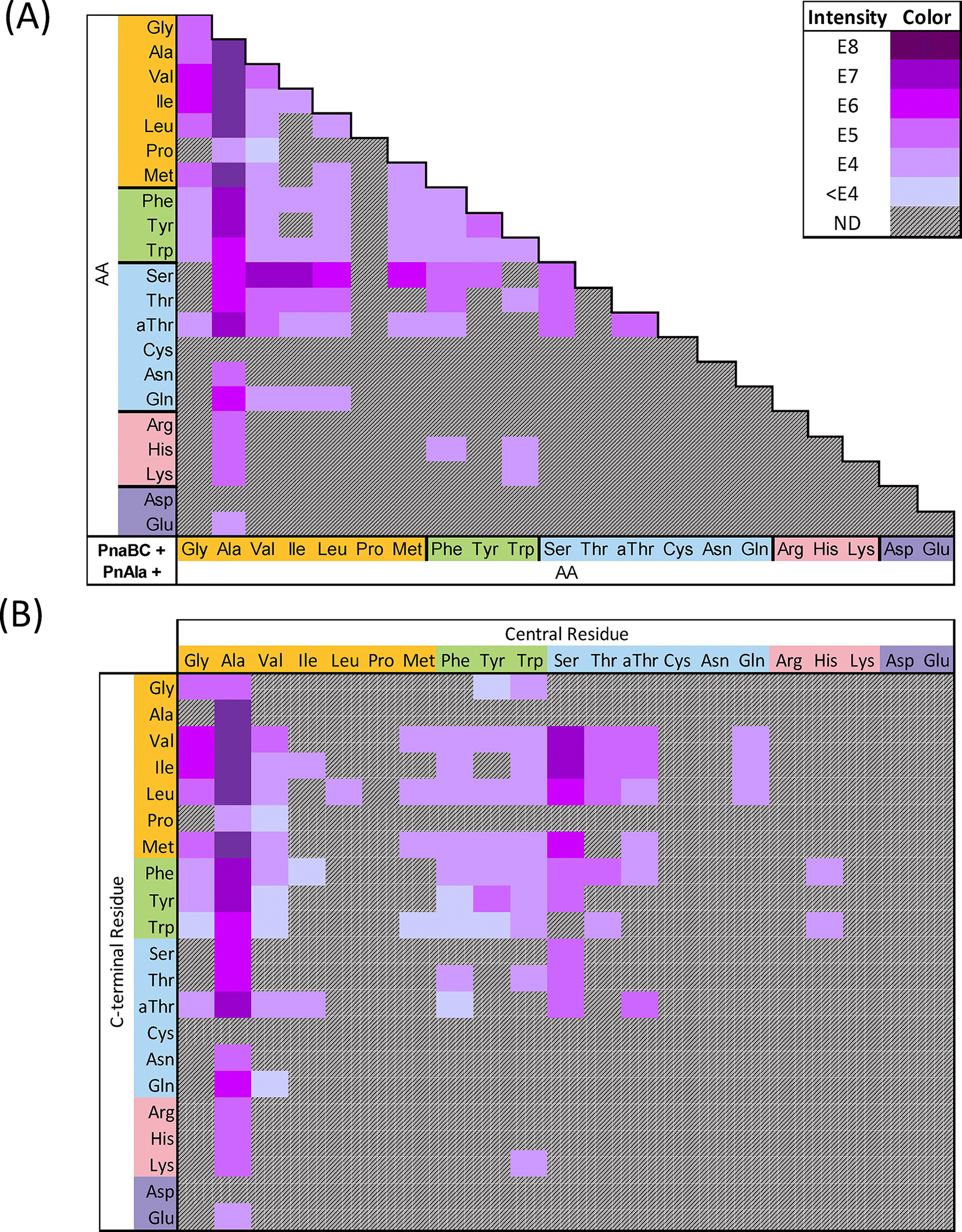
A) Summary of PnaBC reactions yielding product. Each cell indicates a single reaction utilizing PnAla and the corresponding amino acids. Cells are shaded based on the peak intensity of the LC-HRMS EIC for the respective product *m/z* (or not detected, ND). The key in the top right indicates the ion intensity represented by each colour. B) Subsequent LC-HRMS/MS fragmentation analysis was used to determine the arrangement of each tripeptide. Co-elution of tripeptide isomers complicated their individual quantification, such that the relative intensities of their b_2_ ions was used to calculate their contribution to the overall ion intensity.

Nearly half of the dipeptides produced by PnaC were accepted by PnaB as nucleophiles to form PnAla tripeptides. PnaB preferred dipeptides with smaller *N*-terminal side chains, while largely avoiding those bearing charges. All alanyl dipeptides, except for Ala-Cys and Ala-Asp, were ligated to PnAla. Tripeptides produced in the greatest abundance included PnAla conjugates of Ala-Ala, Ala-Val, Ala-Ile, Ala-Leu, and Ala-Met. Aversion for charged side chains was reflected by the limited number of PnAla tripeptides (7) that contained Arg, His, Lys, Asp, or Glu (Figures 5B&S147).

Our data also suggested that PnaB modulates the dipeptide synthetase activity of PnaC. Nearly a quarter of the PnAla tripeptides produced from one-pot reactions contained dipeptides that were not observed in reactions with PnaC alone (Figure 4B). This trend was observed among tripeptides containing Ala, Val, Ile, Met, Phe, Tyr, Trp, and His in the central position. The differences were most pronounced in PnaBC reactions containing Met and Trp, and Ala and Pro. To our surprise, PnaBC produced PnAla-Ala-Pro and PnAla-Val-Pro tripeptides even though only Pro-Ala and Pro-Val were detected in the PnaC-only reactions. Also unexpected was the production of PnAla-Trp-Met (major species) and PnAla-Met-Trp by the two-enzyme system. This contrasted with the PnaC-only reactions, where Met-Trp was detected as the sole product (Figures S123, S131). A total of 26 “hidden” dipeptides were revealed within our tripeptide synthesis data, raising the total number of dipeptides produced by PnaC to 181 (Figure 4B).

In these cases, we reasoned PnaB acts as a coupling enzyme for PnaC. For a given pair of amino acids, PnaC may have inherent preference for which is the activated carboxylate species and which performs nucleophilic attack. This may manifest as different ratios of the dipeptides, including those at concentrations below the limits of detection in our assays. However, selectivity of PnaB for ligating PnAla with the minor products (e.g. Trp-Met) would in turn drive their synthesis by PnaC. While the specificity of production of dipeptides by LALs can be shifted by modulating host amino acid flux,^[29]^ to the best of our knowledge this is the first example of an oligopeptide ligase directing the activity of a preceding dipeptide ligase within the same biosynthetic pathway.

Given the promiscuity of PnaB for nucleophilic substrates, we sought to determine whether it could accept additional carboxylates by incubating PnaB with Ala-Val and the proteinogenic isostere of PnAla, Asp. This reaction was analyzed by LC-HRMS (Figure S148A) and the putative tripeptide signal was confirmed by LC-HRMS/MS fragmentation to be Asp-Ala-Val (Figure S149, Table S89). Subsequent reactions were set up using a PnAla derivative with an extended side chain, l-2-amino-4-phosphonobutyrate (AP4), and Glu (its proteinogenic isostere) in place of Asp but yielded no product (Figure S148A). While the signal intensity for PnAla-Ala-Val and Asp-Ala-Val produced by PnaB was within the same magnitude, we did not observe Asp-Ala-Val within our heterologous expression samples (Figure S148B). Together, this suggests that PnaB is more selective for carboxylate substrates than it is for nucleophilic ones and prefers PnAla in vivo.

PnaB and PnaC add to the growing number of ATP-grasp amino acid ligases involved in phosphonopeptide biosynthesis. Both exhibited extremely broad specificity, producing more than 10 PnAla-tripeptides within strains and an additional 87 PnAla tripeptides by direct enzymatic synthesis. While there is a stark contrast in the number of products observed within strains and those observed in vitro, this likely reflects competition between substrates as well as their intracellular concentrations. Within Nature, ATP-grasp ligases may underly a strategy to produce multiple phosphonopeptides from one biosynthetic pathway. The rhizocticin, plumbemycin, and valinophos pathways encode ATP-grasp ligases and can produce multiple compounds all with the same Pn headgroup.^[6d,27e, f, 30]^ In contrast, phosphonopeptides biosynthesized using non-ribosomal peptide synthetase (phosphinothricin tripeptide, phosalacine)^[4b,31]^ or the tRNA-dependent GCN5-related *N*-acetyltransferase family enzymes (argolaphos, dehydrophos, fosfazinomycin)^[6e,32]^ are invariable in the amino acid composition of their products.

The ability to generate multiple phosphonopeptides may provide producing bacteria advantages against other competitor microorganisms sharing the same environmental niche. Phosphonopeptides commonly utilize a “Trojan horse” mechanism to manifest their bioactivity and organismal specificity. The composition of amino acids attached to the Pn moiety mediates recognition and import by different oligopeptide transporters, after which hydrolysis by endogenous peptidases releases the active Pn moiety.^[33]^ This is reflected in the rhizocticins and plumbemycins, which both contain the threonine synthase inhibitor (*Z*)-l-2-amino-5-phosphono-3-pentenoic acid, but exhibit selectivity as antifungal or antibacterial agents based upon the composition of ligated amino acids.^[26,30,33b,34]^ Within the phosphonoalamides, phosphonoalamide A (PnAla-Ala-Val, from *Streptomyces*) and phosphonoalamide F (Ala-Ala-PnAla, from *Bacillus*) display differing spectrums of antimicrobial activity.^[6b,c]^ In this manner, production of a diverse array of tripeptides may allow for an enhanced spectrum of antimicrobial activity as opposed to individual compounds. Complementarily, the versatility of PnaB and PnaC could also serve as a self-resistance mechanism. The incorporation of tabtoxinine-β-lactam into tabtoxin by TblF has been proposed as a mode of self-protection,^[35]^ and incorporation of PnAla into tripeptides would similarly provide a pathway for sequestering the toxic free Pn.

It has not escaped our attention that PnaC serves as an ideal starting point for the rational engineering of dipeptide ligases. To the best of our knowledge, PnaC has the broadest specificity of all biochemically characterized dipeptide ligases, accepting all proteinogenic amino acids as nucleophiles and all but Arg and Cys as carboxylates. PnaC produced numerous important dipeptides, including Ala-Gln, (used in patient infusion for nutrients), Leu-Ile (antidepressant effect), and Leu-Ser (savory flavor enhancer).^[27c]^ It is likely that only small changes would enable PnaC to accept Arg and Cys as carboxylates, as single mutations have been shown to significantly alter LAL specificity.^[36]^ A greater understanding of the molecular determinants of ligase substrate specificity will enable improved prediction of peptide NPs encoded by BGCs and empower their application as biocatalysts.

## Conclusion

Using a combination of comparative genomics, heterologous expression, gene deletion, and biochemical reconstitution experiments, we have elucidated the complete biosynthetic pathway for the *Streptomyces* phosphonoalamides. Of the 11 genes conserved between *Streptomyces* strains, only 4 encoded enzymes were essential for biosynthesis. The pathway begins with the isomerization of PEP to PnPy by PepM (PnaD), coupled to the immediate transamination of PnPy to PnAla by PnaA. Concurrently, PnaC ligates two amino acids to afford a wide variety of dipeptides. The pathway then converges, as PnaB ligates the PnAla produced by PnaA to a dipeptide produced by PnaC, resulting in a diverse array of phosphonoalamides. We note that the two enzymatic steps from PEP to PnAla represent the shortest biosynthetic pathway towards a bioactive Pn. The biosynthetic pathway for the *Streptomyces* phosphonoalamides establishes transamination of PnPy as a branch of Pn NP metabolism, which is broadly distributed among taxonomically diverse organisms and environments.^[6c]^ Peptide ligation reactions were employed to produce an extensive series of PnAla-containing phosphonopeptides, emphasizing the diversity of products resulting from a single BGC. More broadly, these findings highlight the wealth of PnAla-containing NPs which await discovery.

## Supplementary Material

Supporting Information

## Data Availability

The data that support the findings of this study are available in the supplementary material of this article.
