# Supplementary material for: Phosphonoalamides Reveal the Biosynthetic Origin of Phosphonoalanine Natural Products and a Convergent Pathway for Their Diversification
Source: Angew Chem Int Ed Engl. Author manuscript; Available in PMC 2025 Feb 27. (PMC11867202; doi:10.1002/anie.202405052)
Supplement: Supporting Information [file NIHMS2048962-supplement-Supporting_Information.pdf]

Angewandte Chemie
Eine Zeitschrift der Gesellschaft Deutscher Chemiker

Supporting Information

Phosphonoalamides Reveal the Biosynthetic Origin of Phosphonoalanine Natural Products and a Convergent Pathway for Their Diversification

*J. J. Cui, Y. Zhang, K.-S. Ju**

Supporting Information

Phosphonoalamides Reveal the Biosynthetic Origin of Phosphonoalanine Natural Products and a Convergent Pathway for Their Diversification

Jerry Cui[1,#], Yeying Zhang[1,#], and Kou-San Ju[*,1,2,3,4]

[1]Department of Microbiology, The Ohio State University, Columbus, OH 43210

[2]Division of Medicinal Chemistry and Pharmacognosy, The Ohio State University, Columbus, OH 43210

[3]Center for Applied Plant Sciences, The Ohio State University, Columbus, OH 43210

[4]Infectious Diseases Institute, The Ohio State University, Columbus, OH 43210

[#]Equal contribution

[*]Address correspondence to:

Kou-San Ju

Email: ju.109@osu.edu

Tel: 614-292-8847

Table of Contents

EXPERIMENTAL PROCEDURES 12

Chemicals 12

Strains, Media, General Culture Conditions 12

Molecular Biology 13

Genome sequencing, assembly, and annotation 13

Bioinformatic analyses 13

Construction and screening of a *Streptomyces* sp. S-515 fosmid library 13

Gene-deletions 14

Heterologous expression 14

Production of phosphonate natural products from native, heterologous expression, and deletion strains 14

NMR spectroscopy 14

Mass spectrometry 15

Expression and Purification of His_6-SUMO-PnaA 15

Expression and purification of His_6-PnaB 16

Expression and purification of His_6-PnaC 16

Expression and purification of His_6-PnaD 16

Expression and purification of His_6-VlpB 16

Expression and purification of His_6-MDH 17

Expression and purification of His_6-SerA 17

Biochemical assays of His_6-SUMO-PnaA with His_6-PnaD 17

Biochemical assays of His_6-SUMO-PnaA 17

Kinetic analyses 17

Biochemical assays of His_6-PnaB 19

Biochemical assays of His_6-PnaC 19

Tripeptide synthesis assays using PnaB and PnaC 19

Analysis of PnaC and PnaB substrate specificity reactions 19

SUPPLEMENTAL FIGURES 20

Figure S1. The *pepM* gene neighborhoods of strains containing the phosphonoalamide BGC 20

Figure S2. Synteny analysis between the phosphonoalamide gene neighborhood of S-515 and the genomes of strains lacking *pepM* 21
Figure S3. ^{1}H-^{31}P NMR spectra of *S. lividans* 66 *attB*::pKSJ588 extract 22
Figure S4. LC-HRMS analysis of phosphonates produced by heterologous expression strains. 23
Figure S5. SDS-PAGE of purified Pna proteins 24
Figure S6. ^{1}H-^{31}P HMBC of the PnaD-PnaA coupled reaction 25
Figure S7. ^{31}P NMR spectra of PnaD-PnaA reactions with different amino donors. 26
Figure S8. ^{1}H-^{31}P HMBC spectra of PnaA catalyzed transamination of OAA to Asp and PnAla to PnPy. 27
Figure S9. LC-HRMS analysis of PnaA catalyzed transamination reactions. 28
Figure S10. LC-HRMS analysis of derivatized PnaA catalyzed transamination reactions 29
Figure S11. Timecourse of PnPy formation in the PnaA-catalyzed transamination of PnAla to PnPy using OAA as the keto-acid acceptor 30
Figure S12. Timecourse of PnAla formation in the PnaA-catalyzed transamination of Asp to OAA using PnPy as the keto-acid acceptor 31
Figure S13. Transamination reactions with varied amounts of Asp or OAA after 2 h 32
Figure S14. Kinetic analyses for the conversion of PnPy and L-Asp to L-PnAla and OAA by PnaA. 33
Figure S15. Kinetic analyses for the conversion of L-PnAla and OAA to PnPy and L-Asp by PnaA. 34
Figure S16. Kinetic analyses for the PEP conversion to L-PnAla in a coupled reaction with PnaD and PnaA. 35
Figure S17. Kinetic analyses of αKG conversion to 2-hydroxyglutarate by 3-phosphoglycerate dehydrogenase SerA 36
Figure S18. Kinetic analyses for the conversion of OAA to malate by 3-phosphoglycerate dehydrogenase SerA 37
Figure S19. Kinetic analyses for the conversion of L-Glu and OAA to αKG and L-Asp by PnaA 38
Figure S20. Kinetic analyses for the conversion of αKG and L-Asp to L-Glu and OAA to by PnaA 39
Figure S21. LC-MS analyses of PnaB and PnaC reactions for dipeptide ligation 40
Figure S22. LC-MS analyses of PnaB ligation reactions with chemically synthesized dipeptides 41

Figure S23. LC-HRMS detection of PnAla and phosphonoalamides within culture extracts of *S. lividans* 66 *attB*::pKSJ595 42
Figure S24. LC-HRMS detection of X-Ala dipeptides synthesized in PnaC reactions 43
Figure S25. LC-HRMS detection of X-Val dipeptides synthesized in PnaC reactions 44
Figure S26. LC-HRMS detection of X-Ile dipeptides synthesized in PnaC reactions 45
Figure S27. LC-HRMS detection of X-Leu dipeptides synthesized in PnaC reactions 46
Figure S28. LC-HRMS detection of X-Met dipeptides synthesized in PnaC reactions 47
Figure S29. LC-HRMS detection of X-Phe dipeptides synthesized in PnaC reactions 48
Figure S30. LC-HRMS detection of X-Tyr dipeptides synthesized in PnaC reactions 49
Figure S31. LC-HRMS detection of X-Trp dipeptides synthesized in PnaC reactions 50
Figure S32. LC-HRMS detection of X-Ser dipeptides synthesized in PnaC reactions 51
Figure S33. LC-HRMS detection of X-Thr dipeptides synthesized in PnaC reactions 52
Figure S34. LC-HRMS detection of X-aThr dipeptides synthesized in PnaC reactions 53
Figure S35. LC-HRMS detection of X-Asn dipeptides synthesized in PnaC reactions 54
Figure S36. LC-HRMS detection of X-Gln dipeptides synthesized in PnaC reactions 55
Figure S37. LC-HRMS detection of X-Arg dipeptides synthesized in PnaC reactions 56
Figure S38. LC-HRMS detection of X-Arg dipeptides synthesized in PnaC reactions, cntd. 57
Figure S39. LC-HRMS detection of X-His dipeptides synthesized in PnaC reactions 58
Figure S40. LC-HRMS detection of X-His dipeptides synthesized in PnaC reactions, cntd. 59
Figure S41. LC-HRMS detection of X-Lys dipeptides synthesized in PnaC reactions 60
Figure S42. LC-HRMS detection of X-Lys dipeptides synthesized in PnaC reactions, cntd. 61
Figure S43. LC-HRMS detection of X-Asp dipeptides synthesized in PnaC reactions 62
Figure S44. LC-HRMS detection of X-Glu dipeptides synthesized in PnaC reactions 63
Figure S45. LC-HRMS detection of X-Cys dipeptides synthesized in PnaC reactions 64
Figure S46. LC-HRMS detection of X-Gly dipeptides synthesized in PnaC reactions 65
Figure S47. LC-HRMS detection of X-Pro dipeptides synthesized in PnaC reactions 66
Figure S48. LC-HRMS detection of PnAla-Ala-X tripeptides synthesized in PnaBC reactions ... 67
Figure S49. LC-HRMS detection of PnAla-Ala-X tripeptides synthesized in PnaBC reactions, cntd. 68
Figure S50. LC-HRMS detection of PnAla-Val-X tripeptides synthesized in PnaBC reactions ... 69

Figure S51. LC-HRMS detection of PnAla-Ile-X tripeptides synthesized in PnaBC reactions, cntd. 70
Figure S52. LC-HRMS detection of PnAla-Leu-X tripeptides synthesized in PnaBC reactions .. 71
Figure S53. LC-HRMS detection of PnAla-Met-X tripeptides synthesized in PnaBC reactions . 72
Figure S54. LC-HRMS detection of PnAla-Phe-X tripeptides synthesized in PnaBC reactions .. 73
Figure S55. LC-HRMS detection of PnAla-Tyr-X tripeptides synthesized in PnaBC reactions ... 74
Figure S56. LC-HRMS detection of PnAla-Trp-X tripeptides synthesized in PnaBC reactions ... 75
Figure S57. LC-HRMS detection of PnAla-Ser-X tripeptides synthesized in PnaBC reactions ... 76
Figure S58. LC-HRMS detection of PnAla-Thr-X tripeptides synthesized in PnaBC reactions ... 77
Figure S59. LC-HRMS detection of PnAla-aThr-X tripeptides synthesized in PnaBC reactions . 78
Figure S60. LC-HRMS detection of PnAla-Gln-X tripeptides synthesized in PnaBC reactions ... 79
Figure S61. LC-HRMS detection of PnAla-His-X tripeptides synthesized in PnaBC reactions ... 80
Figure S62. LC-HRMS detection of PnAla-Gly-X tripeptides synthesized in PnaBC reactions ... 81
Figure S63. LC-MS/MS fragmentation of Ala-Ala, Gly-Ala, and Pro-Ala 82
Figure S64. LC-MS/MS fragmentation of Ala-Val, Val-Val, Leu-Val, and Ser-Val 83
Figure S65. LC-MS/MS fragmentation of Thr-Val, aThr-Val, Asn-Val, and Gln-Val 84
Figure S66. LC-MS/MS fragmentation of Asp-Val, Glu-Val, Gly-Val, and Pro-Val 85
Figure S67. LC-MS/MS fragmentation of Ala-Ile, Val-Ile, Ile-Ile, and Leu-(^{15}N-Ile) 86
Figure S68. LC-MS/MS fragmentation of Ser-Ile, Thr-Ile, aThr-Ile, and Gln-Ile 87
Figure S69. LC-MS/MS fragmentation of Asp-Ile, Gly-Ile, and Pro-Ile 88
Figure S70. LC-MS/MS fragmentation of Ala-Leu, Val-Leu, (^{15}N-Ile)-Leu, and Leu-Leu 89
Figure S71. LC-MS/MS fragmentation of Ser-Leu, Thr-Leu, aTHr-Leu, and Gln-Leu 90
Figure S72. LC-MS/MS fragmentation of Gly-Leu and Pro-Leu 91
Figure S73. LC-MS/MS fragmentation of Ala-Met, Val-Met, Leu-Met, and Met-Met 92
Figure S74. LC-MS/MS fragmentation of Phe-Met, Tyr-Met, Ser-Met, and Thr-Met 93
Figure S75. LC-MS/MS fragmentation of aThr-Met, Gly-Met, and Pro-Met 94
Figure S76. LC-MS/MS fragmentation of Ala-Phe, Val-Phe, Ile-Phe, and Leu-Phe 95
Figure S77. LC-MS/MS fragmentation of Met-Phe, Phe-Phe, Tyr-Phe, and Ser-Phe 96
Figure S78. LC-MS/MS fragmentation of Thr-Phe, aThr-Phe, Gly-Phe, and Pro-Phe 97
Figure S79. LC-MS/MS fragmentation of Ala-Tyr, Val-Tyr, and Leu-Tyr 98
Figure S80. LC-MS/MS fragmentation of Met-Tyr, Phe-Tyr, and Tyr-Tyr 99

Figure S81. LC-MS/MS fragmentation of Ser-Tyr, Gly-Tyr, and Pro-Tyr 100
Figure S82. LC-MS/MS fragmentation of Ala-Trp, Val-Trp, Ile-Trp, and Leu-Trp 101
Figure S83. LC-MS/MS fragmentation of Met-Trp, Phe-Trp, Tyr-Trp, and Trp-Trp 102
Figure S84. LC-MS/MS fragmentation of Ser-Trp, Thr-Trp, Gly-Trp, and Pro-Trp 103
Figure S85. LC-MS/MS fragmentation of Ala-Ser, Ser-Ser, Glu-Ser, and Gly-Ser 104
Figure S86. LC-MS/MS fragmentation of Ala-Thr, Ser-Thr, Gly-Thr, and Pro-Thr 105
Figure S87. LC-MS/MS fragmentation of aThr-(4-^{13}C-2,3-D_2-Thr) 106
Figure S88. LC-MS/MS fragmentation of Ala-aThr, Ser-aThr, (4-^{13}C-2,3-D_2-Thr)-aThr, and aThr-aThr 107
Figure S89. LC-MS/MS fragmentation of Glu-aThr and Gly-aThr 108
Figure S90. LC-MS/MS fragmentation of Ala-Asn, Ser-Asn, Asp-Asn, and Pro-Asn 109
Figure S91. LC-MS/MS fragmentation of Ala-Gln, Ser-Gln, Glu-Gln, and Pro-Gln 110
Figure S92. LC-MS/MS fragmentation of Ala-Arg, Val-Arg, Ile-Arg, and Leu-Arg 111
Figure S93. LC-MS/MS fragmentation of Met-Arg, Phe-Arg, Tyr-Arg, and Trp-Arg 112
Figure S94. LC-MS/MS fragmentation of Ser-Arg, Thr-Arg, aThr-Arg, and Gln-Arg 113
Figure S95. LC-MS/MS fragmentation of Asp-Arg, Gly-Arg, and Pro-Arg 114
Figure S96. LC-MS/MS fragmentation of Ala-His, Val-His, Ile-His, and Leu-His 115
Figure S97. LC-MS/MS fragmentation of Phe-His, Tyr-His, Trp-His, and Ser-His 116
Figure S98. LC-MS/MS fragmentation of Thr-His, aThr-His, Asp-His, and Glu-His 117
Figure S99. LC-MS/MS fragmentation of Gly-His and Pro-His 118
Figure S100. LC-MS/MS fragmentation of Ala-Lys, Ile-Lys, Leu-Lys, and Phe-Lys 119
Figure S101. LC-MS/MS fragmentation of Tyr-Lys, Trp-Lys, Ser-Lys, and Thr-Lys 120
Figure S102. LC-MS/MS fragmentation of aThr-Lys, Asn-Lys, Lys-Lys, and Asp-Lys 121
Figure S103. LC-MS/MS fragmentation of Glu-Lys, Gly-Lys, and Pro-Lys 122
Figure S104. LC-MS/MS fragmentation of Asp-Asp 123
Figure S105. LC-MS/MS fragmentation of Ala-Glu and Glu-Glu 124
Figure S106. LC-MS/MS fragmentation of Ala-Cys 125
Figure S107. LC-MS/MS fragmentation of Glu-Gly and Gly-Gly... 126
Figure S108. LC-MS/MS fragmentation of aThr-Pro and His-Pro 127
Figure S109. LC-MS/MS fragmentation of PnAla-Ala-Ala, PnAla-Ala-Val, and PnAla-Ala-Ile .. 128

Figure S110. LC-MS/MS fragmentation of PnAla-Ala-Leu, PnAla-Ala-Met, and PnAla-Ala-Phe 129
Figure S111. LC-MS/MS fragmentation of PnAla-Ala-Tyr, PnAla-Ala-Trp, and PnAla-Ala-Ser 130
Figure S112. LC-MS/MS fragmentation of PnAla-Ala-Thr, PnAla-Ala-aThr, and PnAla-Ala-Asn 131
Figure S113. LC-MS/MS fragmentation of PnAla-Ala-Gln, PnAla-Ala-Arg, and PnAla-Ala-His 132
Figure S114. LC-MS/MS fragmentation of PnAla-Ala-Lys, PnAla-Ala-Glu, and PnAla-Ala-Gly 133
Figure S115. LC-MS/MS fragmentation of PnAla-Ala-Pro 134
Figure S116. LC-MS/MS fragmentation of PnAla-Val-Val, PnAla-Val-Ile, and PnAla-Val-Leu . 135
Figure S117. LC-MS/MS fragmentation of PnAla-Val-Met, PnAla-Val-Phe, and PnAla-Val-Tyr 136
Figure S118. LC-MS/MS fragmentation of PnAla-Val-Trp, PnAla-Val-aThr, and PnAla-Val-Gln 137
Figure S119. LC-MS/MS fragmentation of PnAla-Val-Pro 138
Figure S120. LC-MS/MS fragmentation of PnAla-Ile-Ile, PnAla-Ile-Phe, and PnAla-Ile-aThr .. 139
Figure S121. LC-MS/MS fragmentation of PnAla-Leu-Leu 140
Figure S122. LC-MS/MS fragmentation of PnAla-Met-Val, PnAla-Met-Leu, and PnAla-Met-Met 141
Figure S123. LC-MS/MS fragmentation of PnAla-Met-Trp 142
Figure S124. LC-MS/MS fragmentation of PnAla-Phe-Val, PnAla-Phe-Ile, and PnAla-Phe-Leu 143
Figure S125. LC-MS/MS fragmentation of PnAla-Phe-Met, PnAla-Phe-Phe, and PnAla-Phe-Tyr 144
Figure S126. LC-MS/MS fragmentation of PnAla-Phe-Trp, PnAla-Phe-Thr, and PnAla-Phe-aThr 145
Figure S127. LC-MS/MS fragmentation of PnAla-Tyr-Val, PnAla-Tyr-Leu, and PnAla-Tyr-Met 146
Figure S128. LC-MS/MS fragmentation of PnAla-Tyr-Phe, PnAla-Tyr-Tyr, and PnAla-Tyr-Trp 147
Figure S129. LC-MS/MS fragmentation of PnAla-Tyr-Gly 148
Figure S130. LC-MS/MS fragmentation of PnAla-Trp-Val, PnAla-Trp-Ile, and PnAla-Trp-Leu 149
Figure S131. LC-MS/MS fragmentation of PnAla-Trp-Met, PnAla-Trp-Phe, and PnAla-Trp-Tyr 150
Figure S132. LC-MS/MS fragmentation of PnAla-Trp-Trp, PnAla-Trp-Thr, and PnAla-Trp-Lys 151

Figure S133. LC-MS/MS fragmentation of PnAla-Trp-Gly 152
Figure S134. LC-MS/MS fragmentation of PnAla-Ser-Val and PnAla-Ser-Ile 153
Figure S135. LC-MS/MS fragmentation of PnAla-Ser-Leu and PnAla-Ser-Met 154
Figure S136. LC-MS/MS fragmentation of PnAla-Ser-Phe and PnAla-Ser-Tyr 155
Figure S137. LC-MS/MS fragmentation of PnAla-Ser-Ser, PnAla-Ser-Thr, and PnAla-Ser-aThr 156
Figure S138. LC-MS/MS fragmentation of PnAla-Thr-Val, PnAla-Thr-Ile, and PnAla-Thr-Leu 157
Figure S139. LC-MS/MS fragmentation of PnAla-Thr-Phe and PnAla-Thr-Trp 158
Figure S140. LC-MS/MS fragmentation of PnAla-aThr-Val, PnAla-aThr-Ile, and PnAla-aThr-Leu 159
Figure S141. LC-MS/MS fragmentation of PnAla-aThr-Met, PnAla-aThr-Phe, and PnAla-aThr-aThr 160
Figure S142. LC-MS/MS fragmentation of PnAla-Gln-Val, PnAla-Gln-Ile, and PnAla-Gln-Leu 161
Figure S143. LC-MS/MS fragmentation of PnAla-His-Phe and PnAla-His-Trp 162
Figure S144. LC-MS/MS fragmentation of PnAla-Gly-Val, PnAla-Gly-Ile, and PnAla-Gly-Leu . 163
Figure S145. LC-MS/MS fragmentation of PnAla-Gly-Met, PnAla-Gly-Phe, and PnAla-Gly-Tyr 164
Figure S146. LC-MS/MS fragmentation of PnAla-Gly-Trp, PnAla-Gly-aThr, and PnAla-Gly-Gly 165
Figure S147. All N-terminal L-PnAla-containing tripeptides produced by PnaBC 166
Figure S148. PnaB carboxylate specificity 167
Figure S149. LC-HRMS/MS fragmentation of Asp-Ala-Val 168
Figure S150. Impact of ion suppression on EIC spectra 169
Figure S151. Background noise in EIC spectra 170
Figure S152. Contaminating parent ions in the selection window 171
SUPPLEMENTAL TABLES 172
Table S1. Annotation of the S-515 *pepM* neighborhood 172
Table S2. List of strains used in this study 173
Table S3. List of plasmids used in this study 174
Table S4. Primers used in this study 175
Table S5. Ala-Ala, Gly-Ala, and Pro-Ala dipeptide fragments 176
Table S6. Ala-Val, Val-Val, Leu-Val, and Ser-Val dipeptide fragments 177

Table S7. Thr-Val, aThr-Val, Asn-Val, and Gln-Val dipeptide fragments 178
Table S8. Asp-Val, Glu-Val, Gly-Val, and Pro-Val dipeptide fragments 179
Table S9. Ala-Ile, Val-Ile, Ile-Ile, and Leu-^{15}N-Ile dipeptide fragments 180
Table S10. Ser-Ile, Thr-Ile, aThr-Ile, and Gln-Ile dipeptide fragments 181
Table S11. Asp-Ile, Gly-Ile, and Pro-Ile dipeptide fragments 182
Table S12. Ala-Leu, Val-Leu, ^{15}N-Ile-Leu, and Leu-Leu dipeptide fragments 183
Table S13. Ser-Leu, Thr-Leu, aThr-Leu, and Gln-Leu dipeptide fragments 184
Table S14. Gly-Leu and Pro-Leu dipeptide fragments 185
Table S15. Ala-Met, Val-Met, Leu-Met, and Met-Met dipeptide fragments 186
Table S16. Phe-Met, Tyr-Met, Ser-Met, and Thr-Met dipeptide fragments 187
Table S17. aThr-Met, Gly-Met, and Pro-Met dipeptide fragments 188
Table S18. Ala-Phe, Val-Phe, Ile-Phe, and Leu-Phe dipeptide fragments 189
Table S19. Met-Phe, Phe-Phe, Tyr-Phe, and Ser-Phe dipeptide fragments 190
Table S20. Thr-Phe, aThr-Phe, Gly-Phe, and Pro-Phe dipeptide fragments 191
Table S21. Ala-Tyr, Val-Tyr, and Leu-Tyr dipeptide fragments 192
Table S22. Met-Tyr, Phe-Tyr, and Tyr-Tyr dipeptide fragments 193
Table S23. Ser-Tyr, Gly-Tyr, and Pro-Tyr dipeptide fragments 194
Table S24. Ala-Trp, Val-Trp, Ile-Trp, and Leu-Trp dipeptide fragments 195
Table S25. Met-Trp, Phe-Trp, Tyr-Trp, and Trp-Trp dipeptide fragments 196
Table S26. Ser-Trp, Thr-Trp, Gly-Trp, and Pro-Trp dipeptide fragments 197
Table S27. Ala-Ser, Ser-Ser, Glu-Ser, and Gly-Ser dipeptide fragments 199
Table S28. Ala-Thr, Ser-Thr, Gly-Thr, and Pro-Thr dipeptide fragments 200
Table S29. aThr-(4-^{13}C; 2,3-D_2 Thr) dipeptide fragments 201
Table S30. Ala-aThr, Ser-aThr, (4-^{13}C; 2,3-D_2 Thr)-aThr, and aThr-aThr dipeptide fragments 202
Table S31. Glu-aThr and Gly-aThr dipeptide fragments 203
Table S32. Ala-Asn, Ser-Asn, Asp-Asn, and Pro-Asn dipeptide fragments 204
Table S33. Ala-Gln, Ser-Gln, Glu-Gln, and Pro-Gln dipeptide fragments 205
Table S34. Ala-Arg, Val-Arg, Ile-Arg, and Leu-Arg dipeptide fragments 206
Table S35. Met-Arg, Phe-Arg, Tyr-Arg, and Trp-Arg dipeptide fragments 207
Table S36. Ser-Arg, Thr-Arg, aThr-Arg, and Gln-Arg dipeptide fragments 208
Table S37. Asp-Arg, Gly-Arg, and Pro-Arg dipeptide fragments 209

Table S38. Ala-His, Val-His, Ile-His, and Leu-His dipeptide fragments 210
Table S39. Phe-His, Tyr-His, Trp-His, and Ser-His dipeptide fragments 211
Table S40. Thr-His, aThr-His, Asp-His, and Glu-His dipeptide fragments 212
Table S41. Gly-His and Pro-His dipeptide fragments 213
Table S42. Ala-Lys, Ile-Lys, Leu-Lys, and Phe-Lys dipeptide fragments 214
Table S43. Tyr-Lys, Trp-Lys, Ser-Lys, and Thr-Lys dipeptide fragments 215
Table S44. aThr-Lys, Asn-Lys, Lys-Lys, and Asp-Lys dipeptide fragments 216
Table S45. Glu-Lys, Gly-Lys, and Pro-Lys dipeptide fragments 217
Table S46. Asp-Asp dipeptide fragments 218
Table S47. Ala-Glu and Glu-Glu dipeptide fragments 219
Table S48. Ala-Cys dipeptide fragments 220
Table S49. Glu-Gly and Gly-Gly dipeptide fragments 221
Table S50. aThr-Pro and His-Pro dipeptide fragments 222
Table S51. PnAla-Ala-Ala, PnAla-Ala-Val, and PnAla-Ala-Ile tripeptide fragments 223
Table S52. PnAla-Ala-Leu, PnAla-Ala-Met, and PnAla-Ala-Phe tripeptide fragments 224
Table S53. PnAla-Ala-Tyr, PnAla-Ala-Trp, and PnAla-Ala-Ser tripeptide fragments 225
Table S54. PnAla-Ala-Thr, PnAla-Ala-aThr, and PnAla-Ala-Asn tripeptide fragments 226
Table S55. PnAla-Ala-Gln, PnAla-Ala-Arg, and PnAla-Ala-His tripeptide fragments 227
Table S56. PnAla-Ala-Lys, PnAla-Ala-Glu, and PnAla-Ala-Gly tripeptide fragments 228
Table S57. PnAla-Ala-Pro tripeptide fragments 229
Table S58. PnAla-Val-Val, PnAla-Val-Ile, and PnAla-Val-Leu tripeptide fragments 230
Table S59. PnAla-Val-Met, PnAla-Val-Phe, and PnAla-Val-Tyr tripeptide fragments 231
Table S60. PnAla-Val-Trp, PnAla-Val-aThr, and PnAla-Val-Gln tripeptide fragments 232
Table S61. PnAla-Val-Pro tripeptide fragments 233
Table S62. PnAla-Ile-Ile, PnAla-Ile-Phe, and PnAla-Ile-aThr tripeptide fragments 234
Table S63. PnAla-Leu-Leu tripeptide fragments 235
Table S64. PnAla-Met-Val, PnAla-Met-Leu, and PnAla-Met-Met tripeptide fragments 236
Table S65. PnAla-Met-Trp tripeptide fragments 237
Table S66. PnAla-Phe-Val, PnAla-Phe-Ile, and PnAla-Phe-Leu tripeptide fragments 238
Table S67. PnAla-Phe-Met, PnAla-Phe-Phe, and PnAla-Phe-Tyr tripeptide fragments 239
Table S68. PnAla-Phe-Trp, PnAla-Phe-Thr, and PnAla-Phe-aThr tripeptide fragments 240

Table S69. PnAla-Tyr-Val, PnAla-Tyr-Leu, and PnAla-Tyr-Met tripeptide fragments 241
Table S70. PnAla-Tyr-Phe, PnAla-Tyr-Tyr, and PnAla-Tyr-Trp tripeptide fragments 242
Table S71. PnAla-Tyr-Gly tripeptide fragments 243
Table S72. PnAla-Trp-Val, PnAla-Trp-Ile, and PnAla-Trp-Leu tripeptide fragments 244
Table S73. PnAla-Trp-Met, PnAla-Trp-Phe, and PnAla-Trp-Tyr tripeptide fragments 245
Table S74. PnAla-Trp-Trp, PnAla-Trp-Thr, and PnAla-Trp-Lys tripeptide fragments 246
Table S75. PnAla-Trp-Gly tripeptide fragments 247
Table S76. PnAla-Ser-Val and PnAla-Ser-Ile tripeptide fragments 248
Table S77. PnAla-Ser-Leu and PnAla-Ser-Met tripeptide fragments 249
Table S78. PnAla-Ser-Phe and PnAla-Ser-Tyr tripeptide fragments 250
Table S79. PnAla-Ser-Ser, PnAla-Ser-Thr, and PnAla-Ser-aThr tripeptide fragments 251
Table S80. PnAla-Thr-Val, PnAla-Thr-Ile, and PnAla-Thr-Leu tripeptide fragments 252
Table S81. PnAla-Thr-Phe and PnAla-Thr-Trp tripeptide fragments 254
Table S82. PnAla-aThr-Val, PnAla-aThr-Ile, and PnAla-aThr-Leu tripeptide fragments 255
Table S83. PnAla-aThr-Met, PnAla-aThr-Phe, and PnAla-aThr-aThr tripeptide fragments 257
Table S84. PnAla-Gln-Val, PnAla-Gln-Ile, and PnAla-Gln-Leu tripeptide fragments 258
Table S85. PnAla-His-Phe and PnAla-His-Trp tripeptide fragments 259
Table S86. PnAla-Gly-Val, PnAla-Gly-Ile, and PnAla-Gly-Leu tripeptide fragments 260
Table S87. PnAla-Gly-Met, PnAla-Gly-Phe, and PnAla-Gly-Tyr tripeptide fragments 261
Table S88. PnAla-Gly-Trp, PnAla-Gly-aThr, and PnAla-Gly-Gly tripeptide fragments 263
Table S89. Asp-Ala-Val tripeptide fragments 264
REFERENCES 265

EXPERIMENTAL PROCEDURES

Chemicals

General chemical reagents were purchased from Sigma-Aldrich, Fisher Scientific, VWR, or Santa Cruz Biotechnology. Stable isotopes were purchased from Cambridge Isotope Laboratories.

Strains, Media, General Culture Conditions

The strains and plasmids used in this study are listed in Supplemental Tables S2 and S3. *Escherichia coli* strains were routinely grown on LB broth or agar at 37 °C. *Streptomyces* strains were grown at 30 °C. The following additives and antibiotics were included for plasmid maintenance and selection as appropriate: 20 µg mL^{-1} 2,6-diaminopimelic acid (DAP), 25 µg mL^{-1} kanamycin (Km), 100 µg mL^{-1} ampicillin (Amp), 15 µg mL^{-1} chloramphenicol (Clm), 25 µg mL^{-1} apramycin (Apr). All components were dissolved in deionized water (dI). For plates, 16 g agar was added per liter of media. All media formulations are given per liter.

ATCC 172: 20 g Soluble starch (potato), 10 g glucose, 5 g yeast extract, 5 g N-Z Amine Type A, 1 g $CaCO_3$. Adjusted to pH 7.3 prior to autoclaving.

Balch's vitamins solution:[1] 5 mg *p*-Aminobenzoic acid, 2 mg folic acid, 2 mg biotin, 5 mg nicotinic acid, 5 mg calcium pantothenate, 5 mg riboflavin, 5 mg thiamine HCl, 10 mg pyridoxine HCl (B6), 100 µg cyanocobalamin (B12), 5 mg thioctic acid (lipoic acid). Adjusted pH to 7.0 with 1M NaOH. Filter sterilized.

GUBC: 10 g Sucrose, 5 g beef extract, 5 g Casamino acids, 10 mL 50% glycerol solution (w/v), 5 mL 1 M Na_2HPO_4-KH_2PO_4 buffer (pH 7.3), 2 mL Hunter's Concentrated Base. 10 mL of filter-sterilized Balch's vitamins added after autoclaving.

Hunter's Concentrated Base:[2] 20 g Nitrilotriacetic acid, 14 g KOH, 59.3 g $MgSO_4$-$7H_2O$, 6.67 g $CaCl_2$-$2H_2O$, 18.5 mg $(NH_4)_6Mo_7O_{24}$-$4H_2O$, 0.198 g $FeSO_4$-$7H_2O$, 100 mL Hunter's Metals 44. Adjusted pH to 6.8 with 10M KOH.

Hunter's Metals 44: 2.5 g EDTA (free acid), 10.95 g $ZnSO_4$-$7H_2O$, 5 g $FeSO_4$-H_2O, 1.54 g $MnSO_4$-H_2O, 0.392 g $CuSO_4$-$5H_2O$, 0.25 g $Co(NO_3)_2$-$6H_2O$, 0.177 g $Na_2B_4O_7$-$10H_2O$. Acidified with 5 drops of sulfuric acid.

LB: 10 g Tryptone, 5 g yeast extract, 5 g NaCl.

ISP2: 10 g Malt extract, 4 g yeast extract, 4 g glucose.

ISP4: 10 g Soluble starch (potato), 2g $(NH_4)_2SO_4$, 2g $CaCO_3$, 1 g K_2HPO_4, 1 g $MgSO_4$-$7H_2O$, 1 mg $FeSO_4$-$7H_2O$, 1 mg $ZnSO_4$-$7H_2O$, 1 mg $MnCl_2$-$2H_2O$.

M9: 12.8 g Na_2HPO_4-$7H_2O$, 3g KH_2PO_4, 1 g NH_4Cl, 0.5 g NaCl. $MgSO_4$ (2 mL) and $CaCl_2$ (100 µL) were added from 1 M filter-sterilized stock solutions after autoclaving.

MS1: 10 g Mannitol, 10 g roasted soy flour.

R2AS: 0.5 g Yeast extract, 0.5 g peptone, 0.5 g Casamino acids, 0.5 g glucose, 0.5 g soluble starch (potato), 0.3 g sodium pyruvate, 0.3 g K_2HPO_4, 0.05 g of $MgSO_4$-$7H_2O$. 10 mL sterile Balch's Vitamins and 20 mL sterile 1M sodium succinate were added after autoclaving.

Molecular Biology

DNA manipulations were performed according to standard methods[3] and manufacturer protocols. Lysozyme, achromapeptidase, and RNase A were from Sigma-Aldrich, restriction endonucleases and recombinant shrimp alkaline phosphatase (SAP) were from New England Biolabs (Beverly, MA). Plasmids were routinely purified from using Zymopure miniprep or midiprep kits (Zymo Research, Irvine, CA). Genomic DNA was isolated from *Streptomyces* spp. as previously described.[4]

PCR reactions to generate DNA fragments for cloning were performed using Phusion or Q5 DNA polymerase, whereas PCR reactions for verification of strain constructions and fosmid library screening used OneTaq DNA polymerase (New England Biolabs). DNA fragments for cloning were purified from agarose gel slices using a Zymoclean DNA extraction kit (Zymo Research). The HiFi DNA assembly mix (New England Biolabs) was used for Gibson assembly except only 25% of each component was used per reaction. Oligonucleotides (Life Technologies, Carlsbad, CA) are listed in Table S4. Sanger sequencing was performed at the Ohio State University Comprehensive Cancer Center Genomics Shared Resource facility.

Genome sequencing, assembly, and annotation

Genomic DNA isolated from *Streptomyces* sp. strains NRRL B-2790 and S-448 was sequenced at SeqCenter (Pittsburgh, PA) using Oxford Nanopore and Illumina technologies. Genomes were assembled with Unicycler,[5] annotated using PGAP,[6] and deposited in NCBI within Bioproject PRJNA1021995, under the accessions JBAFSW000000000 (B-2790) and JBAISJ000000000 (S-448). The phosphonoalamide BGC from *Streptomyces* sp. NRRL S-515 was deposited in NCBI under the accession PP239091.

Bioinformatic analyses

Gene neighborhoods were annotated by NCBI Blast, Pfam, and CDD.[7] Synteny were analyzed based on the gene cluster similarity comparisons performed using Easyfig.[8]

Construction and screening of a *Streptomyces* sp. S-515 fosmid library

High-molecular weight genomic DNA was isolated from *Streptomyces* sp. S-515 and used to construct a fosmid library as previously described.[4] The library was screened for *pepM* using primers S515-pepM-screen-F and S515-pepM-screen-R. Two fosmids, pKSJ546 and pKSJ549, were selected for further analysis. These were recombined in-vitro with pAE4 using BP Clonase II (Invitrogen, Carlsbad, CA) to yield pKSJ553 and pKSJ554.

Gene-deletions

Gene deletions were constructed using the λ Red recombination method of Datsenko and Wanner as previously described.[4, 9] Briefly, PCR primers were designed to amplify an included sequence homologous to the upstream and downstream of each target (Table S4). All primers were designed to amplify the kanamycin resistance cassette from plasmid pAE5.[10] This cassette includes a synthetic promoter to ensure expression of downstream genes. Resulting fosmids (pKSJ588, 595, 596) were verified with diagnostic restriction digestion analysis and Sanger sequencing using primers aph-seq-up and aph-seq-down to validate constructs. These primers bind within the Km^R cassette and produce sequencing reads that span outwards across the designed junctions.

Heterologous expression

Plasmid constructs were introduced for integration at the phiC31 *attB* locus of *S. lividans* 66 by conjugation from *E. coli*. Plasmids pAE4, pKSJ553, 554, 588, 595, and 596 were transformed into electrocompetent WM6029. Strains were grown in LB-Apr-DAP to OD_{600} of 0.5-0.6 and 1 mL harvested by centrifugation, washed and re-suspended in 500 µL LB. Spore stocks of *S. lividans* 66 (50 µL per conjugation, titer $1x10^9$ colony forming units (CFU) mL^{-1}) were thawed on ice, centrifuged, washed and re-suspended in 500 µL LB, and germinated at 50 ^oC for 10 min. Germinated spores and washed *E. coli* cells were combined, centrifuged at 6,000 rpm for 5 min. The cell pellet was re-suspended in 100 µL M9 media and spotted on MS1 plates in 5 µl aliquots. Plates were dried in a biosafety cabinet, incubated at 30°C for 16 h, and flooded with 500 µl of apramycin (2 mg ml^{-1} stock). After 5 days, three exconjugants from each mating were struck for isolation on MS-Apr and purified by three successive passages from fully sporulated cultures. Genomic DNA was isolated from each strain and used for diagnostic PCR reactions. One primer bound to an internal region of the kanamycin resistance cassette, and the second primer bound upstream or downstream region of the disrupted gene. (Table S4).

Production of phosphonate natural products from native, heterologous expression, and deletion strains

Strains were cultivated in 20x150 mm test tubes containing 5 mL of ATCC 172 medium on an orbital shaker (200 rpm) at 30 °C for 4-5 days. Cultures of *Streptomyces* sp. S-515 and B-2790 were inoculated onto ISP2, ISP4, GUBC, and R2AS agar (200 µL per plate). Cultures of *S. lividans* heterologous expression and deletion strains were inoculated onto GUBC and R2AS. After 10 days plates were frozen at -20 ^oC and thawed to recover 25 mL of liquid extracts from individual cultures (3 plates each). Extracts were then lyophilized. Dried samples were reconstituted in 1 mL dI H_2O and analyzed by mass spectrometry and NMR spectroscopy as described below.

NMR spectroscopy

NMR spectroscopy was performed at the OSU Campus Chemical Instrument Center. All NMR spectra were recorded at 25 ^oC on a Bruker Avance III HD Ascend 600 MHz spectrometer (600 MHz for ^1H, 150 MHz for ^{13}C and 243 MHz for ^{31}P) equipped with a Bruker 5 mm Smart Broadband

Observe solution probe (BBFO), a Bruker Avance Neo 400 MHz spectrometer (400 MHz for ^{1}H, 100 MHz for ^{13}C and 162 MHz for ^{31}P) equipped with a 5 mm Prodigy Cryoprobe, or a Bruker Avance III HD Ascend 700 MHz spectrometer (700 MHz for ^{1}H, 176 MHz for ^{13}C and 283 MHz for ^{31}P) equipped with a 5 mm Triple-resonance Observe (TXO) cryoprobe. Proton and carbon chemical shifts are reported in δ values relative to an external standard of 0.1% tetramethylsilane in D_2O. Phosphorus chemical shifts are reported in δ values relative to an external standard of 85% phosphoric acid. ^{1}H-^{31}P gHMBC (gradient Heteronuclear Multiple-Bond Correlation) spectra were collected after optimization of long-range proton-phosphorus coupling at 18 Hz. Spectra were processed in MestReNova 12 software.

Mass spectrometry

Weak-anion exchange was routinely used as a preparative step to enrich phosphonic acids from extracts and improve signal to noise with Chelex-100-Fe resin was previously described.[4]

Mass spectrometry analyses were performed on an Agilent 6540B Q-ToF system equipped with a 1260 Infinity II HPLC system as previously described.[4]

Samples were also analyzed on a Thermo Q-Exactive Orbitrap with a Vanquish-H UHPLC system. The data were acquired under high resolution mode (RP = 70,000) with an AGC target of 1E6 and a maximum IT of 200ms. For positive mode analyses, samples were diluted to a total volume of 100uL with 85% MeCN and 0.1% formic acid, 5uL of which was injected onto a Waters XBridge Amide (2.1 x 150 mm) HPLC column. The buffer used for UHPLC was H_2O with 0.1% formic acid (solvent A) and MeCN with 0.1% formic acid (solvent B). The flow rate was set at 0.35 mL/min. The elution gradient started at 85% solvent B for 2 minutes followed by a linear gradient to 40% solvent B over 4 min, a maintenance at 40% solvent B over 3 minutes, a return to 85% solvent B over 6 seconds, and re-equilibrated for 5.4 min before the next injection. For MS/MS, the same settings, gradient, and column were used. Target ion(s) were added to the inclusion list with a starting collision energy of 10eV for dipeptides and 15eV dipeptides, adjusted in 5eV increments in subsequent runs if needed.

Expression and Purification of His_6-SUMO-PnaA

The *pnaA* gene was amplified by PCR from the genomic DNA of S-515 using primers listed in Table S4. The product was gel purified and cloned into linearized 2-ST by Gibson assembly to yield pKSJ599, which encodes His_6-SUMO-PnaA. The plasmid was transformed into *E. coli* Rosetta (DE3) pLysSRARE. The strain was grown in 8 L LB-Amp-Clm at 37 ^{o}C, 220 rpm to OD_{600} 0.4 and cold shocked on ice for 10 min. Protein production was induced by the addition of IPTG to 0.2 mM and the culture returned to 16 °C, 220 rpm for 16 h. The culture was harvested by centrifugation, and the cell pellet was re-suspended in 60 mL lysis buffer (50 mM HEPES pH 7.5, 250 mM NaCl, 10 mM imidazole) containing 10 mg lysozyme and 100 U DNase. The suspension was gently mixed at room temperature for 20 min. Cells were lysed by sonication and centrifuged at 12,000 rpm for 30 min at 4 ^{o}C.

Clarified cell lysates was combined with 5 mL HisTrap FF column at 2.5 mL min^{-1} using an Akta Go FPLC system. His_6-SUMO-PnaA was purified using buffer A (50 mM HEPES pH 7.5, 250 mM NaCl, 10% glycerol), and buffer B (50 mM HEPES pH 7.5, 250 mM NaCl, 10% glycerol, 500 mM imidazole) by the following program: 2 column volumes (CV) 6% buffer B; 1 CV 6-10% buffer B; 2

CV 10% buffer B; 4 CV 10-50% buffer B; 2 CV 50% buffer B; 10 CV 0% buffer B. Fractions containing the target protein (analyzed by Bradford and SDS-PAGE) were concentrated to 2.5 mL by 10 kDa Amicon Ultra-15 Millipore centrifugal filter and then desalted by PD-10 column with storage buffer (50 mM HEPES pH 7.5, 250 mM NaCl, 10% glycerol). Yield: 1.84 mg L^{-1} of culture.

Expression and purification of His_6-PnaB

The *pnaB* gene was amplified by PCR from the genomic DNA of S-515 using primers listed in Table S4. The product was gel purified and cloned into linearized pET28B by Gibson assembly to yield pKSJ521, which encodes His_6-PnaB. The plasmid was transformed into *E. coli* Rosetta (DE3) pLysSRARE. The overproduction of His_6-PnaB was the same as His_6-SUMO-PnaA except 1 L LB-Km-Clm media was used. The culture was harvested by centrifugation, and the cell pellet was re-suspended in 20 mL lysis buffer (50 mM HEPES pH 7.5, 250 mM NaCl, 10 mM imidazole) containing 10 mg lysozyme and 100 U DNase. The suspension was gently mixed at room temperature for 20 min. Cells were lysed by sonication and centrifuged at 12,000 rpm for 30 min at 4 °C.

Clarified cell lysates was combined with 5 mL HisPur™ Ni-NTA affinity resin (Thermo scientific) in a column and gently nutated at 4 °C for 30 min. The resin was washed with 100 mL wash buffer (50 mM HEPES pH 7.5, 250 mM NaCl, 30 mM imidazole, 10% glycerol), and elute with 20 mL elution buffer A (50 mM HEPES pH 7.5, 250 mM NaCl, 50 mM imidazole, 10% glycerol), 20 mL elution buffer B (50 mM HEPES pH 7.5, 250 mM NaCl, 100 mM imidazole, 10% glycerol), and 20 mL elution buffer C (50 mM HEPES pH 7.5, 250 mM NaCl, 250 mM imidazole, 10% glycerol). Fractions containing the target protein (analyzed by Bradford and SDS-PAGE) were concentrated to 2.5 mL by 10 kDa Amicon Ultra-15 Millipore centrifugal filter and then desalted by PD-10 column with storage buffer (50 mM HEPES pH 7.5, 250 mM NaCl, 10% glycerol). Yield: 19 mg L^{-1} of culture.

Expression and purification of His_6-PnaC

The *pnaC* gene was amplified by PCR from the genomic DNA of S-515 using primers listed in Table S4. The product was gel purified and cloned into linearized pET28B by Gibson assembly to yield pKSJ522, which encodes His_6-PnaC. The plasmid was transformed into *E. coli* Rosetta (DE3) pLysSRARE. The overproduction and purification procedures for His_6-PnaB were the same as His_6-PnaC. Yield: 14 mg L^{-1} of culture.

Expression and purification of His_6-PnaD

The *pnaD* gene was amplified by PCR from the genomic DNA of S-515 using primers listed in Table S4. The product was gel purified and cloned into linearized pET28B by Gibson assembly to yield pKSJ409, which encodes His_6-PnaD. The plasmid was transformed into *E. coli* Rosetta (DE3) pLysSRARE. The overproduction and purification procedures for His_6-PnaB were the same as His_6-PnaC. Yield: 26.1 mg L^{-1} of culture.

Expression and purification of His_6-VlpB

Recombinant phosphonopyruvate reductase (VlpB) was purified from expressed and purified from *E. coli* as previously described.[11]

Expression and purification of His_6-MDH

The malate dehydrogenase gene (locus tag SCO4927) was amplified by PCR from the genomic DNA of *S. coelicolor* A3(2) using primers listed in Table S4. The product was gel purified and cloned into linearized pET28B by Gibson assembly to yield pKSJ441, which encodes His_6-MDH. The overproduction and purification procedures for His_6-MDH were the same as His_6-PnaC. Yield: 25 mg L^{-1} of culture.

Expression and purification of His_6-SerA

The *serA* gene was amplified by PCR from the genomic DNA of *E. coli* BL21 (DE3) using primers listed in Table S4. The product was gel purified and cloned into linearized pET28B by Gibson assembly to yield pKSJ635, which encodes His_6-MDH. The overproduction and purification procedures for His_6-MDH were the same as His_6-PnaC. Yield: 12 mg L^{-1} of culture.

Biochemical assays of His_6-SUMO-PnaA with His_6-PnaD

Typical reaction mixtures (300 µL) contained 20 µM His_6-PnaD, 20 µM His_6-SUMO-PnaA, 1.5 mM PEP, 3 mM L-Asp, 100 µM pyridoxal 5'-phosphate (PLP), and 2 mM $MgCl_2$ in 50 mM HEPES, 150 mM NaCl, pH 7.5. Reactions were incubated at 30 °C for 2 h, heat inactivated at 65 °C for 10 minutes, and then analyzed by ^{31}P NMR and LC-MS as described above.

Reactions in timecourse experiments (150 µL) contained 10 µM His_6-PnaD, 10 µM His_6-SUMO-PnaA, 1.5 mM PEP, 10 mM L-Asp, 100 µM PLP, and 2 mM $MgCl_2$ in 50 mM HEPES, 150 mM NaCl, pH 7.5. Samples were where heat inactivated 1, 5, 10, 30, 60, 120, and 240 min after initiation and then analyzed by ^{31}P NMR as described above.

Reactions examining the effect of amino donor concentrations were the same as above, except they contained 1.5, 3, 5, or 10 mM of (L-Asp). Samples were heat inactivated after 120 min and then analyzed by ^{31}P NMR as described above.

Biochemical assays of His_6-SUMO-PnaA

Typical reaction mixtures (300 µL) contained 20 µM His_6-SUMO-PnaA, 1.5 mM L-PnAla, 3 mM oxaloacetate (OAA), and 100 µM PLP in 50 mM HEPES, 150 mM NaCl, pH 7.5. Reactions were incubated at 30 °C for 2 h, heat inactivated at 65 °C for 10 minutes, and then analyzed by NMR and LC-MS as described above.

Reactions in time course experiments (150 µL) contained 10 µM His_6-SUMO-PnaA, 1.5 mM L-PnAla, 10 mM OAA, and 100 µM PLP in 50 mM HEPES, 150 mM NaCl, pH7.5. Samples were where heat inactivated 1, 5, 10, 30, 60, 120, and 240 min after initiation and then analyzed by ^{31}P NMR as described above.

Reactions examining the effect of keto-acid acceptor concentrations were the same as above, except they contained 1.5, 3, 5, or 10 mM of (OAA). Samples were heat inactivated after 120 min and then analyzed by ^{31}P NMR as described above.

Kinetic analyses

All reactions (400 µL) were performed in 50 mM HEPES, 150 mM NaCl, pH 7.5, 30 °C using an Agilent Cary 300 spectrophotometer. The instrument was first blanked using the reaction

containing the enzymes and the fixed substrate (without NADH). Initial rates were calculated from continual changes in absorbance (340 nm) before and after substrate addition. All replicates (n = 3) for each set kinetic measurements were performed in the same sitting using substrates and protein drawn from the same stock. Single reactions yielding data points were collected one full curve at a time (starting with reactions containing the lowest concentration of substrate). Replicate assays were then immediately performed (again, sequentially one full curve at a time). Data were analyzed using SigmaPlot 15.

Conversion of PnPy and L-Asp to L-PnAla and OAA by His_6-SUMO-PnaA

Each reaction contained 0.5 μM PnaA, 5 μM MDH, 200 μM NADH, 5 mM L-Asp, and were initiated by the addition of 0.0025, 0.00375, 0.005, 0.0065, 0.008, 0.01, or 0.015 mM PnPy. PnaA was pre-incubated with PLP (1:5) on ice for 10 min prior to use.

Conversion of L-PnAla and OAA to PnPy and L-Asp by His_6-SUMO-PnaA

Each reaction contained 1 μM PnaA, 5 μM VlpB, 200 μM NADH, 5 mM OAA, the 340 nm absorbance change was recorded for 1 min to measure the background assumption of OAA by VlpB. Then the reactions were initiated by the addition of 0.1, 0.2, 0.35, 0.5, 0.65, 0.8, or 1 mM PnAla. PnaA was pre-incubated with PLP (1:5) on ice for 10 min prior to use.

Conversion of PEP and L-Asp to L-PnAla and OAA by His_6-SUMO-PnaA and His_6-PnaD

Each reaction contained 1 μM PnaA, 5 μM PnaD, 5 μM MDH, 200 μM NADH, 2 mM MgCl2, 5 mM L-Asp, and were initiated by the addition of 0.1, 0.2, 0.35, 0.5, 0.65, 0.8, or 1 mM PEP. PnaA was pre-incubated with PLP (1:5) on ice for 10 min prior to use.

Conversion of L-Glu and OAA to αKG and L-Asp by His_6-SUMO-PnaA

Each reaction contained 1 μM PnaA, 5 μM SerA, 200 μM NADH, 5 mM OAA, the 340 nm absorbance change was recorded for 1 min to measure the background assumption of OAA by SerA, and were initiated by the addition of 0.5, 1, 1.5, 2, 3, 4, 6, 10, or 15 mM L-Glu. PnaA was pre-incubated with PLP (1:5) on ice for 10 min prior to use.

Conversion of αKG and L-Asp to L-Glu and OAA by His_6-SUMO-PnaA

Each reaction contained 1 μM PnaA, 5 μM MDH, 200 μM NADH, 5 mM L-Asp, and were initiated by the addition of 0.1, 0.2, 0.35, 0.5, 0.65, 0.80, 1, or 1.25 mM αKG. PnaA was pre-incubated with PLP (1:5) on ice for 10 min prior to use.

Conversion of αKG to 2-hydroxyglutaric acid (HGA) by His_6-SerA

Each reaction contained 1 μM SerA, 200 μM NADH, and were initiated by the addition of 0.02, 0.035, 0.05, 0.075, 0.1, 0.15, 0.2, 0.35 mM αKG.

Conversion of OAA to malate by His_6-SerA

Each reaction contained 1 μM SerA, 200 μM NADH, and were initiated by the addition of 0.05, 0.1, 0.2, 0.375, 0.5, 0.65, 0.8 or 1 mM OAA.

Biochemical assays of His_6-PnaB

Typical reaction mixtures (500 µL) contained 10 µM His_6-PnaB, 3 mM ATP, 1 mM L-PnAla, 3 mM chemically synthesized L-Ala-L-Val, L-Val-L-Val, L-Ala-L-Ile, or L-Thr-L-Val in 50 mM Tris-HCl, 100 mM NaCl, 2 mM $MgCl_2$ pH 9. Reactions were incubated at 30 °C for 2 h, heat inactivated at 65 °C for 10 minutes, and then analyzed by NMR and LC-MS as described above.

The carboxylate specificity of His_6-PnaB was assessed by setting 500 µL reactions containing 10 µM His_6-PnaB, 3 mM ATP, 3 mM L-Ala-L-Val, and 1 mM of Asp, Glu, or L-2-amino-4-phosphonobutyrate (AP4) in 50 mM Tris-HCl, 100 mM NaCl, 2 mM $MgCl_2$ pH 9. Reactions were incubated at 30 °C for 16 h, heat inactivated at 65 °C for 10 minutes, and then analyzed by LC-MS as described above.

Biochemical assays of His_6-PnaC

Typical reaction mixtures (100 µL) contained 10 µM His_6-PnaC, 3 mM ATP, 3 mM of each L-amino acid, and 5 mM ATP in 50 mM Tris-HCl, 100 mM NaCl, 2 mM $MgCl_2$ pH 9. Reactions were incubated at 30 °C for 16 h, heat inactivated at 65 °C for 10 minutes, and then analyzed by LC-MS as described above.

Tripeptide synthesis assays using PnaB and PnaC

Reaction mixtures (200 µL) contained 10 µM His_6-PnaB, 10 µM His_6-PnaC, 1 mM L-PnAla, 3 mM L-amino acid A, 3 mM L-amino acid B, and 5 mM ATP in 50 mM Tris-HCl, 100 mM NaCl, 2 mM $MgCl_2$ pH 9. Reactions were incubated at 30 °C for 16 h, heat inactivated at 65 °C for 10 minutes, and then analyzed by NMR and LC-MS as described above.

Analysis of PnaC and PnaB substrate specificity reactions

An extracted ion chromatogram (EIC) was obtained for each potential product *m/z*. The shape and retention time of each EIC were used to evaluate the quality and plausibility of true signals, and samples with low-confidence EICs were re-analyzed individually, to address the challenges of ion suppression (Figure S150) and background noise (Figure S151).

LC-HRMS/MS fragmentation analysis was performed to verify and determine the structure of each product. Published analyses of amino acid fragmentation were referenced in structural assignment of fragment ions.[12] Fragment assignment was complicated by limitations in parent ion selection, as the Orbitrap instrument used has a minimum isolation window of 0.4 *m/z*. To determine which fragments resulted from the target parent ion, EICs were compared between each fragment and parent ion (Figure S152).

Peptide fragmentation data was organized according to conserved diagnostic fragments. For dipeptides, this was the y_1 ion, which indicated which amino acid was the *C*-terminal residue. For tripeptides, these were the a_2 and b_2 fragments, which indicated which residue formed an amide bond with PnAla.

SUPPLEMENTAL FIGURES

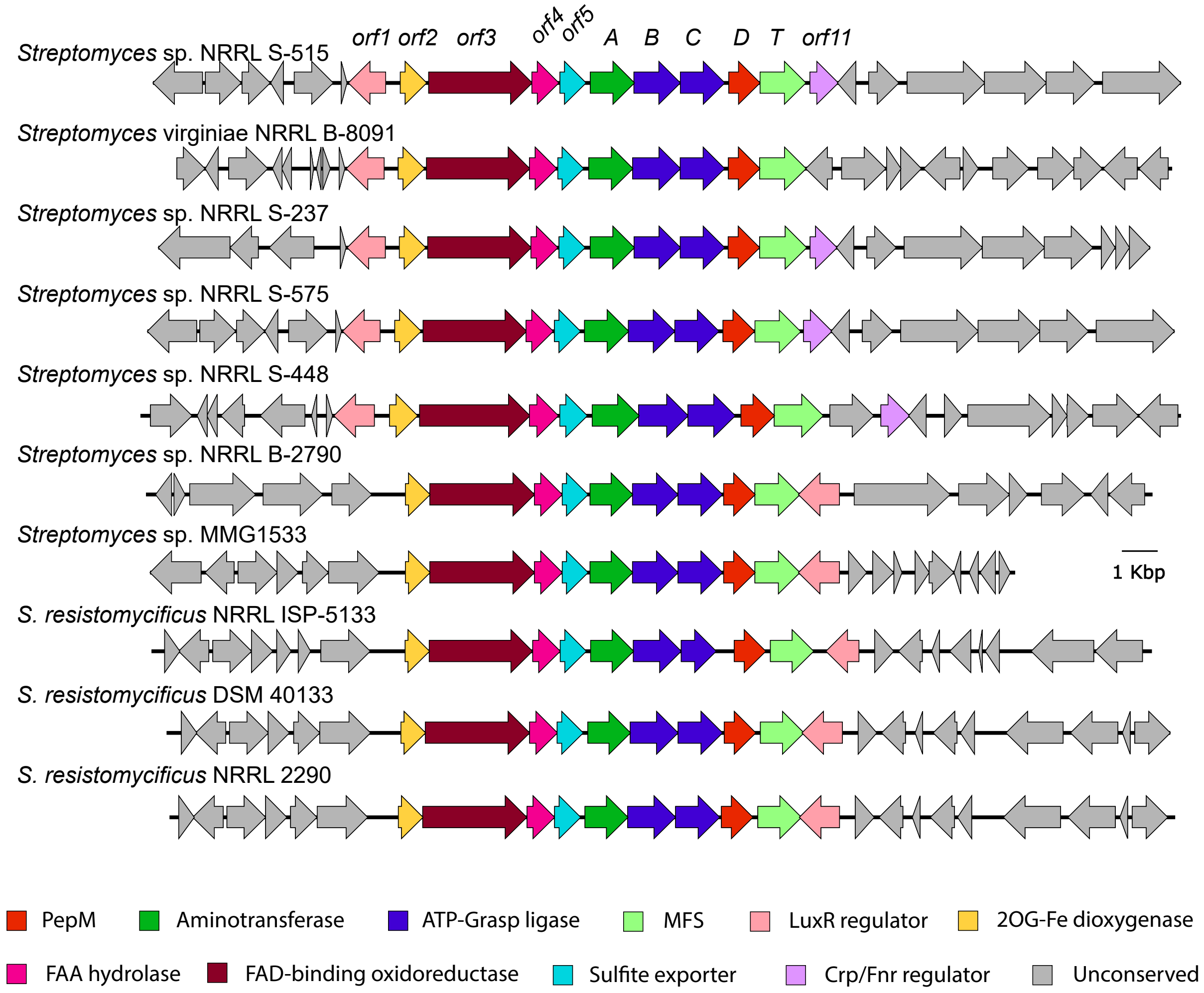

Figure S1. The *pepM* gene neighborhoods of strains containing the phosphonoalamide BGC Detailed annotation can be found in Table S1.

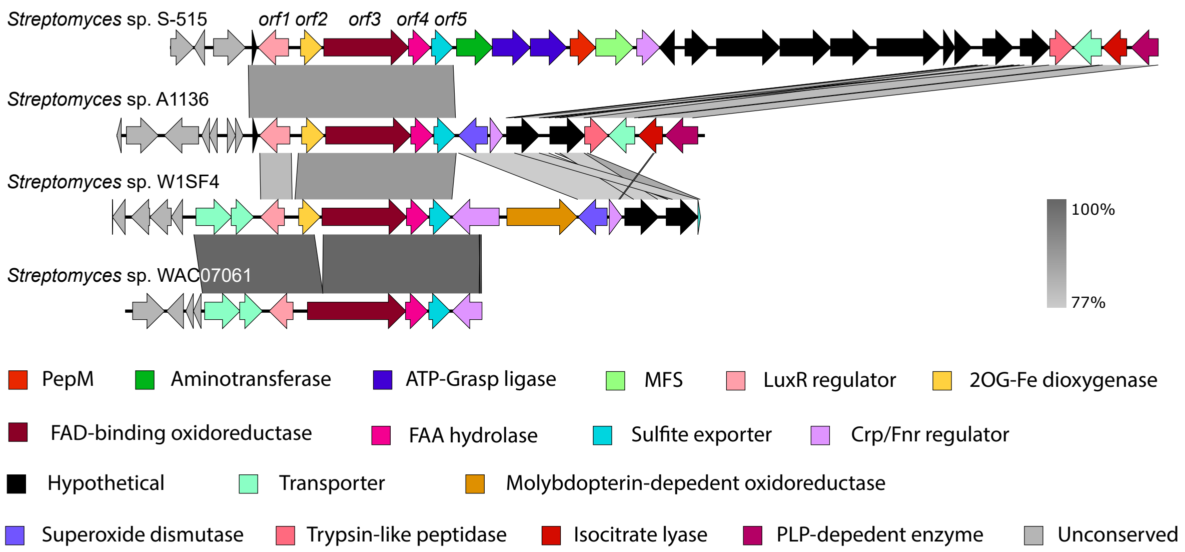

Figure S2. Synteny analysis between the phosphonoalamide gene neighborhood of S-515 and the genomes of strains lacking *pepM*

Detailed annotation of *orf1-5* can be found in Table S1.

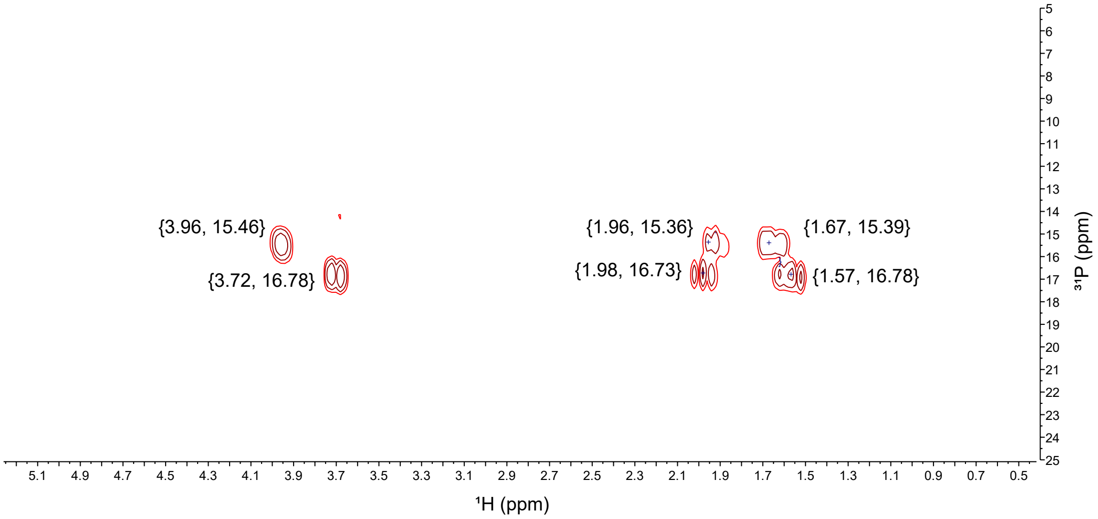

Figure S3. ^{1}H-^{31}P NMR spectra of *S. lividans* 66 *attB*::pKSJ588 extract

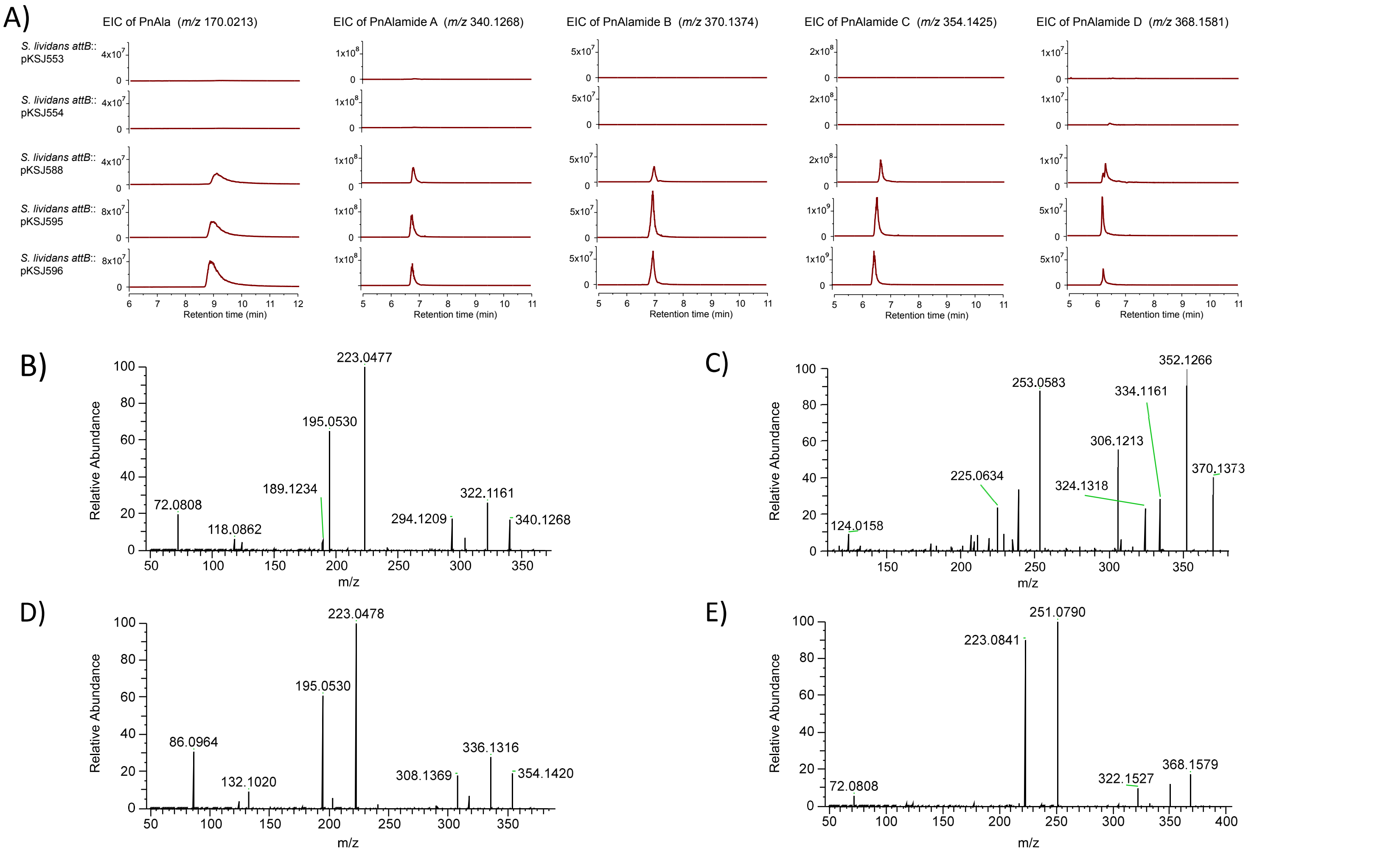

Figure S4. LC-HRMS analysis of phosphonates produced by heterologous expression strains.

A) EICs for PnAla and PnAlamides A-D in heterologous expression strain extracts. LC-HRMS/MS fragmentation was carried out to verify the identity of all phosphonoalamides within each sample, with representative spectra shown for B) PnAlamide A, C) PnAlamide B, D) PnAlamide C, and E) PnAlamide D. All labeled *m/z* are within 5 ppm of theoretical for fragments shown in Tables S51 (PnAlamide A, PnAla-Ala-Val, and PnAlamide C, PnAla-Ala-Ile), S80 (PnAlamide B, PnAla-Thr-Val), and S58 (PnAlamide D, PnAla-Val-Val).

1. His_6-PnaD (PepM): 33.8 kDa
2. His_6-SUMO-PnaA: 56.0 kDa
3. His_6-PnaB: 47.8 kDa
4. His_6-PnaC: 44.9 kDa

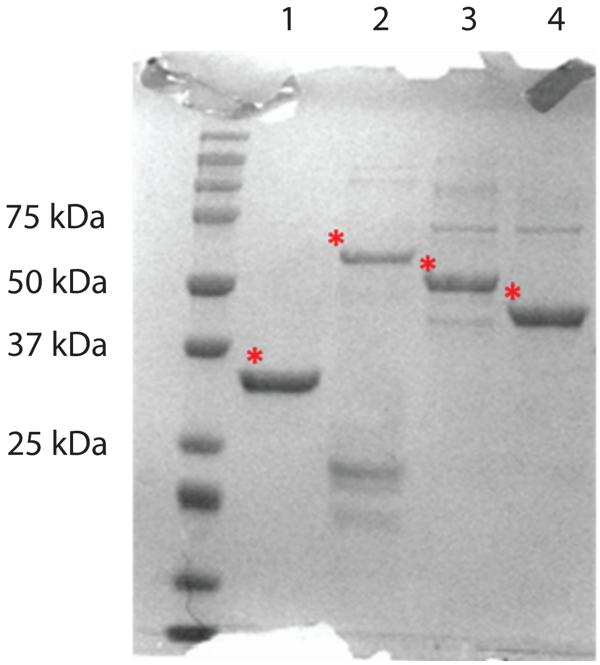

Figure S5. SDS-PAGE of purified Pna proteins
10 µg of PnaD, PnaB, and PnaC and 3 µg of PnaA were loaded, with asterisks indicating bands of expected size

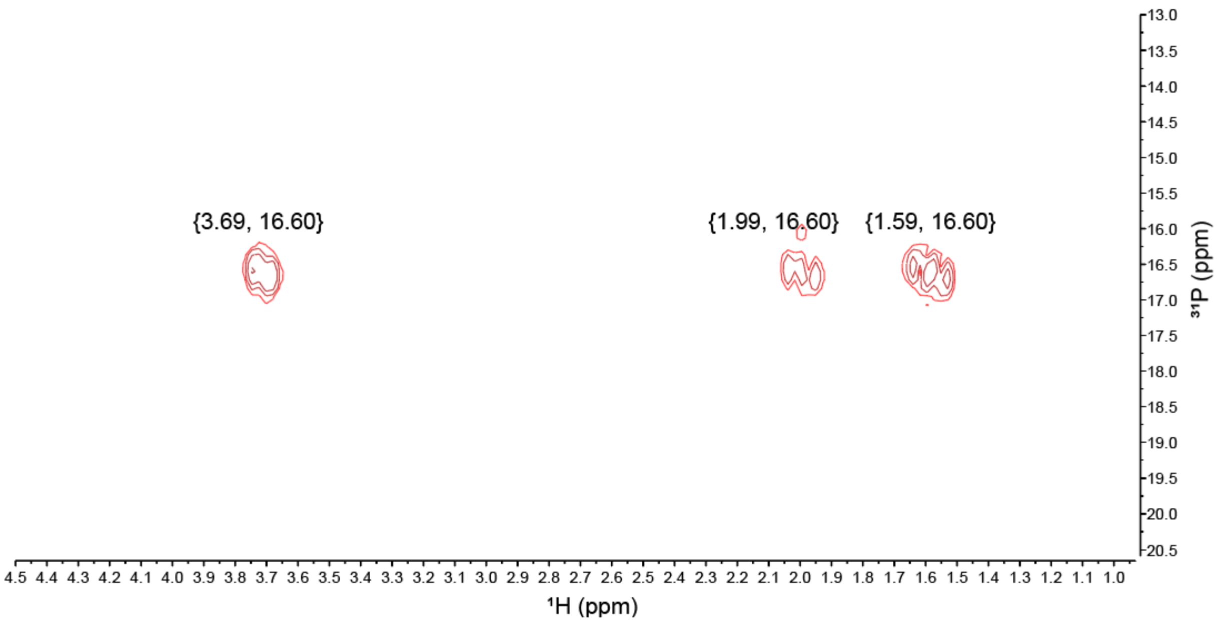

Figure S6. ^{1}H-^{31}P HMBC of the PnaD-PnaA coupled reaction

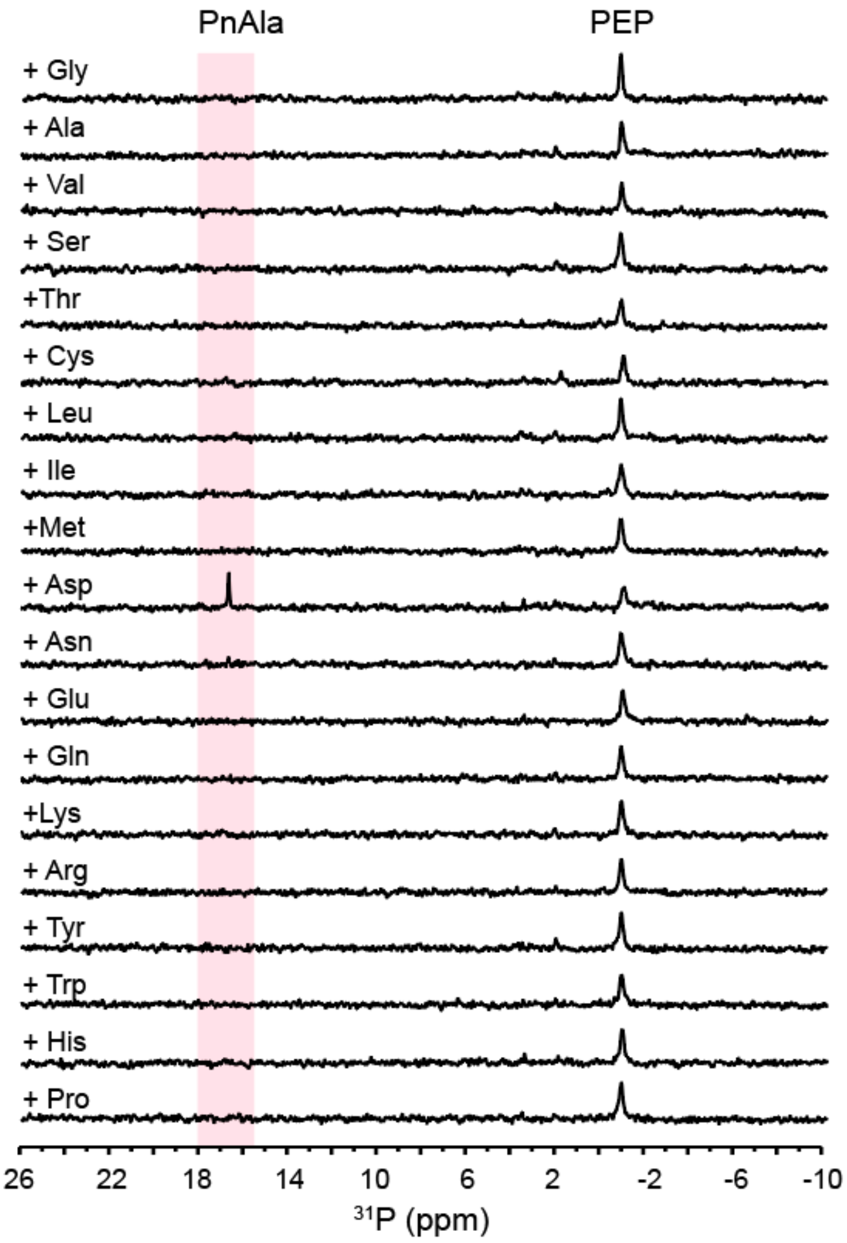

Figure S7. ^{31}P NMR spectra of PnaD-PnaA reactions with different amino donors.

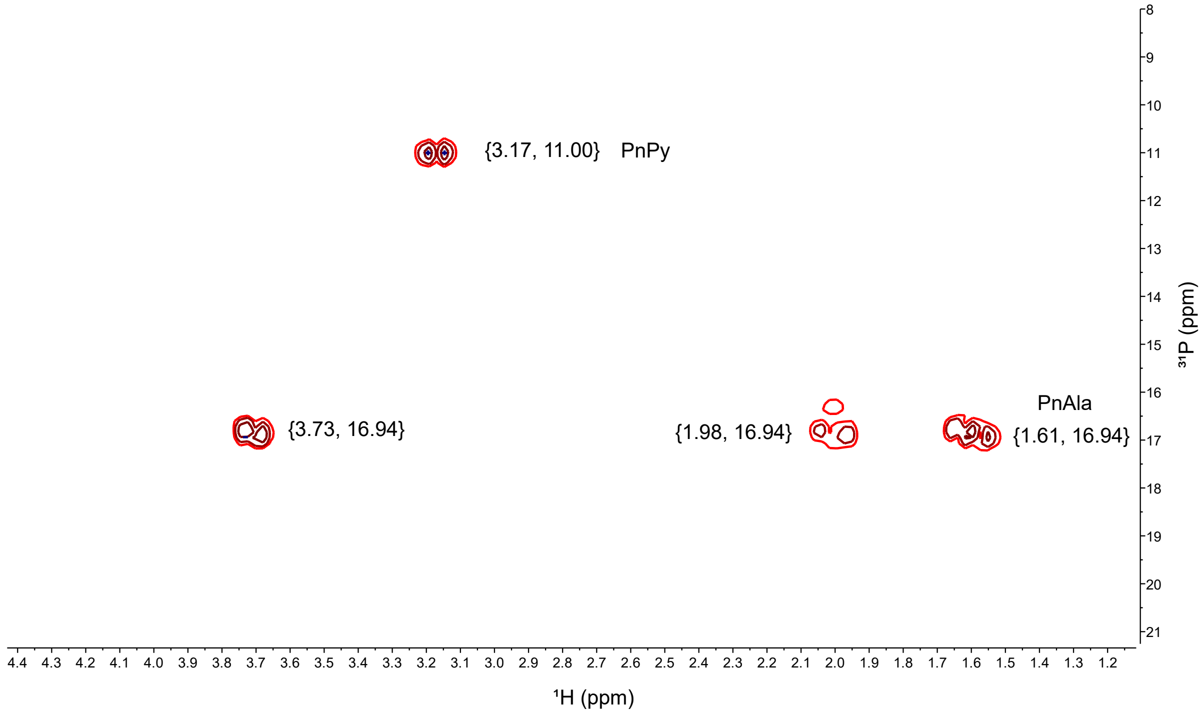

Figure S8. ^{1}H-^{31}P HMBC spectra of PnaA catalyzed transamination of OAA to Asp and PnAla to PnPy.

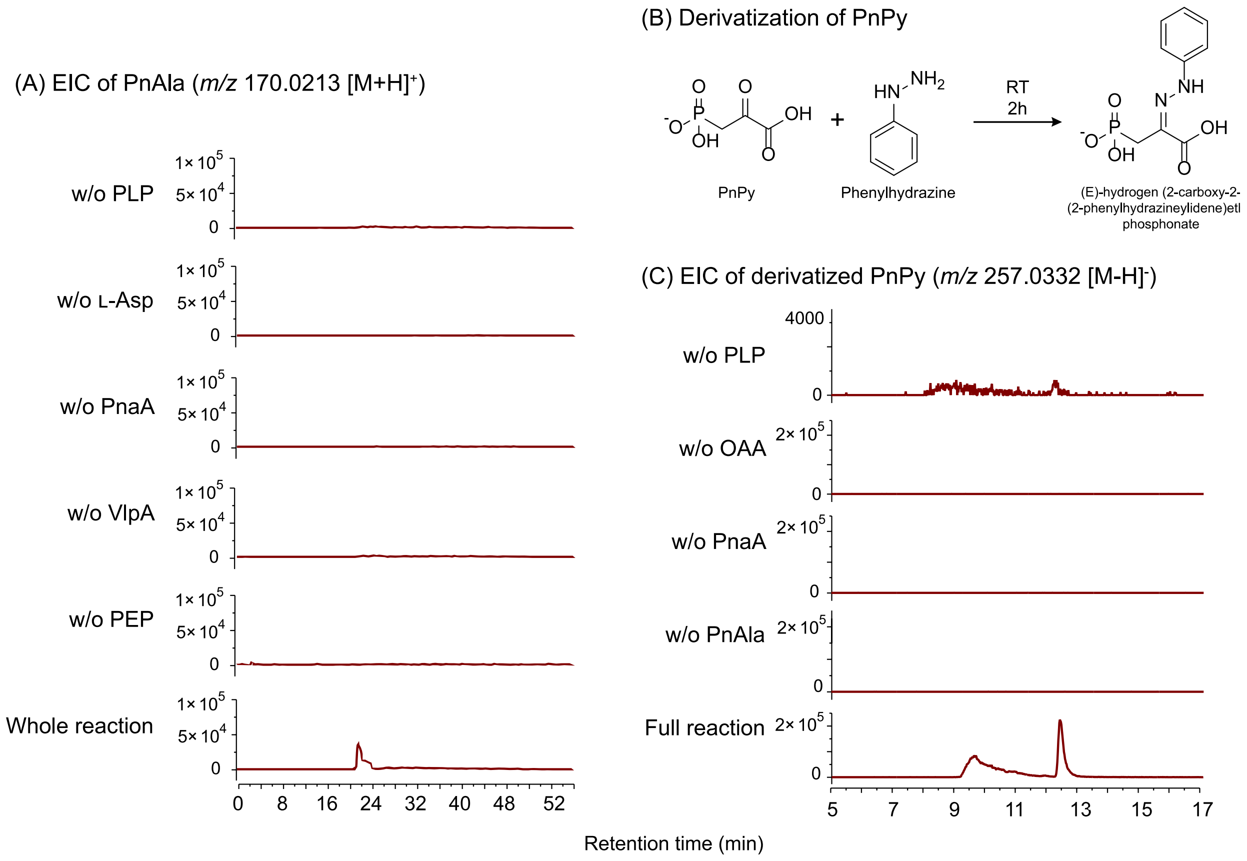

Figure S9. LC-HRMS analysis of PnaA catalyzed transamination reactions.

A) EIC of PnAla from PnaA-PnaD reactions

B) Reaction scheme of PnPy derivatization with phenylhydrazine

C) EIC of phenylhydrazine-derivatized PnPy formed from PnaA reactions

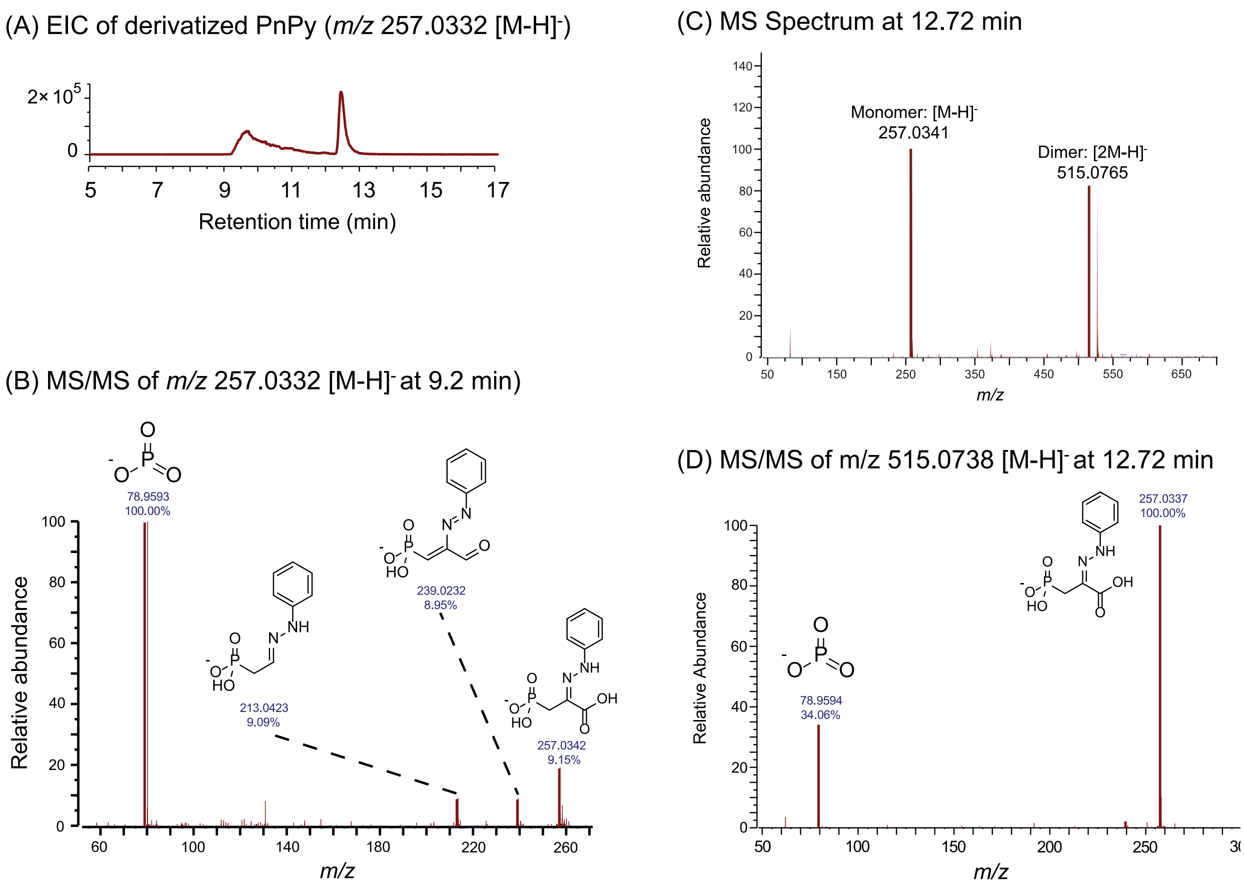

Figure S10. LC-HRMS analysis of derivatized PnaA catalyzed transamination reactions

A) The phenylhydrazine derivatization reaction shows two EIC peaks corresponding to derivatized PnPy (*m/z* 257.0332), one with a retention time of 9.2 min and another with a retention time of 12.72 min. B) MS/MS fragmentation of *m/z* 257.0332 at 9.2 min verified this to be derivatized PnPy. C) The mass spectrum at 12.72 min shows two major species, corresponding to the [M-H]- and [2M-H]- ions of derivatized PnPy. D) MS/MS fragmentation of *m/z* 515.0738 at 12.72 min verified this species to be a dimer of derivatized PnPy, indicating that the *m/z* 257.0322 peak at 12.72 min stems from ionization source fragmentation of the dimer.

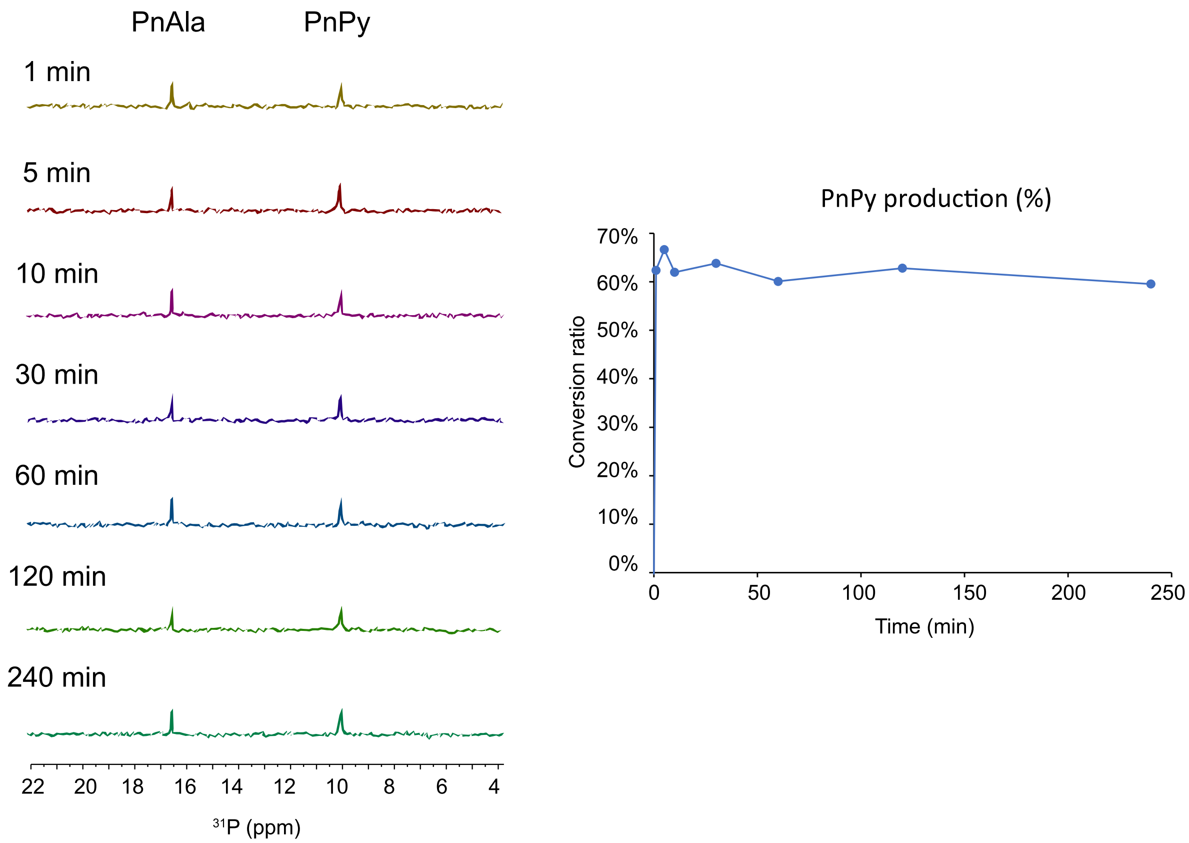

Figure S11. Timecourse of PnPy formation in the PnaA-catalyzed transamination of PnAla to PnPy using OAA as the keto-acid acceptor

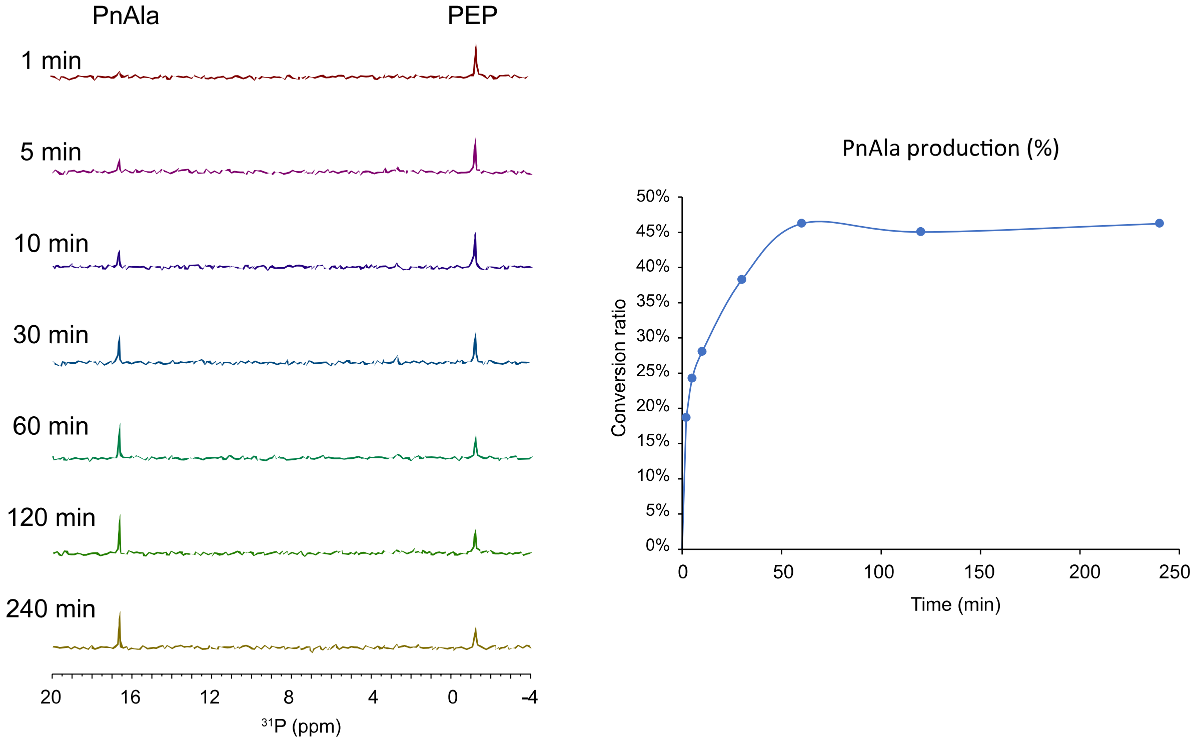

Figure S12. Timecourse of PnAla formation in the PnaA-catalyzed transamination of Asp to OAA using PnPy as the keto-acid acceptor

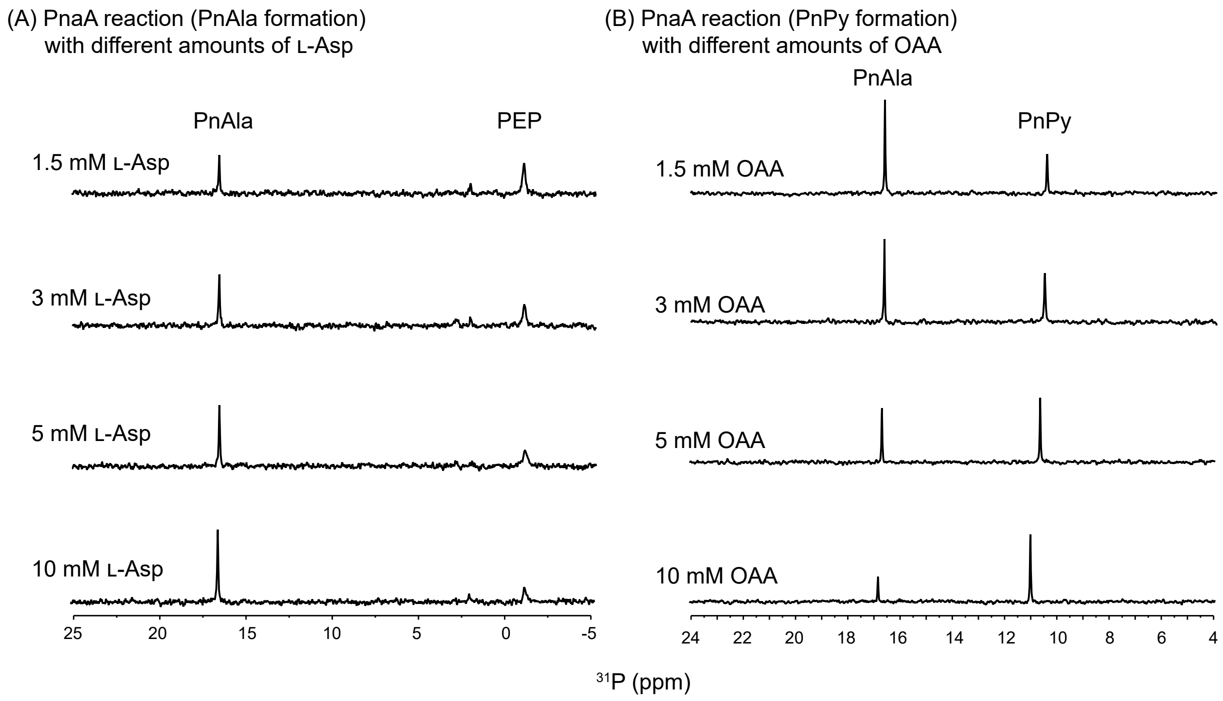

Figure S13. Transamination reactions with varied amounts of Asp or OAA after 2 h
A) The PnaA forward reaction containing 1.5-10 mM Asp
B) The PnaA reverse reaction containing 1.5-10 mM OAA

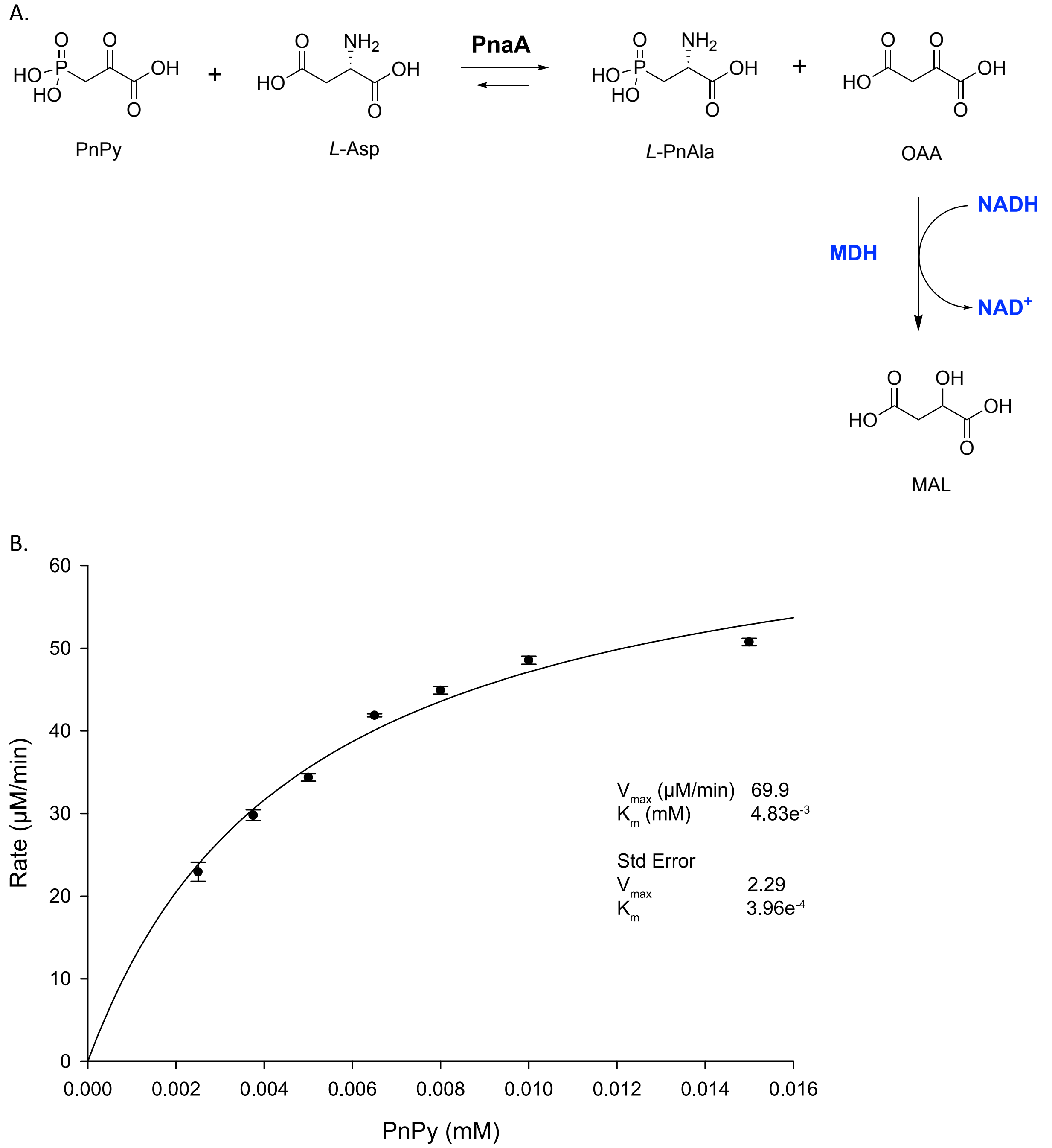

Figure S14. Kinetic analyses for the conversion of PnPy and L-Asp to L-PnAla and OAA by PnaA.
A) Apparent steady-state parameters were determined by coupling the formation of OAA to its reduction to malate (MAL) and the concurrent oxidation of NADH by malate dehydrogenase (MDH)
B) The Michaelis-Menten plot fit to the resulting measurements. Each datapoint is the average from three independent experimental replicates (n=3), with error bars indicating standard error.

A.

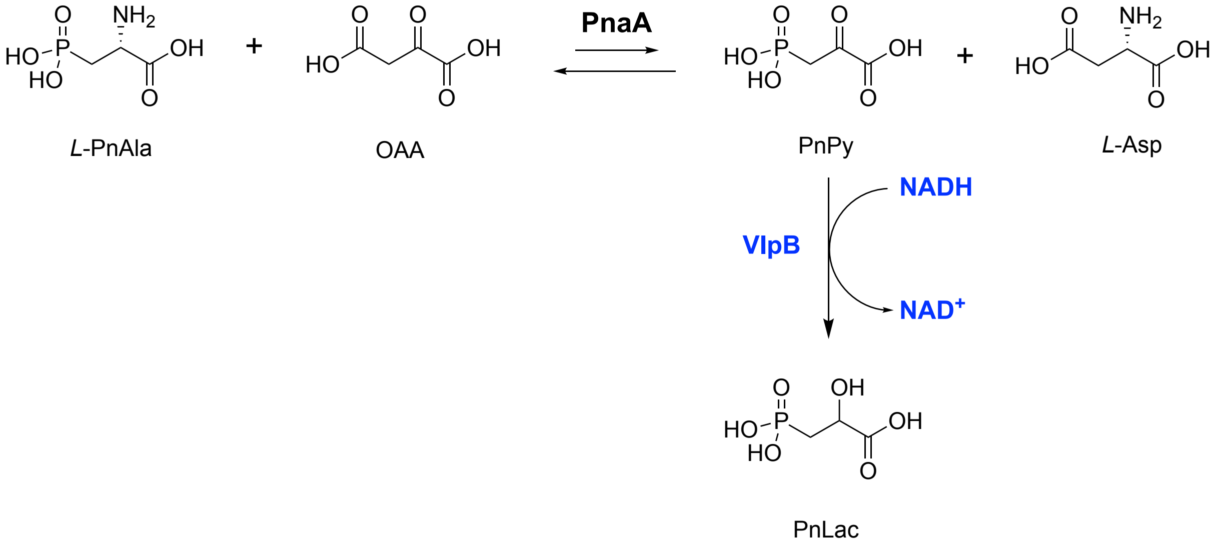

B.

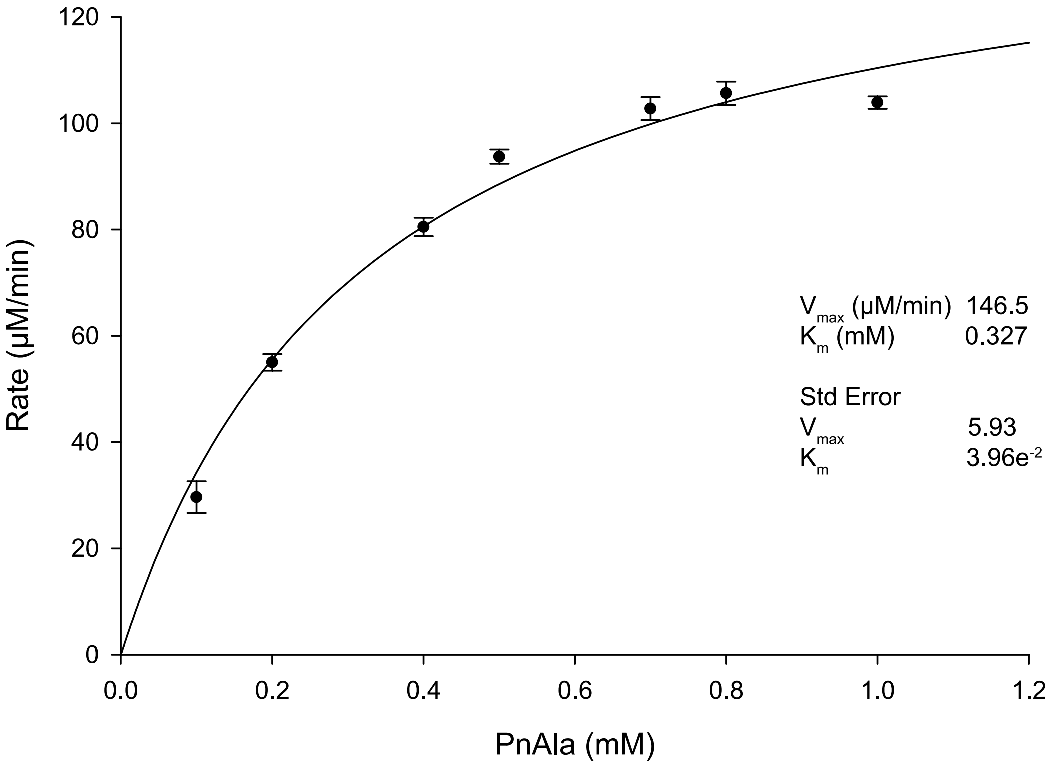

Figure S15. Kinetic analyses for the conversion of L-PnAla and OAA to PnPy and L-Asp by PnaA. A) Apparent steady-state parameters were determined by coupling the formation of PnPy to its reduction to PnLac and the concurrent oxidation of NADH by PnPy reductase (VlpB)

B) The Michaelis-Menten plot fit to the resulting measurements. Each datapoint is the average from three independent experimental replicates (n=3), with error bars indicating standard error.

A.

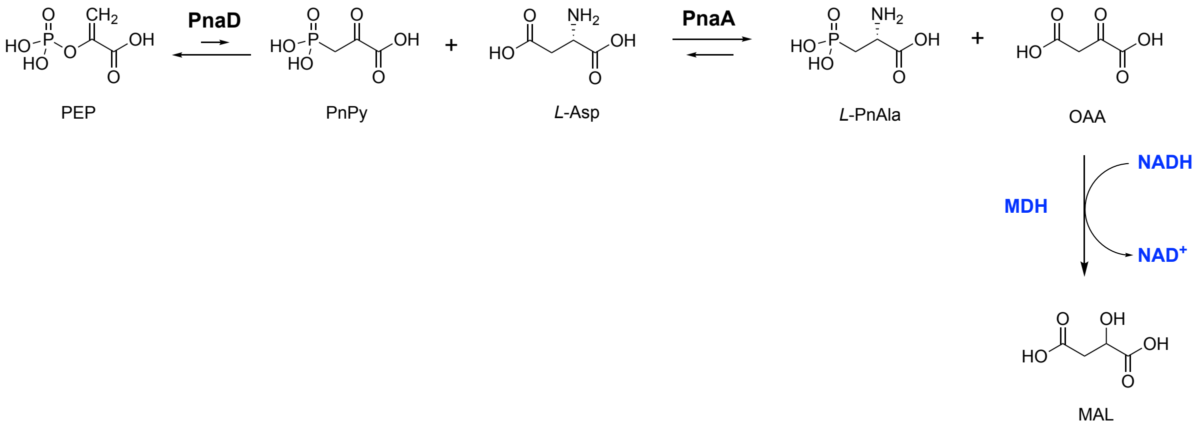

B.

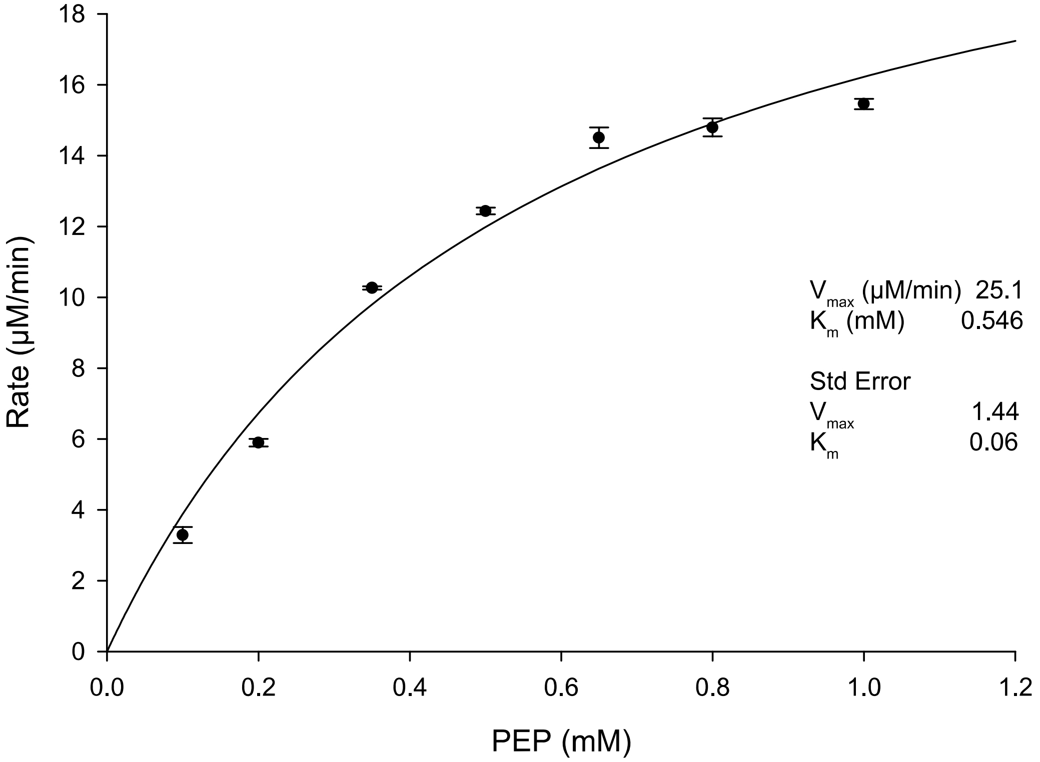

Figure S16. Kinetic analyses for the PEP conversion to L-PnAla in a coupled reaction with PnaD and PnaA.

A) Apparent steady-state parameters were determined by coupling the formation of OAA to its reduction and the concurrent oxidation of NADH by malate dehydrogenase (MDH)

B) The Michaelis-Menten plot fit to the resulting measurements. Each datapoint is the average from three independent experimental replicates (n=3), with error bars indicating standard error.

A.

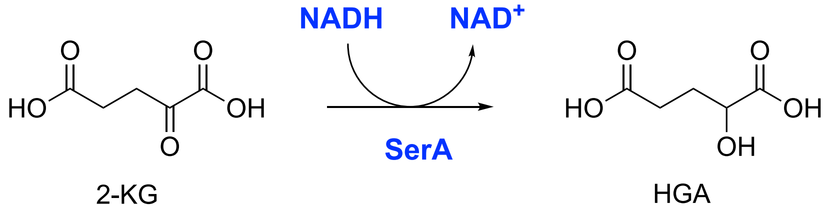

B.

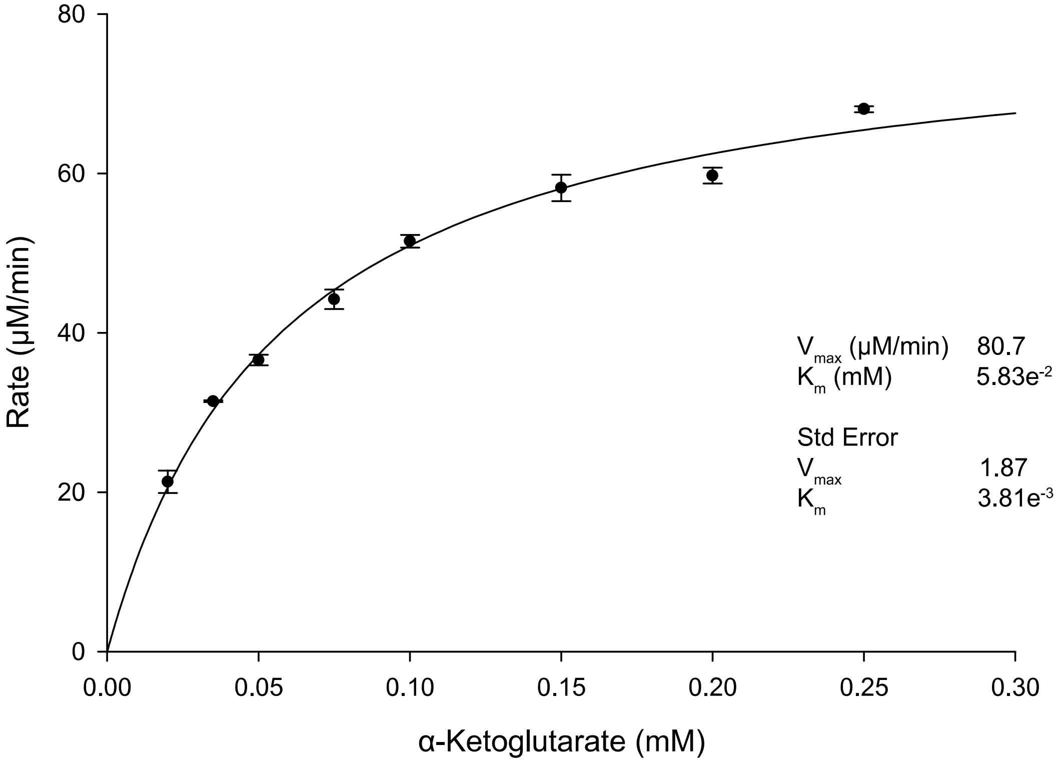

Figure S17. Kinetic analyses of αKG conversion to 2-hydroxyglutarate by 3-phosphoglycerate dehydrogenase SerA

A) Apparent steady-state parameters were determined from the oxidation of NADH by SerA.

B) The Michaelis-Menten plot fit to the resulting measurements. Each datapoint is the average from three independent experimental replicates (n=3), with error bars indicating standard error.

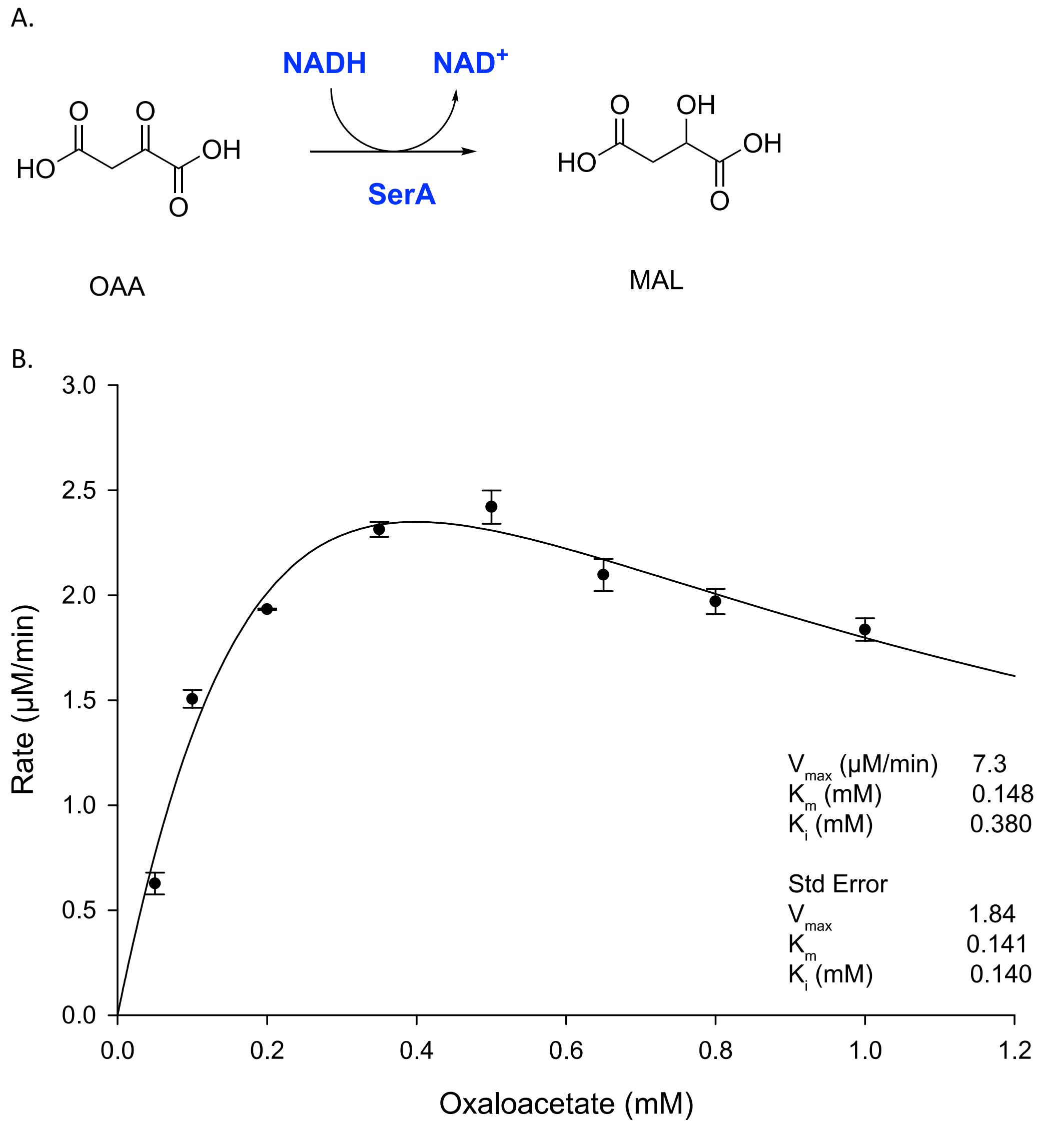

Figure S18. Kinetic analyses for the conversion of OAA to malate by 3-phosphoglycerate dehydrogenase SerA

A) Apparent steady-state parameters were determined from the oxidation of NADH by SerA.

B) The Michaelis-Menten plot fit to the resulting measurements. Each datapoint is the average from three independent experimental replicates (n=3), with error bars indicating standard error.

A.

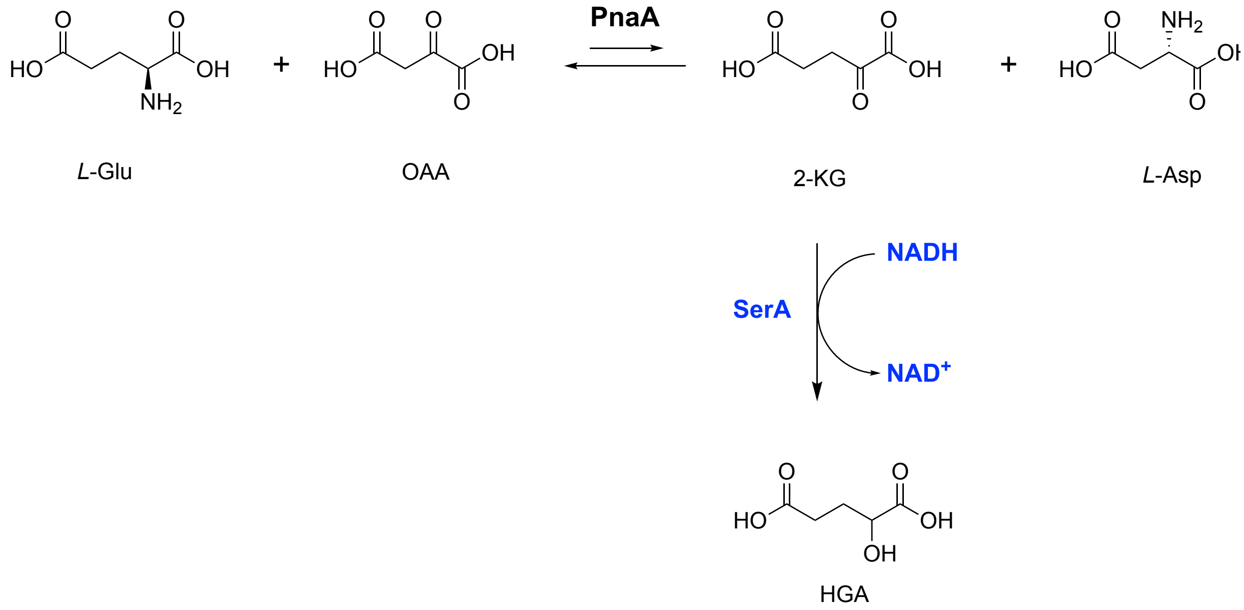

B.

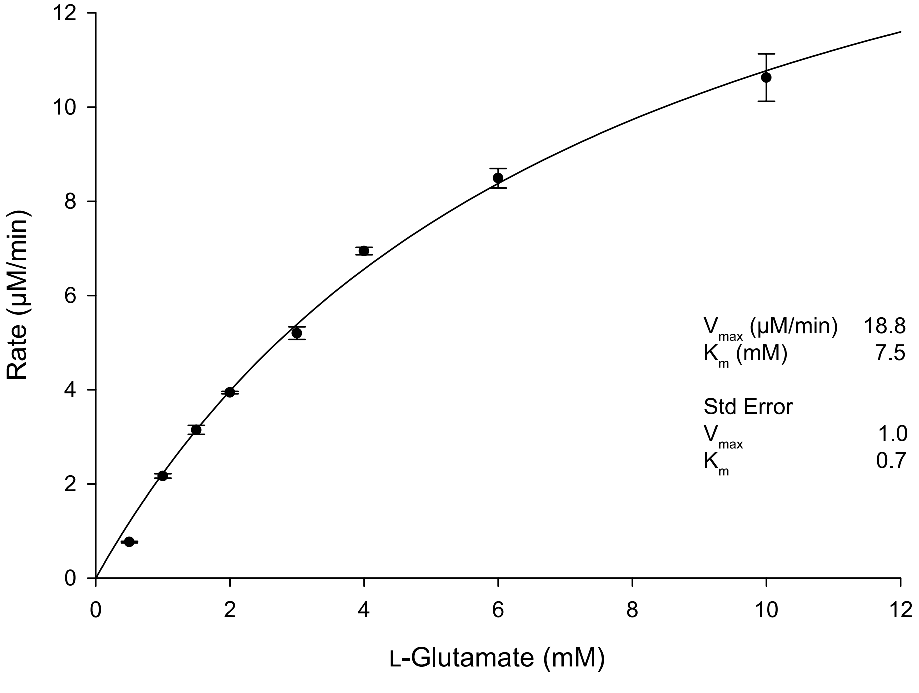

Figure S19. Kinetic analyses for the conversion of L-Glu and OAA to αKG and L-Asp by PnaA

A) Apparent steady-state parameters were determined by coupling the formation of αKG to its reduction to 2-hydroxyglutarate and the concurrent oxidation of NADH by SerA.

B) The Michaelis-Menten plot fit to the resulting measurements. Each datapoint is the average from three independent experimental replicates (n=3), with error bars indicating standard error.

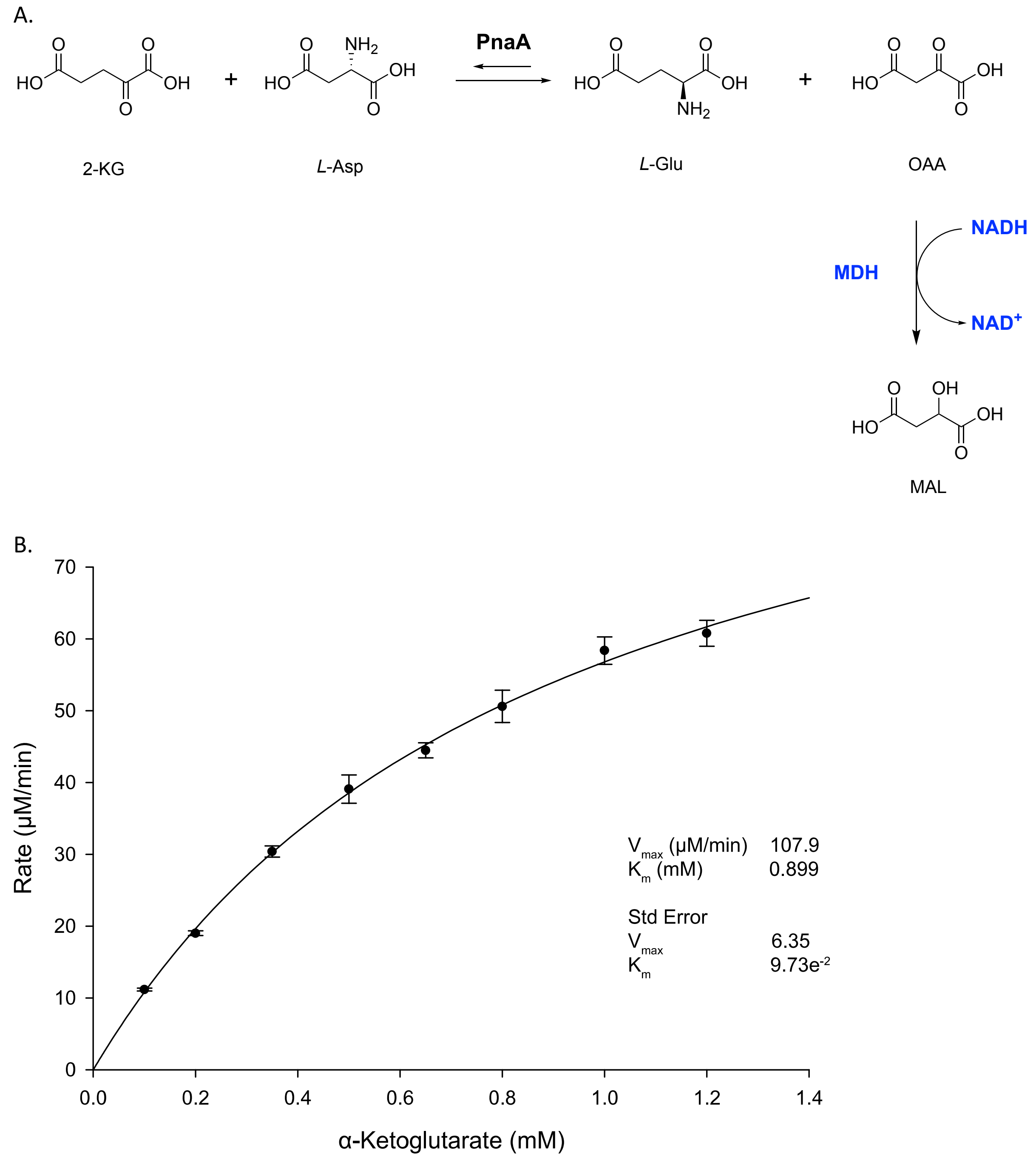

Figure S20. Kinetic analyses for the conversion of αKG and L-Asp to L-Glu and OAA to by PnaA
A) Apparent steady-state parameters were determined by coupling the formation of OAA to its reduction to malate and the concurrent oxidation of NADH by malate dehydrogenase.
B) The Michaelis-Menten plot fit to the resulting measurements. Each datapoint is the average from three independent experimental replicates (n=3), with error bars indicating standard error.

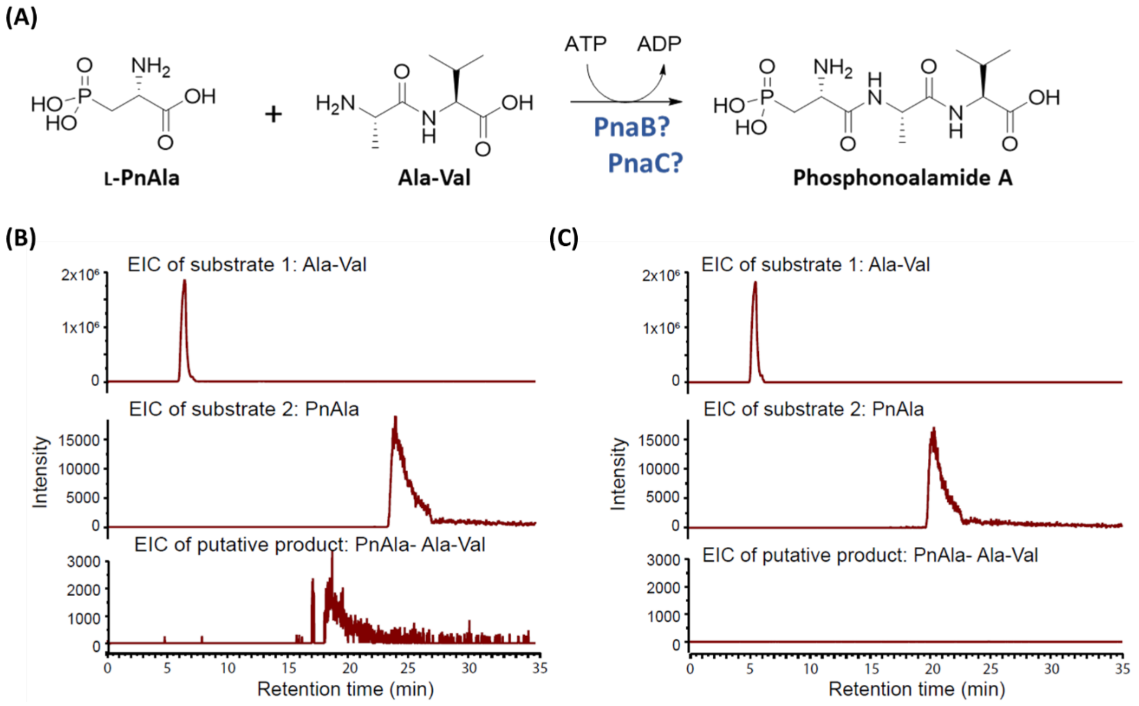

Figure S21. LC-MS analyses of PnaB and PnaC reactions for dipeptide ligation

A) Reaction scheme for the ligation of PnAla and Ala-Val

B) LC-MS analysis of the reaction containing PnaB, ATP, L-PnAla, and filtered PnaC reaction (PnaC, L-Ala, L-Val, ATP).

C) LC-MS analysis of the reaction containing PnaC, ATP, L-PnAla, and filtered PnaC reaction (PnaC, L-Ala, L-Val, ATP).

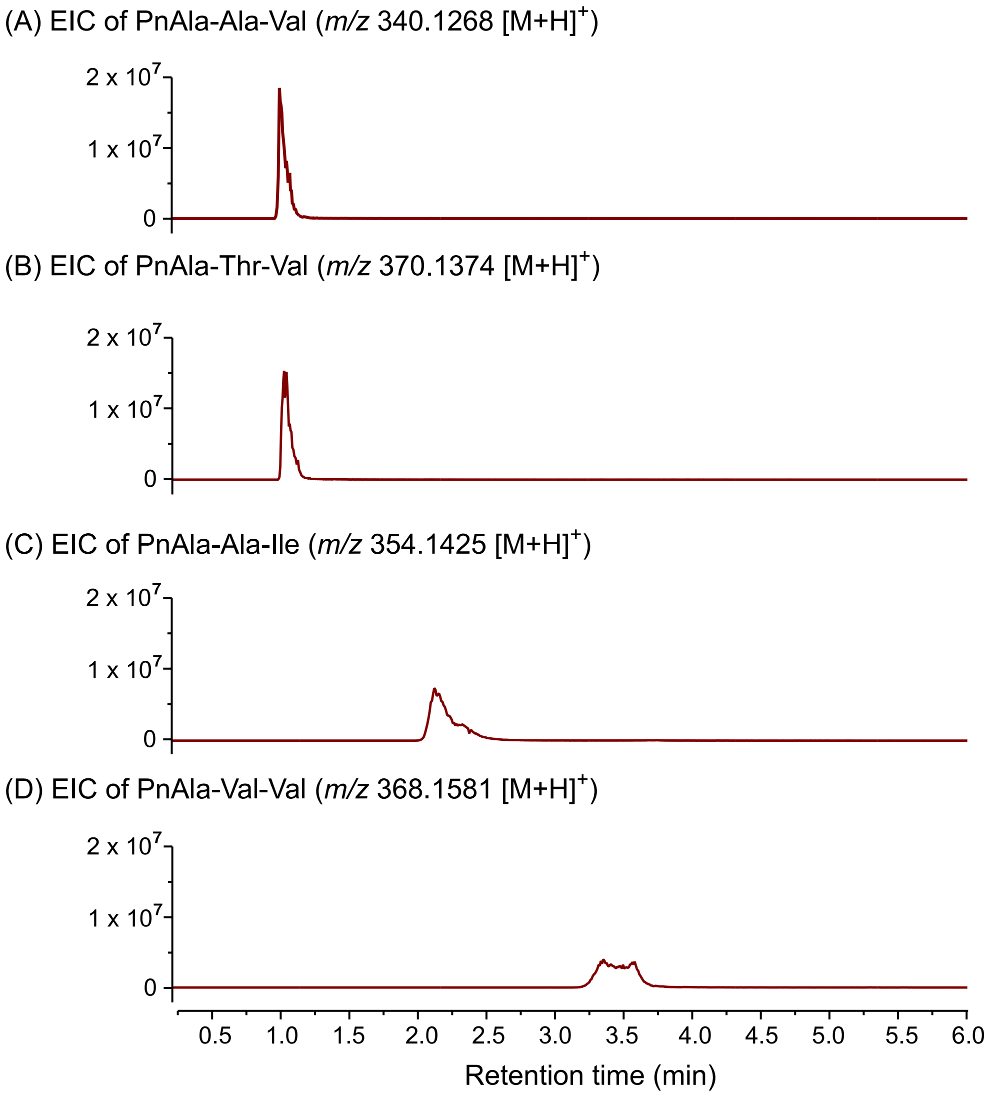

Figure S22. LC-MS analyses of PnaB ligation reactions with chemically synthesized dipeptides
Using Ala-Val (A), Thr-Val (B), Ala-Ile (C), and Val-Val (D)

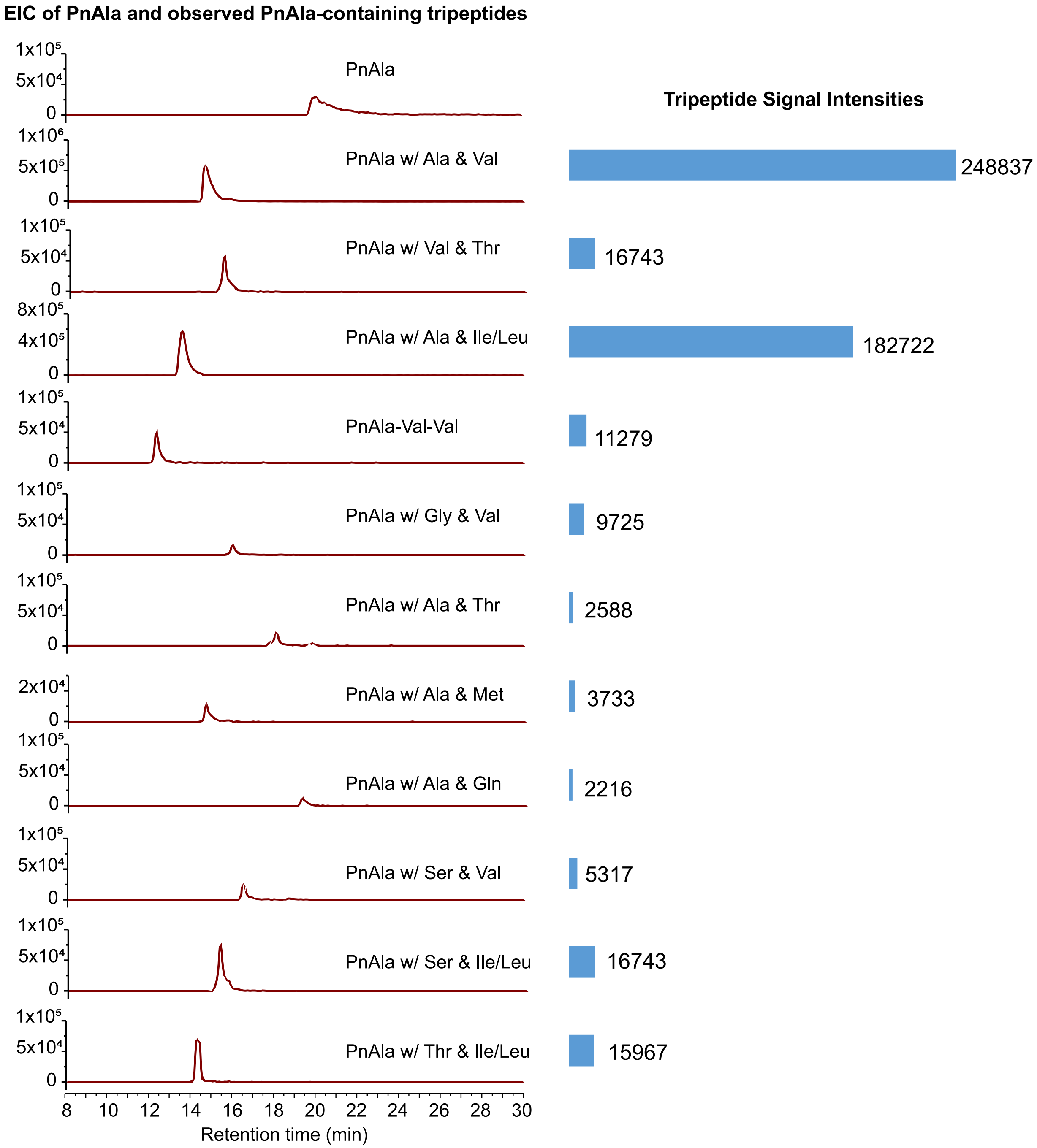

Figure S23. LC-HRMS detection of PnAla and phosphonoalamides within culture extracts of *S. lividans* 66 *attB*::pKSJ595

As Ile and Leu are isomers, it was not possible to distinguish whether a specific tripeptide ion contained Ile or Leu from LC-HRMS alone, nor was it possible to determine the arrangement of residues within the tripeptides.

A) EIC of Ala-Ala B) EIC of Gly-Ala C) EIC of Pro-Ala

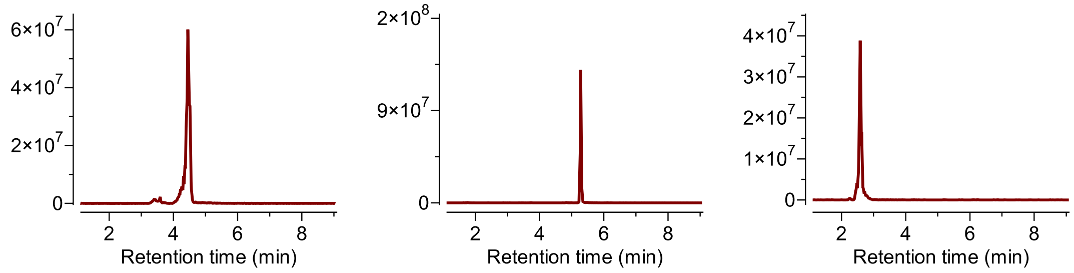

Figure S24. LC-HRMS detection of X-Ala dipeptides synthesized in PnaC reactions
A) Ala-Ala 161.0921 *m/z*, B) Gly-Ala 147.0764 *m/z*, C) Pro-Ala 187.1077 *m/z*

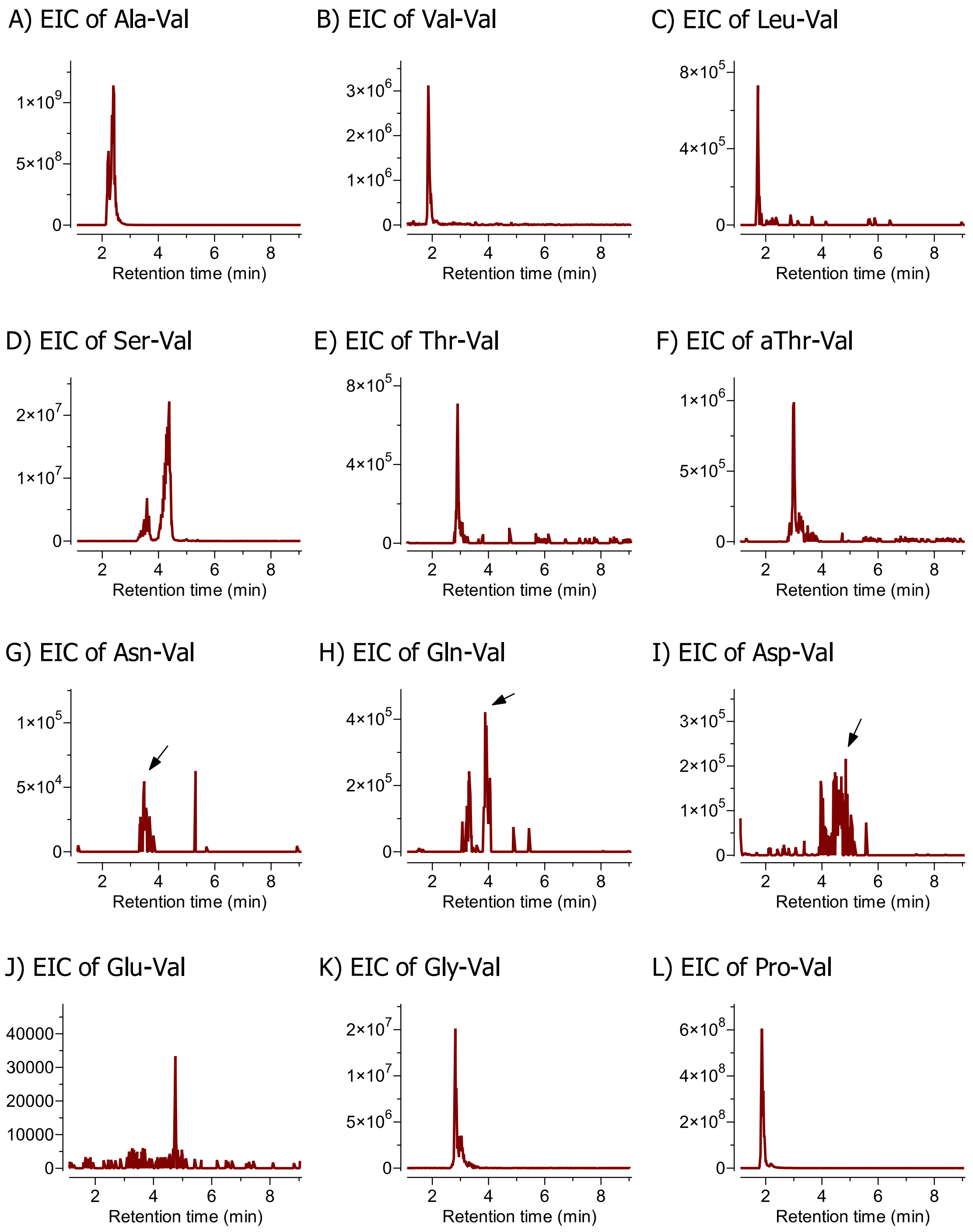

Figure S25. LC-HRMS detection of X-Val dipeptides synthesized in PnaC reactions

A) Ala-Val 189.1234 *m/z*, B) Val-Val 217.1547 *m/z*, C) Leu-Val 231.1703 *m/z*, D) Ser-Val 205.1183 *m/z*, E) Thr-Val 219.1339 *m/z*, F) aThr-Val 219.1339 *m/z*, G) Asn-Val 232.1292 *m/z*, H) Gln-Val 247.1448 *m/z*, I) Asp-Val 233.1132 *m/z*, J) Glu-Val 247.1289 *m/z*, K) Gly-Val 175.1077 *m/z*, L) Pro-Val 215.1390 *m/z*

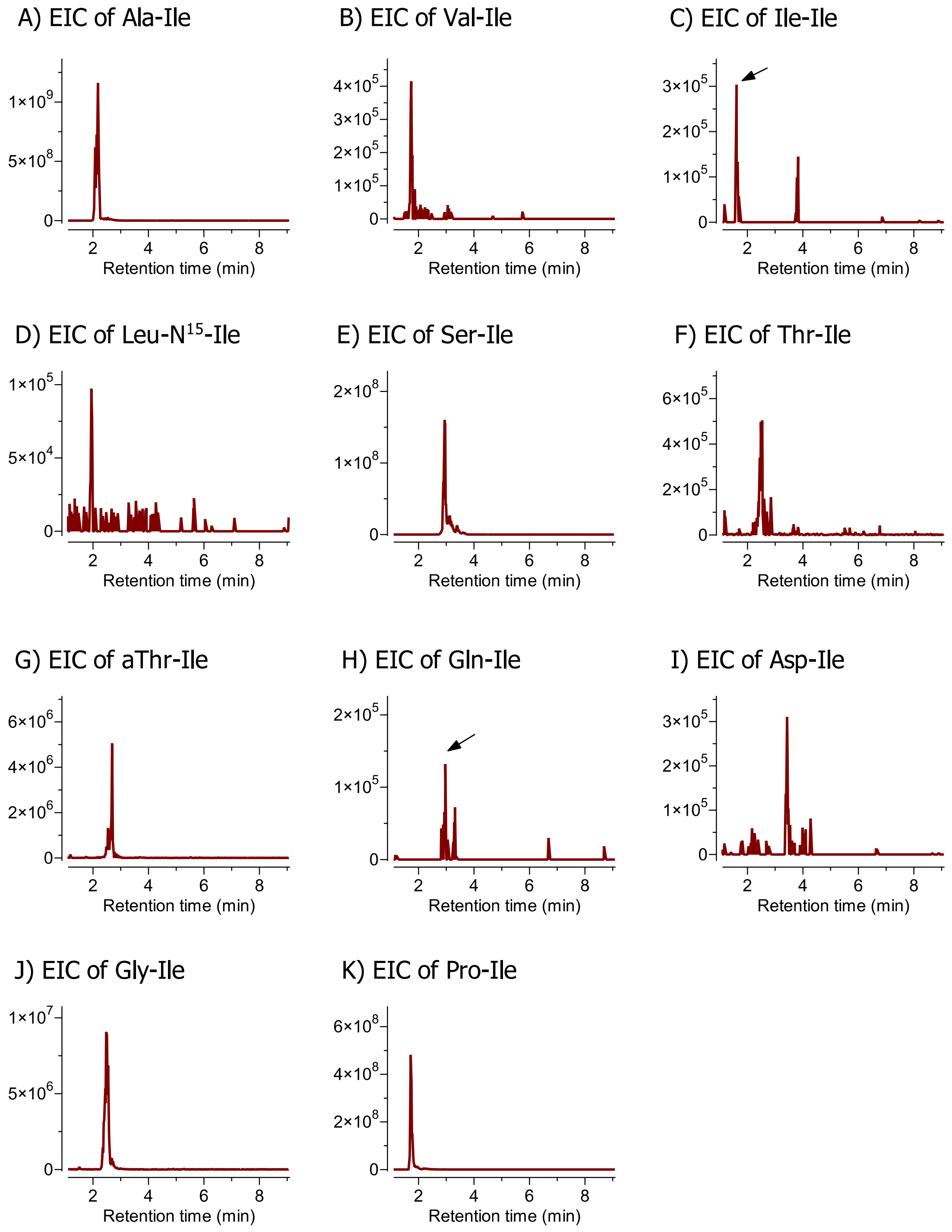

Figure S26. LC-HRMS detection of X-Ile dipeptides synthesized in PnaC reactions

A) Ala-Ile 203.1390 *m/z*, B) Val-Ile 231.1703 *m/z*, C) Ile-Ile 245.1860 *m/z*, D) Leu-^{15}N-Ile 246.1830 *m/z*, E) Ser-Ile 219.339 *m/z*, F) Thr-Ile 233.1496 *m/z*, G) aThr-Ile 233.1496 *m/z*, H) Gln-Ile 260.1605 *m/z*, I) Asp-Ile 247.1289 *m/z*, J) Gly-Ile 189.1234 *m/z*, K) Pro-Ile 229.1587 *m/z*

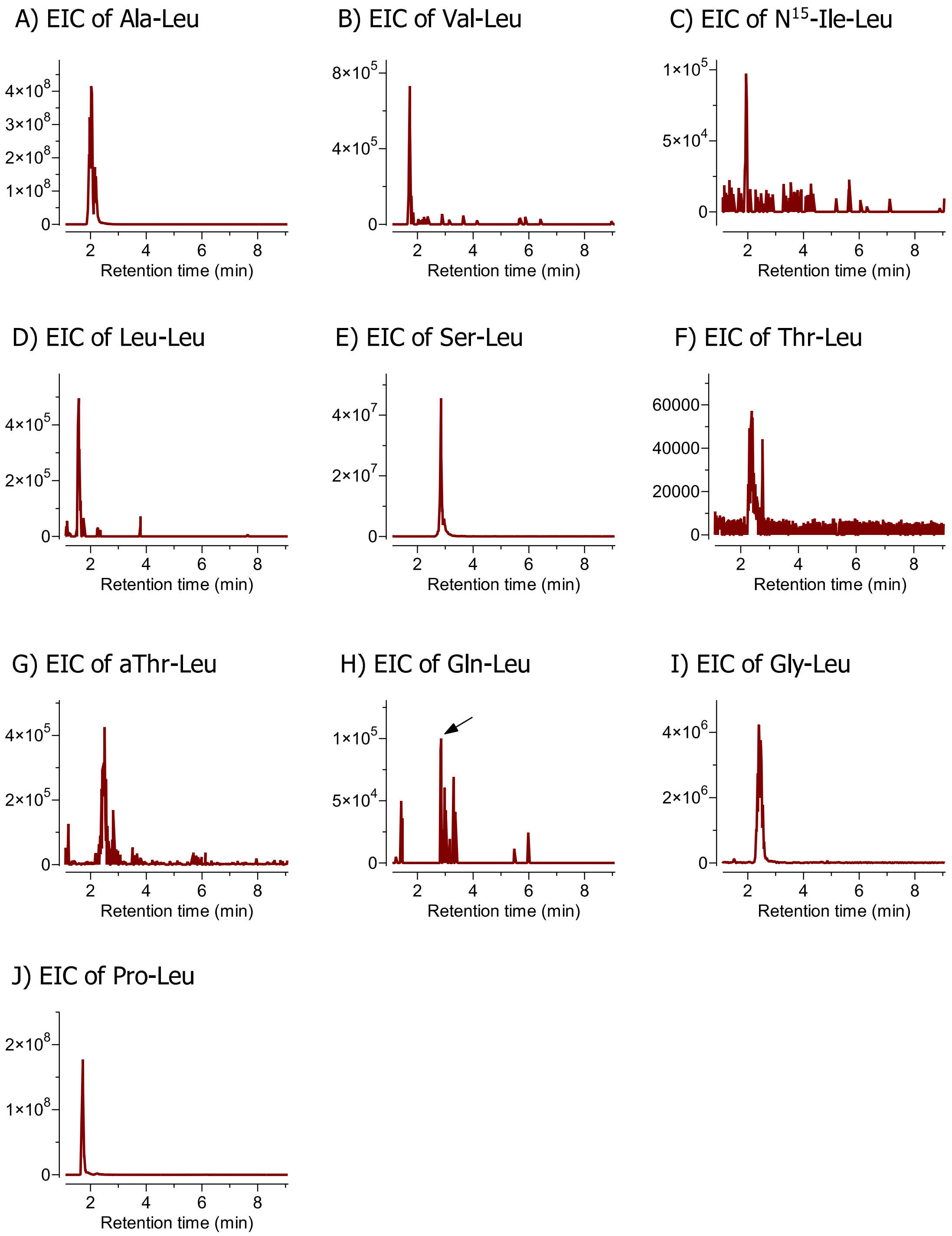

Figure S27. LC-HRMS detection of X-Leu dipeptides synthesized in PnaC reactions

A) Ala-Leu 203.1390 *m/z*, B) Val-Leu 231.1703 *m/z*, C) ^{15}N-Ile-Leu 246.1830 *m/z*, D) Leu-Leu 245.1860 *m/z*, E) Ser-Leu 219.1339 *m/z*, F) Thr-Leu 233.1496 *m/z*, G) aThr-Leu 233.1496 *m/z*, H) Gln-Leu 260.1605 *m/z*, I) Gly-Leu 189.1234 *m/z*, J) Pro-Leu 229.1547 *m/z*

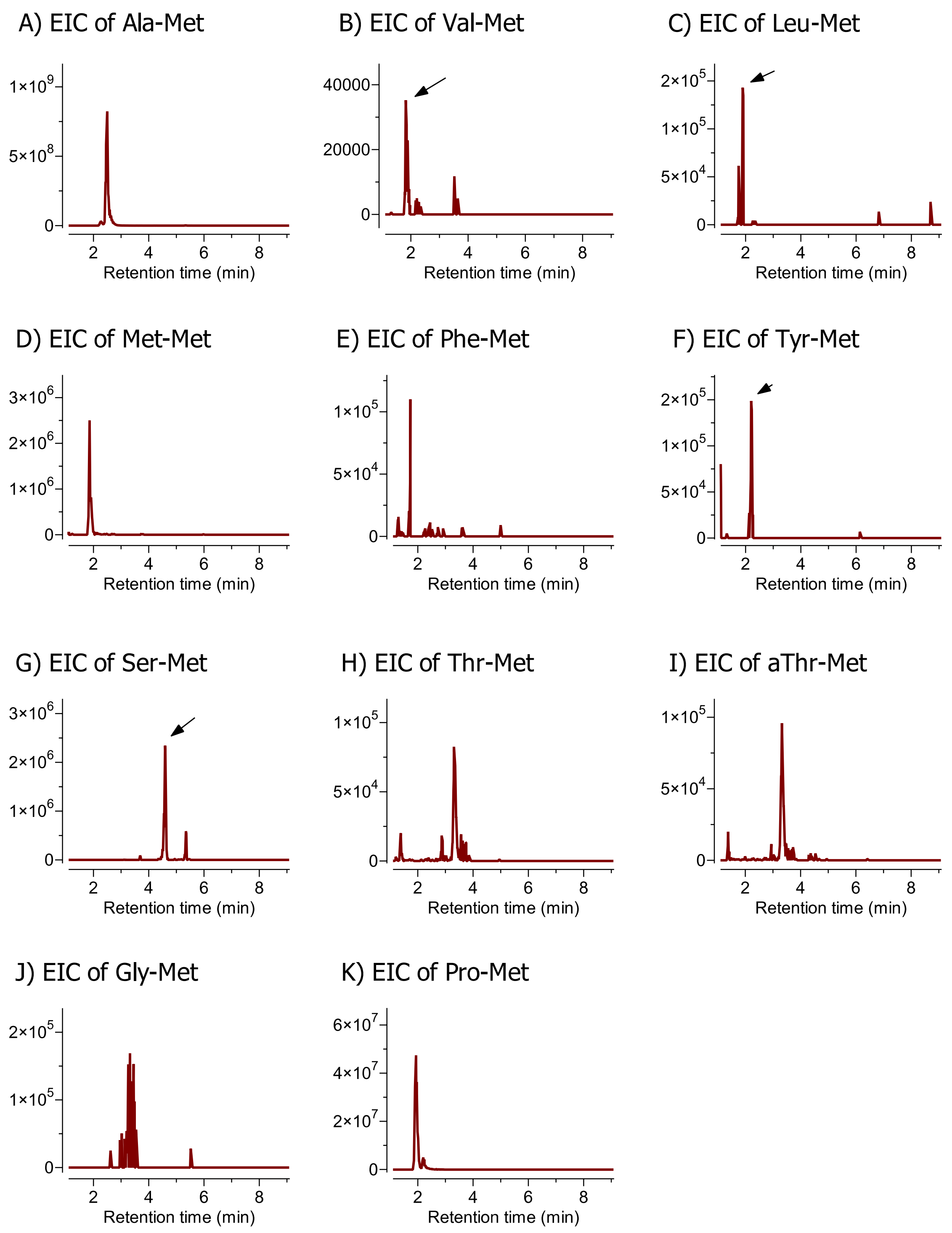

Figure S28. LC-HRMS detection of X-Met dipeptides synthesized in PnaC reactions

A) Ala-Met 221.0954 *m/z*, B) Val-Met 249.1267 *m/z*, C) Leu-Met 263.1424 *m/z*, D) Met-Met 281.0988 *m/z*, E) Phe-Met 297.1267 *m/z*, F) Tyr-Met 313.1217 *m/z*, G) Ser-Met 237.0904 *m/z*, H) Thr-Met 251.1060 *m/z*, I) aThr-Met 251.1060 *m/z*, J) Gly-Met 207.0798 *m/z*, K) Pro-Met 247.1111 *m/z*

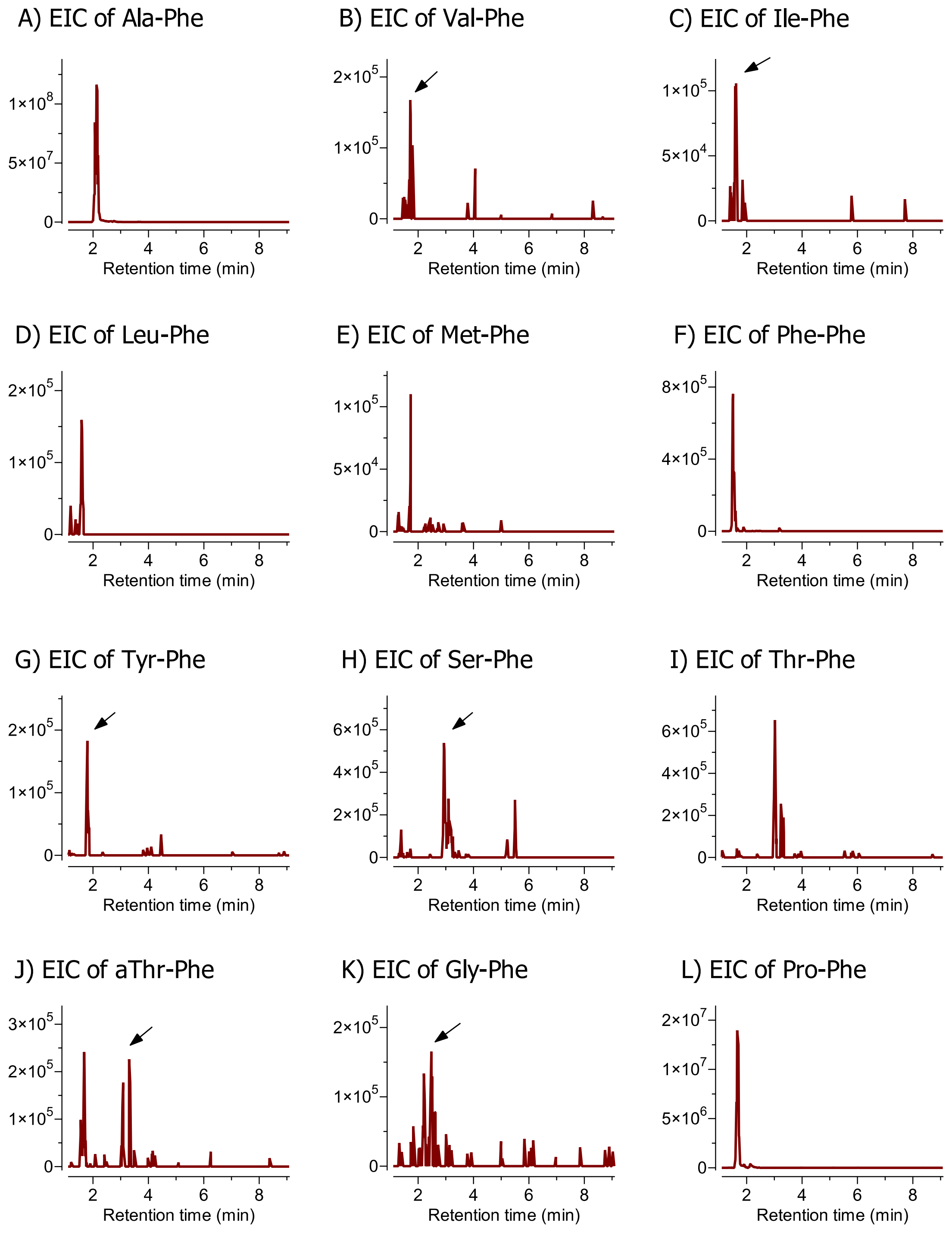

Figure S29. LC-HRMS detection of X-Phe dipeptides synthesized in PnaC reactions
A) Ala-Phe 237.1234 *m/z*, B) Val-Phe 265.1547 *m/z*, C) Ile-Phe 279.1703 *m/z*, D) Leu-Phe 279.1703 *m/z*, E) Met-Phe 297.1267 *m/z*, F) Phe-Phe 313.1547 *m/z*, G) Tyr-Phe 329.1496 *m/z*, H) Ser-Phe 253.1183 *m/z*, I) Thr-Phe 267.1339 *m/z*, J) aThr-Phe 267.1339 *m/z*, K) Gly-Phe 223.1077 *m/z*, L) Pro-Phe 263.1390 *m/z*

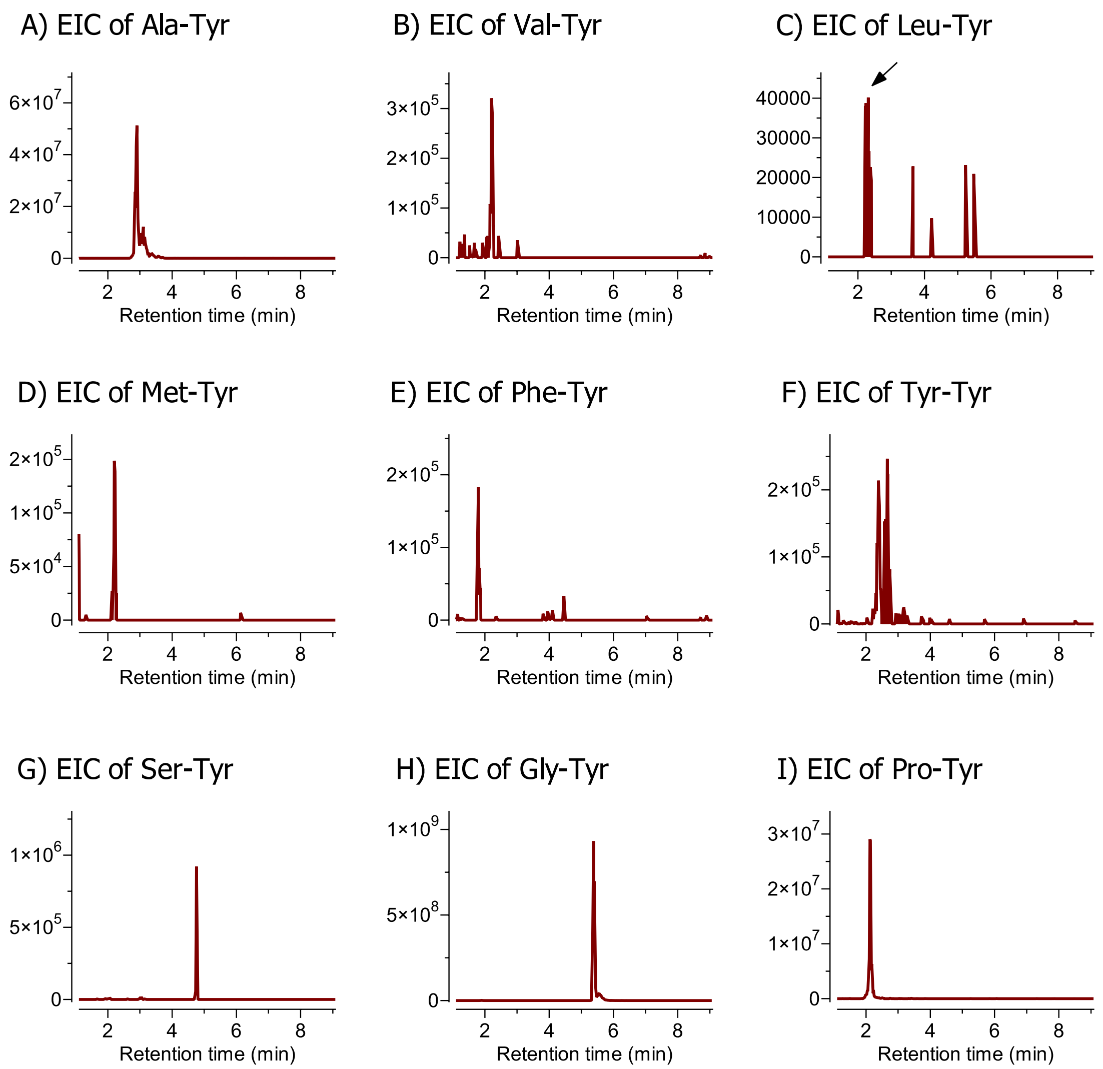

Figure S30. LC-HRMS detection of X-Tyr dipeptides synthesized in PnaC reactions

A) Ala-Tyr 253.1183 *m/z*, B) Val-Tyr 281.1497 *m/z*, C) Leu-Tyr 295.1652 *m/z*, D) Met-Tyr 313.1217 *m/z*, E) Phe-Tyr 329.1496 *m/z*, F) Tyr-Tyr 345.1445 *m/z*, G) Ser-Tyr 269.1132 *m/z*, H) Gly-Tyr 239.1026 *m/z*, I) Pro-Tyr 279.1339 *m/z*

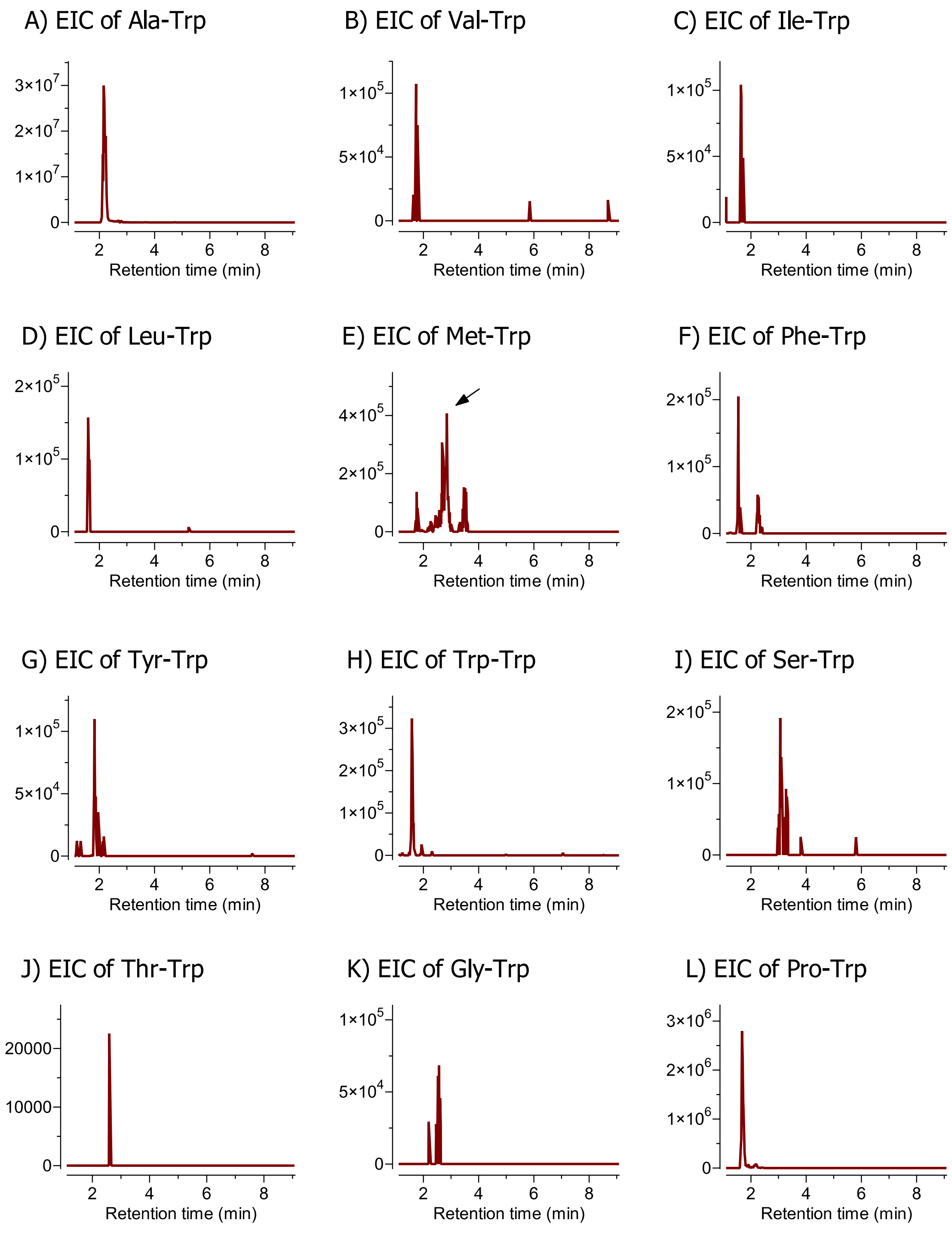

Figure S31. LC-HRMS detection of X-Trp dipeptides synthesized in PnaC reactions

A) Ala-Trp 276.1343 *m/z*, B) Val-Trp 304.1656 *m/z*, C) Ile-Trp 318.1812 *m/z*, D) Leu-Trp 318.1812 *m/z*, E) Met-Trp 336.1376 *m/z*, F) Phe-Trp 352.1656 *m/z*, G) Tyr-Trp 368.1605 *m/z*, H) Trp-Trp 391.1765 *m/z*, I) Ser-Trp 292.1292 *m/z*, J) Thr-Trp 306.1448 *m/z*, K) Gly-Trp 262.1186 *m/z*, L) Pro-Trp 302.1499 *m/z*

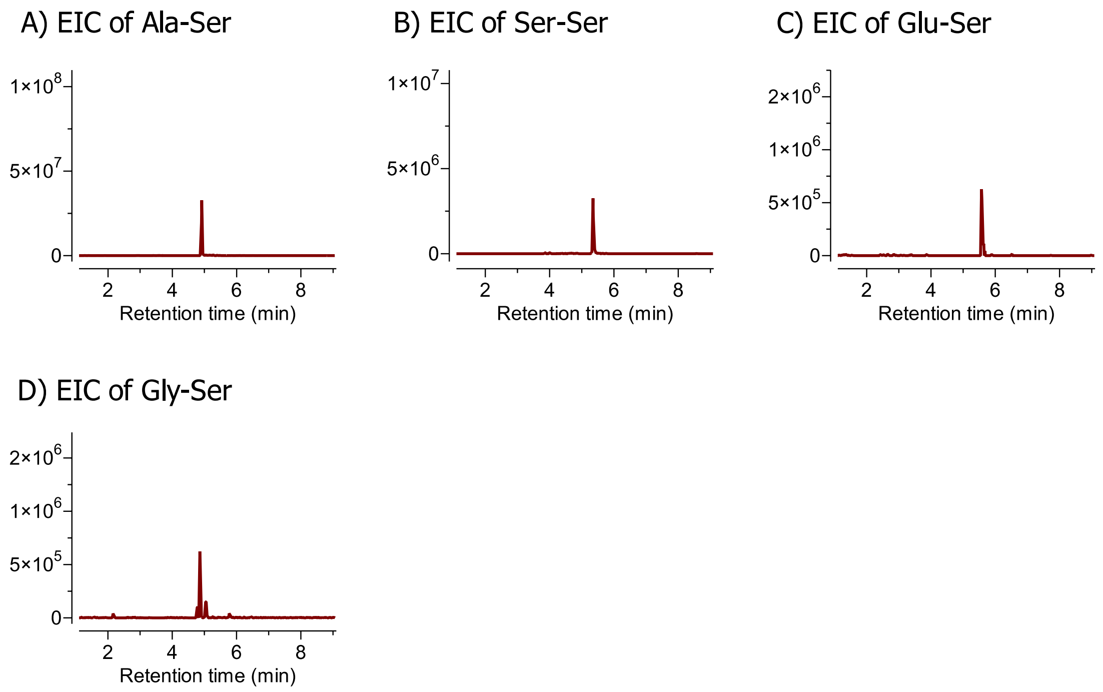

Figure S32. LC-HRMS detection of X-Ser dipeptides synthesized in PnaC reactions

A) Ala-Ser 177.0870 *m/z*, B) Ser-Ser 193.0819 *m/z*, C) Glu-Ser 235.0925 *m/z*, D) Gly-Ser 163.0713 *m/z*

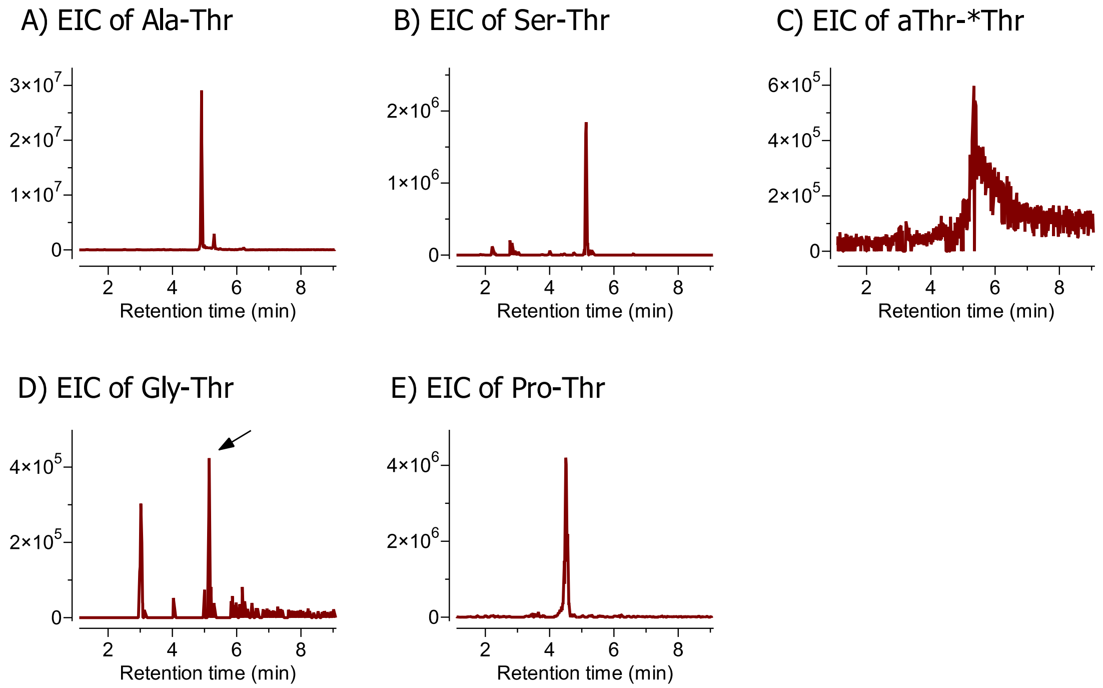

Figure S33. LC-HRMS detection of X-Thr dipeptides synthesized in PnaC reactions

A) Ala-Thr 191.1026 *m/z*, B) Ser-Thr 207.0976 *m/z*, C) aThr-(4-^{13}C-2,3-D_2-Thr) 224.1291 *m/z*, D) Gly-Thr 177.0870 *m/z*, E) Pro-Thr 217.1183 *m/z*

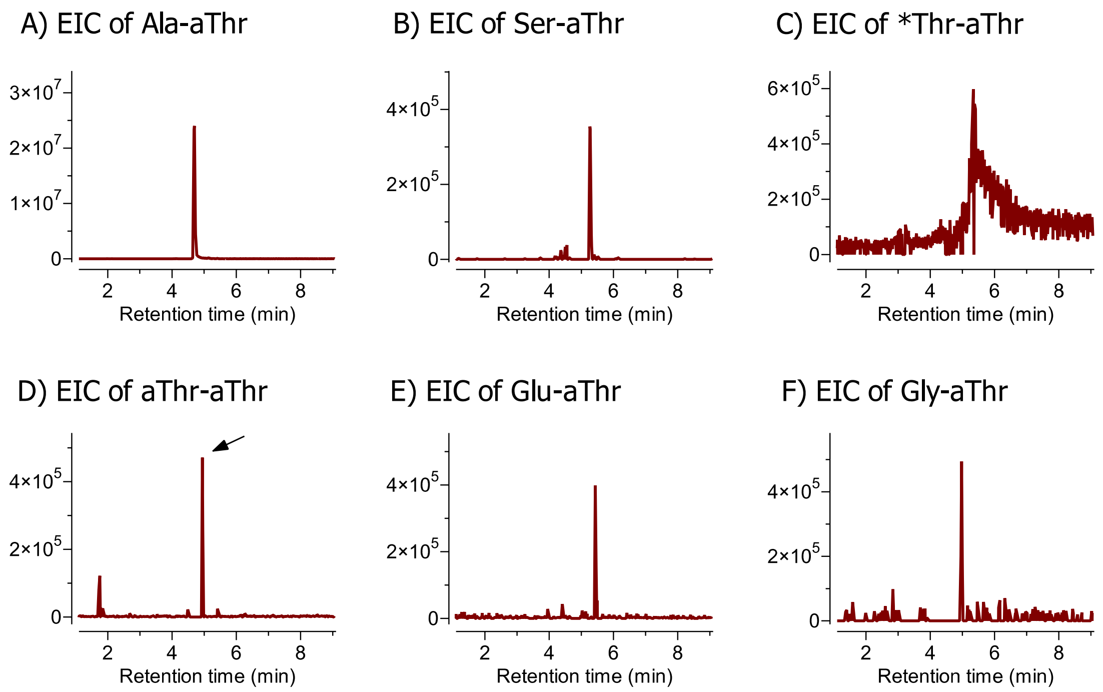

Figure S34. LC-HRMS detection of X-aThr dipeptides synthesized in PnaC reactions

A) Ala-aThr 191.1026 *m/z*, B) Ser-aThr 207.0976 *m/z*, C) (4-^{13}C-2,3-D_2-Thr)-aThr 224.1291 *m/z*, D) aThr-aThr 224.1291 *m/z*, E) Glu-aThr 249.1081 *m/z*, F) Gly-aThr 177.0870 *m/z*

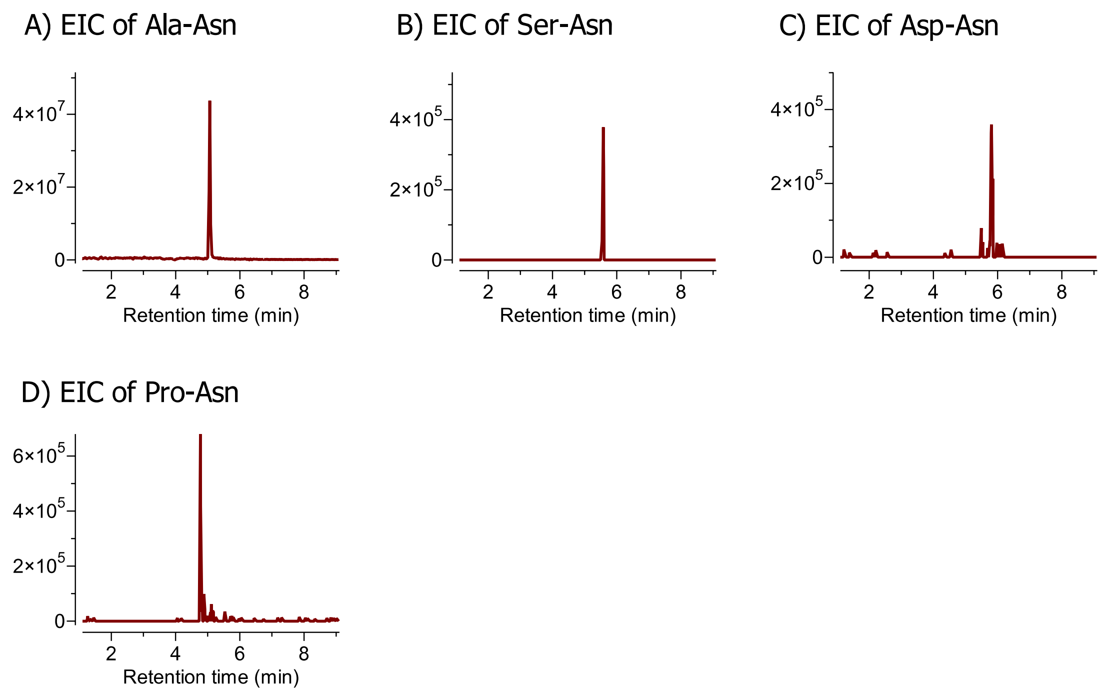

Figure S35. LC-HRMS detection of X-Asn dipeptides synthesized in PnaC reactions

A) Ala-Asn 204.0979 *m/z*, B) Ser-Asn 220.0928 *m/z*, C) Asp-Asn 248.0877 *m/z*, D) Pro-Asn 230.1135 *m/z*

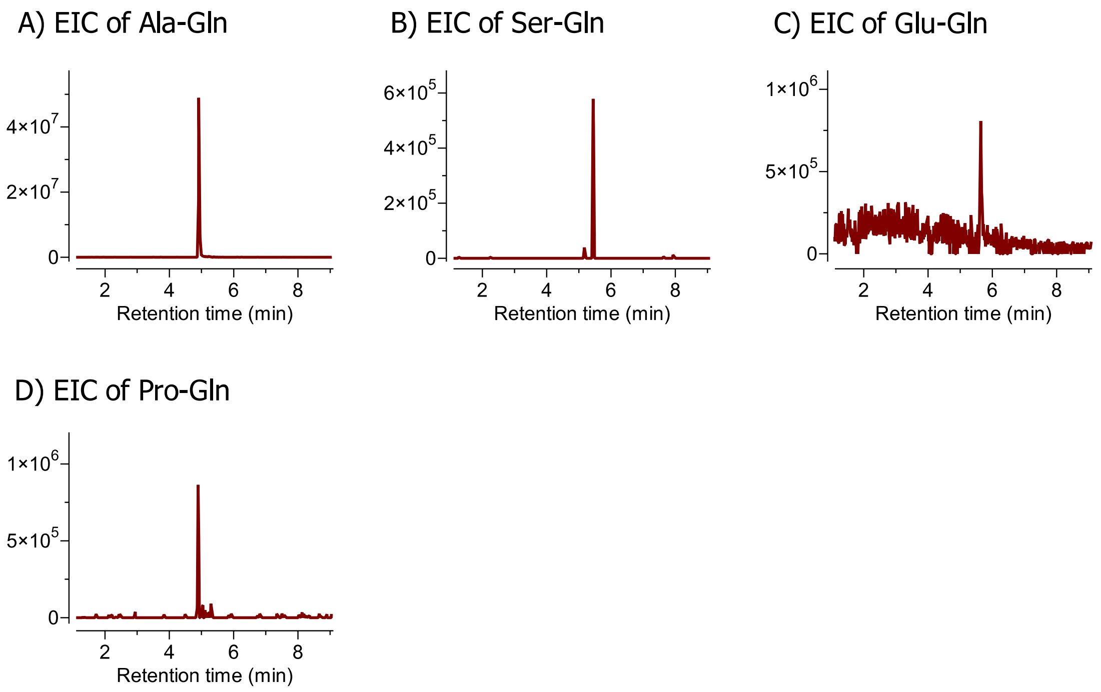

Figure S36. LC-HRMS detection of X-Gln dipeptides synthesized in PnaC reactions

A) Ala-Gln 218.1134 *m/z*, B) Ser-Gln 234.1085 *m/z*, C) Glu-Gln 276.1190 *m/z*, D) Pro-Gln 244.1292 *m/z*

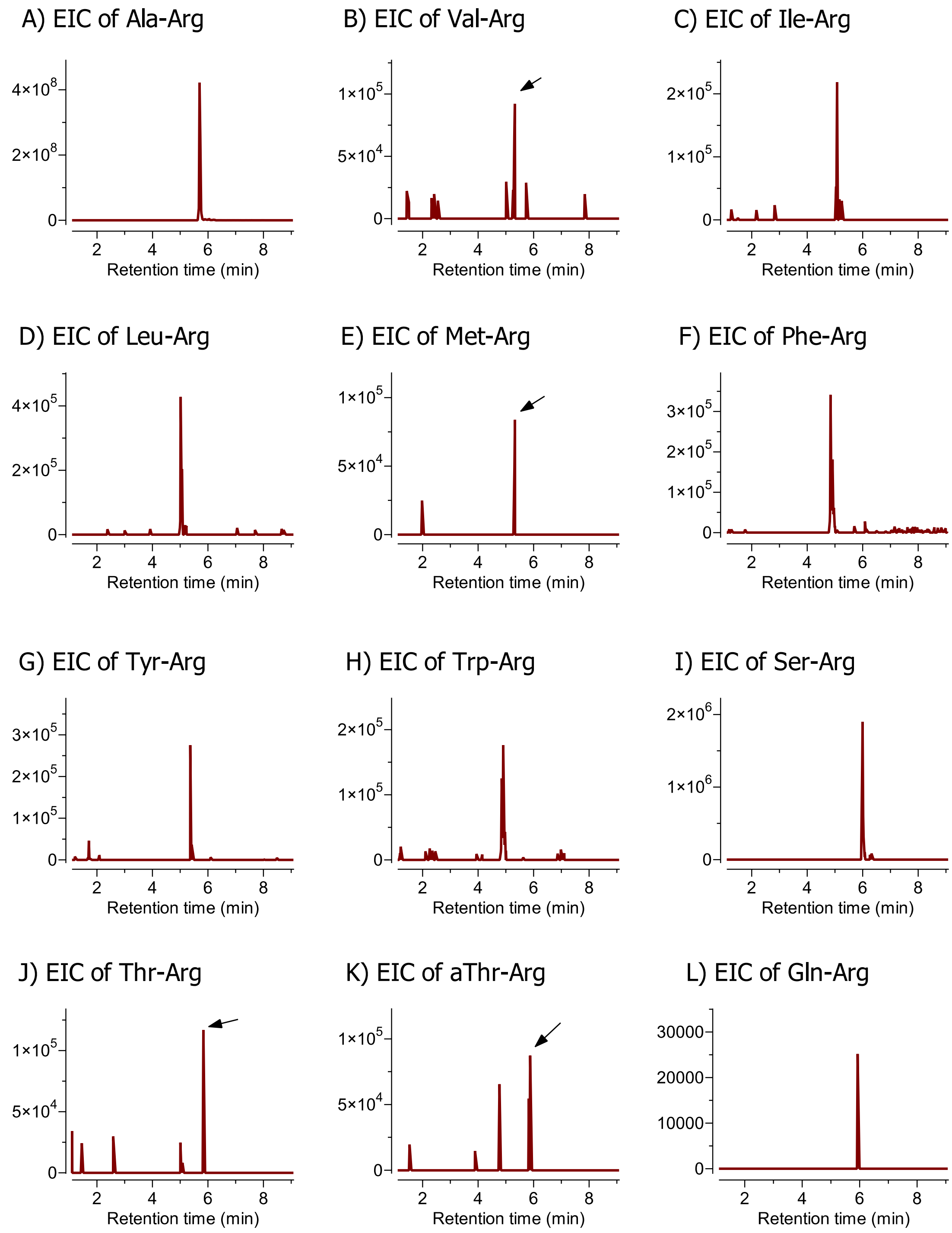

Figure S37. LC-HRMS detection of X-Arg dipeptides synthesized in PnaC reactions

A) Ala-Arg 246.1561 *m/z*, B) Val-Arg 274.1874 *m/z*, C) Ile-Arg 288.2030 *m/z*, D) Leu-Arg 288.2030 *m/z*, E) Met-Arg 306.1594 *m/z*, F) Phe-Arg 322.1874 *m/z*, G) Tyr-Arg 338.1823 *m/z*, H) Trp-Arg 361.1983 *m/z*, I) Ser-Arg 262.1510 *m/z*, J) Thr-Arg 276.1666 *m/z*, K) aThr-Arg 276.1666 *m/z*, L) Gln-Arg 303.1775 *m/z*

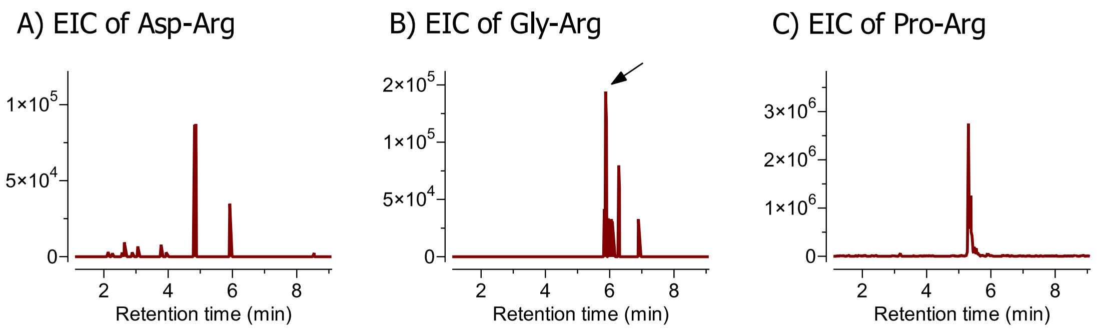

Figure S38. LC-HRMS detection of X-Arg dipeptides synthesized in PnaC reactions, cntd.
A) Asp-Arg 290.1459 *m/z*, B) Gly-Arg 232.1404 *m/z*, C) Pro-Arg 272.1717 *m/z*

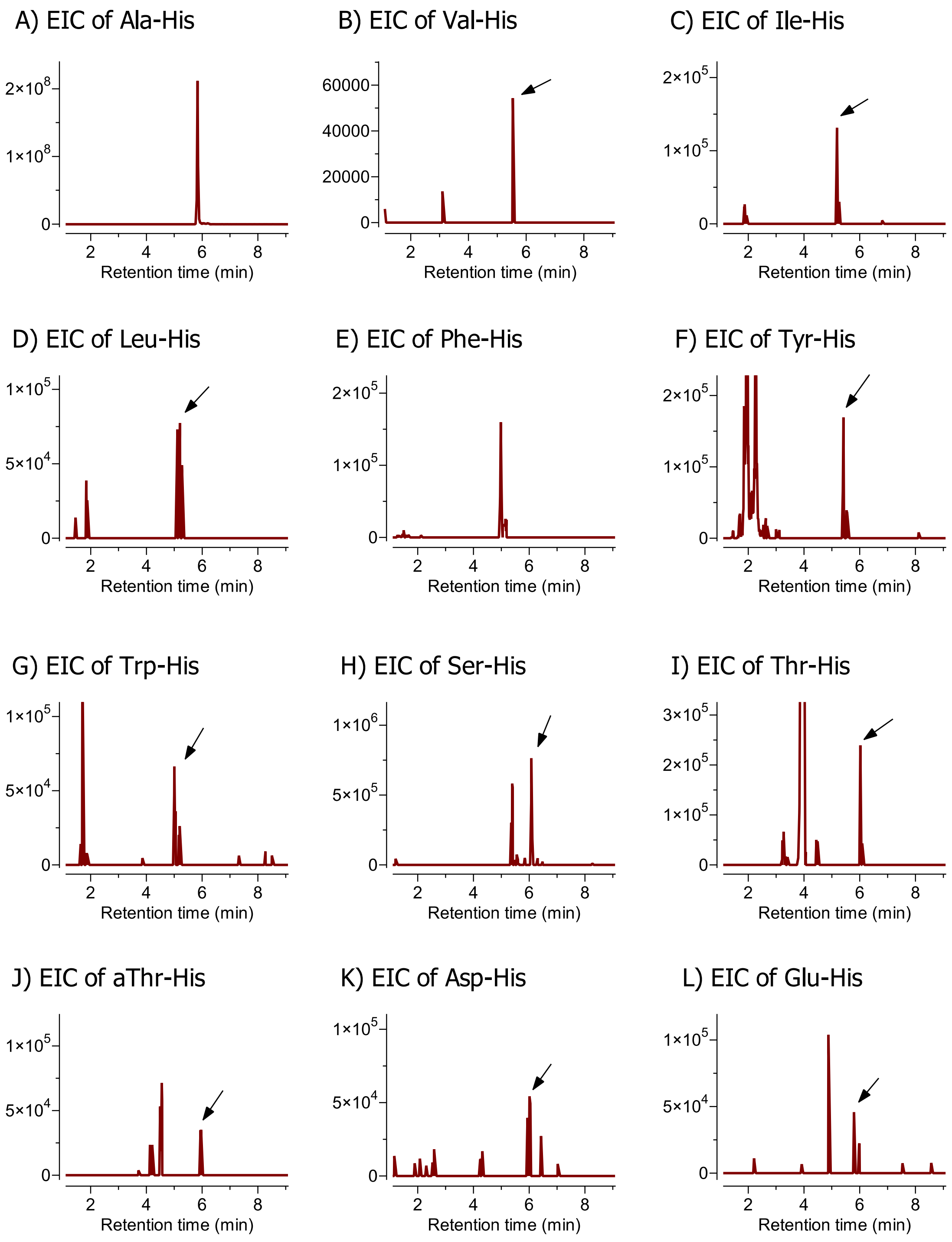

Figure S39. LC-HRMS detection of X-His dipeptides synthesized in PnaC reactions

A) Ala-His 227.1139 *m/z*, B) Val-His 255.1452 *m/z*, C) Ile-His 269.1608 *m/z*, D) Leu-His 269.1608 *m/z*, E) Phe-His 303.1452 *m/z*, F) Tyr-His 319.1401 *m/z*, G) Trp-His 342.1561 *m/z*, H) Ser-His 243.1077 *m/z*, I) Thr-His 257.1244 *m/z*, J) aThr-His 257.1244 *m/z*, K) Asp-His 271.1037 *m/z*, L) Glu-His 284.1194 *m/z*

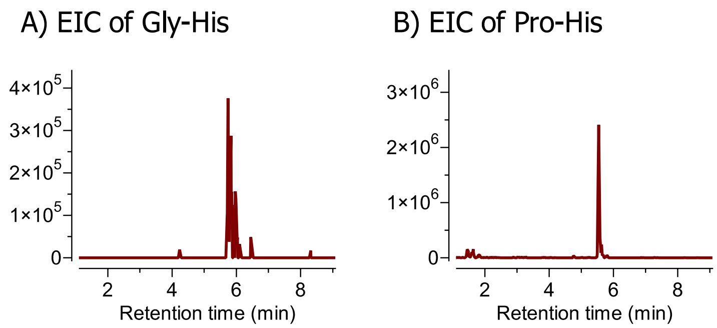

Figure S40. LC-HRMS detection of X-His dipeptides synthesized in PnaC reactions, cntd.
A) Gly-His 213.0982 *m/z*, B) Pro-His 253.1295 *m/z*

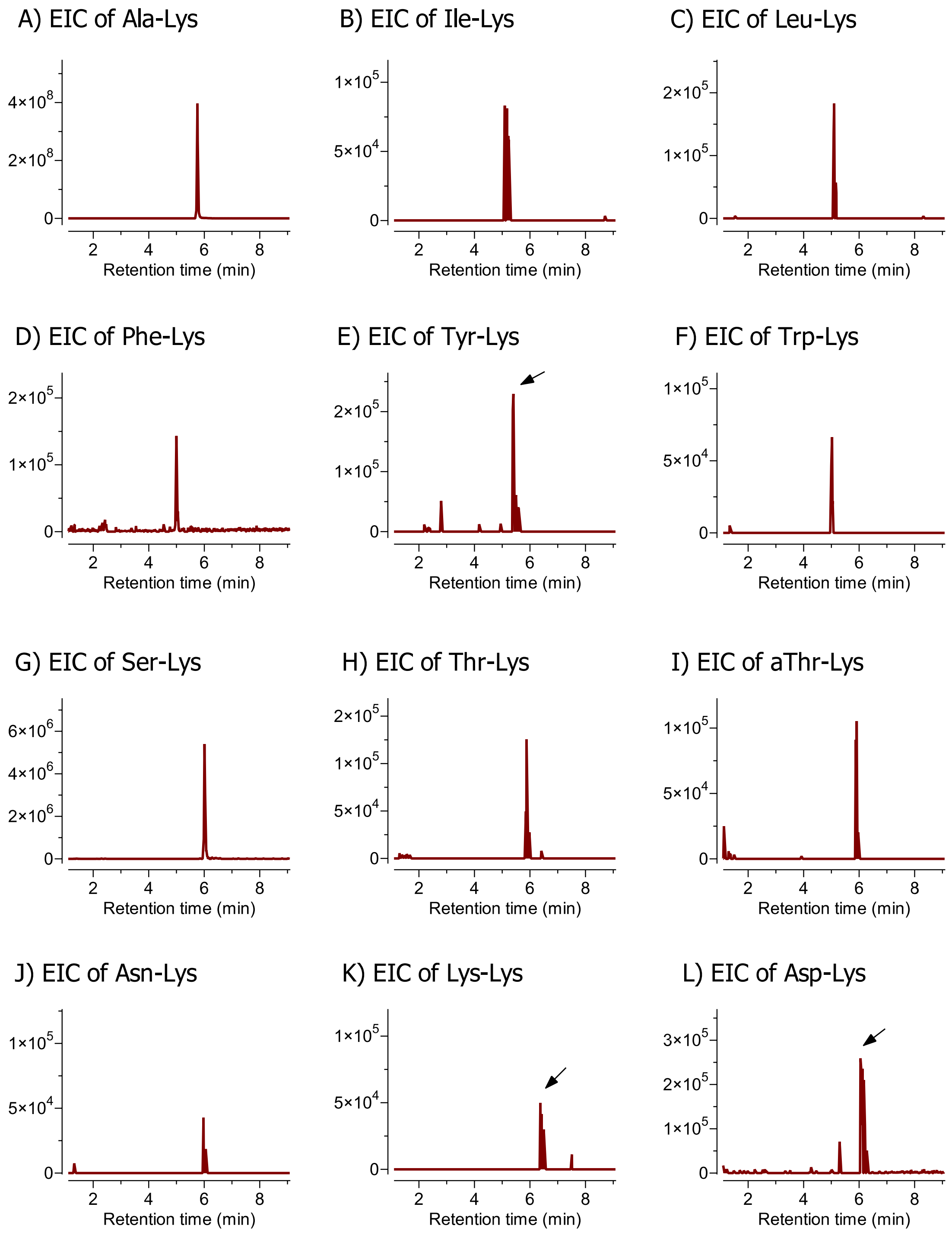

Figure S41. LC-HRMS detection of X-Lys dipeptides synthesized in PnaC reactions

A) Ala-Lys 218.1499 *m/z*, B) Ile-Lys 260.1969 *m/z*, C) Leu-Lys 260.1969 *m/z*, D) Phe-Lys 294.1812 *m/z*, E) Tyr-Lys 310.1761 *m/z*, F) Trp-Lys 333.1921 *m/z*, G) Ser-Lys 234.1448 *m/z*, H) Thr-Lys 248.1605 *m/z*, I) aThr-Lys 248.1605 *m/z*, J) Asn-Lys 261.1557 *m/z*, K) Lys-Lys 275.2078 *m/z*, L) Asp-Lys 262.1393 *m/z*

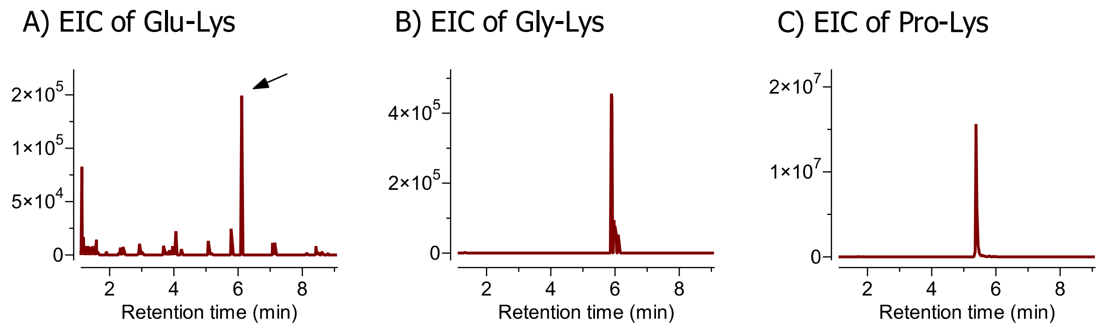

Figure S42. LC-HRMS detection of X-Lys dipeptides synthesized in PnaC reactions, cntd.
A) Glu-Lys 267.1554 *m/z*, B) Gly-Lys 204.1343 *m/z*, C) Pro-Lys 244.1656 *m/z*

A) EIC of Asp-Asp

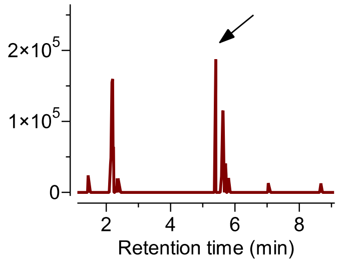

Figure S43. LC-HRMS detection of X-Asp dipeptides synthesized in PnaC reactions
A) Asp-Asp 249.0717 *m/z*

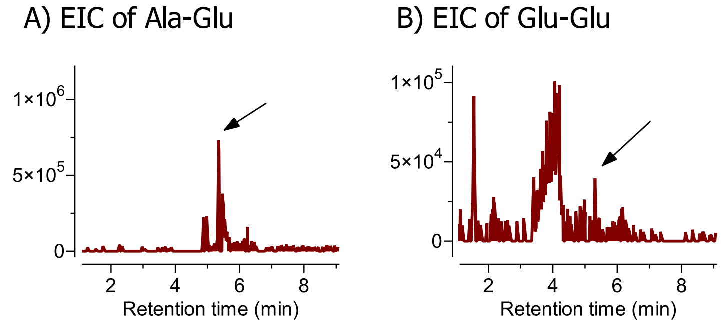

Figure S44. LC-HRMS detection of X-Glu dipeptides synthesized in PnaC reactions

A) Ala-Glu 219.0976 *m/z*, B) Glu-Glu 277.1030 *m/z*

A) EIC of Ala-Cys

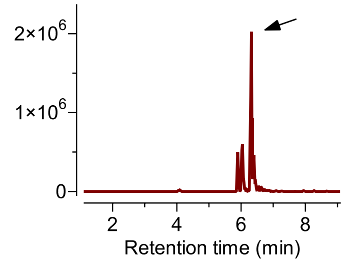

Figure S45. LC-HRMS detection of X-Cys dipeptides synthesized in PnaC reactions
A) Ala-Cys 193.0641 *m/z*

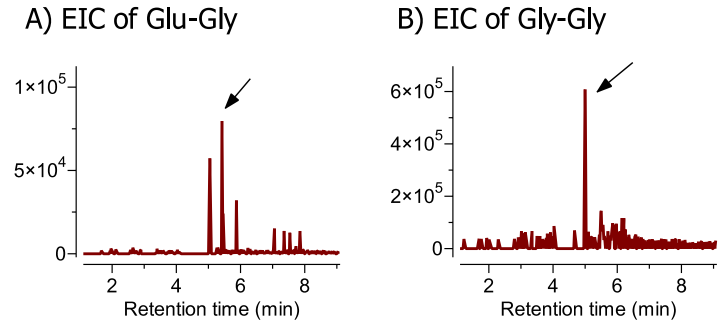

Figure S46. LC-HRMS detection of X-Gly dipeptides synthesized in PnaC reactions

A) Glu-Gly 205.0819 *m/z*, B) Gly-Gly 133.0608 *m/z*,

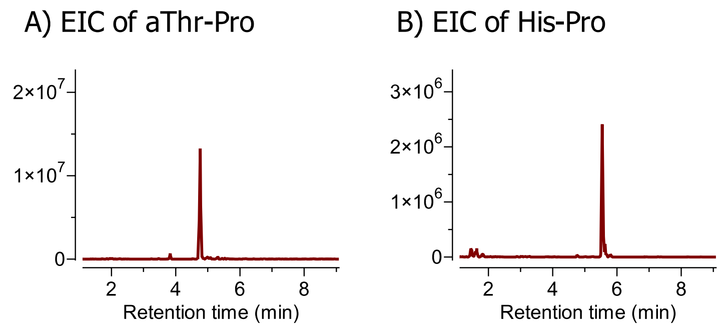

Figure S47. LC-HRMS detection of X-Pro dipeptides synthesized in PnaC reactions

A) aThr-Pro 217.1183 *m/z*, B) His-Pro 253.1295 *m/z*

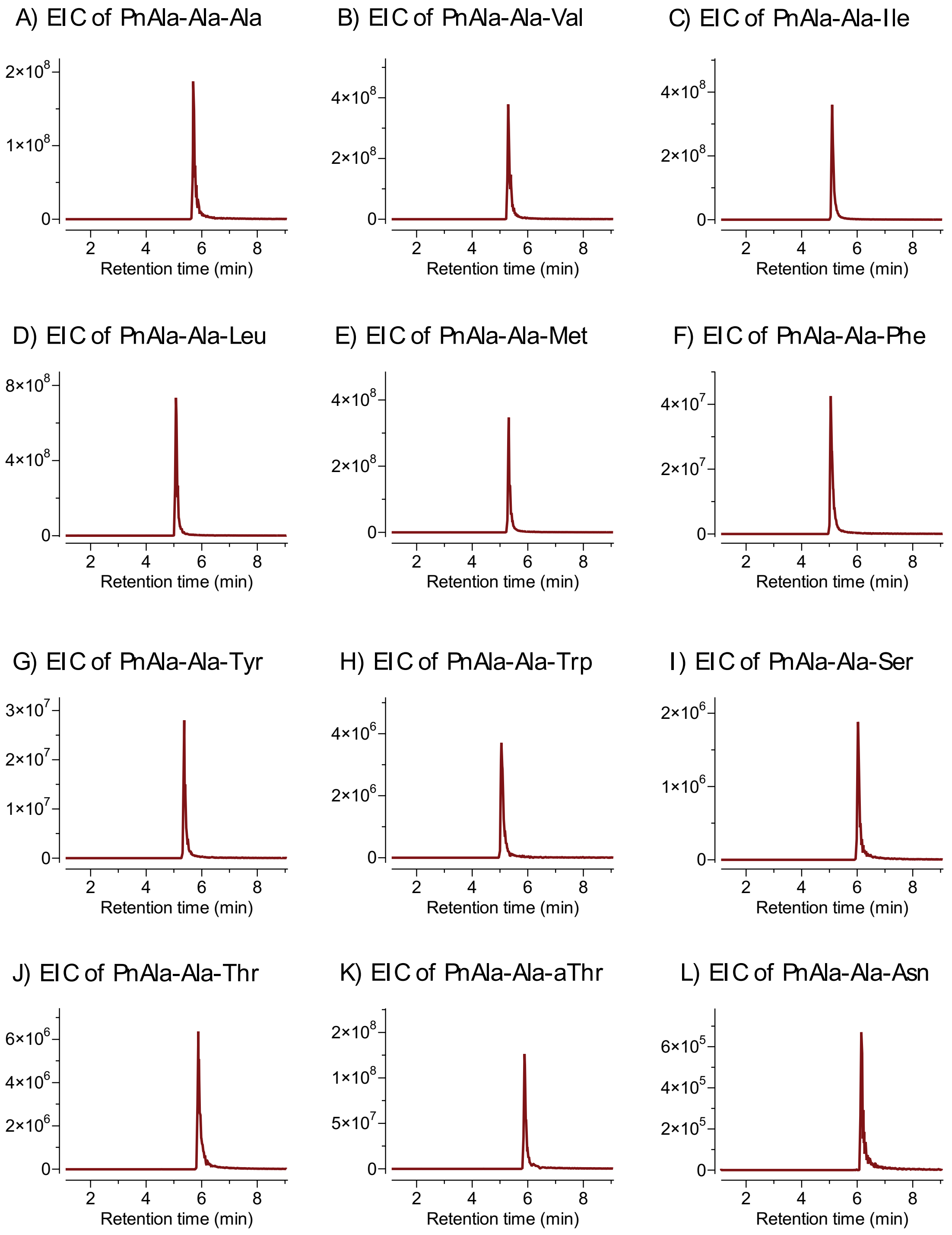

Figure S48. LC-HRMS detection of PnAla-Ala-X tripeptides synthesized in PnaBC reactions

A) PnAla-Ala-Ala 312.0961 *m/z*, B) PnAla-Ala-Val 340.1274 *m/z*, C) PnAla-Ala-Ile 354.1430 *m/z*, D) PnAla-Ala-Leu 354.1430 *m/z*, E) PnAla-Ala-Met 372.0994 *m/z*, F) PnAla-Ala-Phe 388.1274 *m/z*, G) PnAla-Ala-Tyr 404.1223 *m/z*, H) PnAla-Ala-Trp 427.1383 *m/z*, I) PnAla-Ala-Ser 328.0910 *m/z*, J) PnAla-Ala-Thr 342.1061 *m/z*, K) PnAla-Ala-aThr 342.1061 *m/z*, L) PnAla-Ala-Asn 355.1019 *m/z*

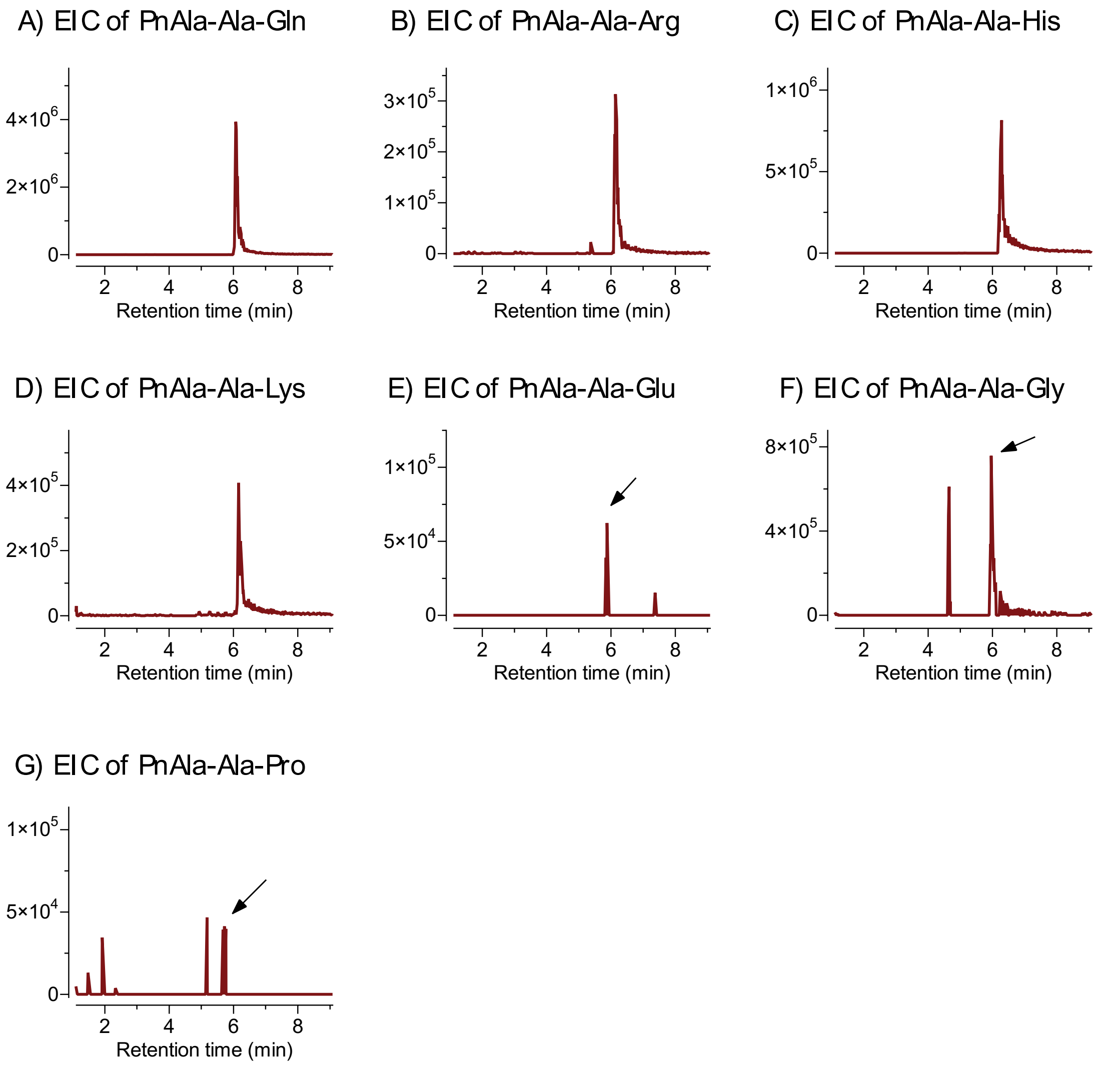

Figure S49. LC-HRMS detection of PnAla-Ala-X tripeptides synthesized in PnaBC reactions, cntd.

A) PnAla-Ala-Gln 369.1175 *m/z*, B) PnAla-Ala-Arg 397.1601 *m/z*, C) PnAla-Ala-His 378.1179 *m/z*, D) PnAla-Ala-Lys 369.1539 *m/z*, E) PnAla-Ala-Glu 370.1010 *m/z*, F) PnAla-Ala-Gly 298.0799 *m/z*, G) PnAla-Ala-Pro 338.1112 *m/z*

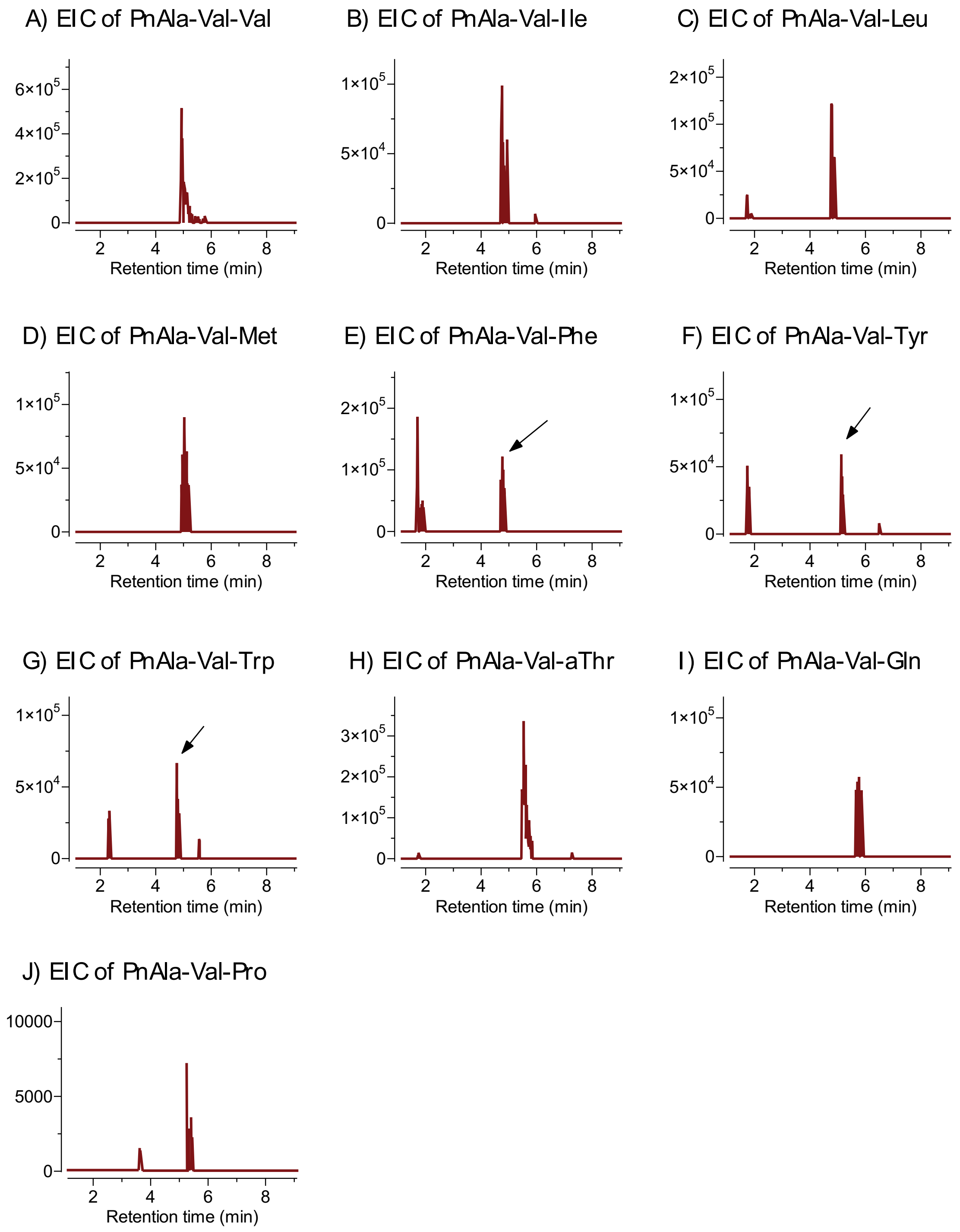

Figure S50. LC-HRMS detection of PnAla-Val-X tripeptides synthesized in PnaBC reactions
A) PnAla-Val-Val 368.1587 *m/z*, B) PnAla-Val-Ile 382.1738 *m/z*, C) PnAla-Val-Leu 382.1738 *m/z*, D) PnAla-Val-Met 400.1302 *m/z*, E) PnAla-Val-Phe 416.1581 *m/z*, F) PnAla-Val-Tyr 432.1530 *m/z*, G) PnAla-Val-Trp 455.1690 *m/z*, H) PnAla-Val-aThr 370.1374 *m/z*, I) PnAla-Val-Gln 397.1483 *m/z*, J) PnAla-Val-Pro 366.1425 *m/z*

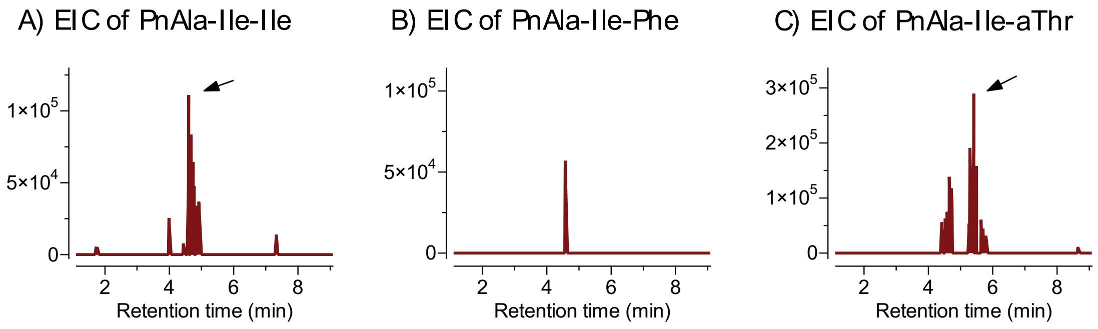

Figure S51. LC-HRMS detection of PnAla-Ile-X tripeptides synthesized in PnaBC reactions, cntd.

A) PnAla-Ile-Ile 396.1894 *m/z*, B) PnAla-Ile-Phe 430.1738 *m/z*, C) PnAla-Ile-aThr 384.1530 *m/z*

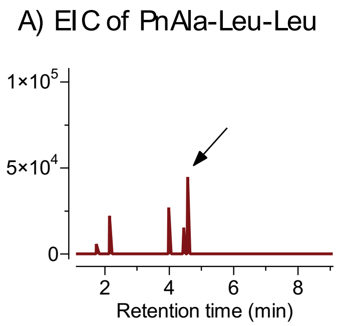

Figure S52. LC-HRMS detection of PnAla-Leu-X tripeptides synthesized in PnaBC reactions
A) PnAla-Leu-Leu 396.1894 *m/z*

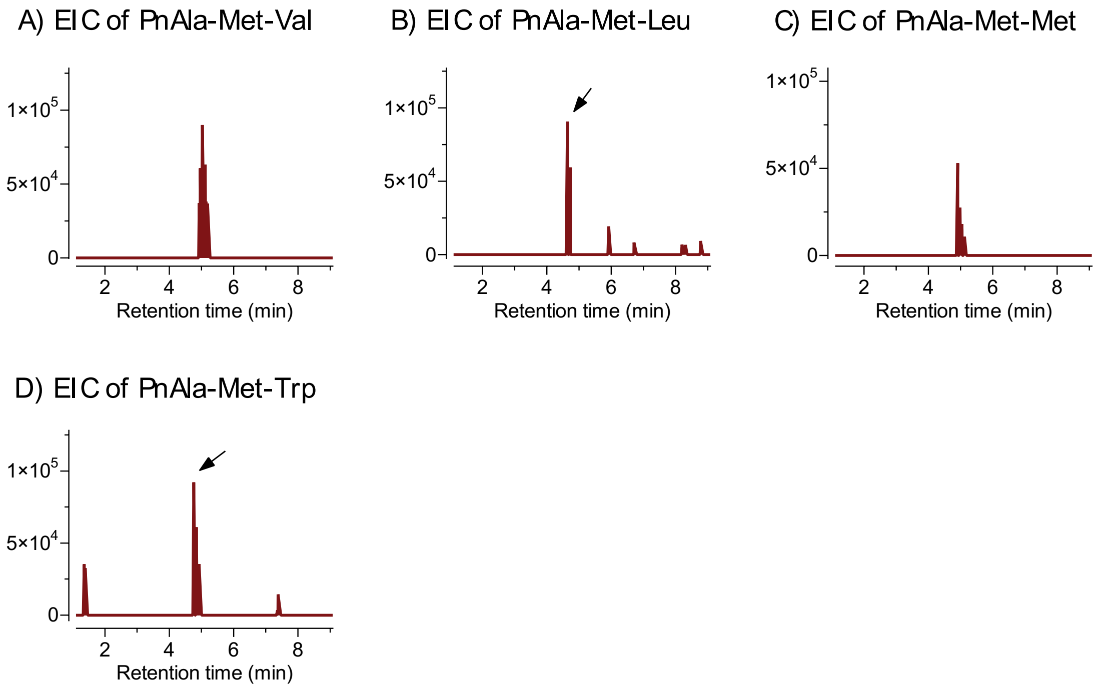

Figure S53. LC-HRMS detection of PnAla-Met-X tripeptides synthesized in PnaBC reactions
A) PnAla-Met-Val 400.1302 *m/z*, B) PnAla-Met-Leu 414.1458 *m/z*, C) PnAla-Met-Met 432.1028 *m/z*, D) PnAla-Met-Trp 487.1411 *m/z*

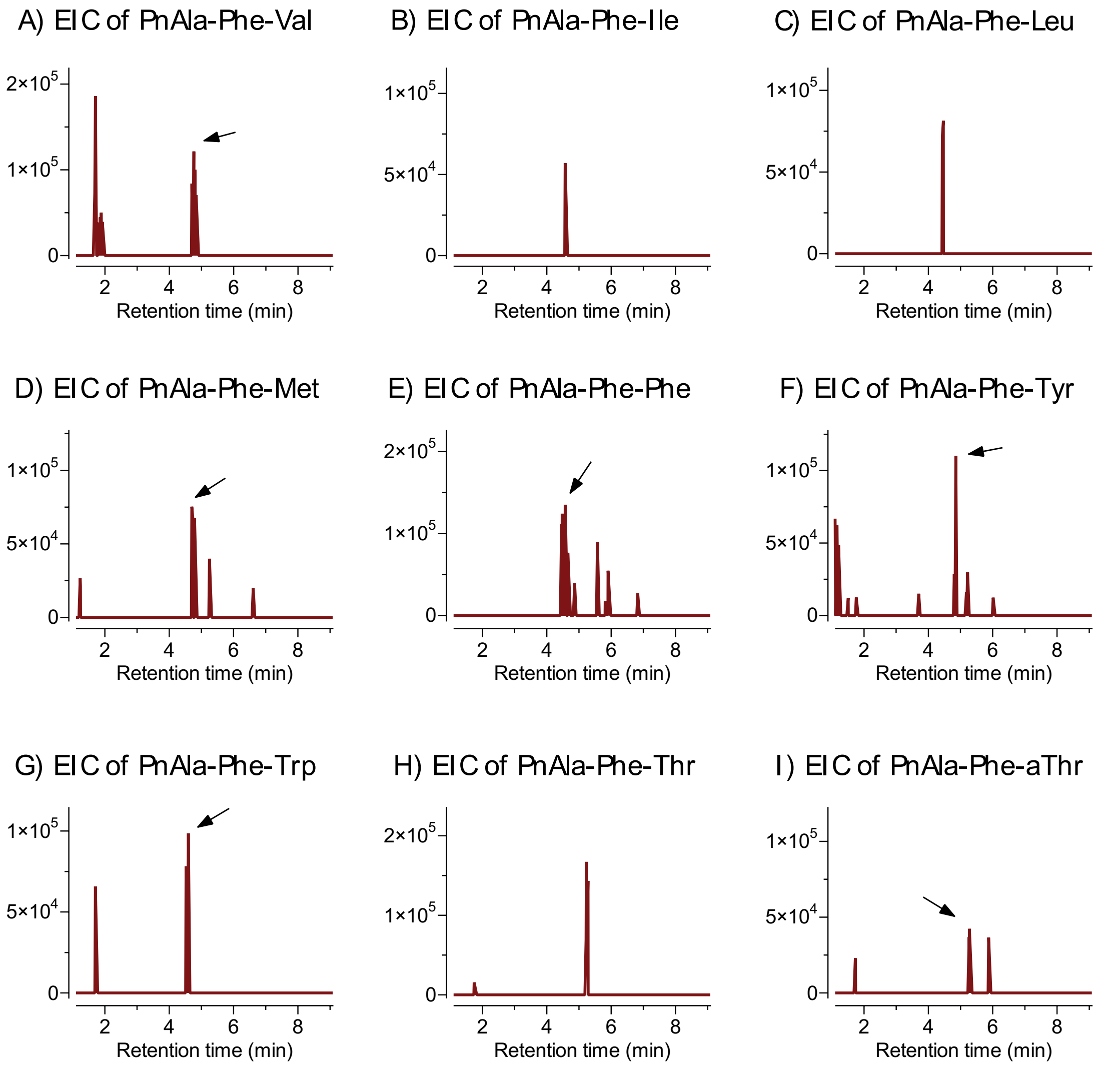

Figure S54. LC-HRMS detection of PnAla-Phe-X tripeptides synthesized in PnaBC reactions

A) PnAla-Phe-Val 416.1581 *m/z*, B) PnAla-Phe-Ile 430.1738 *m/z*, C) PnAla-Phe-Leu 430.1738 *m/z*, D) PnAla-Phe-Met 448.1302 *m/z*, E) PnAla-Phe-Phe 464.1581 *m/z*, F) PnAla-Phe-Tyr 480.1530 *m/z*, G) PnAla-Phe-Trp 503.1690 *m/z*, H) PnAla-Phe-Thr 418.1374 *m/z*, I) PnAla-Phe-aThr 418.1374 *m/z*

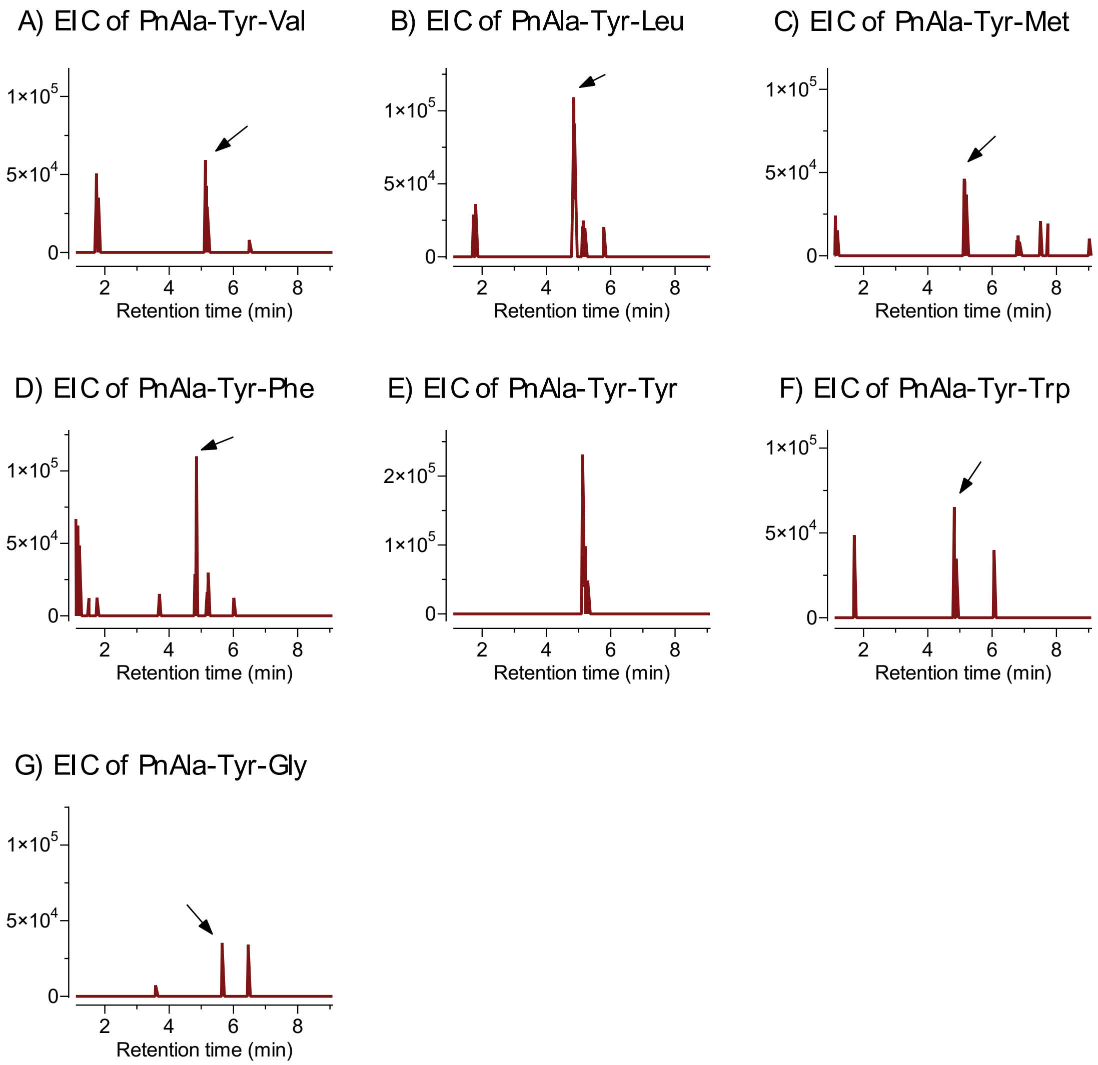

Figure S55. LC-HRMS detection of PnAla-Tyr-X tripeptides synthesized in PnaBC reactions

A) PnAla-Tyr-Val 432.1530 *m/z*, B) PnAla-Tyr-Leu 446.1687 *m/z*, C) PnAla-Tyr-Met 464.1251 *m/z*, D) PnAla-Tyr-Phe 480.1530 *m/z*, E) PnAla-Tyr-Tyr 496.1479 *m/z*, F) PnAla-Tyr-Trp 519.1639 *m/z*, G) PnAla-Tyr-Gly 390.1061 *m/z*

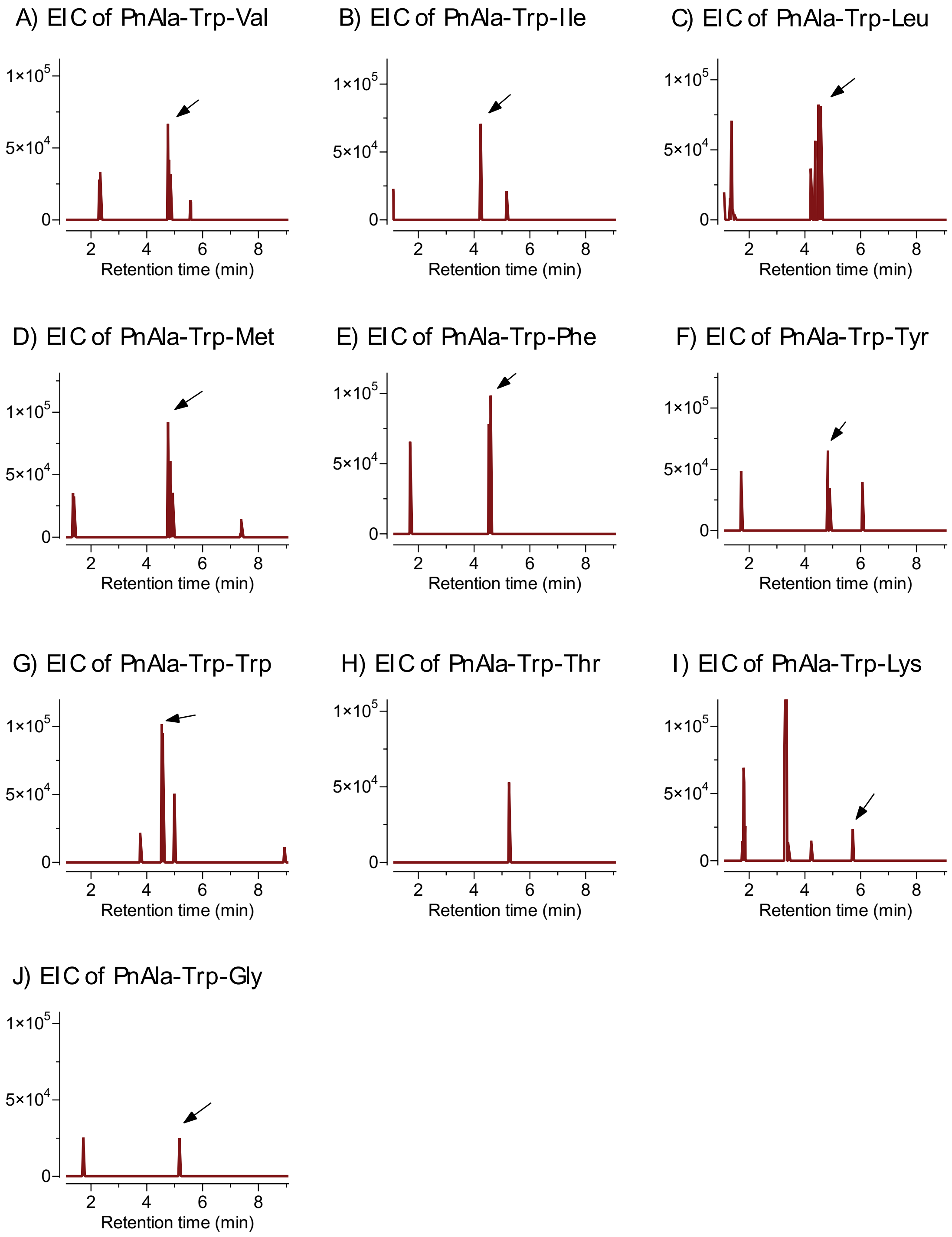

Figure S56. LC-HRMS detection of PnAla-Trp-X tripeptides synthesized in PnaBC reactions

A) PnAla-Trp-Val 455.1690 *m/z*, B) PnAla-Trp-Ile 469.1847 *m/z*, C) PnAla-Trp-Leu 469.1847 *m/z*, D) PnAla-Trp-Met 487.1411 *m/z*, E) PnAla-Trp-Phe 503.1690 *m/z*, F) PnAla-Trp-Tyr 519.1639 *m/z*, G) PnAla-Trp-Trp 542.1799 *m/z*, H) PnAla-Trp-Thr 457.1483 *m/z*, I) PnAla-Trp-Lys 484.1956 *m/z*, J) PnAla-Trp-Gly 413.1221 *m/z*

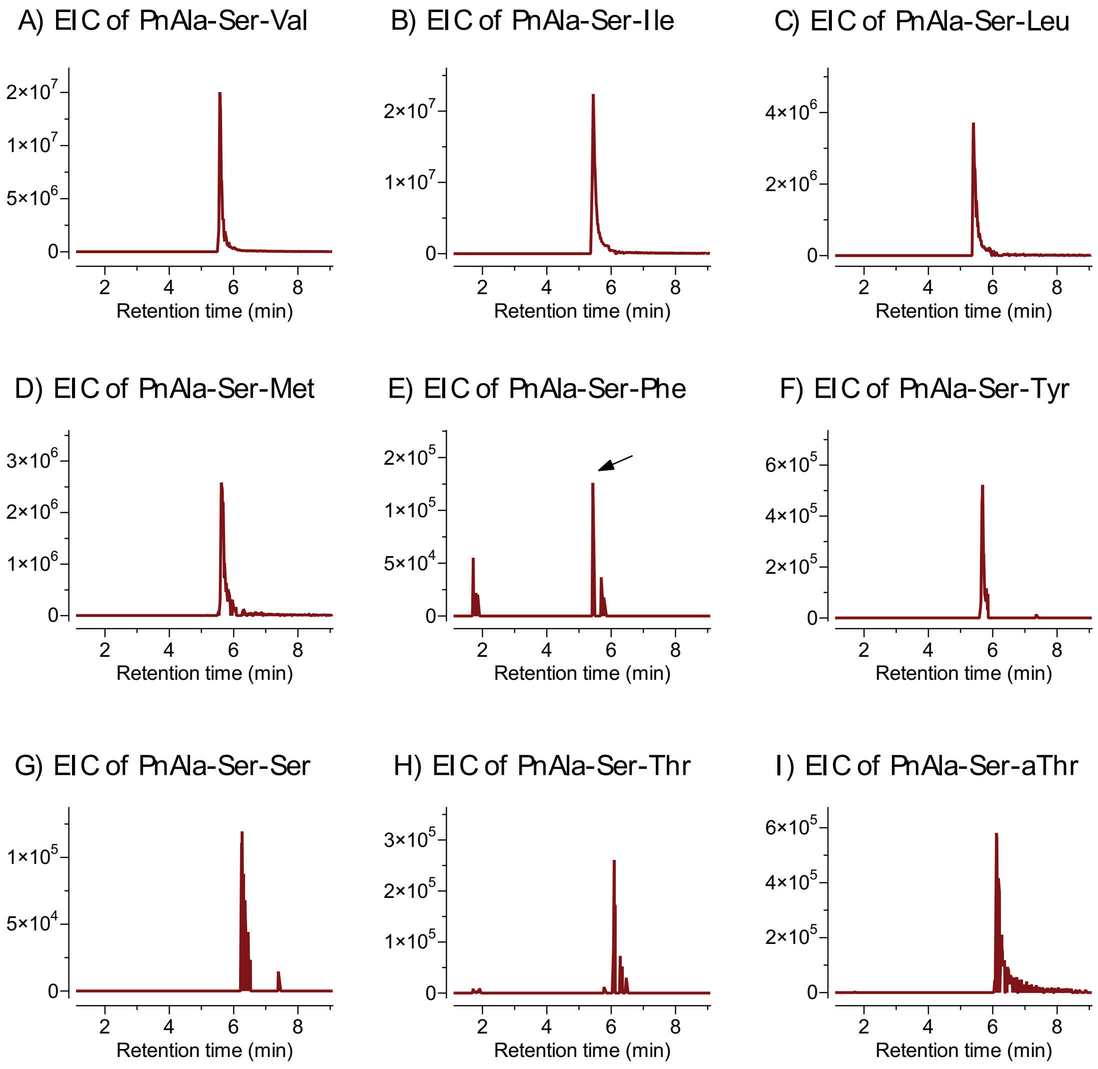

Figure S57. LC-HRMS detection of PnAla-Ser-X tripeptides synthesized in PnaBC reactions

A) PnAla-Ser-Val 356.1223 *m/z*, B) PnAla-Ser-Ile 370.1374 *m/z*, C) PnAla-Ser-Leu 370.1374 *m/z*, D) PnAla-Ser-Met 388.0938 *m/z*, E) PnAla-Ser-Phe 404.1217 *m/z*, F) PnAla-Ser-Tyr 420.1166 *m/z*, G) PnAla-Ser-Ser 344.0859 *m/z*, H) PnAla-Ser-Thr 358.1010 *m/z*, I) PnAla-Ser-aThr 358.1010 *m/z*

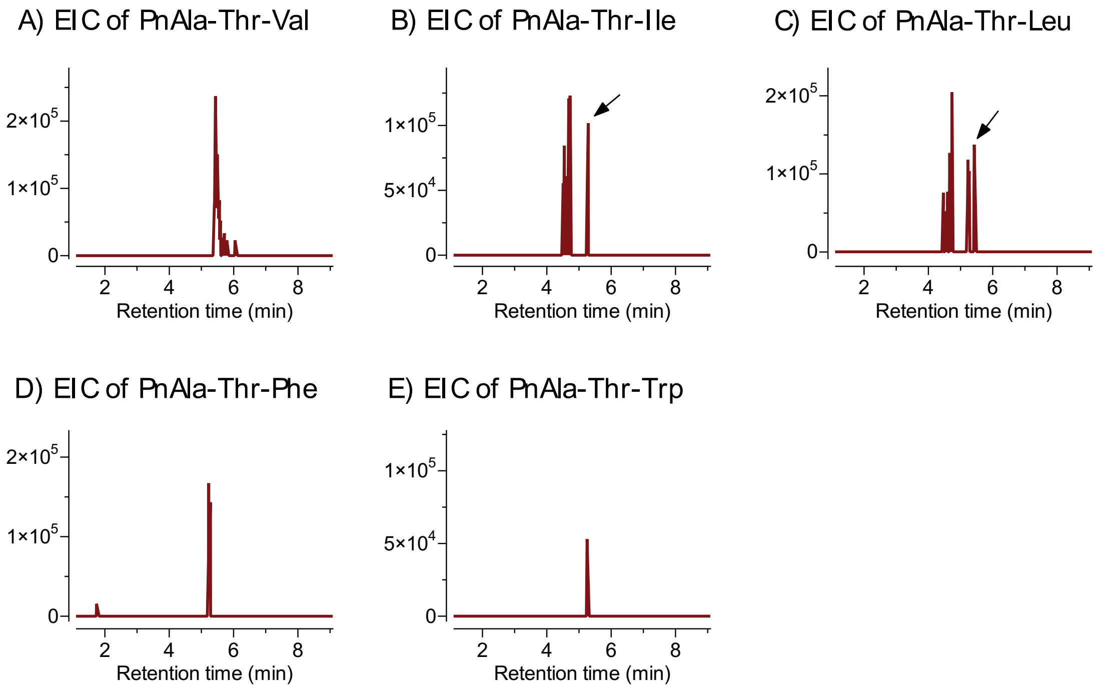

Figure S58. LC-HRMS detection of PnAla-Thr-X tripeptides synthesized in PnaBC reactions

A) PnAla-Thr-Val 370.1374 *m/z*, B) PnAla-Thr-Ile 384.1530 *m/z*, C) PnAla-Thr-Leu 384.1530 *m/z*, D) PnAla-Thr-Phe 418.1374 *m/z*, E) PnAla-Thr-Trp 457.1483 *m/z*

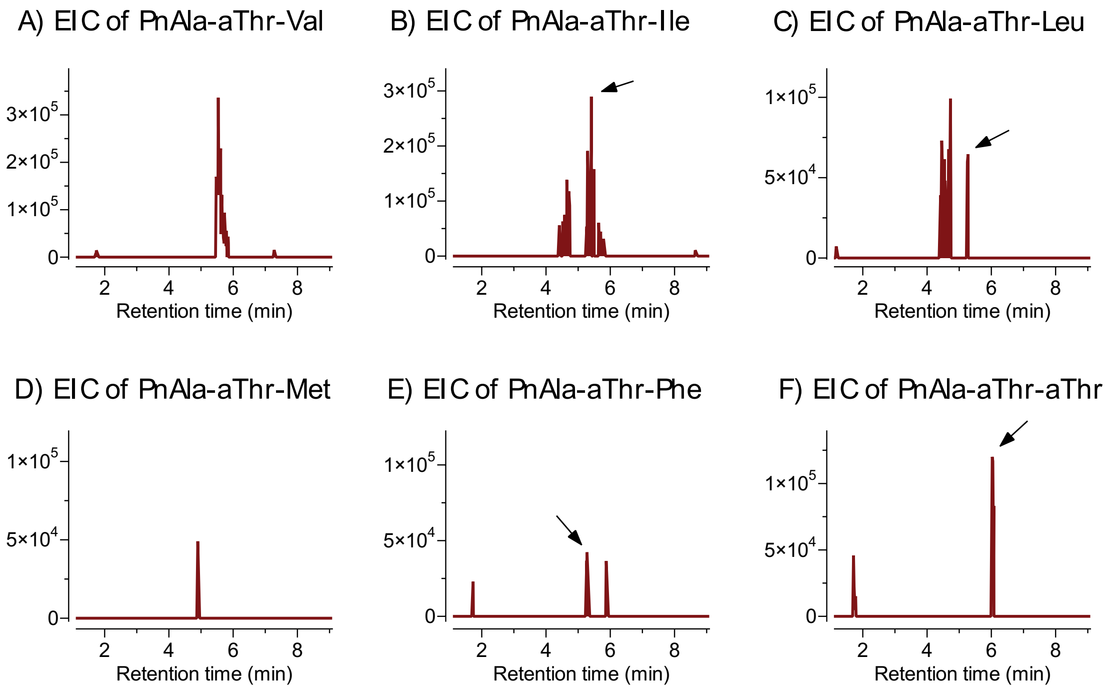

Figure S59. LC-HRMS detection of PnAla-aThr-X tripeptides synthesized in PnaBC reactions

A) PnAla-aThr-Val 370.1374 *m/z*, B) PnAla-aThr-Ile 384.1530 *m/z*, C) PnAla-aThr-Leu 384.1530 *m/z*, D) PnAla-aThr-Met 402.1095 *m/z*, E) PnAla-aThr-Phe 418.1374 *m/z*, F) PnAla-aThr-aThr 372.1166 *m/z*

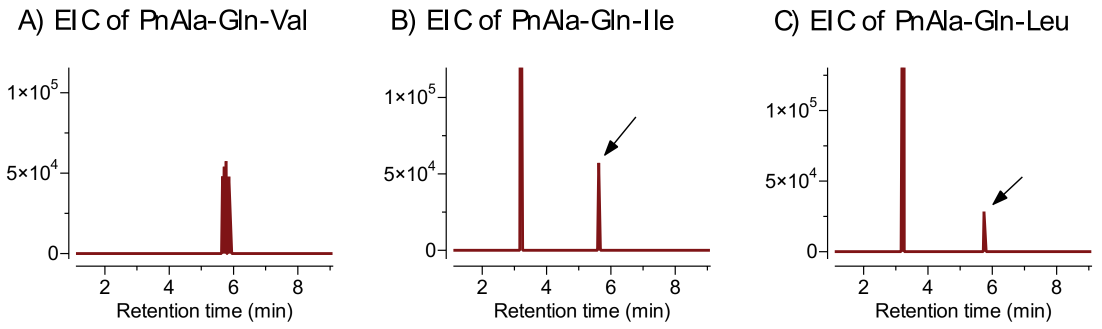

Figure S60. LC-HRMS detection of PnAla-Gln-X tripeptides synthesized in PnaBC reactions

A) PnAla-Gln-Val 397.1483 *m/z*, B) PnAla-Gln-Ile 411.1639 *m/z*, C) PnAla-Gln-Leu 411.1639 *m/z*

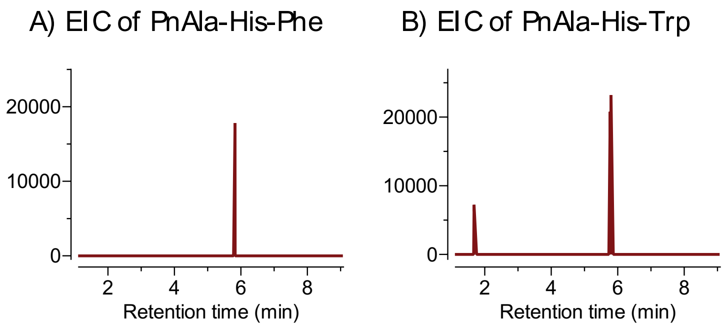

Figure S61. LC-HRMS detection of PnAla-His-X tripeptides synthesized in PnaBC reactions

A) PnAla-His-Phe 454.1486 *m/z*, B) PnAla-His-Trp 493.1595 *m/z*

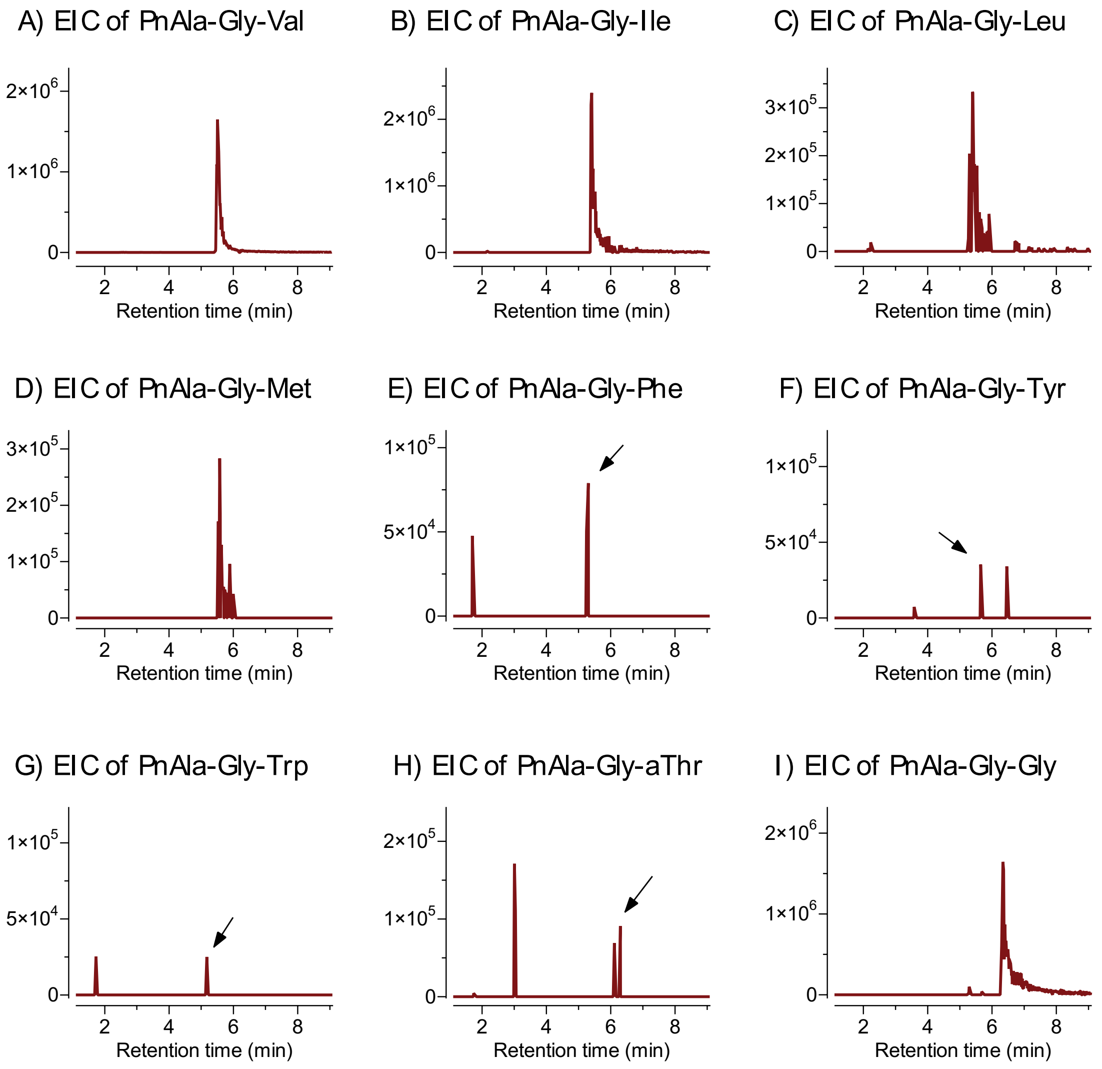

Figure S62. LC-HRMS detection of PnAla-Gly-X tripeptides synthesized in PnaBC reactions

A) PnAla-Gly-Val 326.1117 *m/z*, B) PnAla-Gly-Ile 340.1268 *m/z*, C) PnAla-Gly-Leu 340.1268 *m/z*, D) PnAla-Gly-Met 358.0832 *m/z*, E) PnAla-Gly-Phe 374.1112 *m/z*, F) PnAla-Gly-Tyr 390.1061 *m/z*, G) PnAla-Gly-Trp 413.1221 *m/z*, H) PnAla-Gly-aThr 328.0904 *m/z*, I) PnAla-Gly-Gly 284.0648 *m/z*

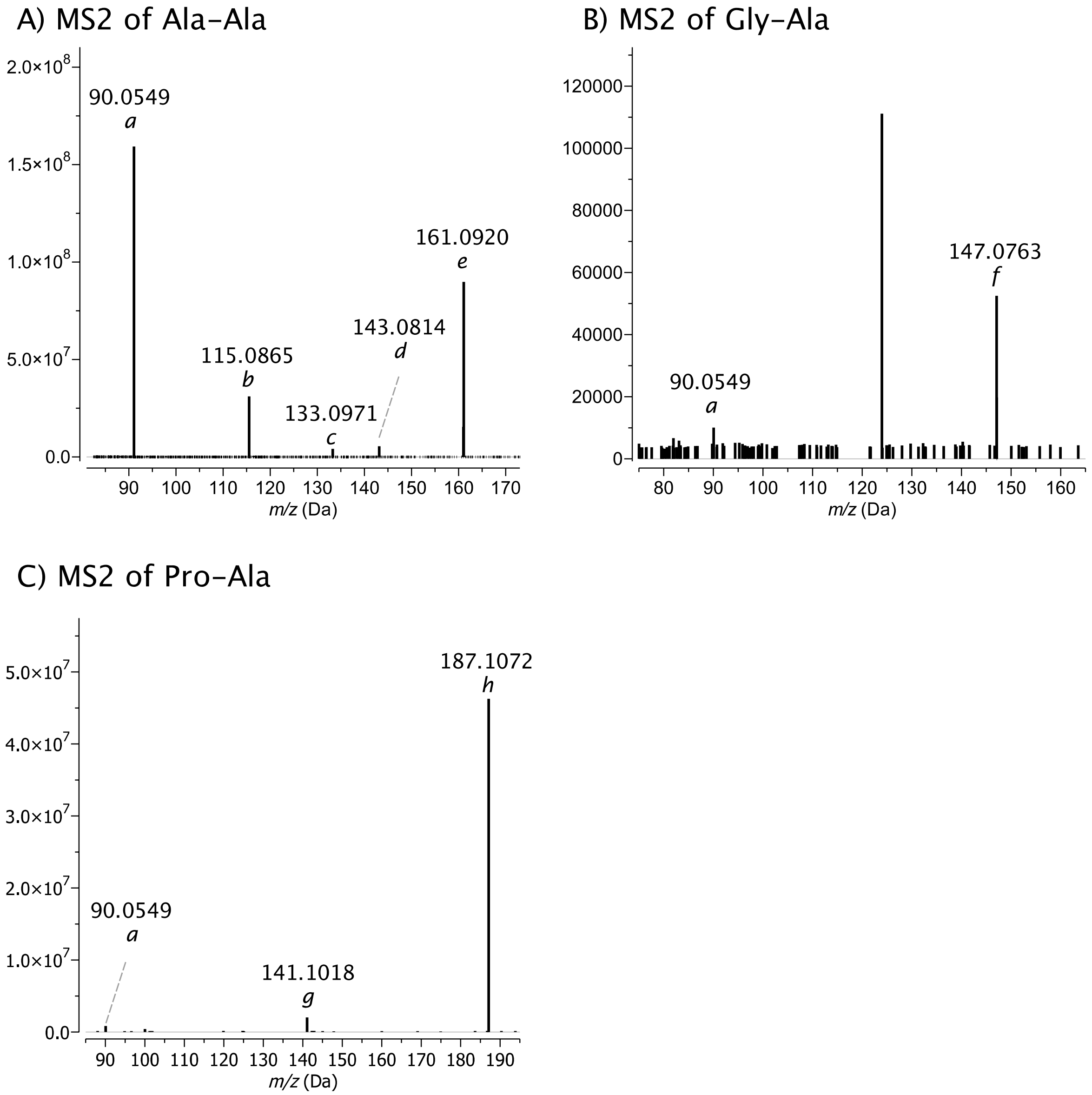

Figure S63. LC-MS/MS fragmentation of Ala-Ala, Gly-Ala, and Pro-Ala

Refer to Table S5 for fragment ion formula, structures, and mass error

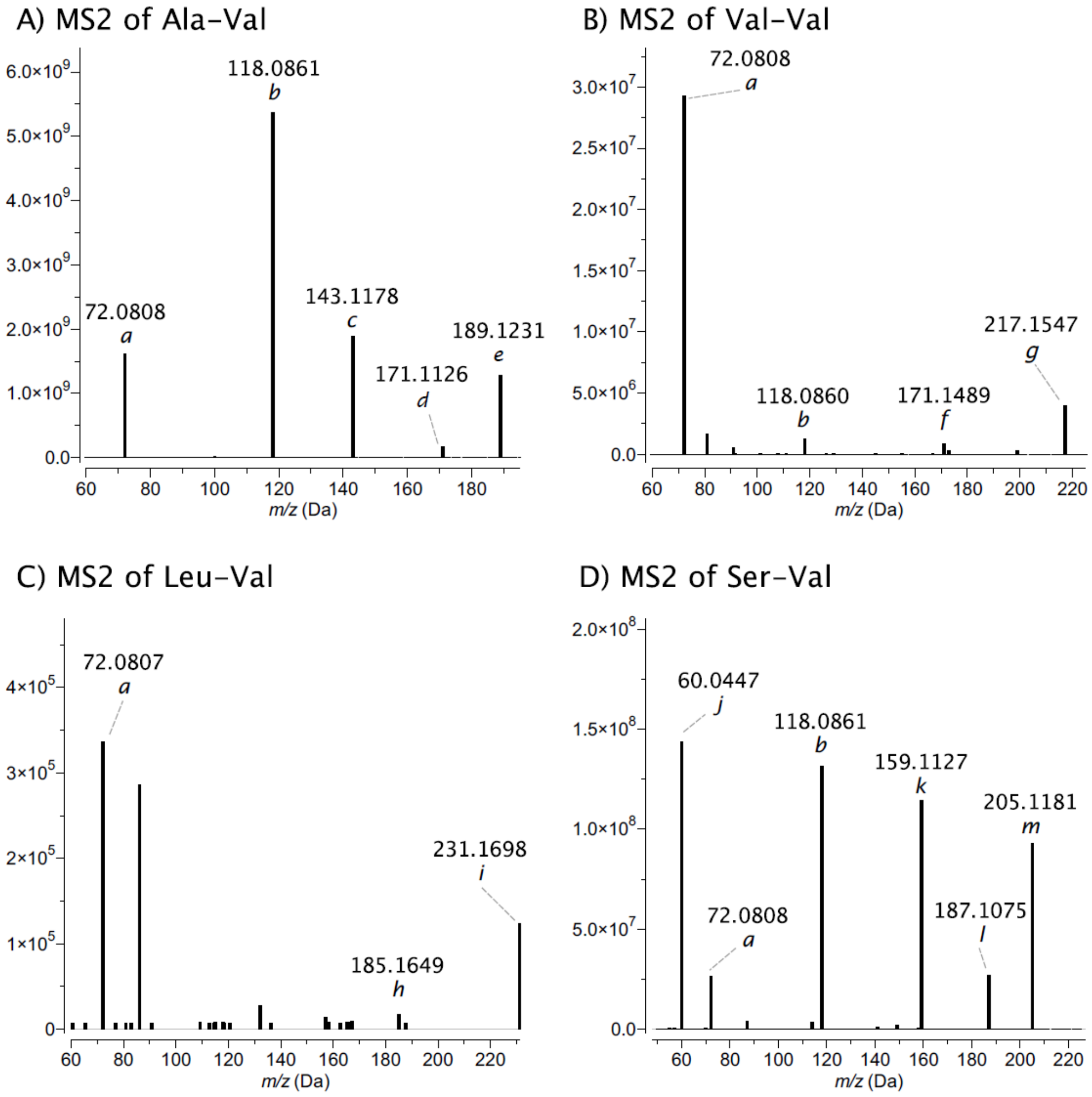

Figure S64. LC-MS/MS fragmentation of Ala-Val, Val-Val, Leu-Val, and Ser-Val

Refer to Table S6 for fragment ion formula, structures, and mass error

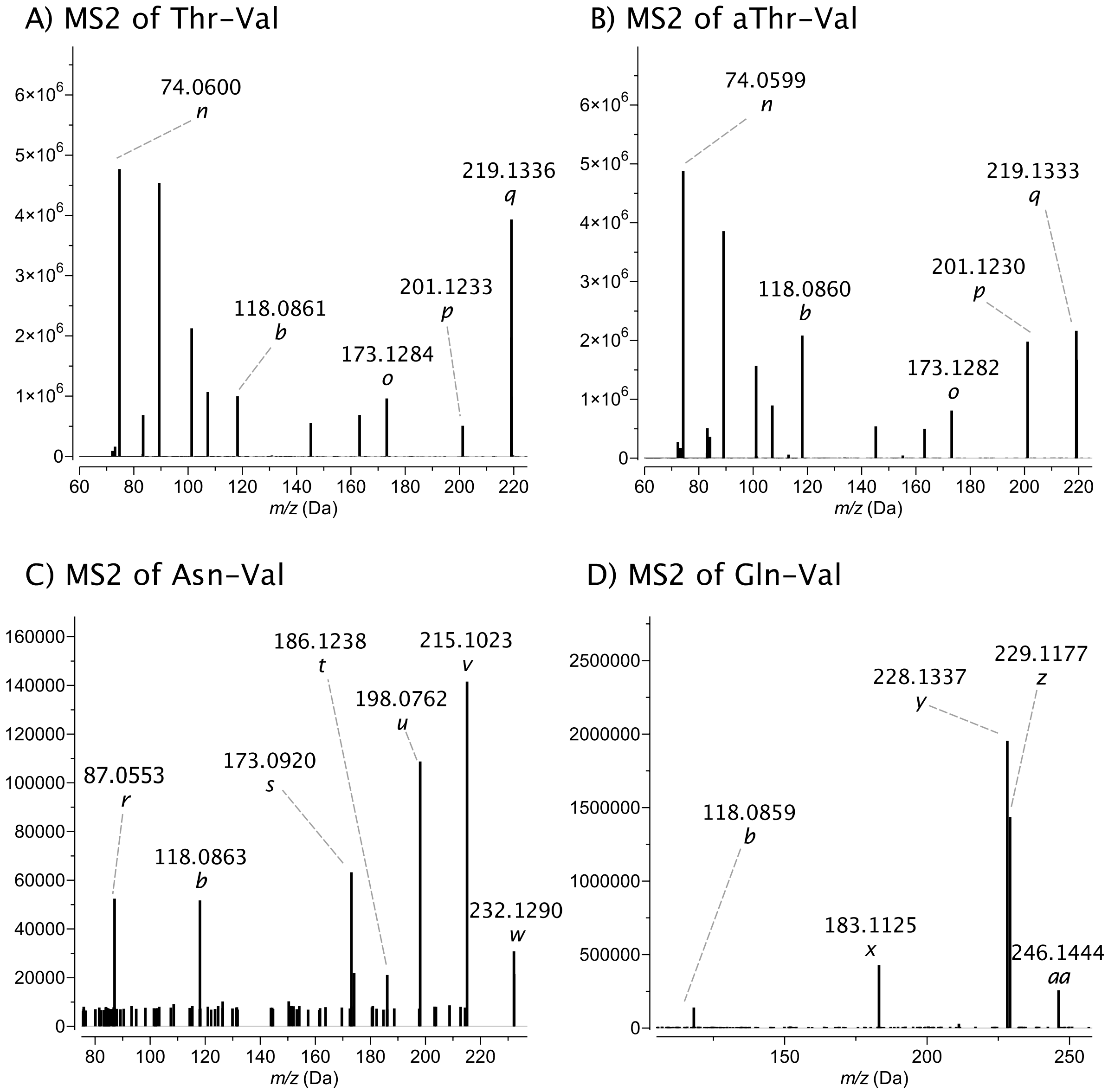

Figure S65. LC-MS/MS fragmentation of Thr-Val, aThr-Val, Asn-Val, and Gln-Val

Refer to Table S7 for fragment ion formula, structures, and mass error

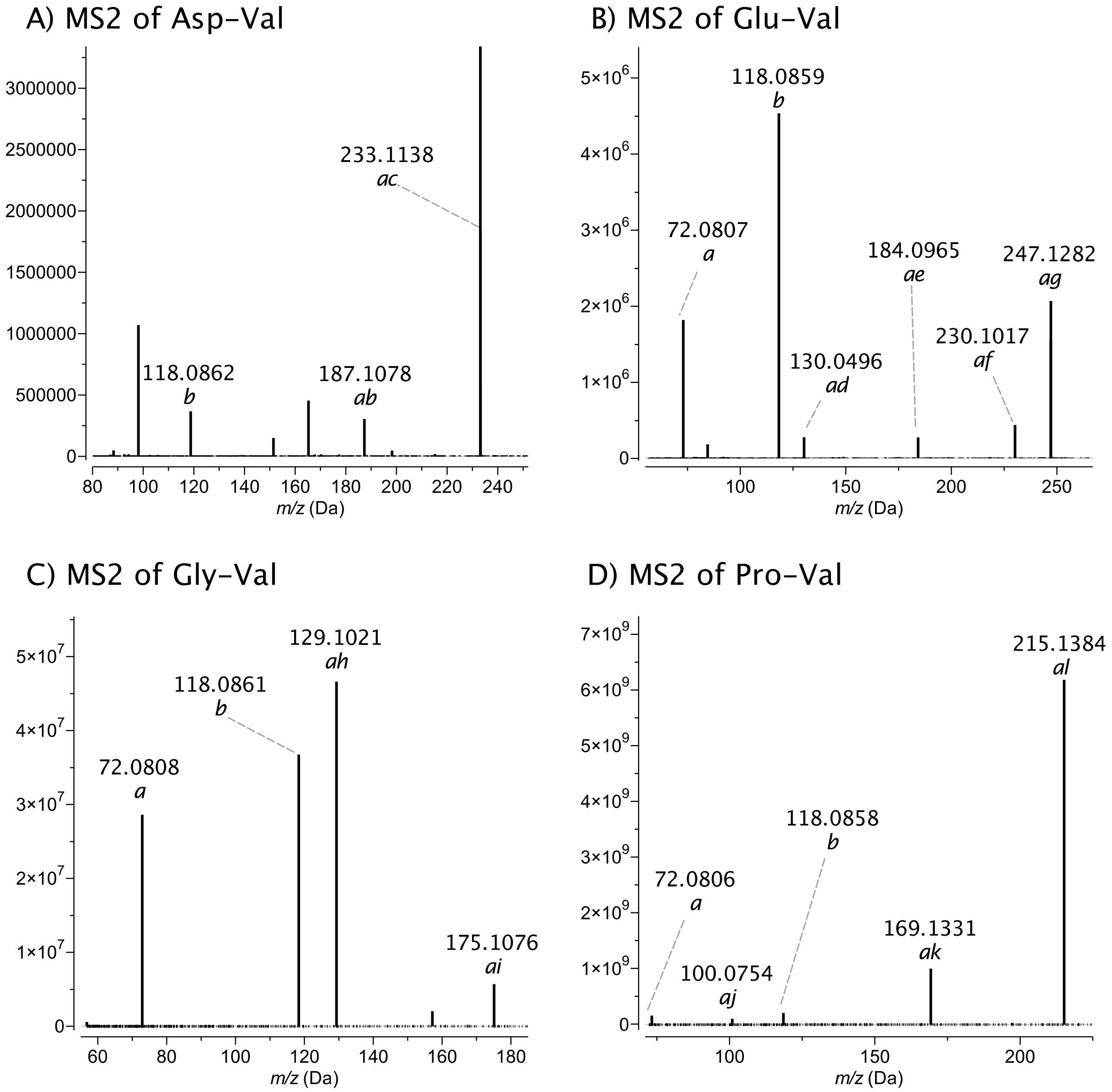

Figure S66. LC-MS/MS fragmentation of Asp-Val, Glu-Val, Gly-Val, and Pro-Val

Refer to Table S8 for fragment ion formula, structures, and mass error

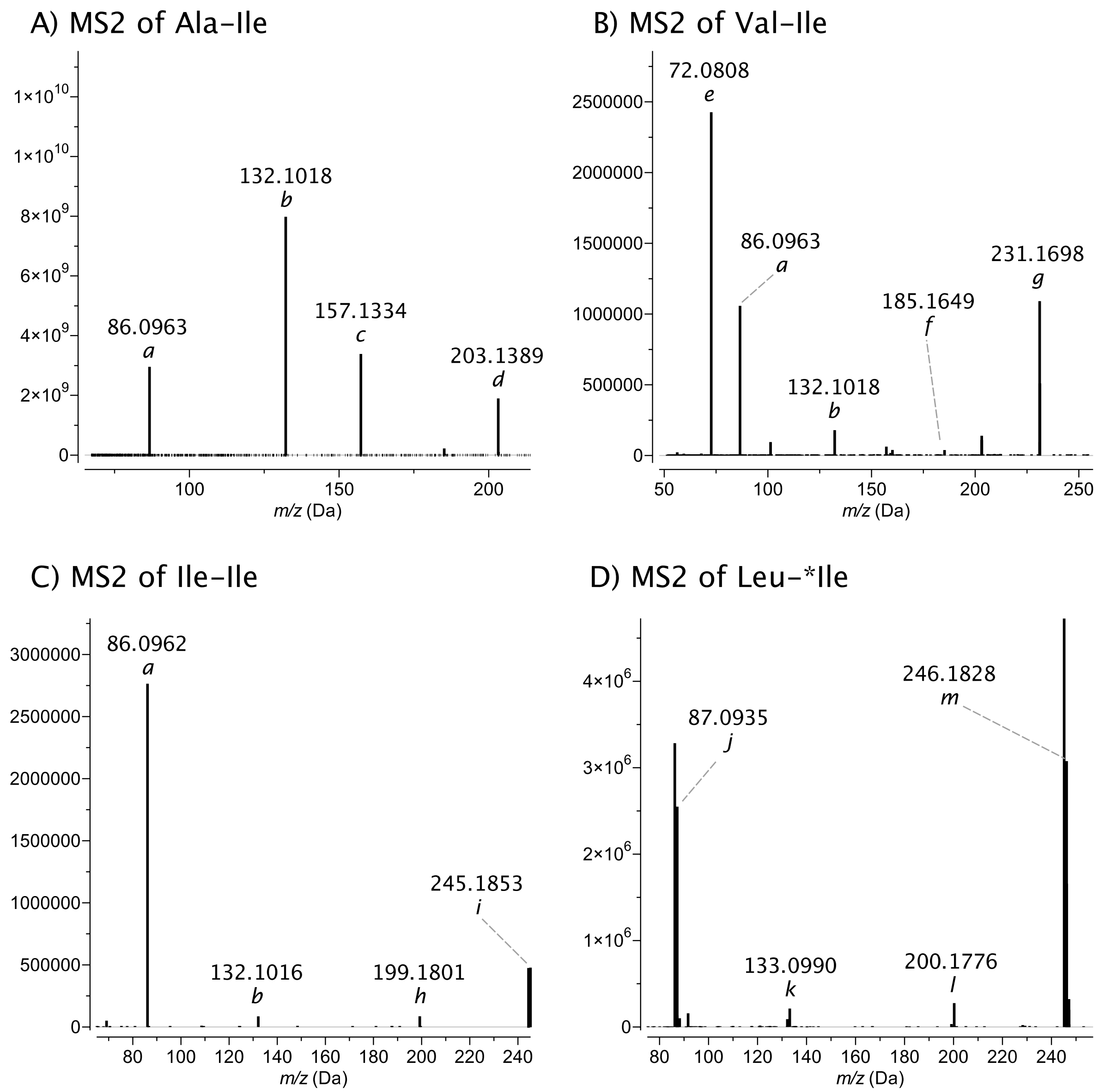

Figure S67. LC-MS/MS fragmentation of Ala-Ile, Val-Ile, Ile-Ile, and Leu-(^{15}N-Ile)

Refer to Table S9 for fragment ion formula, structures, and mass error

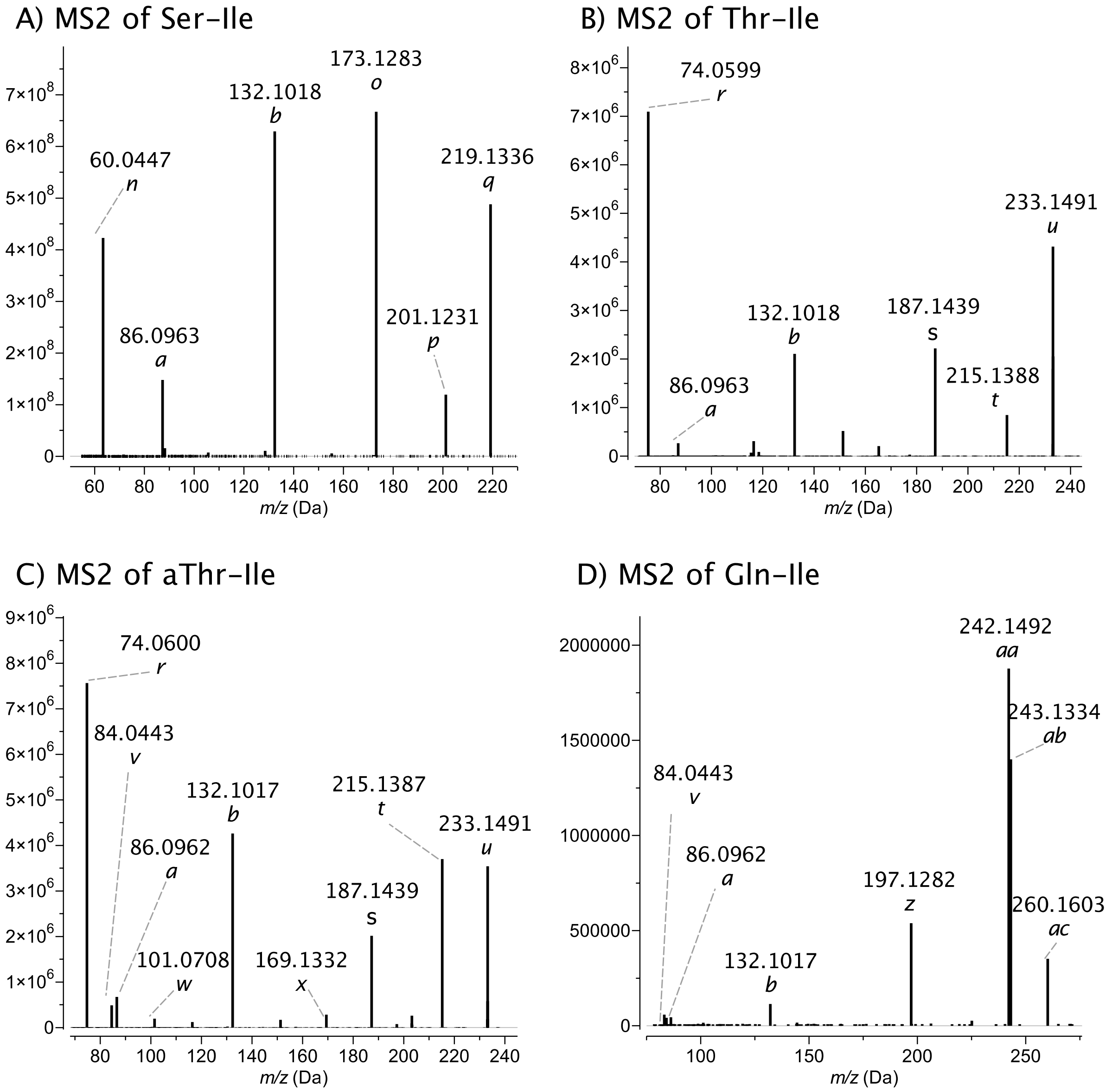

Figure S68. LC-MS/MS fragmentation of Ser-Ile, Thr-Ile, aThr-Ile, and Gln-Ile

Refer to Table S10 for fragment ion formula, structures, and mass error

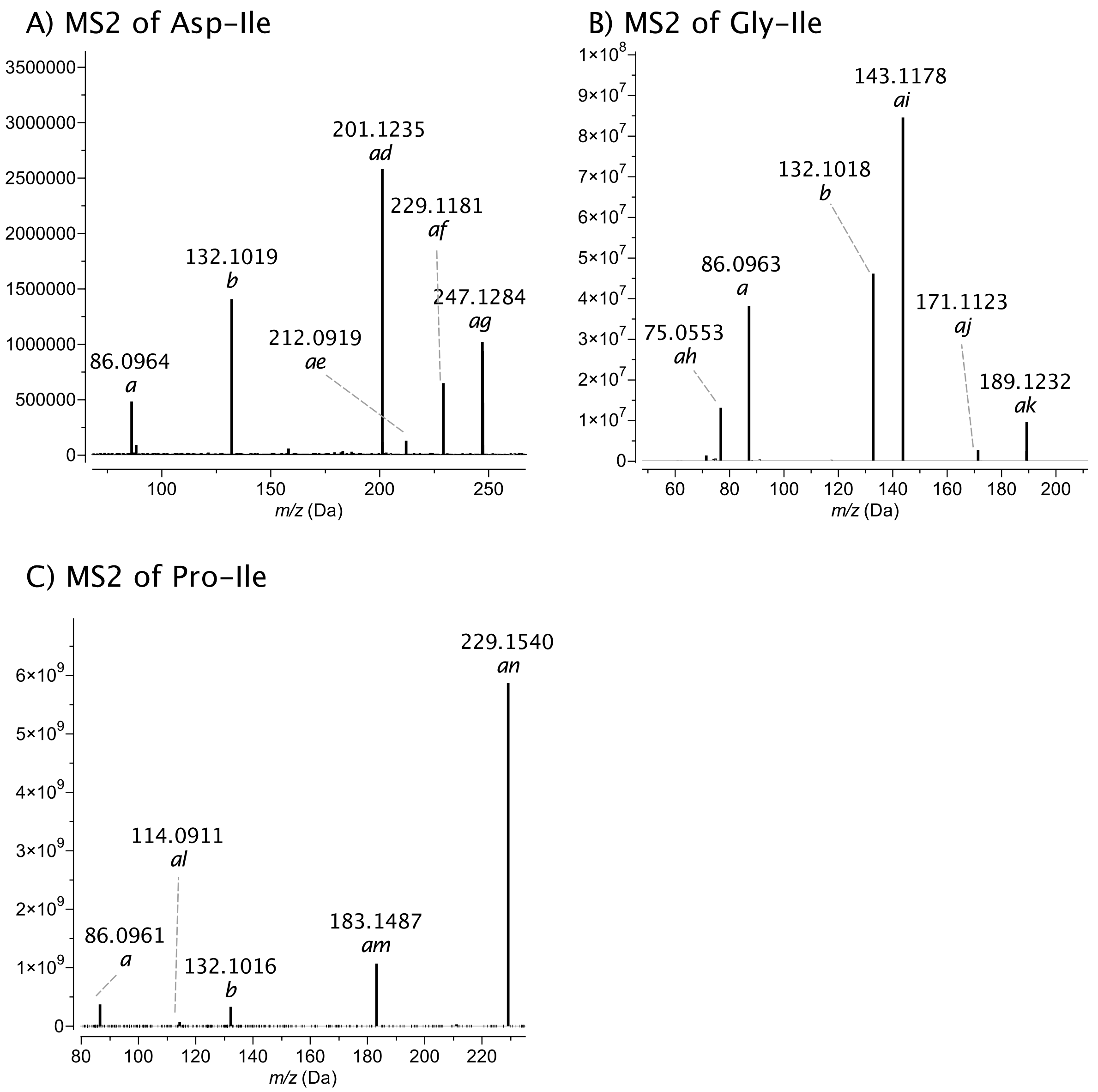

Figure S69. LC-MS/MS fragmentation of Asp-Ile, Gly-Ile, and Pro-Ile

Refer to Table S11 for fragment ion formula, structures, and mass error

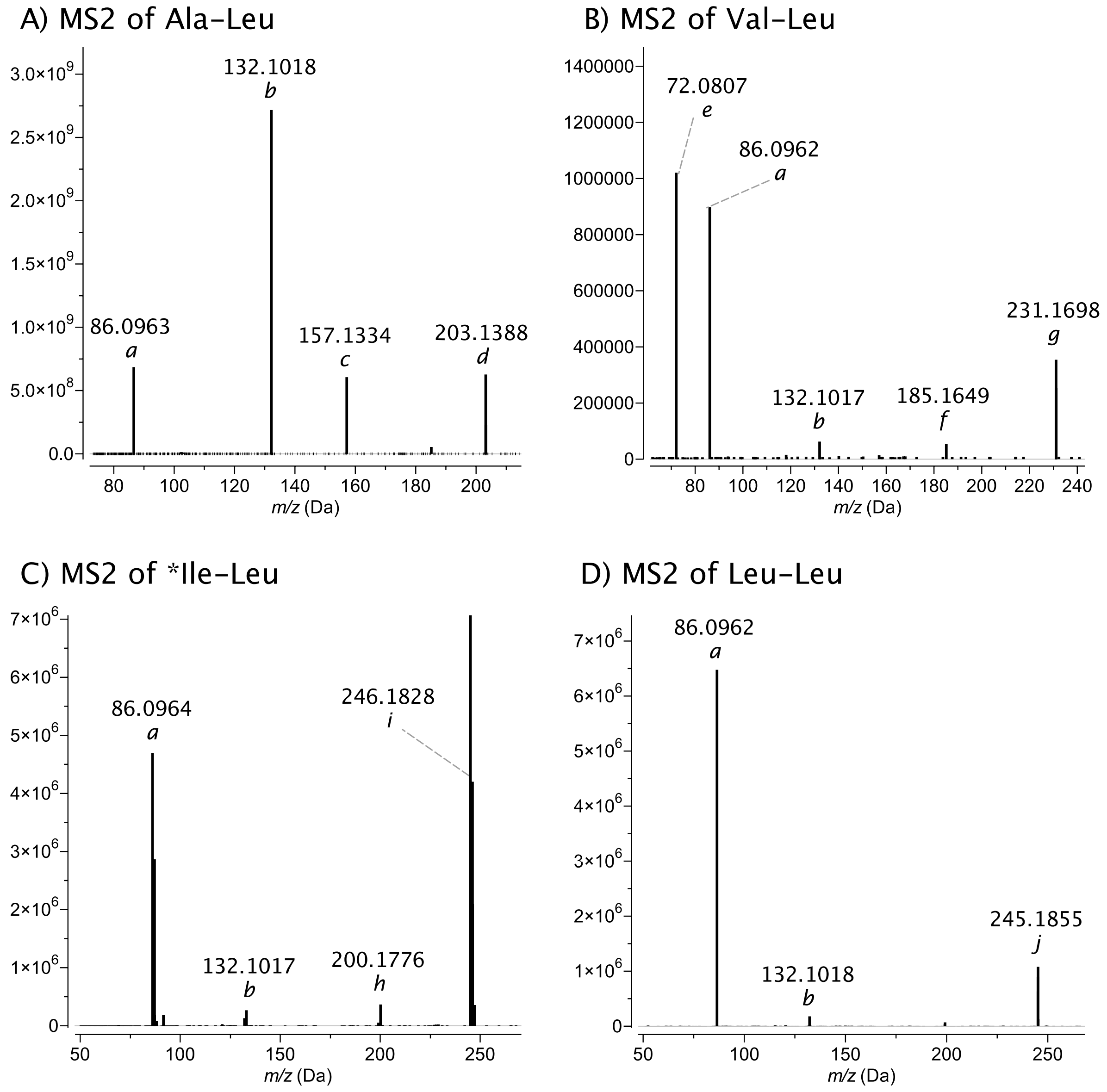

Figure S70. LC-MS/MS fragmentation of Ala-Leu, Val-Leu, (^{15}N-Ile)-Leu, and Leu-Leu

Refer to Table S12 for fragment ion formula, structures, and mass error

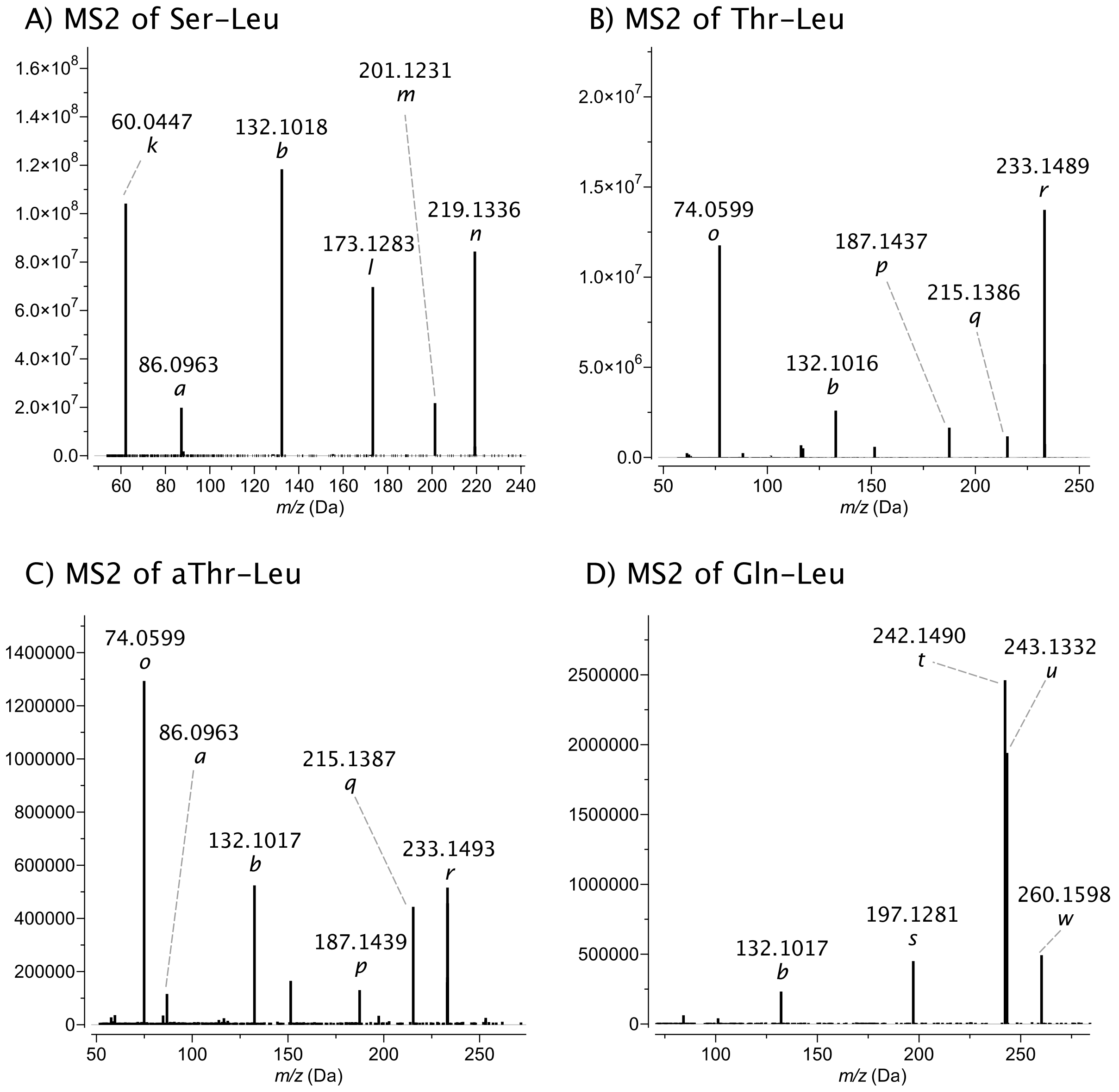

Figure S71. LC-MS/MS fragmentation of Ser-Leu, Thr-Leu, aTHr-Leu, and Gln-Leu

Refer to Table S13 for fragment ion formula, structures, and mass error

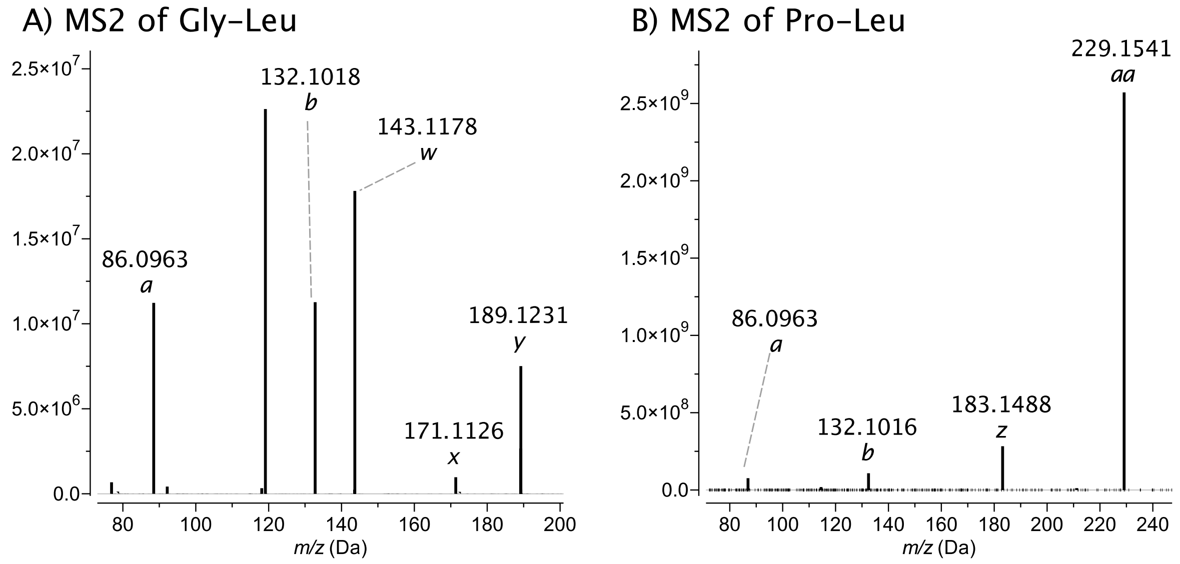

Figure S72. LC-MS/MS fragmentation of Gly-Leu and Pro-Leu

Refer to Table S14 for fragment ion formula, structures, and mass error

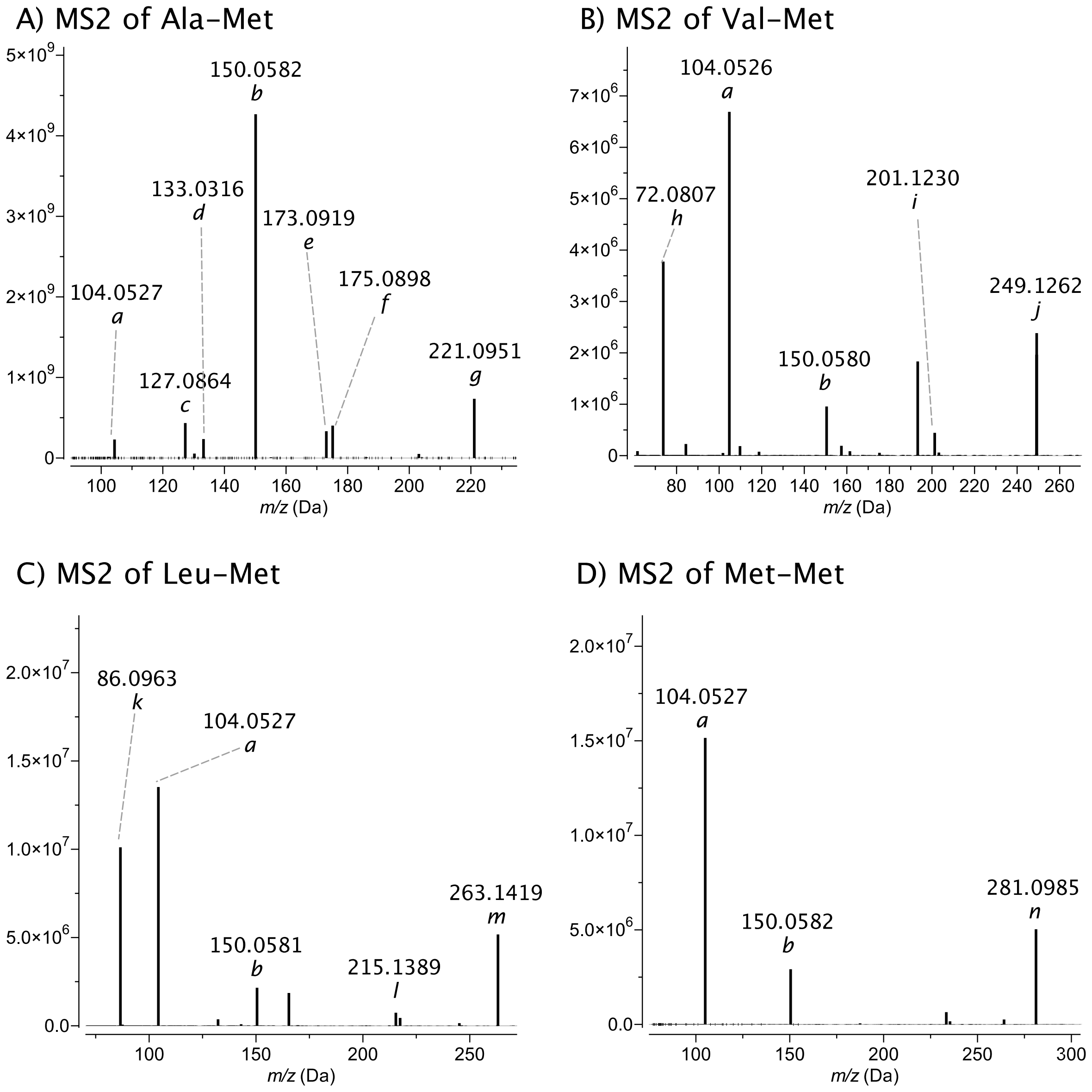

Figure S73. LC-MS/MS fragmentation of Ala-Met, Val-Met, Leu-Met, and Met-Met

Refer to Table S15 for fragment ion formula, structures, and mass error

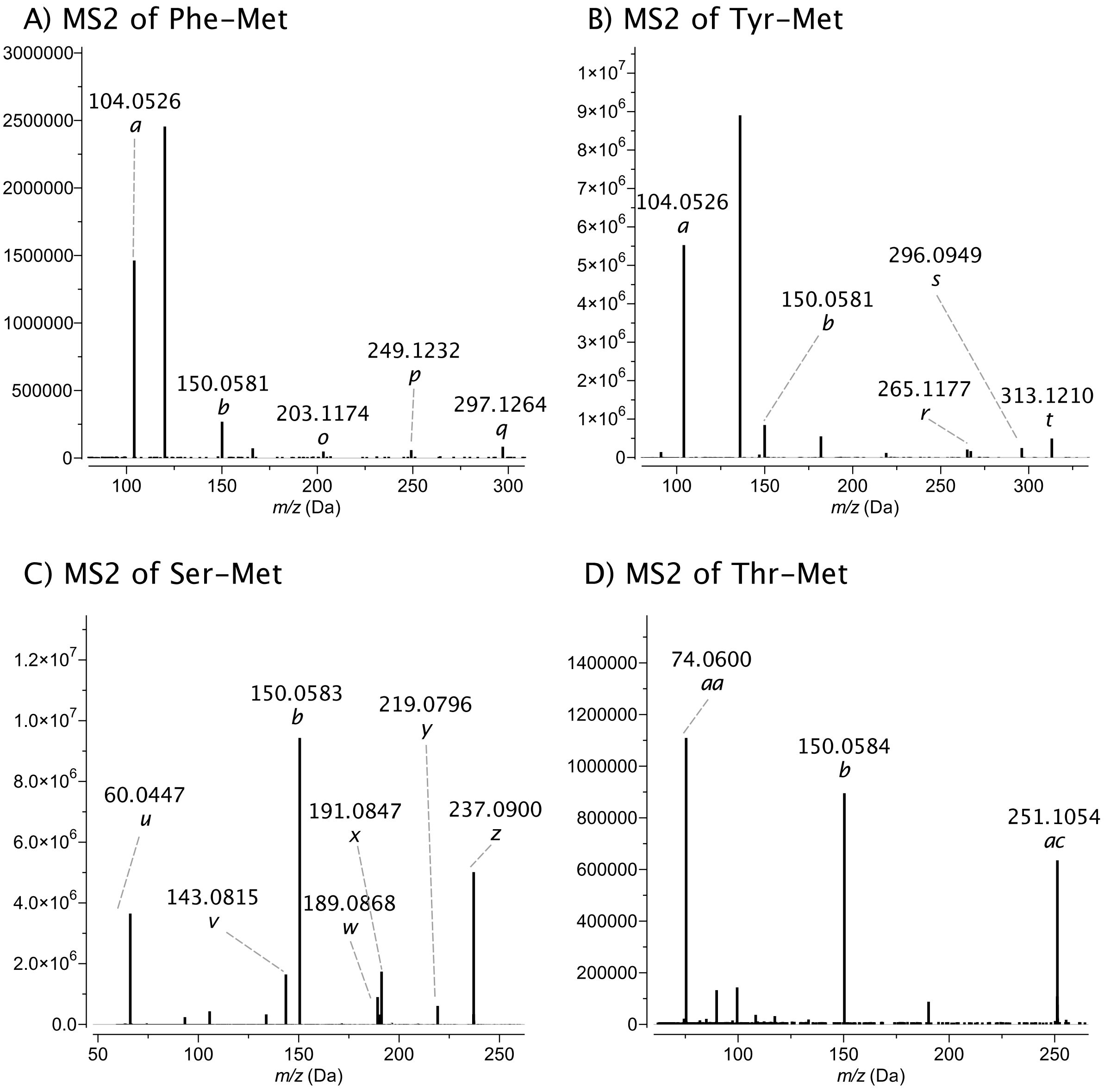

Figure S74. LC-MS/MS fragmentation of Phe-Met, Tyr-Met, Ser-Met, and Thr-Met

Refer to Table S16 for fragment ion formula, structures, and mass error

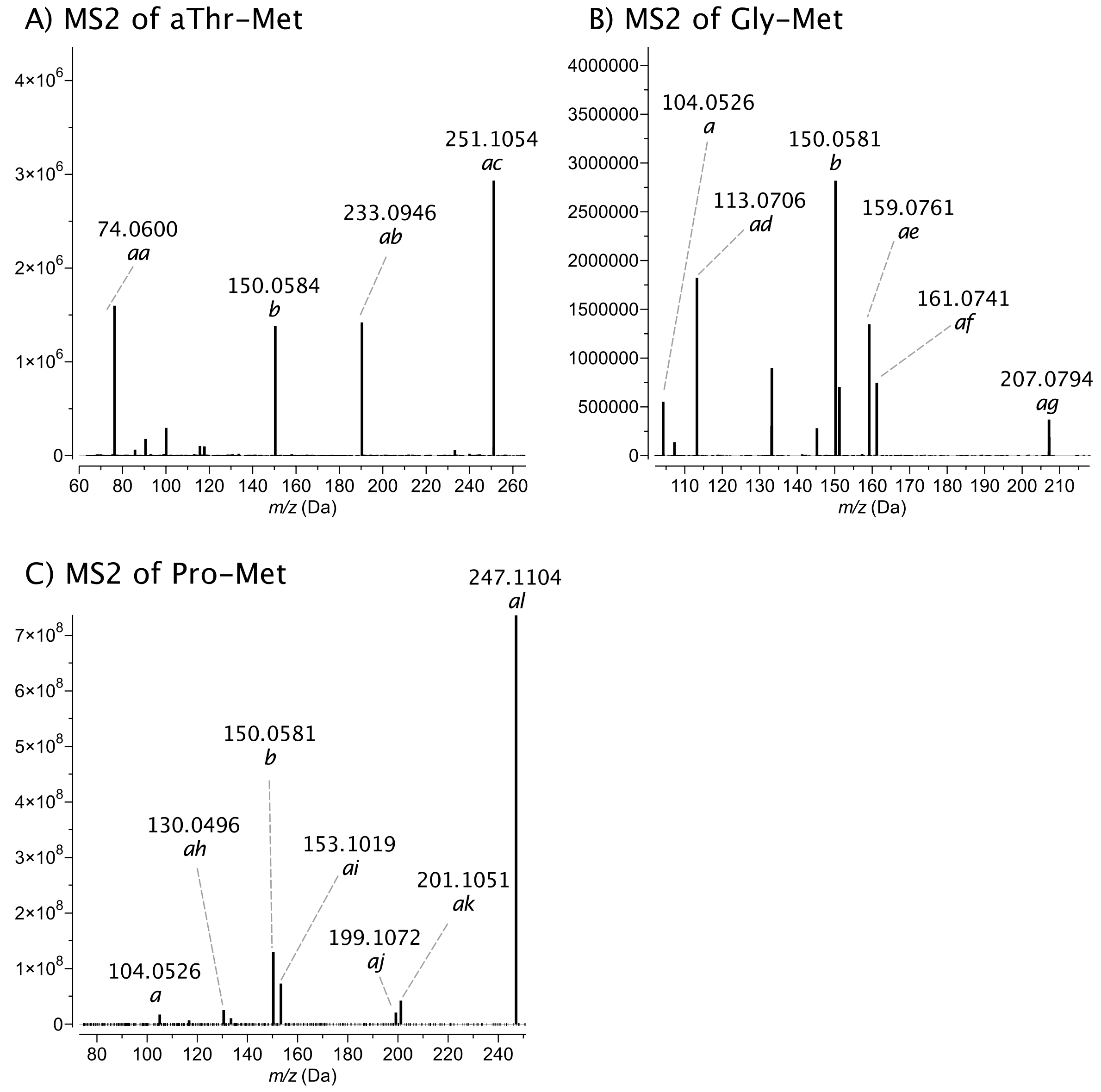

Figure S75. LC-MS/MS fragmentation of aThr-Met, Gly-Met, and Pro-Met

Refer to Table S17 for fragment ion formula, structures, and mass error

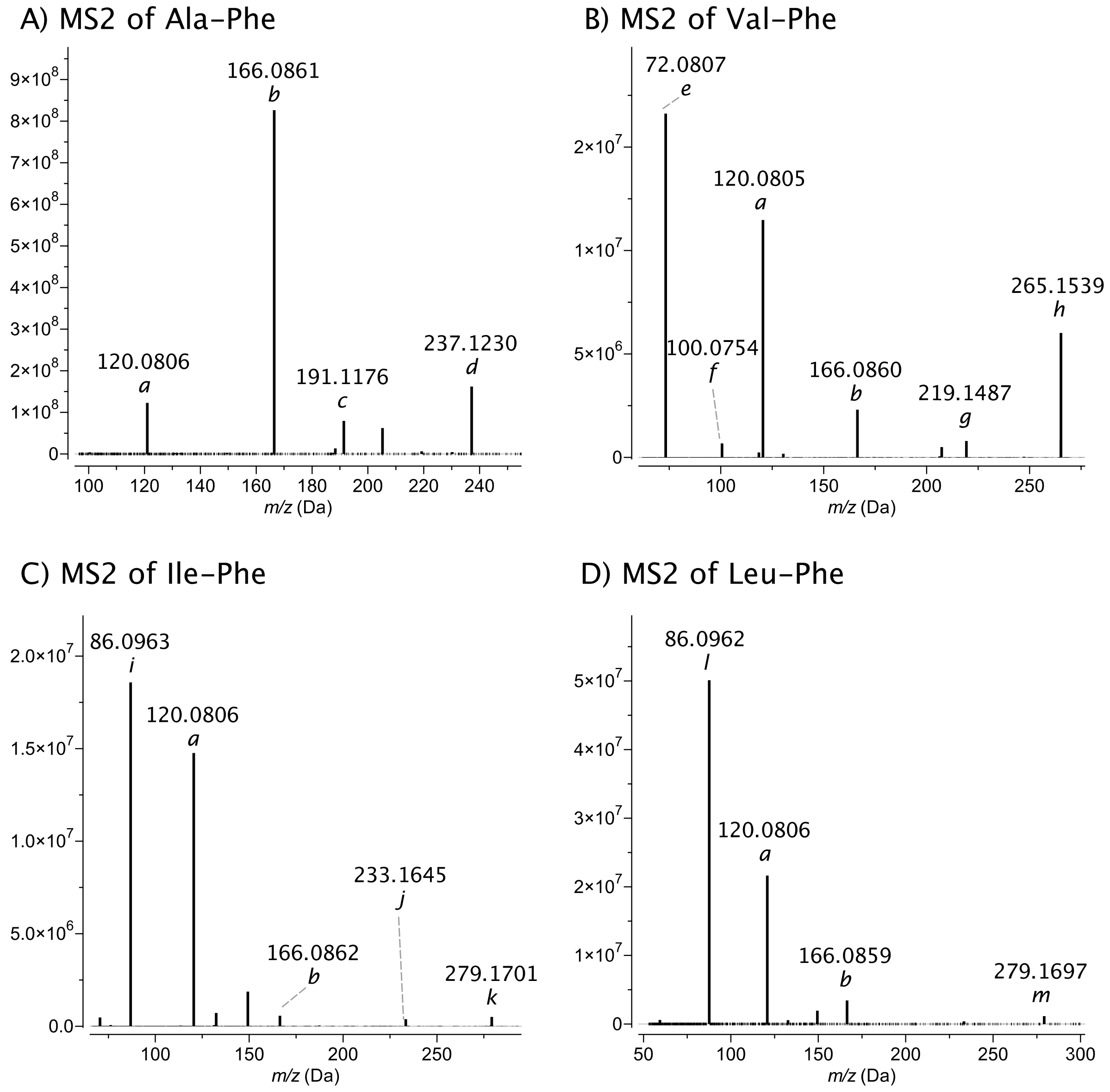

Figure S76. LC-MS/MS fragmentation of Ala-Phe, Val-Phe, Ile-Phe, and Leu-Phe

Refer to Table S18 for fragment ion formula, structures, and mass error

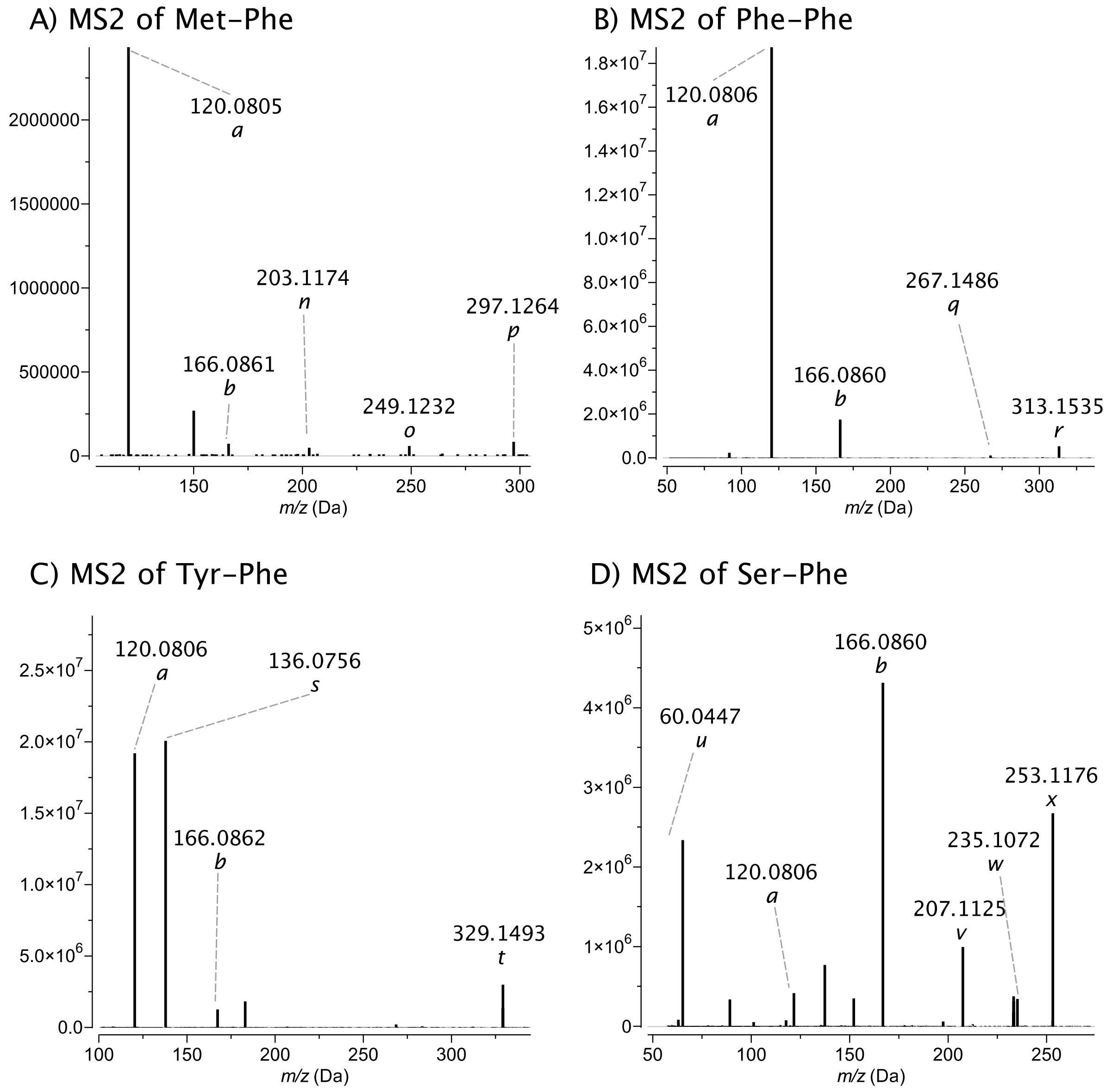

Figure S77. LC-MS/MS fragmentation of Met-Phe, Phe-Phe, Tyr-Phe, and Ser-Phe

Refer to Table S19 for fragment ion formula, structures, and mass error

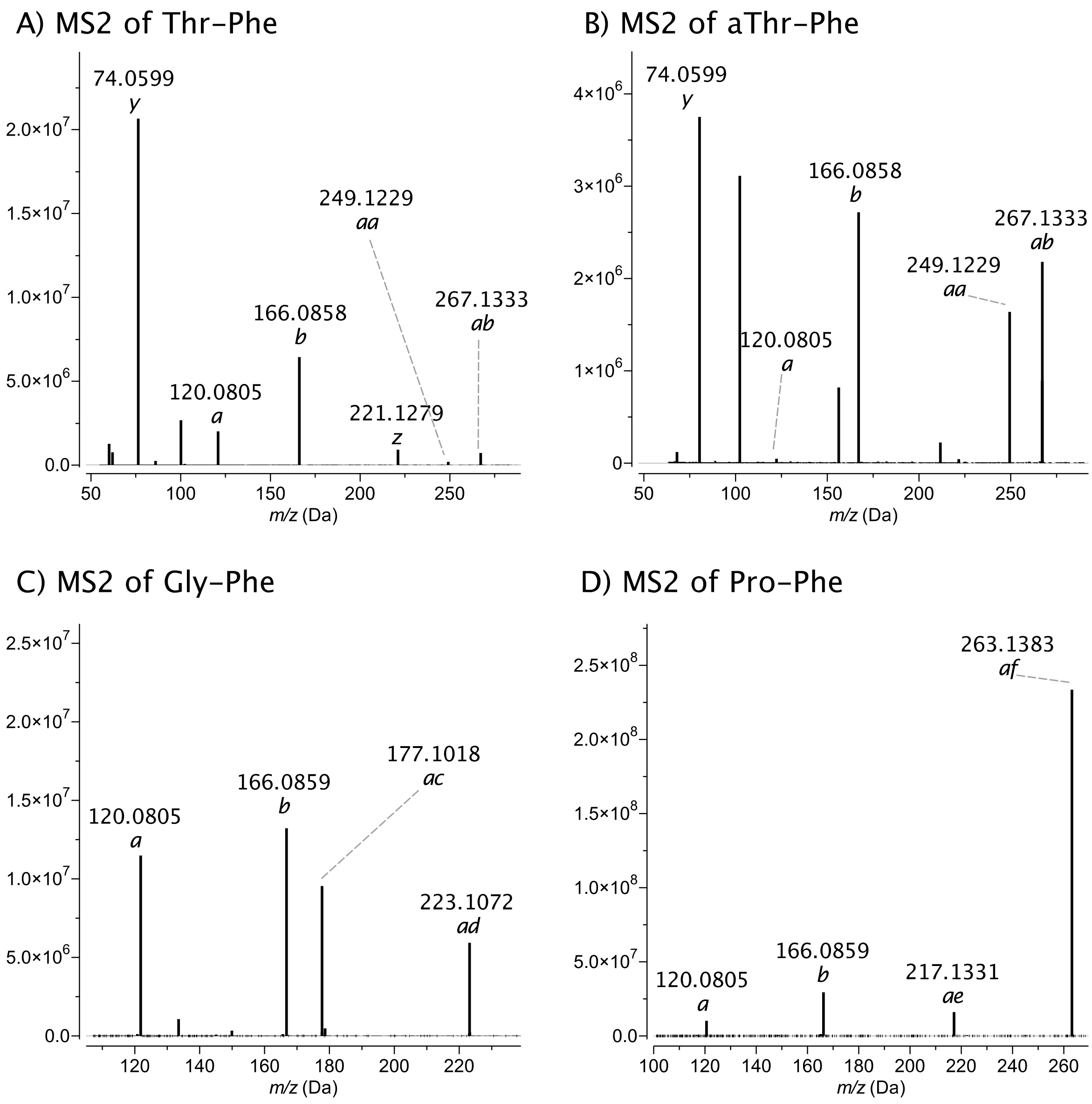

Figure S78. LC-MS/MS fragmentation of Thr-Phe, aThr-Phe, Gly-Phe, and Pro-Phe

Refer to Table S20 for fragment ion formula, structures, and mass error

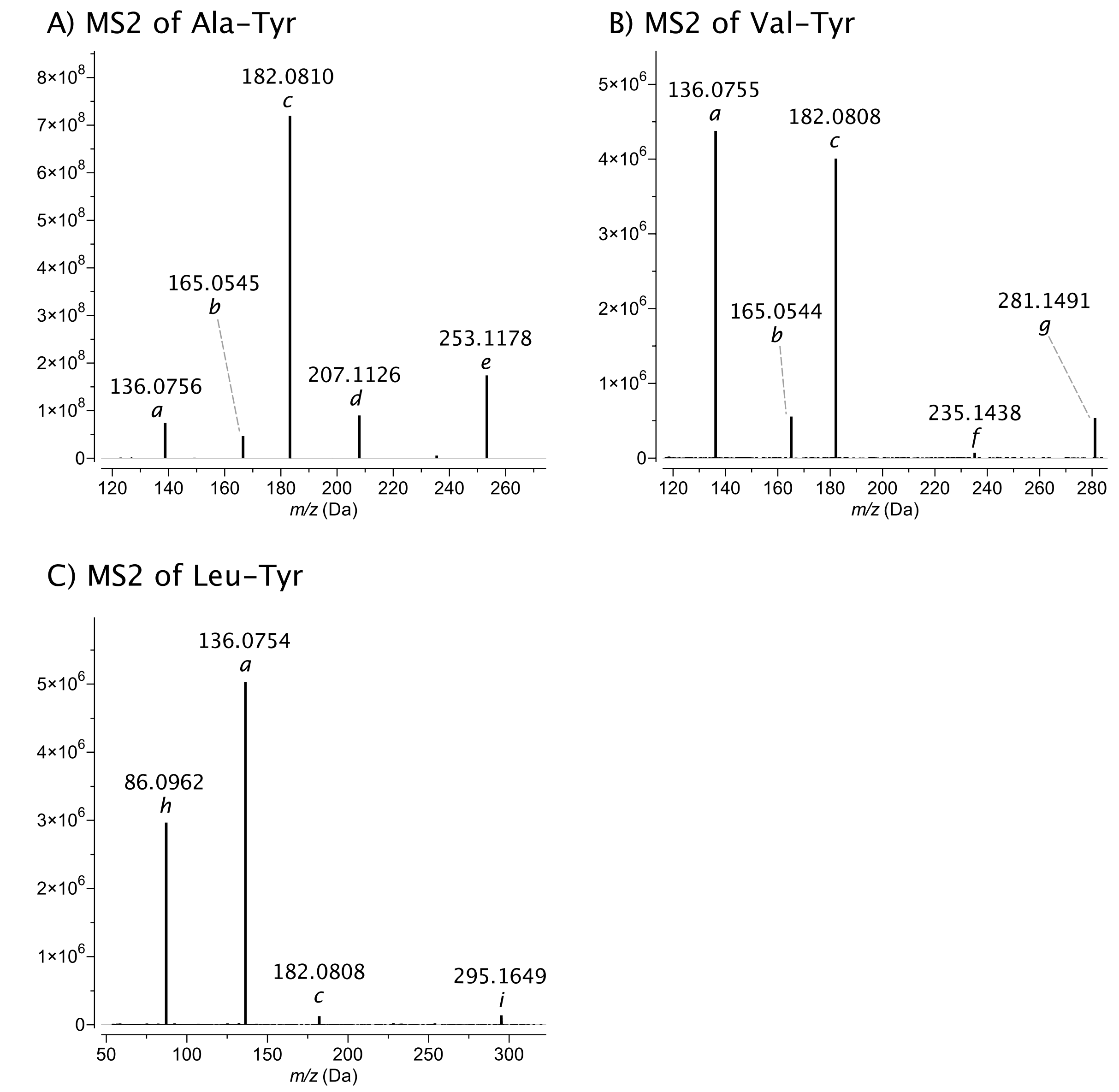

Figure S79. LC-MS/MS fragmentation of Ala-Tyr, Val-Tyr, and Leu-Tyr
Refer to Table S21 for fragment ion formula, structures, and mass error

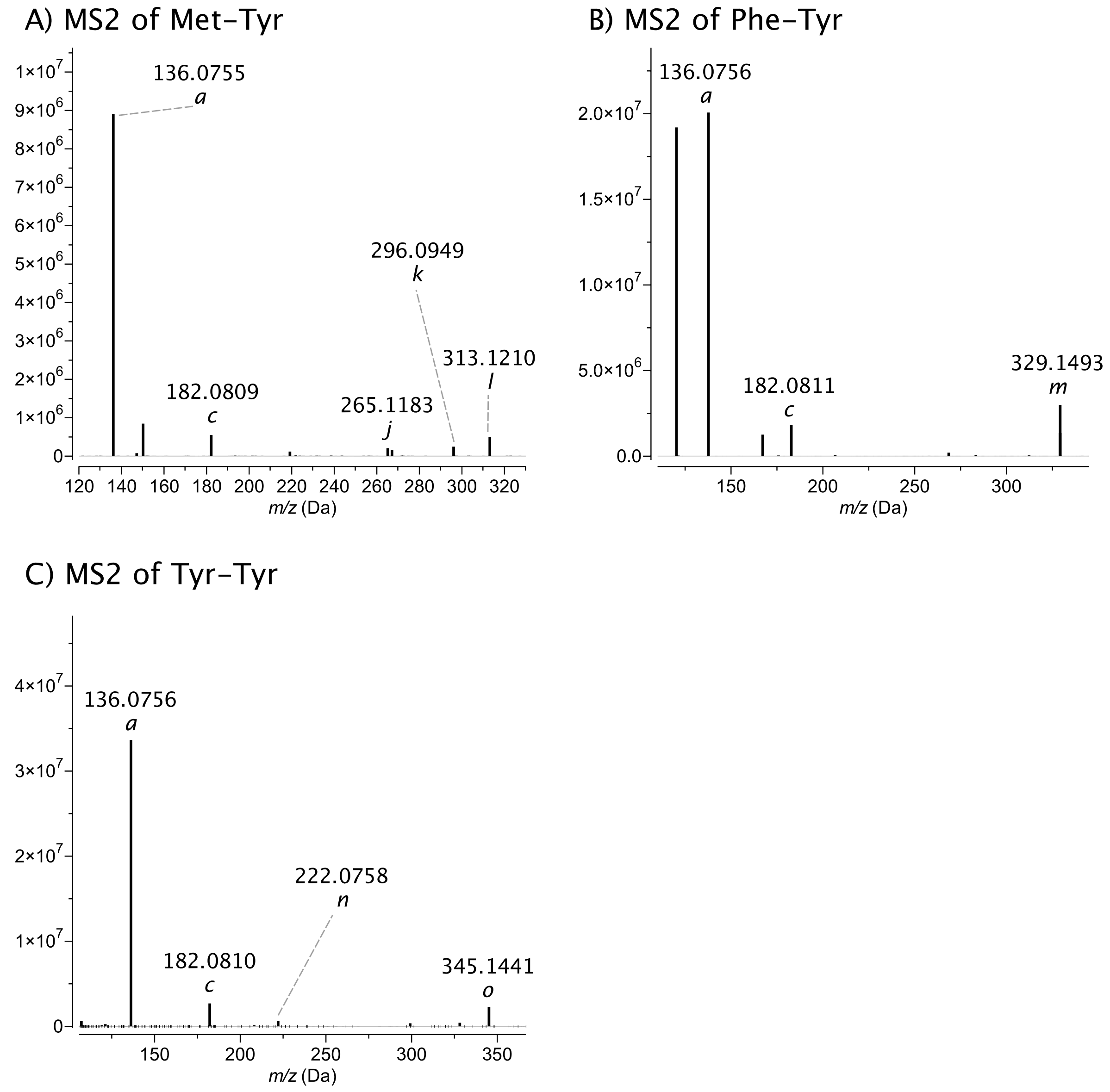

Figure S80. LC-MS/MS fragmentation of Met-Tyr, Phe-Tyr, and Tyr-Tyr

Refer to Table S22 for fragment ion formula, structures, and mass error

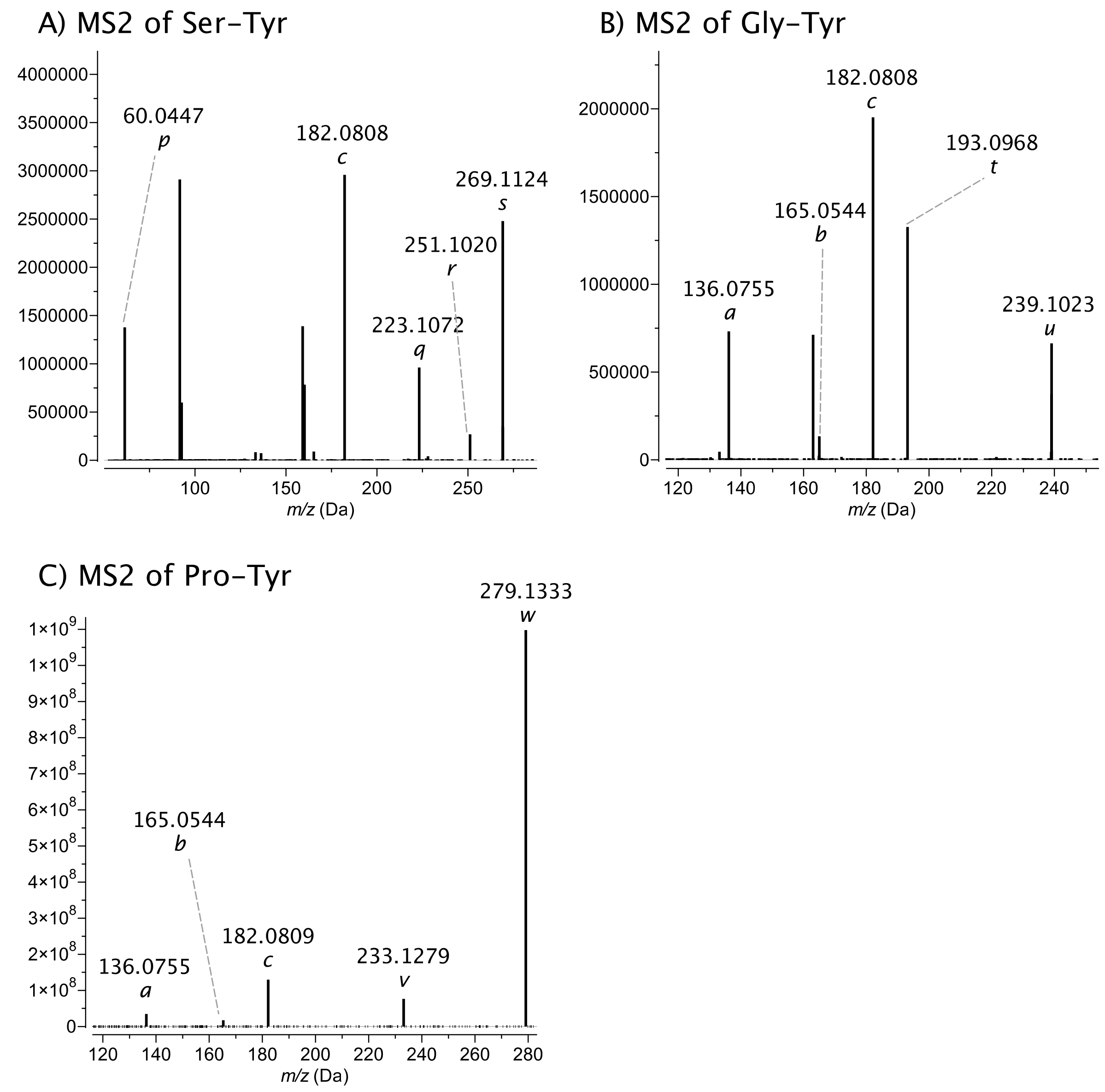

Figure S81. LC-MS/MS fragmentation of Ser-Tyr, Gly-Tyr, and Pro-Tyr

Refer to Table S23 for fragment ion formula, structures, and mass error

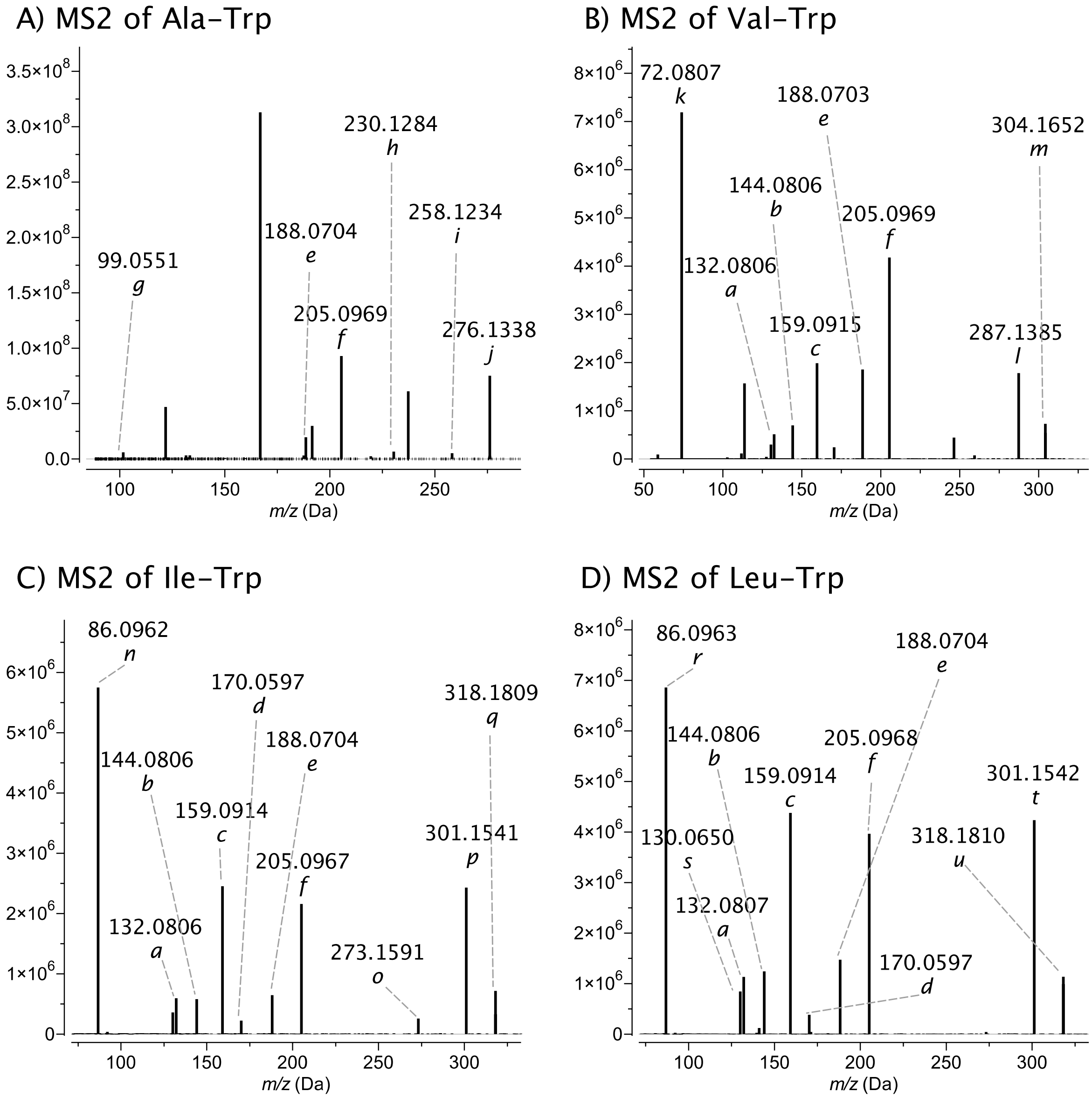

Figure S82. LC-MS/MS fragmentation of Ala-Trp, Val-Trp, Ile-Trp, and Leu-Trp

Refer to Table S24 for fragment ion formula, structures, and mass error

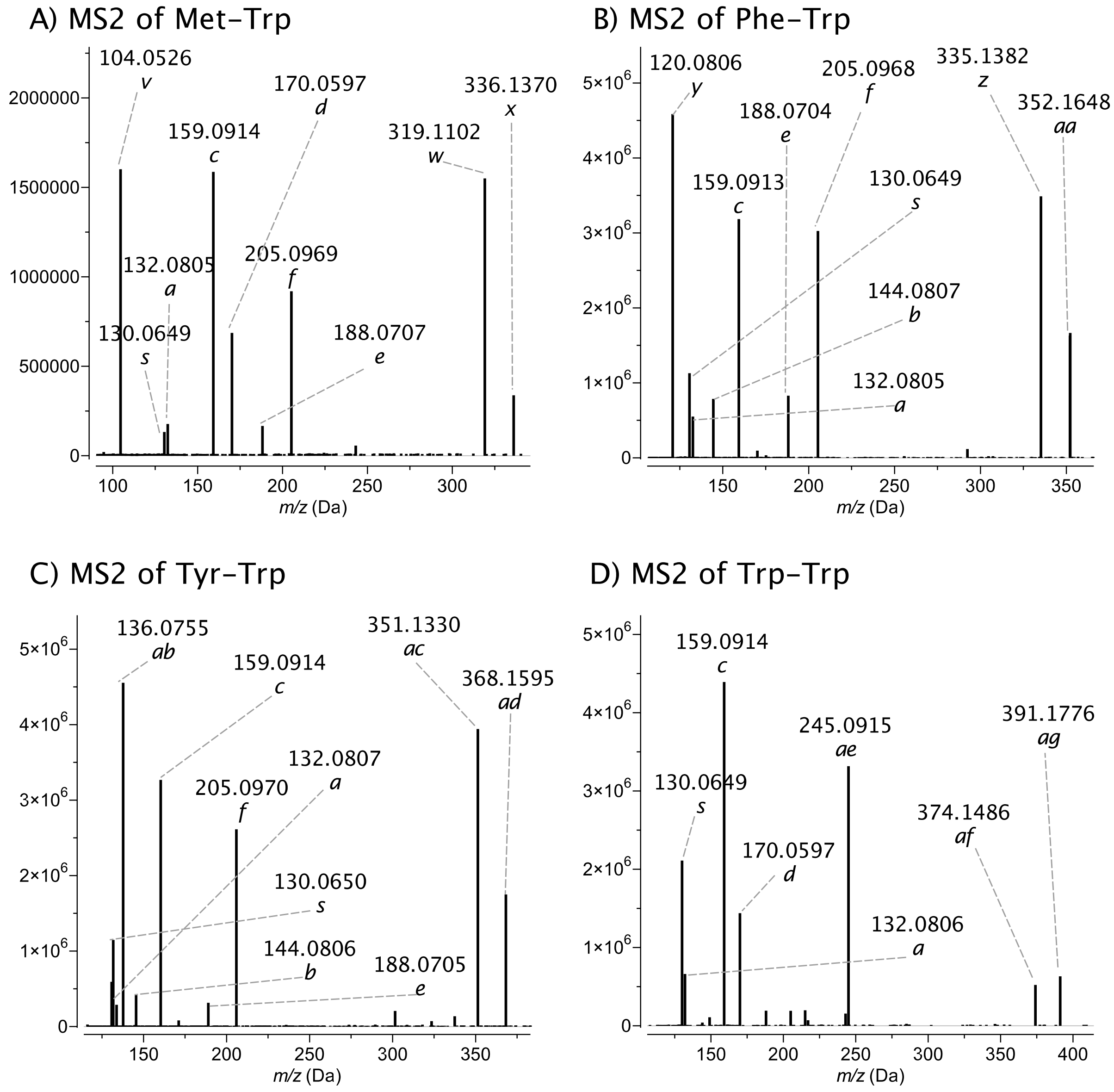

Figure S83. LC-MS/MS fragmentation of Met-Trp, Phe-Trp, Tyr-Trp, and Trp-Trp
Refer to Table S25 for fragment ion formula, structures, and mass error

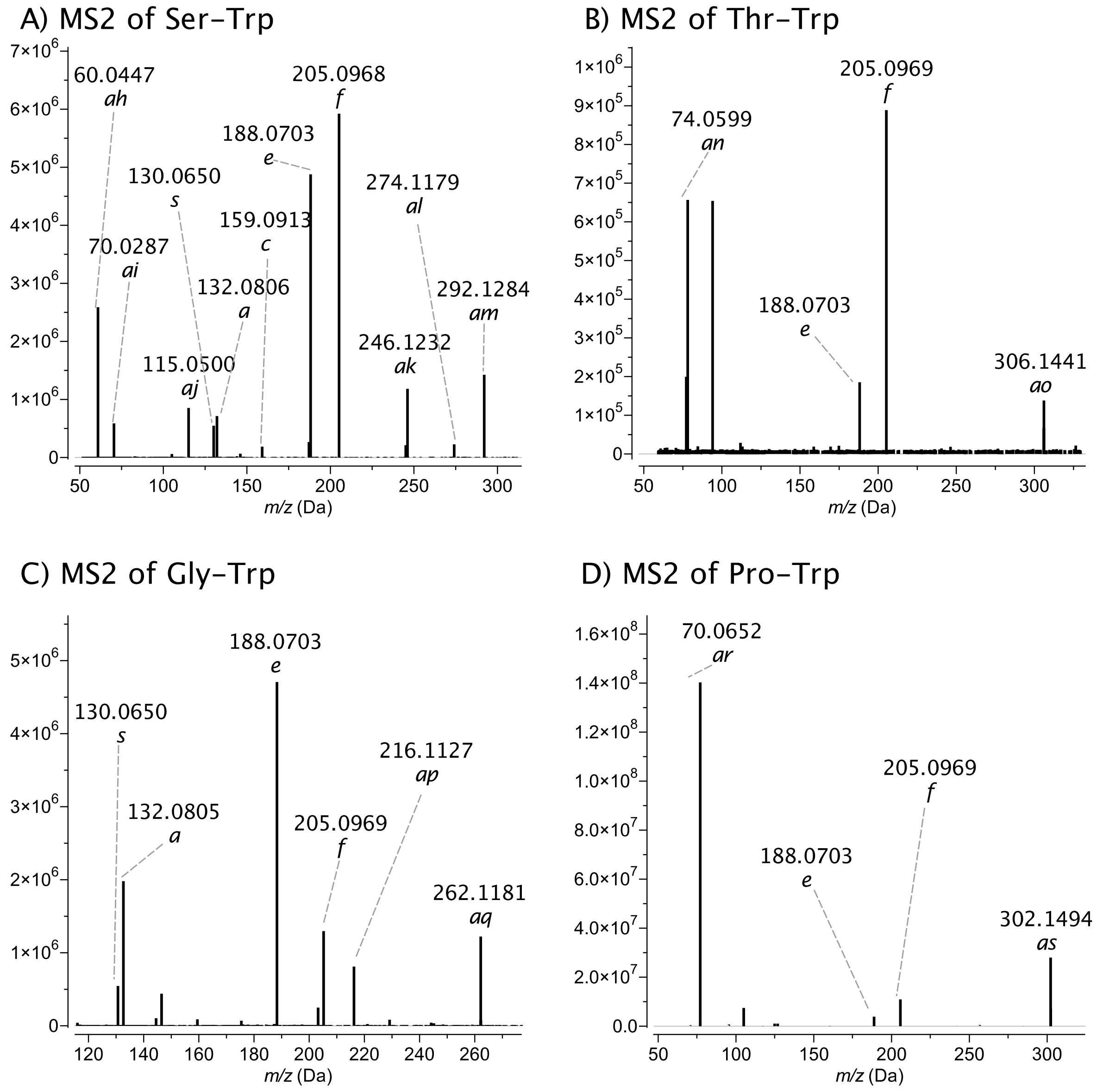

Figure S84. LC-MS/MS fragmentation of Ser-Trp, Thr-Trp, Gly-Trp, and Pro-Trp

Refer to Table S26 for fragment ion formula, structures, and mass error

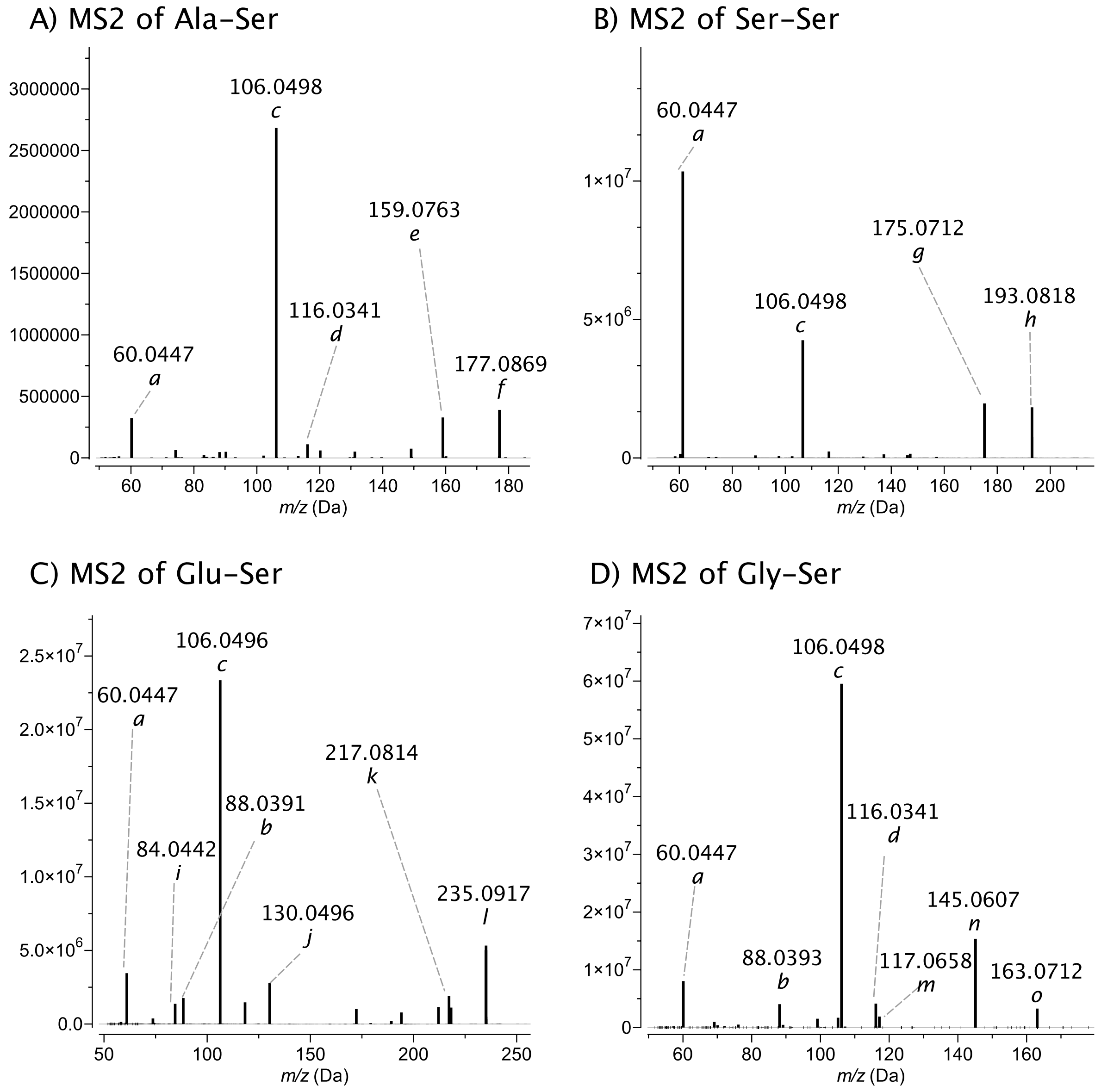

Figure S85. LC-MS/MS fragmentation of Ala-Ser, Ser-Ser, Glu-Ser, and Gly-Ser

Refer to Table S27 for fragment ion formula, structures, and mass error

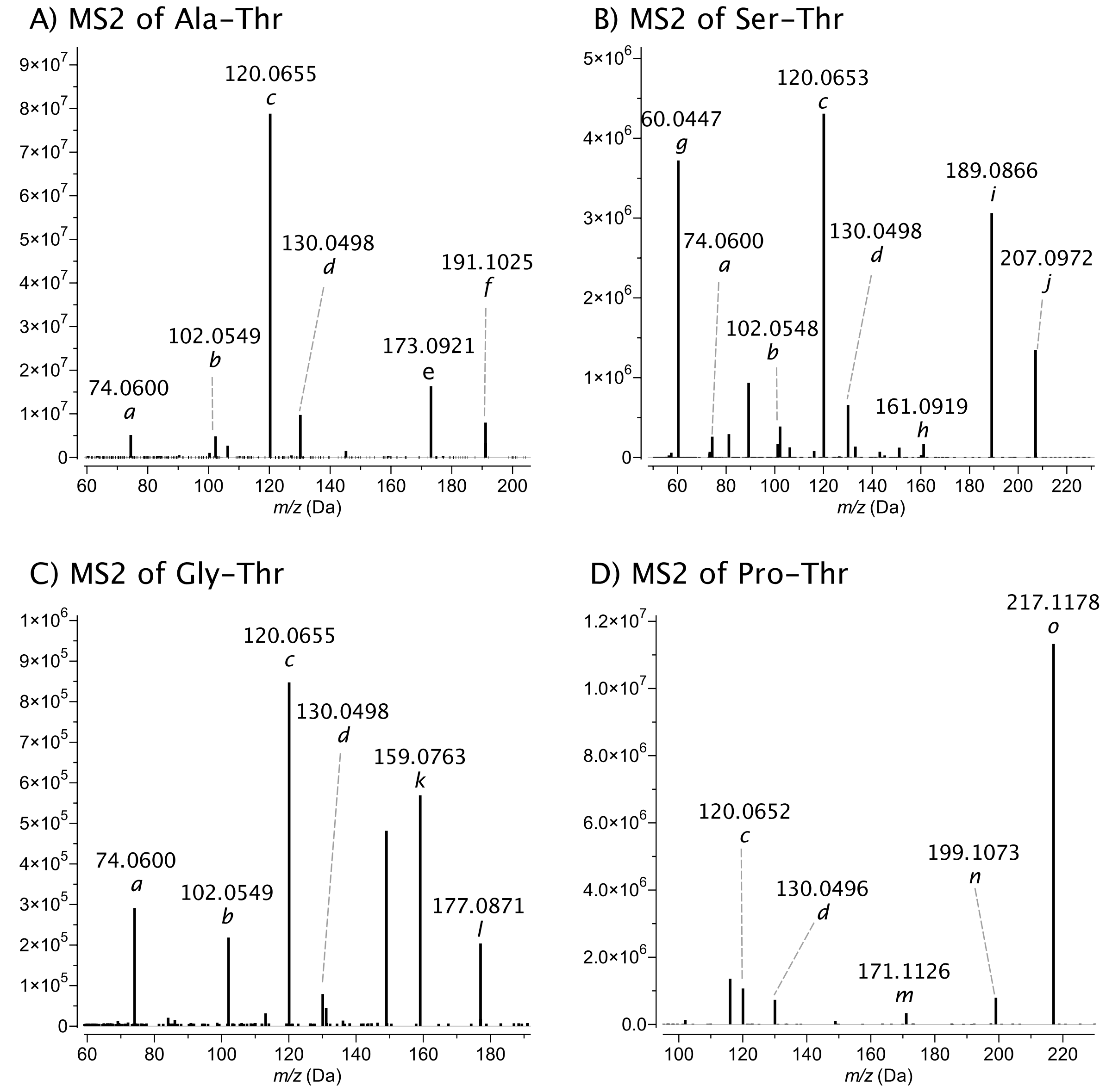

Figure S86. LC-MS/MS fragmentation of Ala-Thr, Ser-Thr, Gly-Thr, and Pro-Thr

Refer to Table S28 for fragment ion formula, structures, and mass error

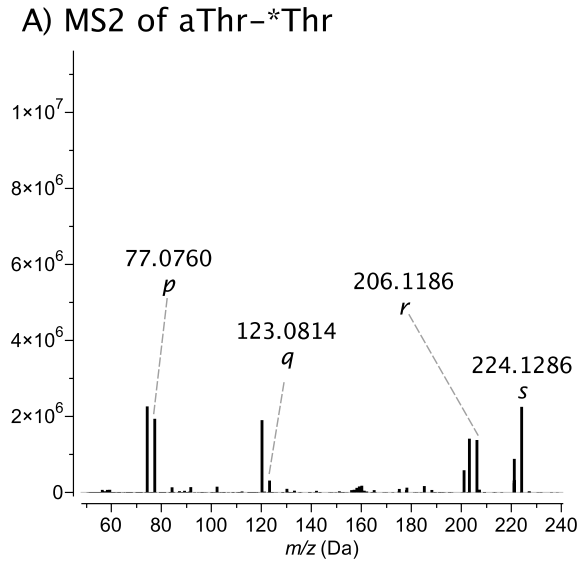

Figure S87. LC-MS/MS fragmentation of aThr-(4-^{13}C-2,3-D_2-Thr)

Refer to Table S29 for fragment ion formula, structures, and mass error

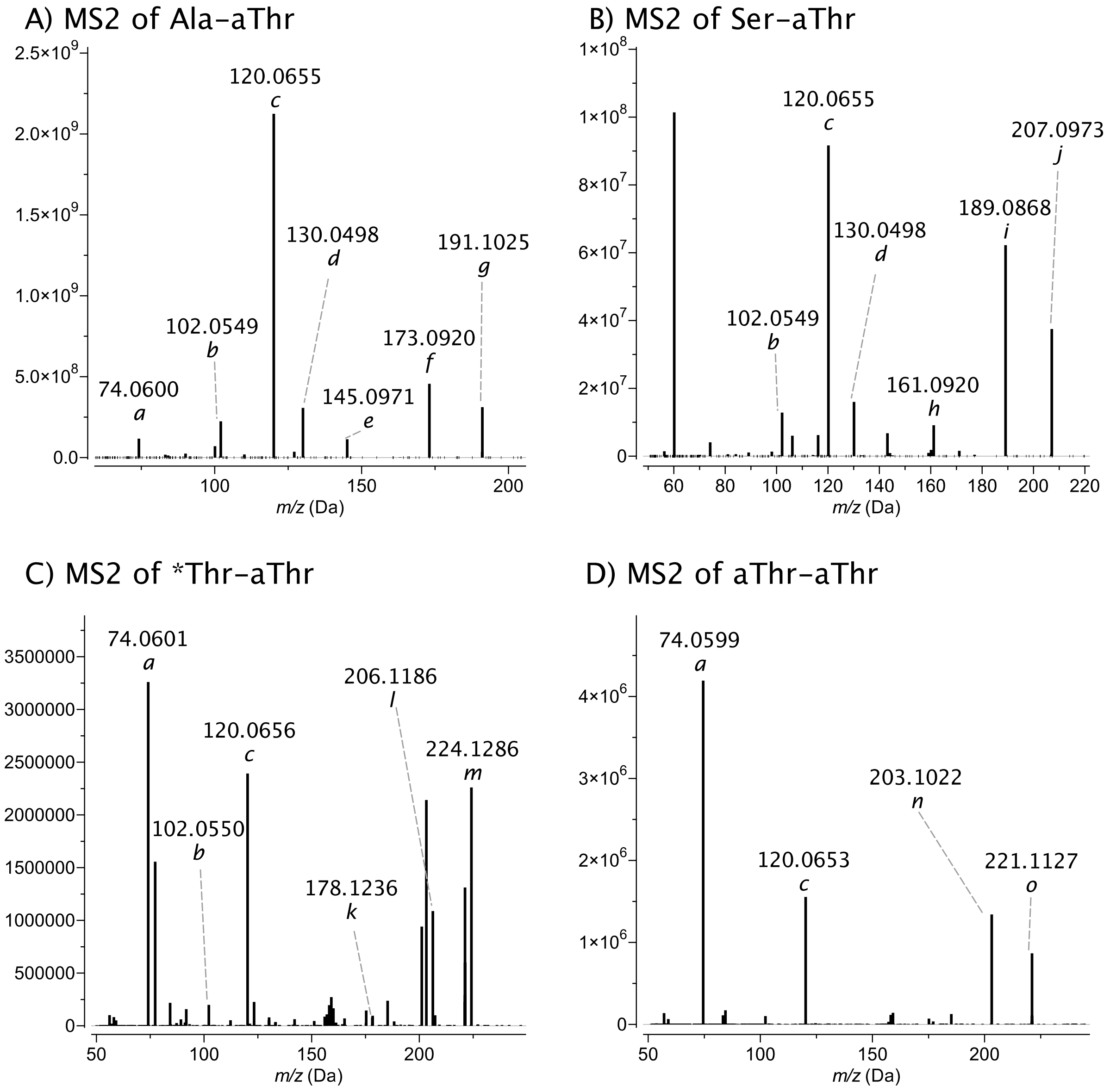

Figure S88. LC-MS/MS fragmentation of Ala-aThr, Ser-aThr, (4-^{13}C-2,3-D_2-Thr)-aThr, and aThr-aThr

Refer to Table S30 for fragment ion formula, structures, and mass error

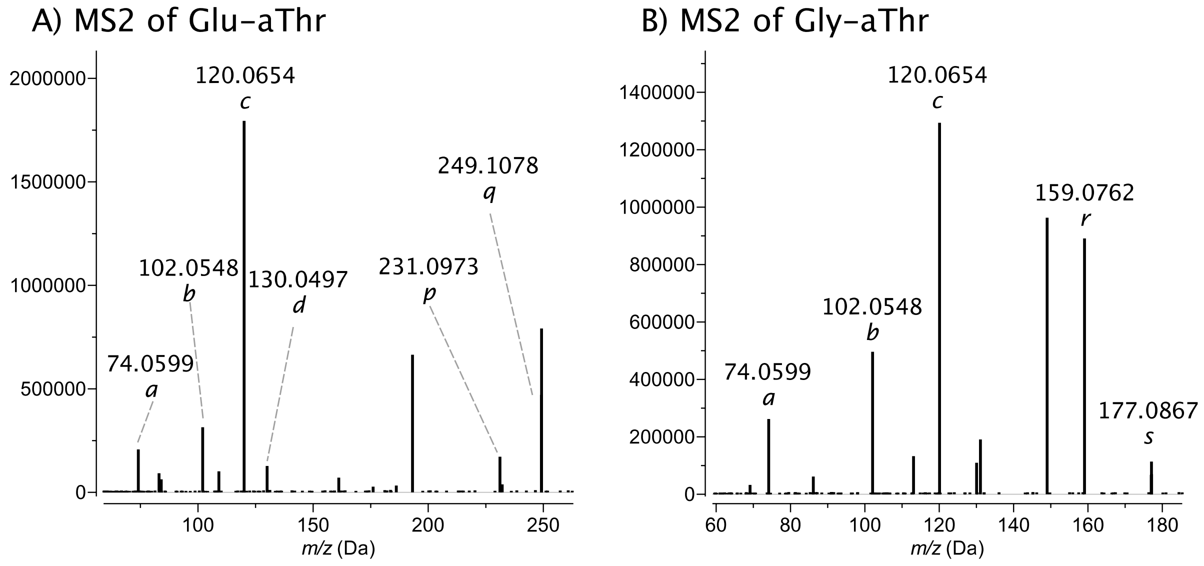

Figure S89. LC-MS/MS fragmentation of Glu-aThr and Gly-aThr

Refer to Table S31 for fragment ion formula, structures, and mass error

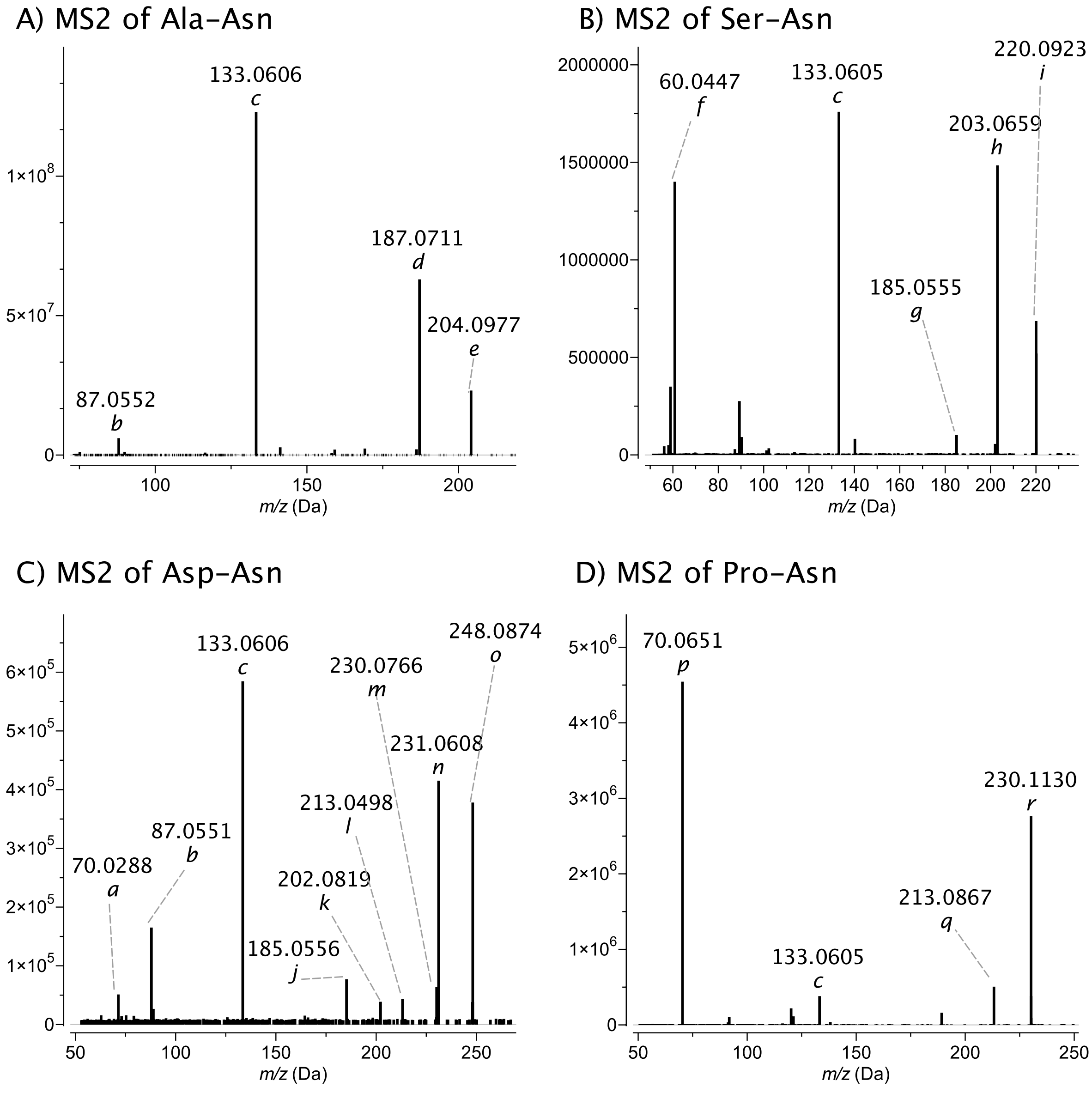

Figure S90. LC-MS/MS fragmentation of Ala-Asn, Ser-Asn, Asp-Asn, and Pro-Asn

Refer to Table S32 for fragment ion formula, structures, and mass error

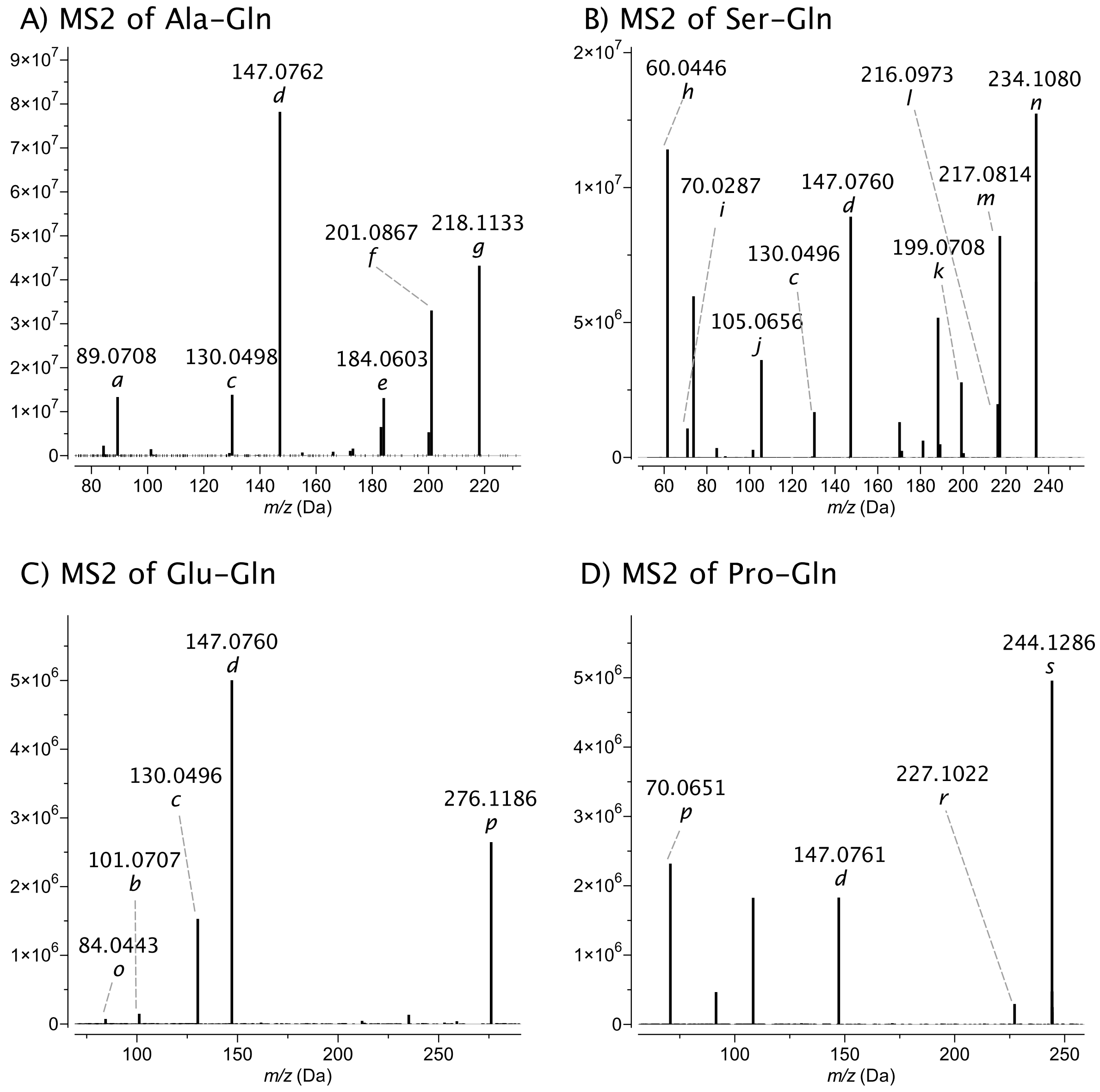

Figure S91. LC-MS/MS fragmentation of Ala-Gln, Ser-Gln, Glu-Gln, and Pro-Gln

Refer to Table S33 for fragment ion formula, structures, and mass error

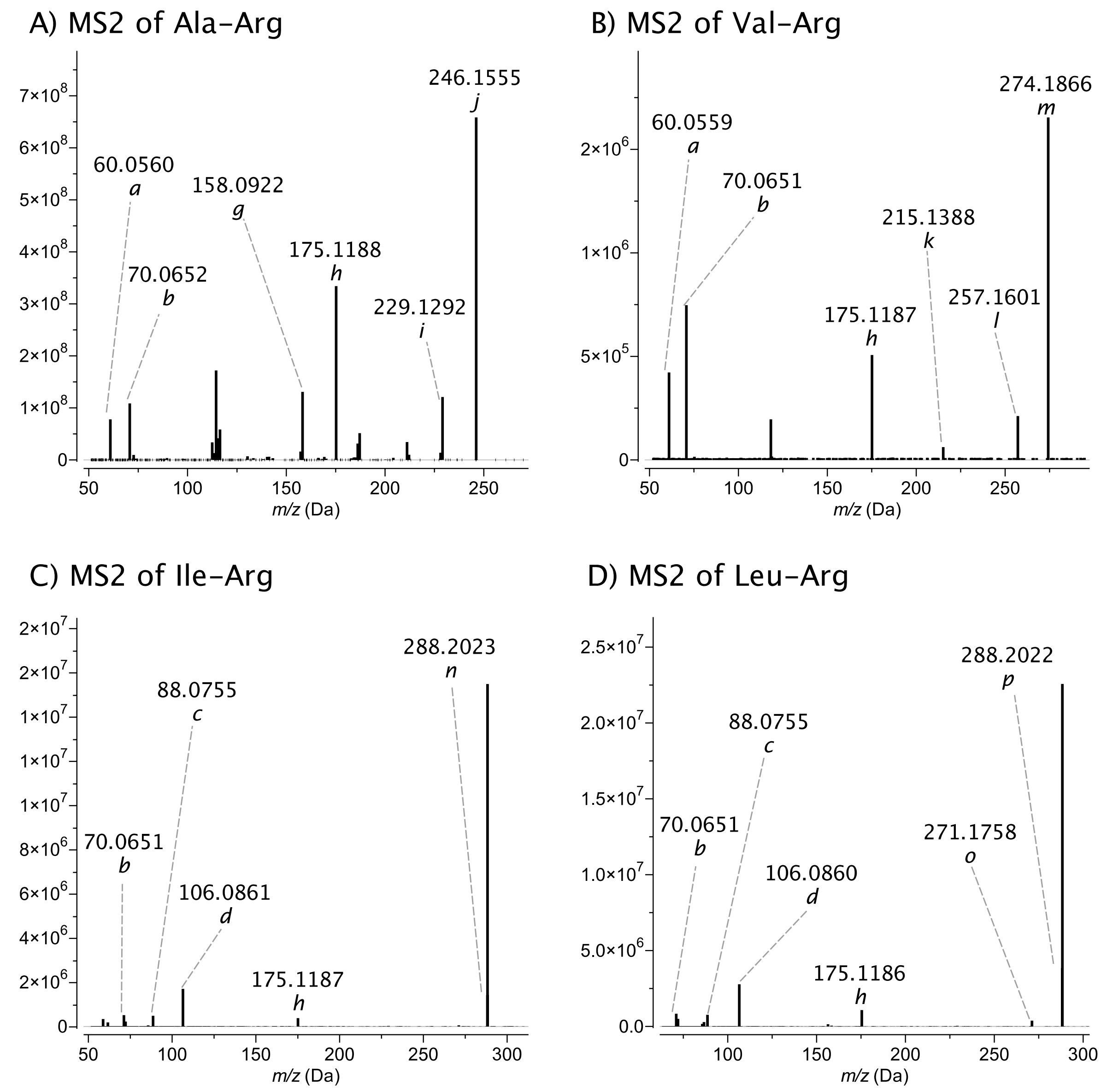

Figure S92. LC-MS/MS fragmentation of Ala-Arg, Val-Arg, Ile-Arg, and Leu-Arg

Refer to Table S34 for fragment ion formula, structures, and mass error

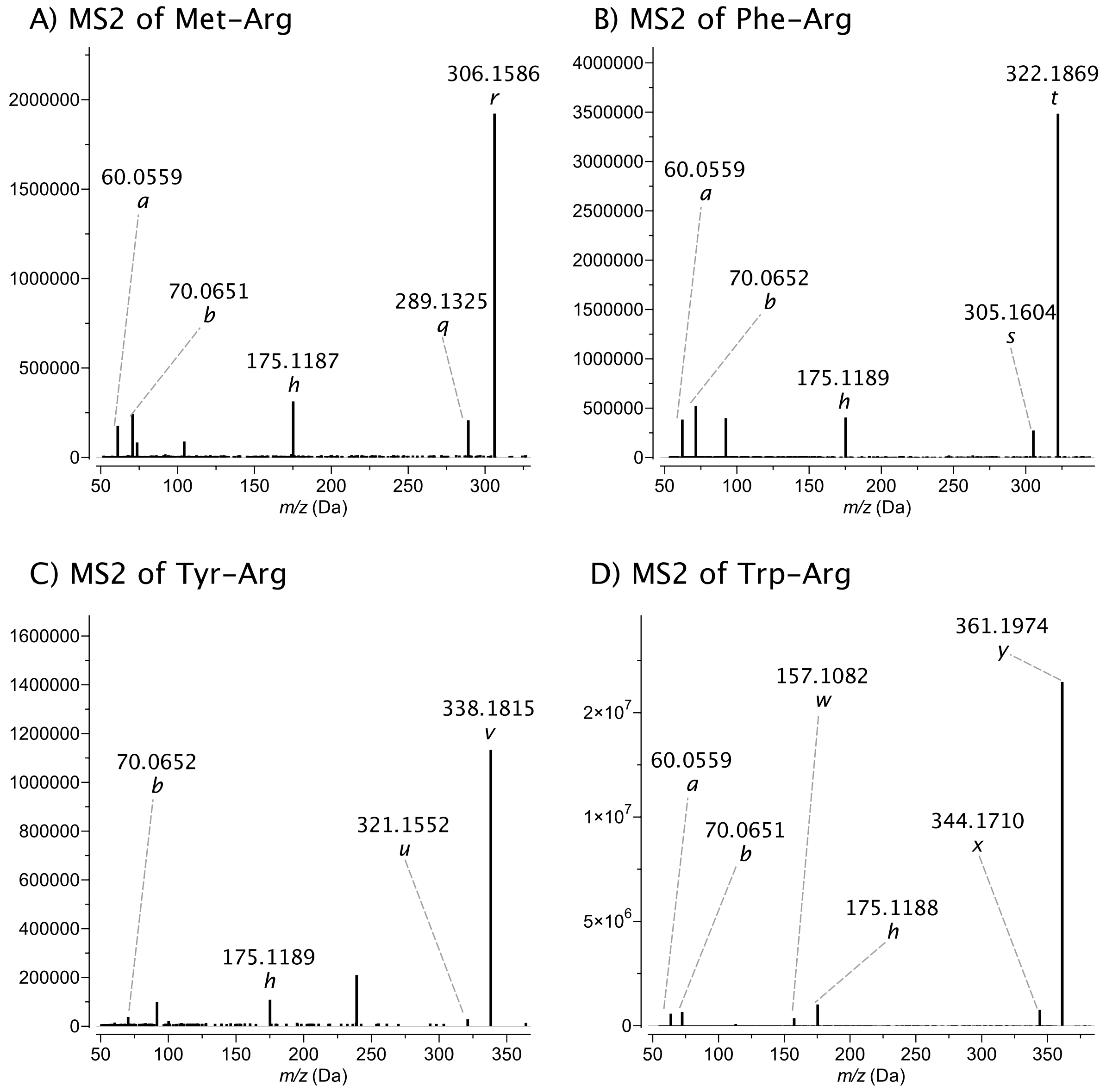

Figure S93. LC-MS/MS fragmentation of Met-Arg, Phe-Arg, Tyr-Arg, and Trp-Arg

Refer to Table S35 for fragment ion formula, structures, and mass error

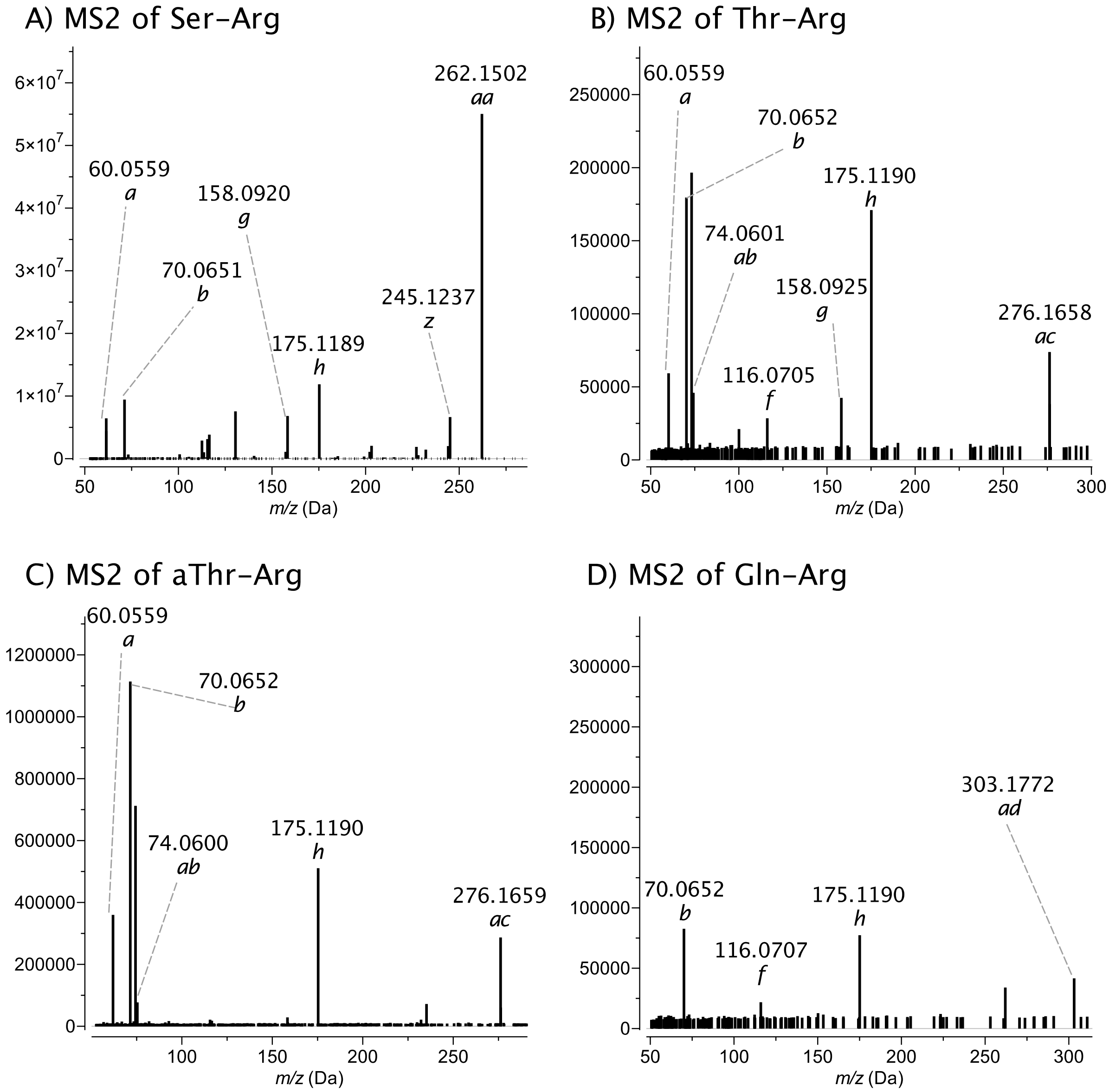

Figure S94. LC-MS/MS fragmentation of Ser-Arg, Thr-Arg, aThr-Arg, and Gln-Arg

Refer to Table S36 for fragment ion formula, structures, and mass error

A) MS2 of Asp-Arg

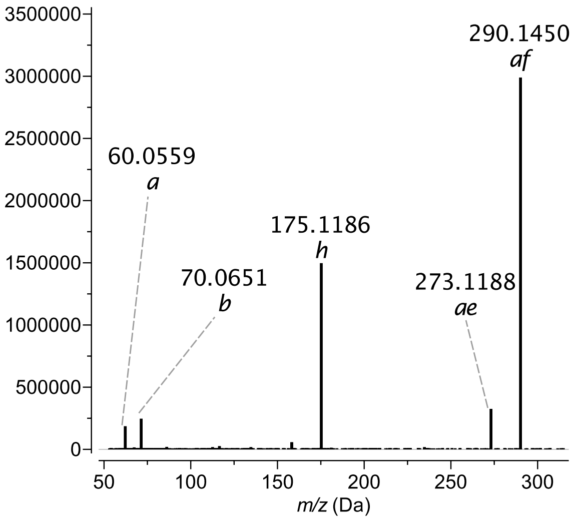

B) MS2 of Gly-Arg

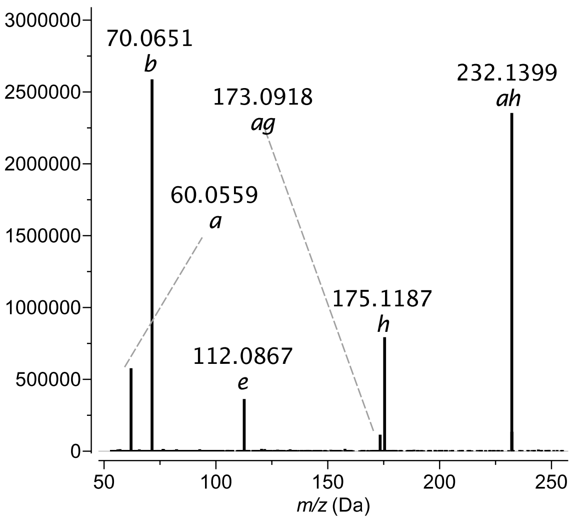

C) MS2 of Pro-Arg

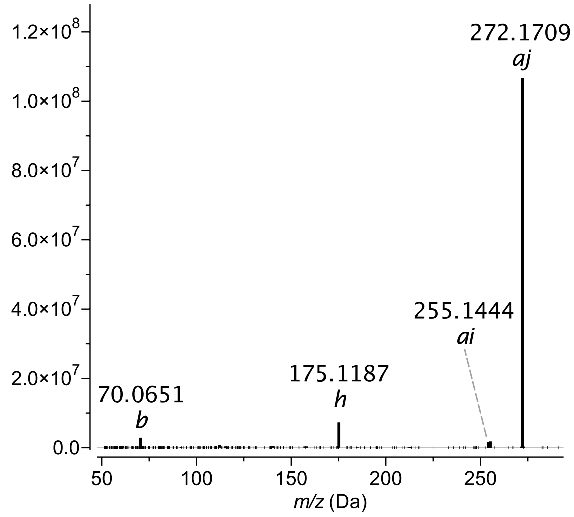

Figure S95. LC-MS/MS fragmentation of Asp-Arg, Gly-Arg, and Pro-Arg

Refer to Table S37 for fragment ion formula, structures, and mass error

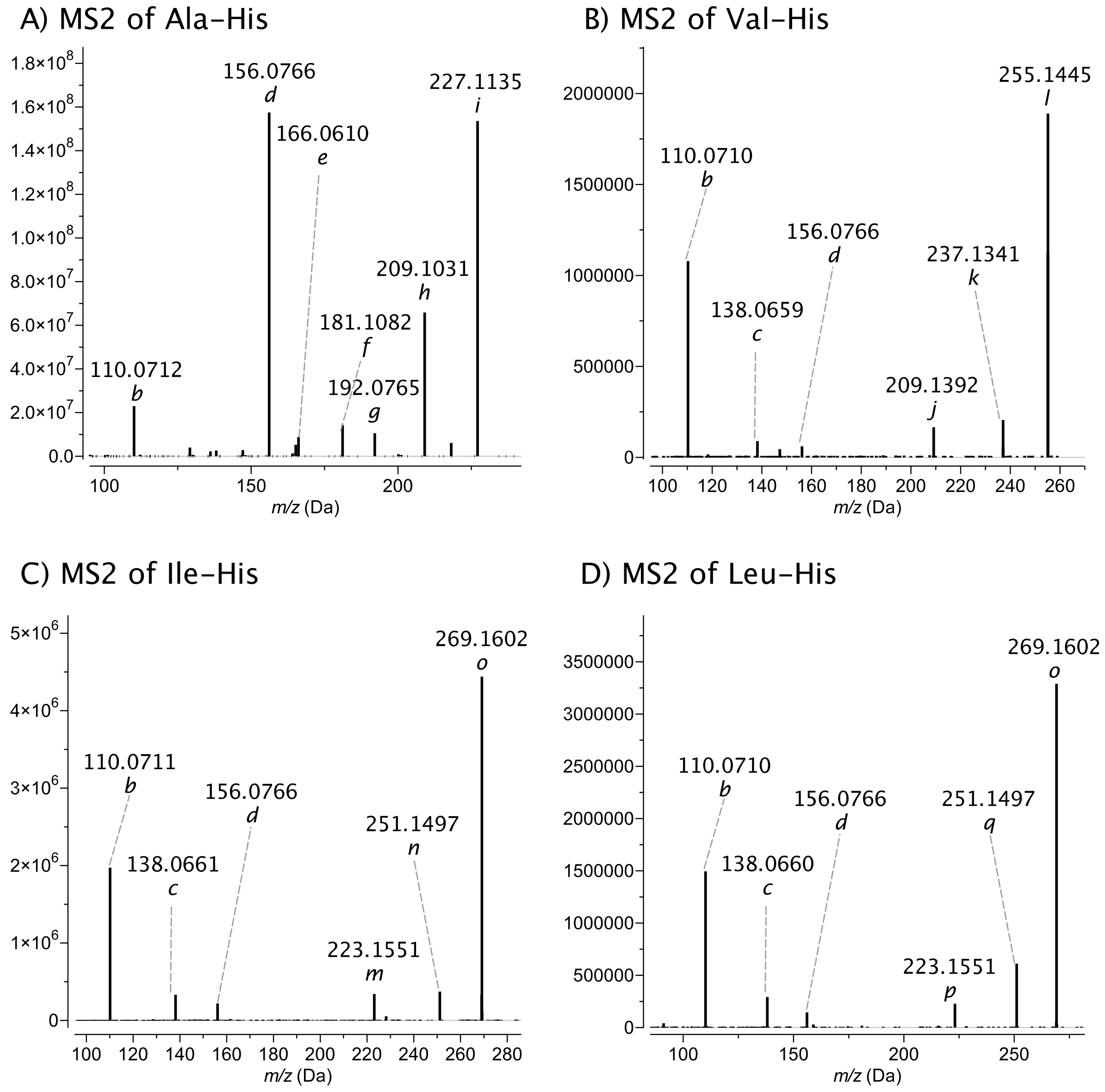

Figure S96. LC-MS/MS fragmentation of Ala-His, Val-His, Ile-His, and Leu-His

Refer to Table S38 for fragment ion formula, structures, and mass error

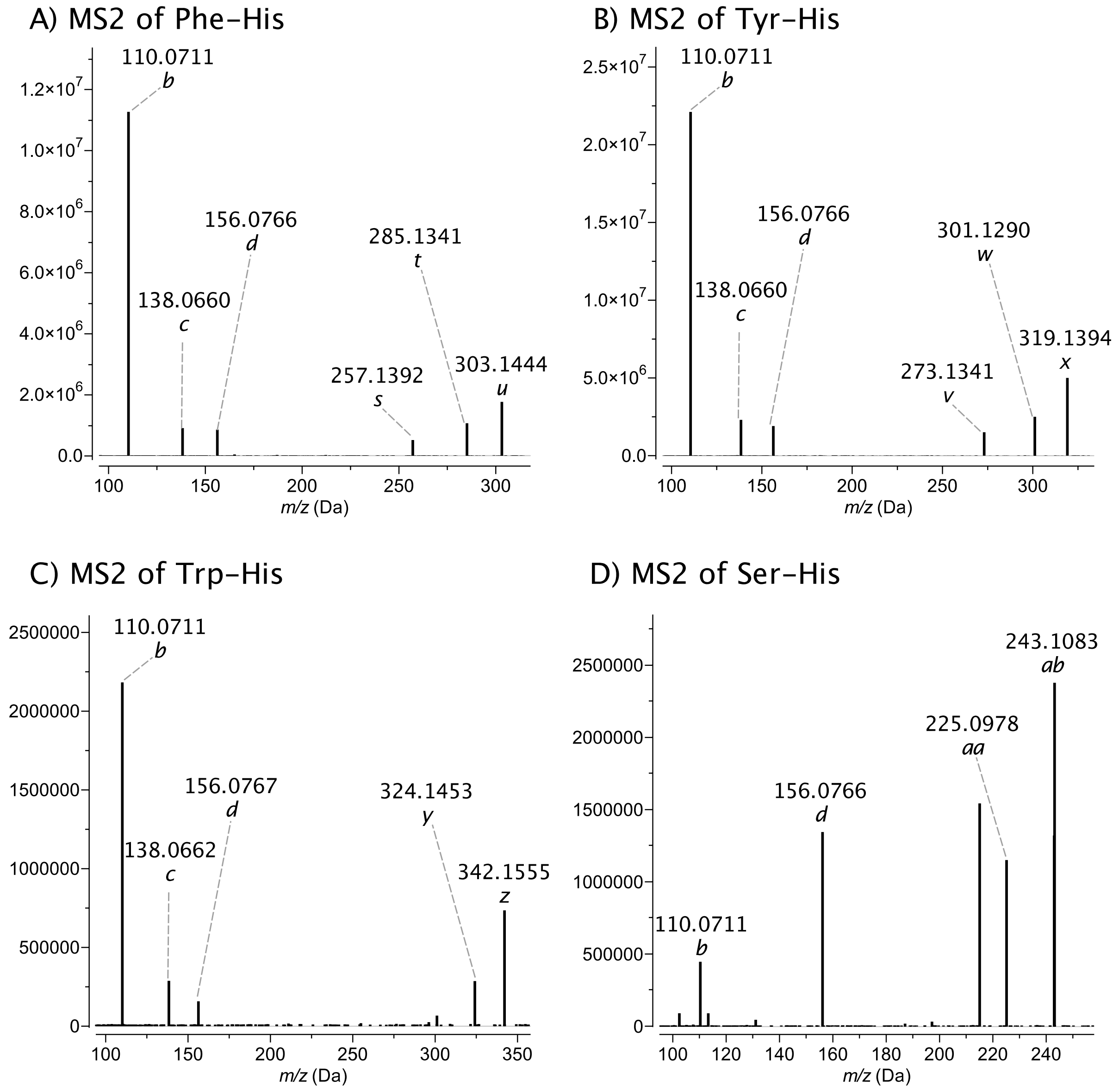

Figure S97. LC-MS/MS fragmentation of Phe-His, Tyr-His, Trp-His, and Ser-His

Refer to Table S39 for fragment ion formula, structures, and mass error

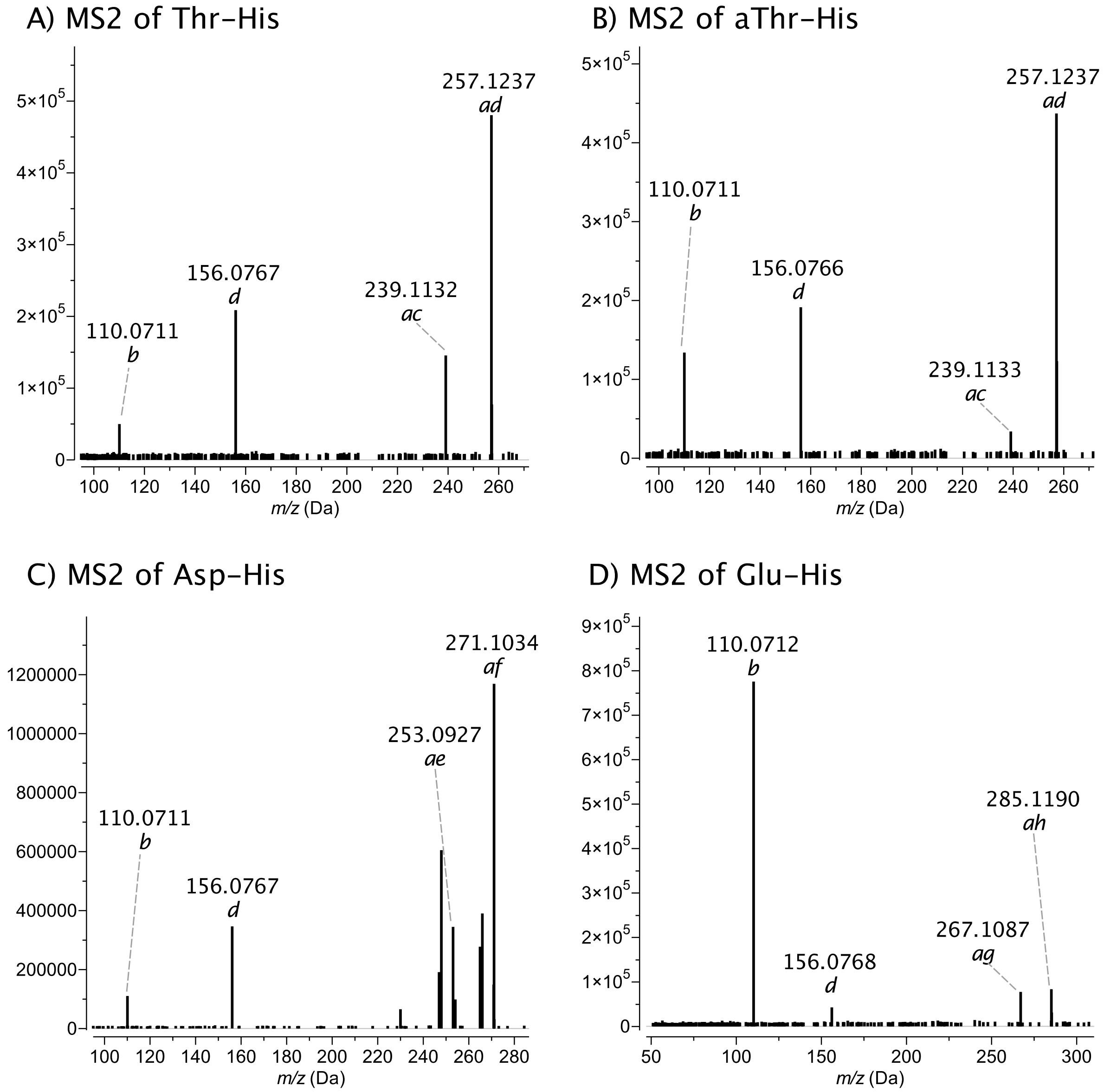

Figure S98. LC-MS/MS fragmentation of Thr-His, aThr-His, Asp-His, and Glu-His

Refer to Table S40 for fragment ion formula, structures, and mass error

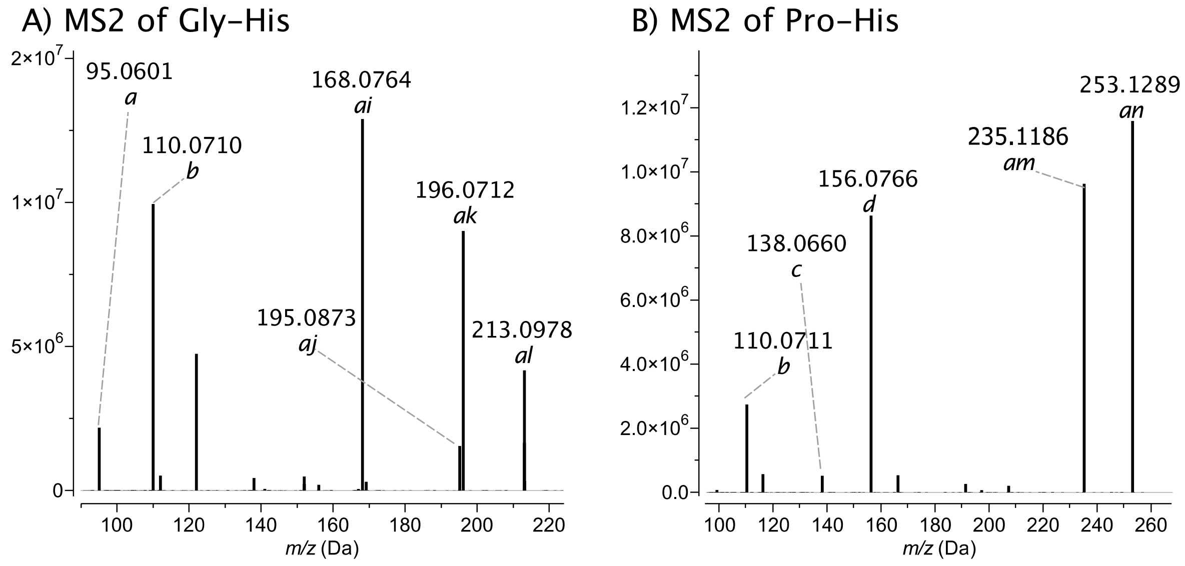

Figure S99. LC-MS/MS fragmentation of Gly-His and Pro-His

Refer to Table S41 for fragment ion formula, structures, and mass error

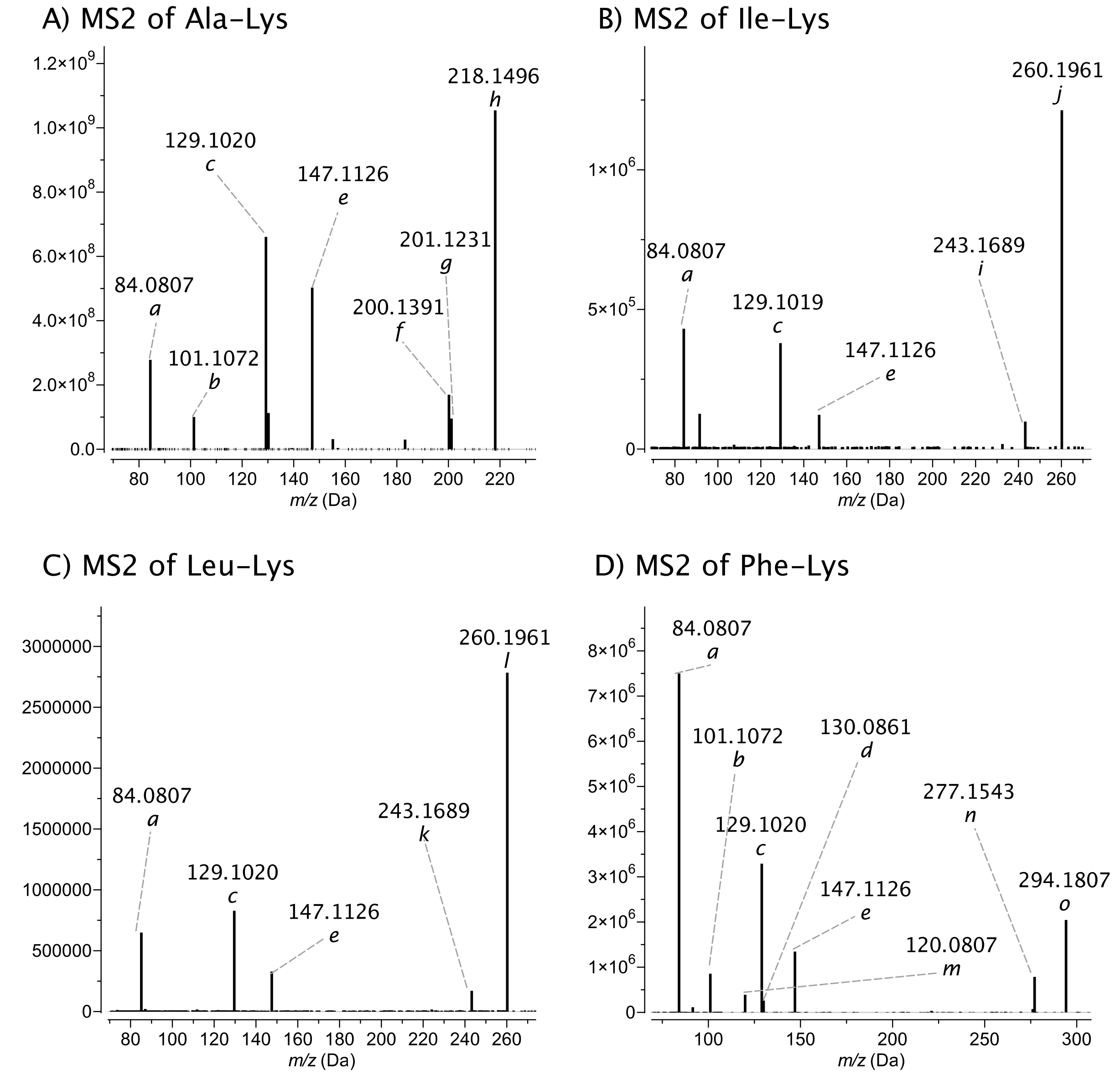

Figure S100. LC-MS/MS fragmentation of Ala-Lys, Ile-Lys, Leu-Lys, and Phe-Lys

Refer to Table S42 for fragment ion formula, structures, and mass error

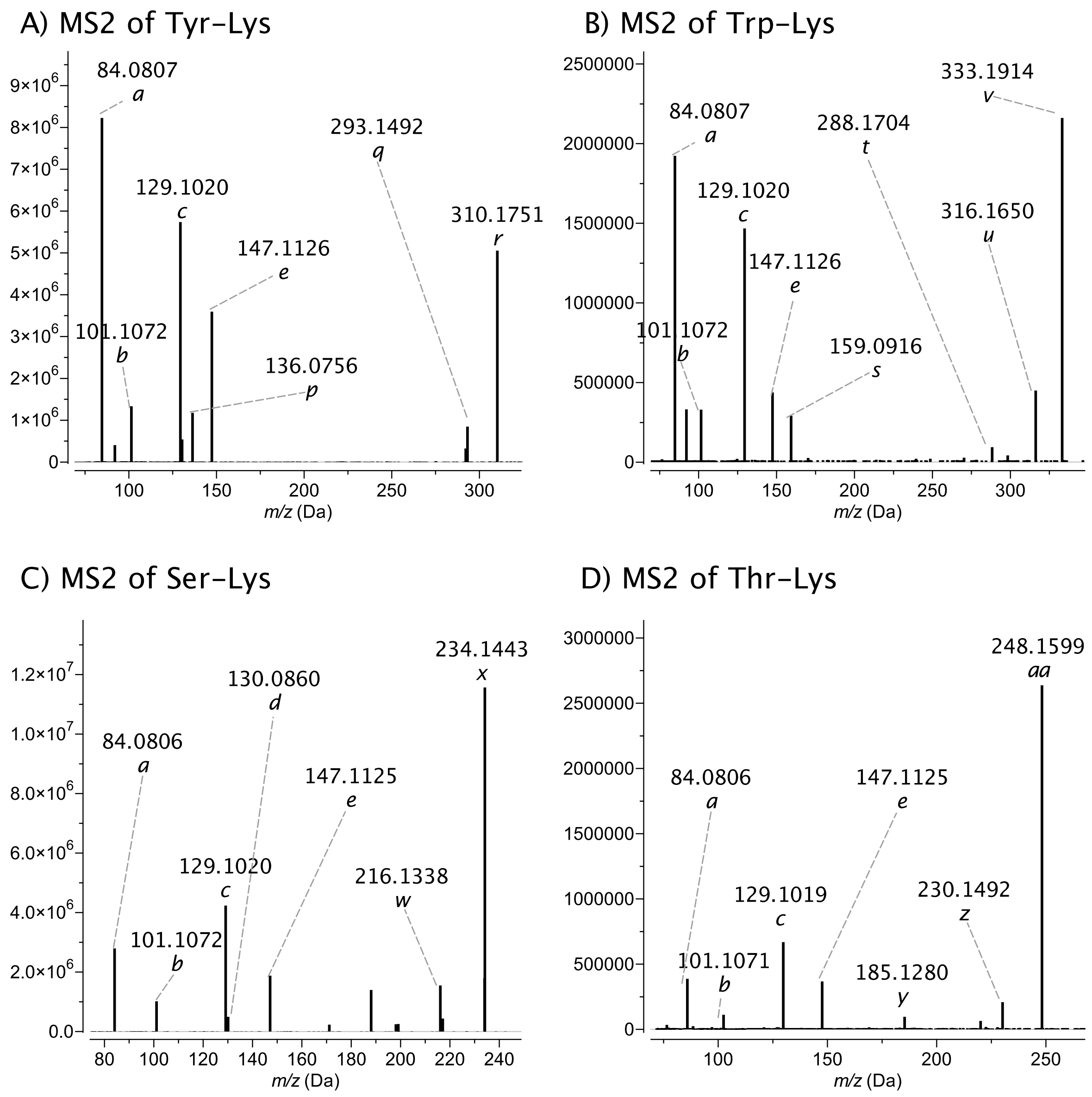

Figure S101. LC-MS/MS fragmentation of Tyr-Lys, Trp-Lys, Ser-Lys, and Thr-Lys

Refer to Table S43 for fragment ion formula, structures, and mass error

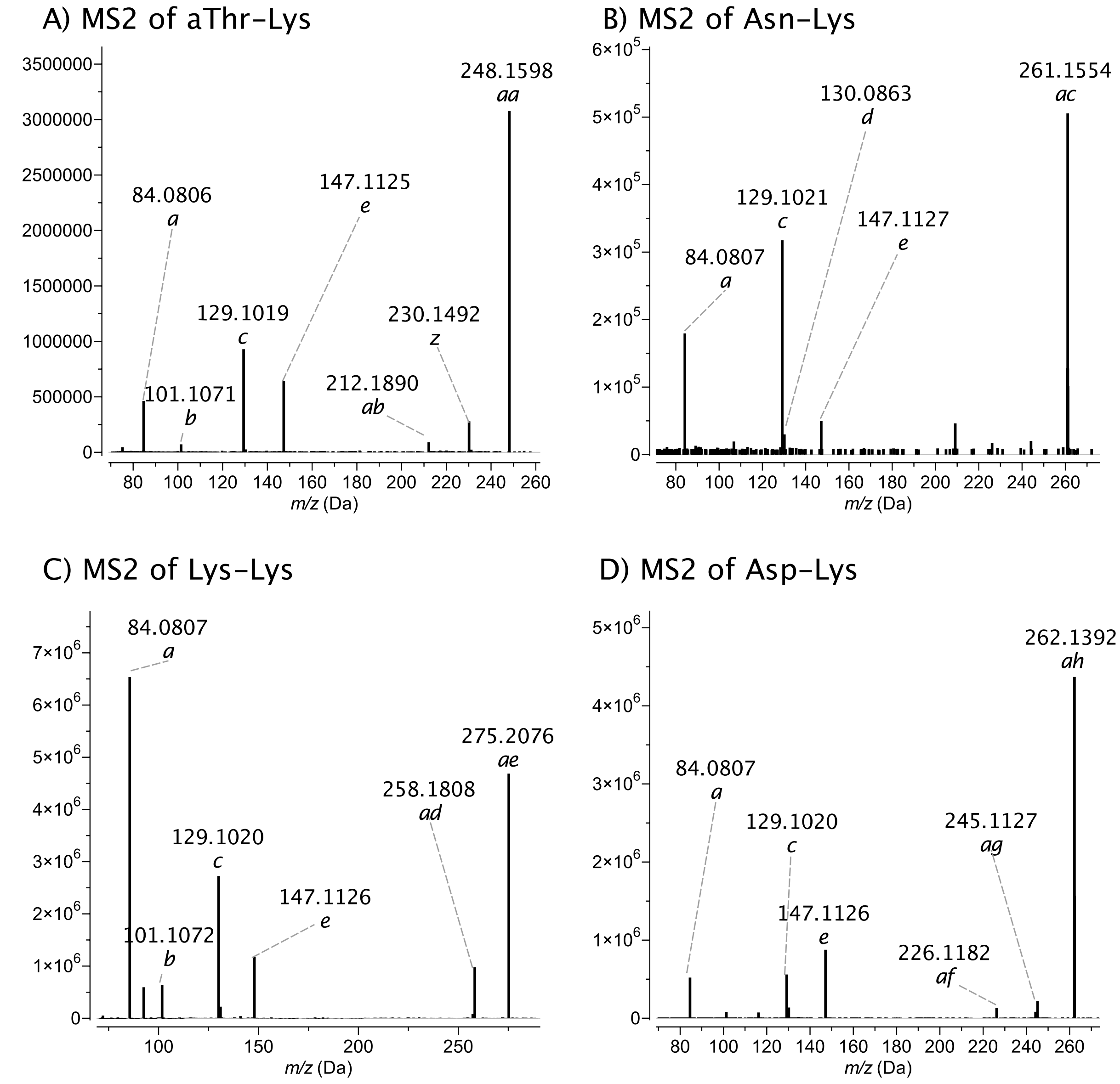

Figure S102. LC-MS/MS fragmentation of aThr-Lys, Asn-Lys, Lys-Lys, and Asp-Lys

Refer to Table S44 for fragment ion formula, structures, and mass error

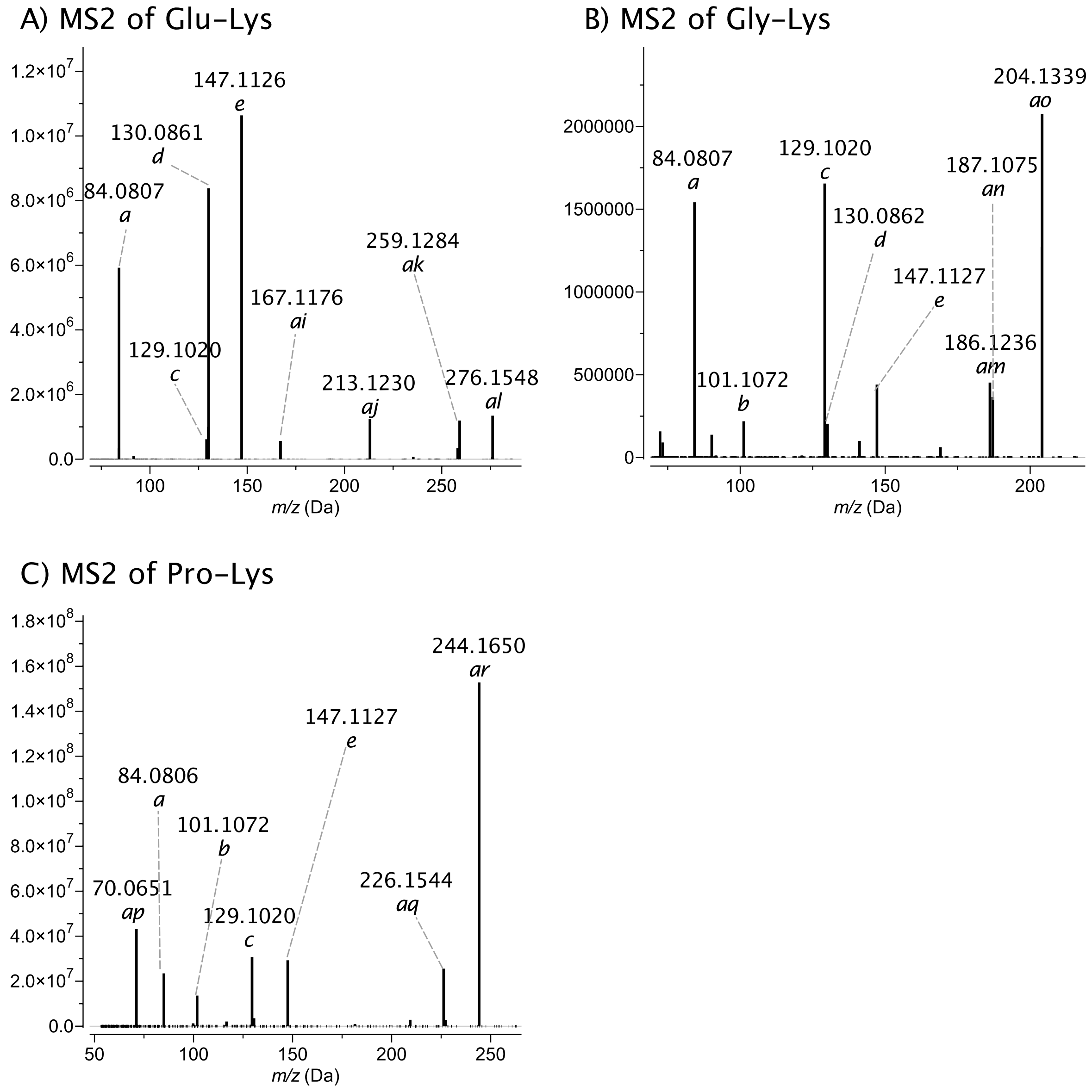

Figure S103. LC-MS/MS fragmentation of Glu-Lys, Gly-Lys, and Pro-Lys

Refer to Table S45 for fragment ion formula, structures, and mass error

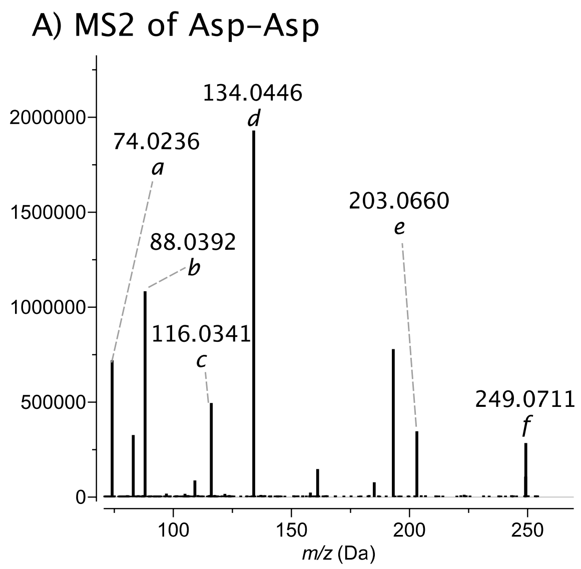

Figure S104. LC-MS/MS fragmentation of Asp-Asp

Refer to Table S46 for fragment ion formula, structures, and mass error

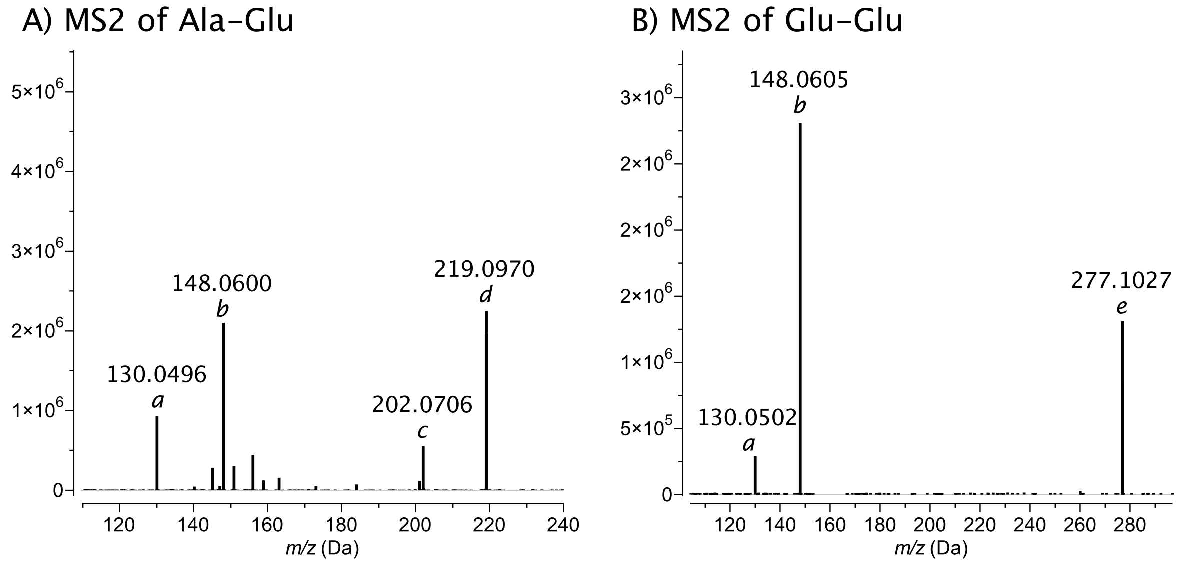

Figure S105. LC-MS/MS fragmentation of Ala-Glu and Glu-Glu
Refer to Table S47 for fragment ion formula, structures, and mass error

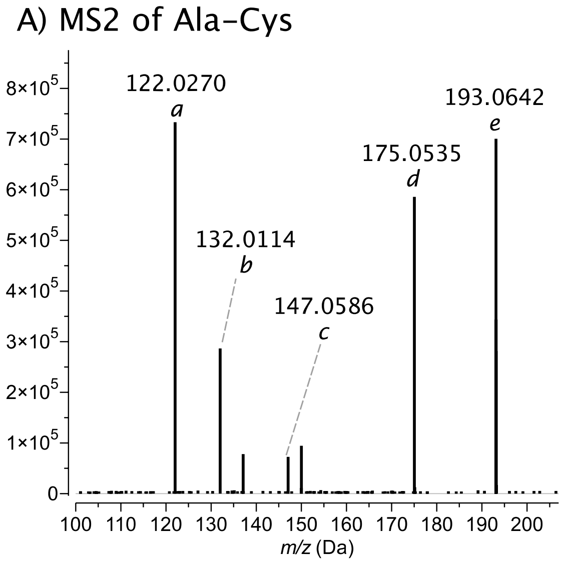

Figure S106. LC-MS/MS fragmentation of Ala-Cys

Refer to Table S48 for fragment ion formula, structures, and mass error

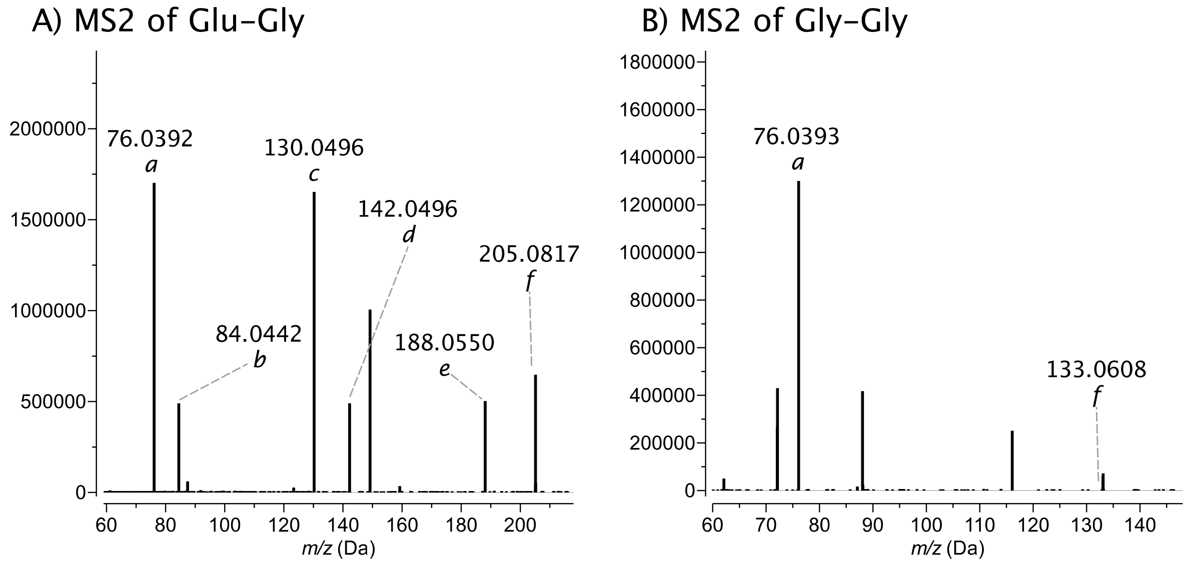

Figure S107. LC-MS/MS fragmentation of Glu-Gly and Gly-Gly

Refer to Table S49 for fragment ion formula, structures, and mass error

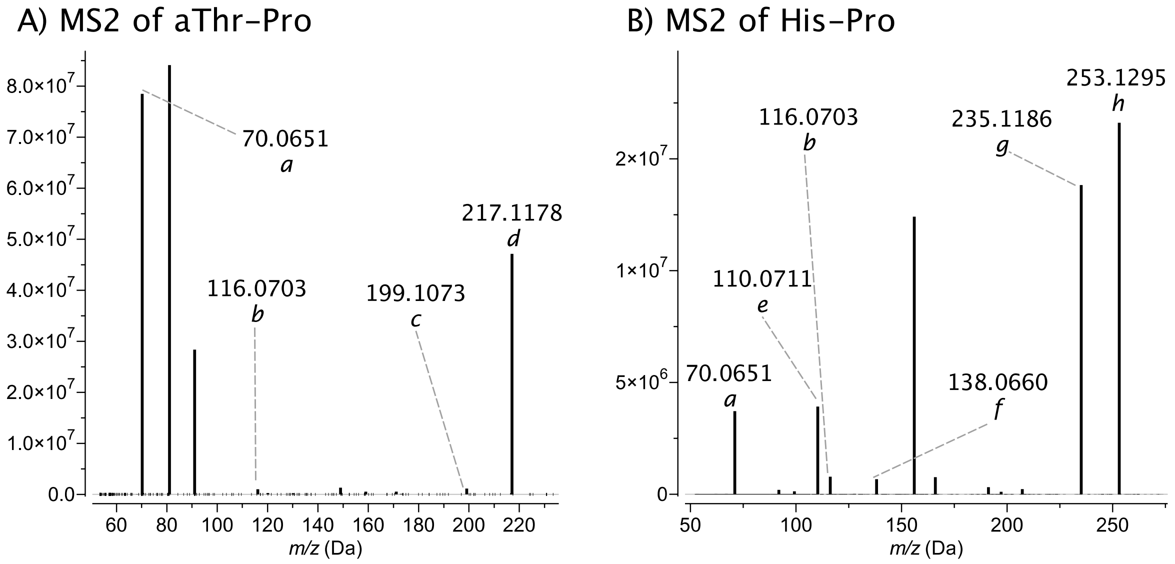

Figure S108. LC-MS/MS fragmentation of aThr-Pro and His-Pro

Refer to Table S50 for fragment ion formula, structures, and mass error

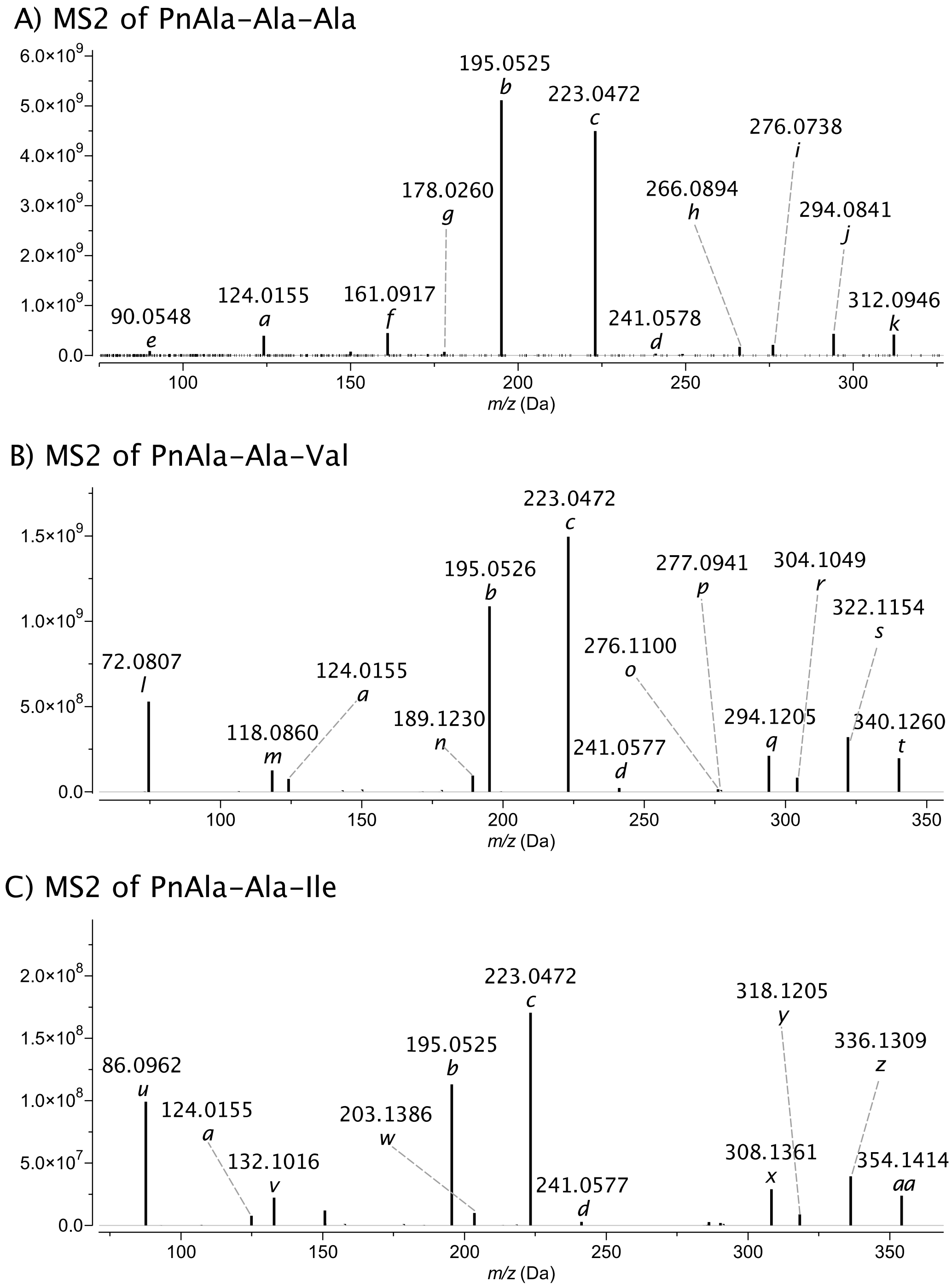

Figure S109. LC-MS/MS fragmentation of PnAla-Ala-Ala, PnAla-Ala-Val, and PnAla-Ala-Ile

Refer to Table S51 for fragment ion formula, structures, and mass error

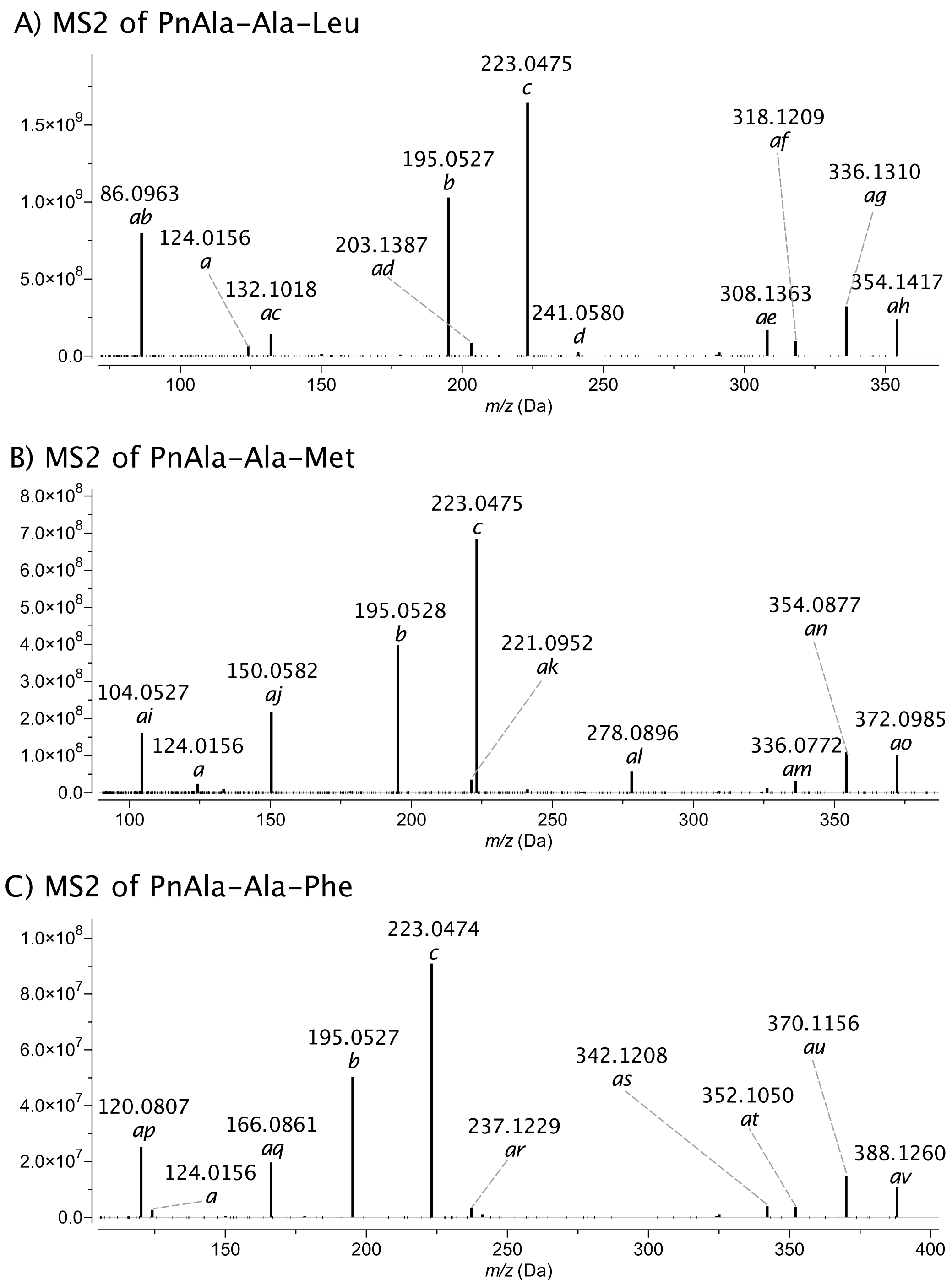

Figure S110. LC-MS/MS fragmentation of PnAla-Ala-Leu, PnAla-Ala-Met, and PnAla-Ala-Phe

Refer to Table S52 for fragment ion formula, structures, and mass error

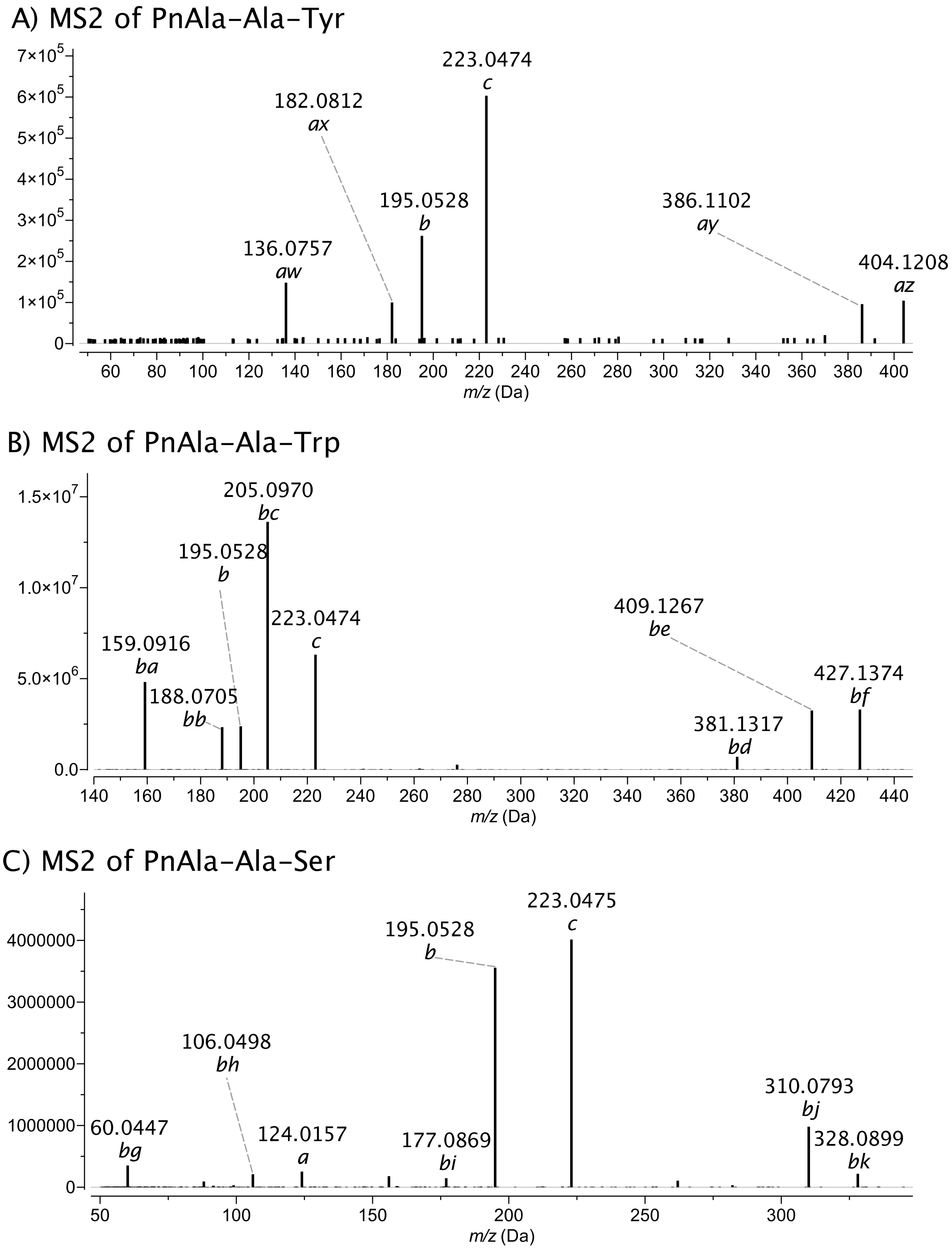

Figure S111. LC-MS/MS fragmentation of PnAla-Ala-Tyr, PnAla-Ala-Trp, and PnAla-Ala-Ser

Refer to Table S53 for fragment ion formula, structures, and mass error

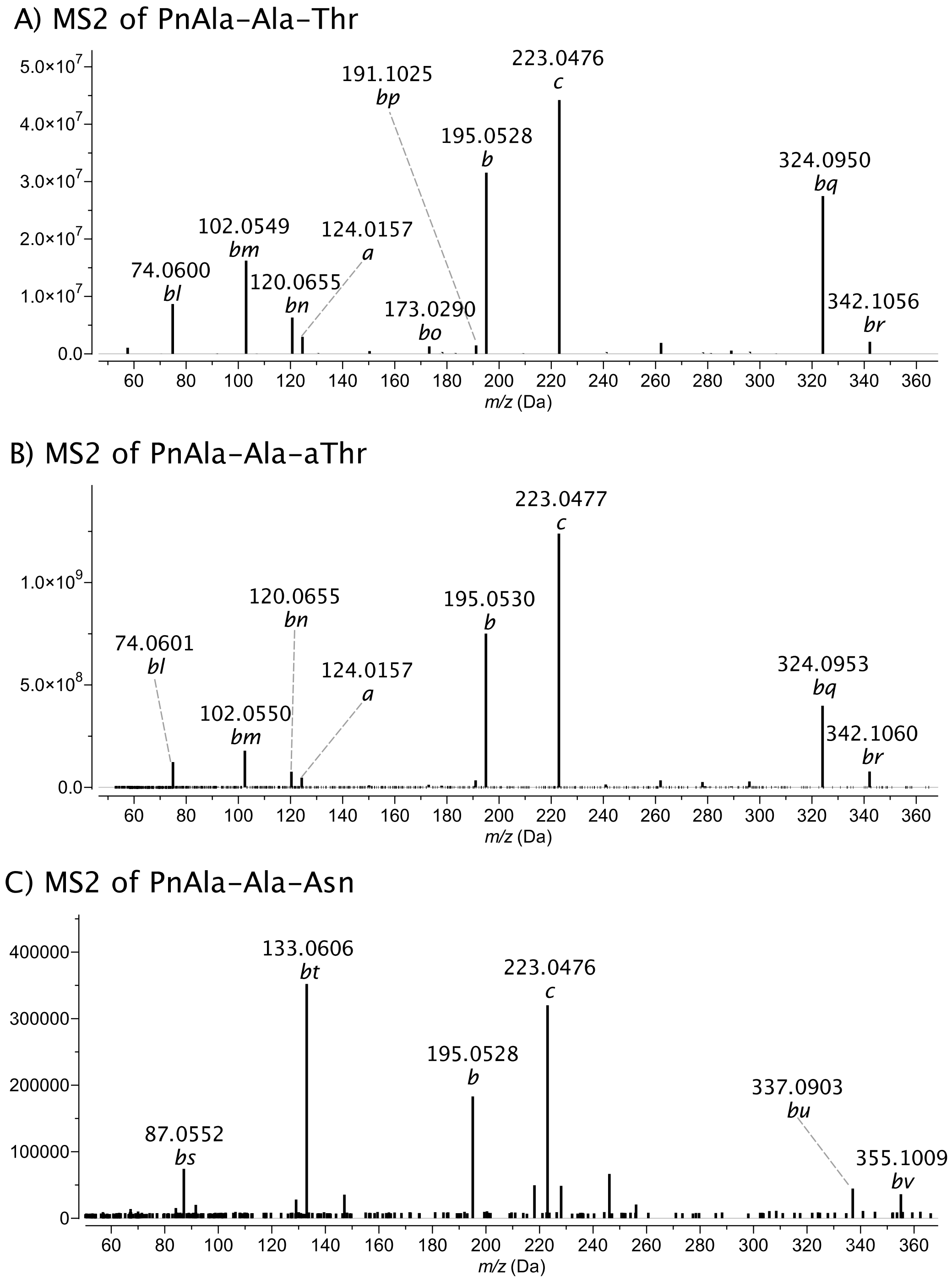

Figure S112. LC-MS/MS fragmentation of PnAla-Ala-Thr, PnAla-Ala-aThr, and PnAla-Ala-Asn

Refer to Table S54 for fragment ion formula, structures, and mass error

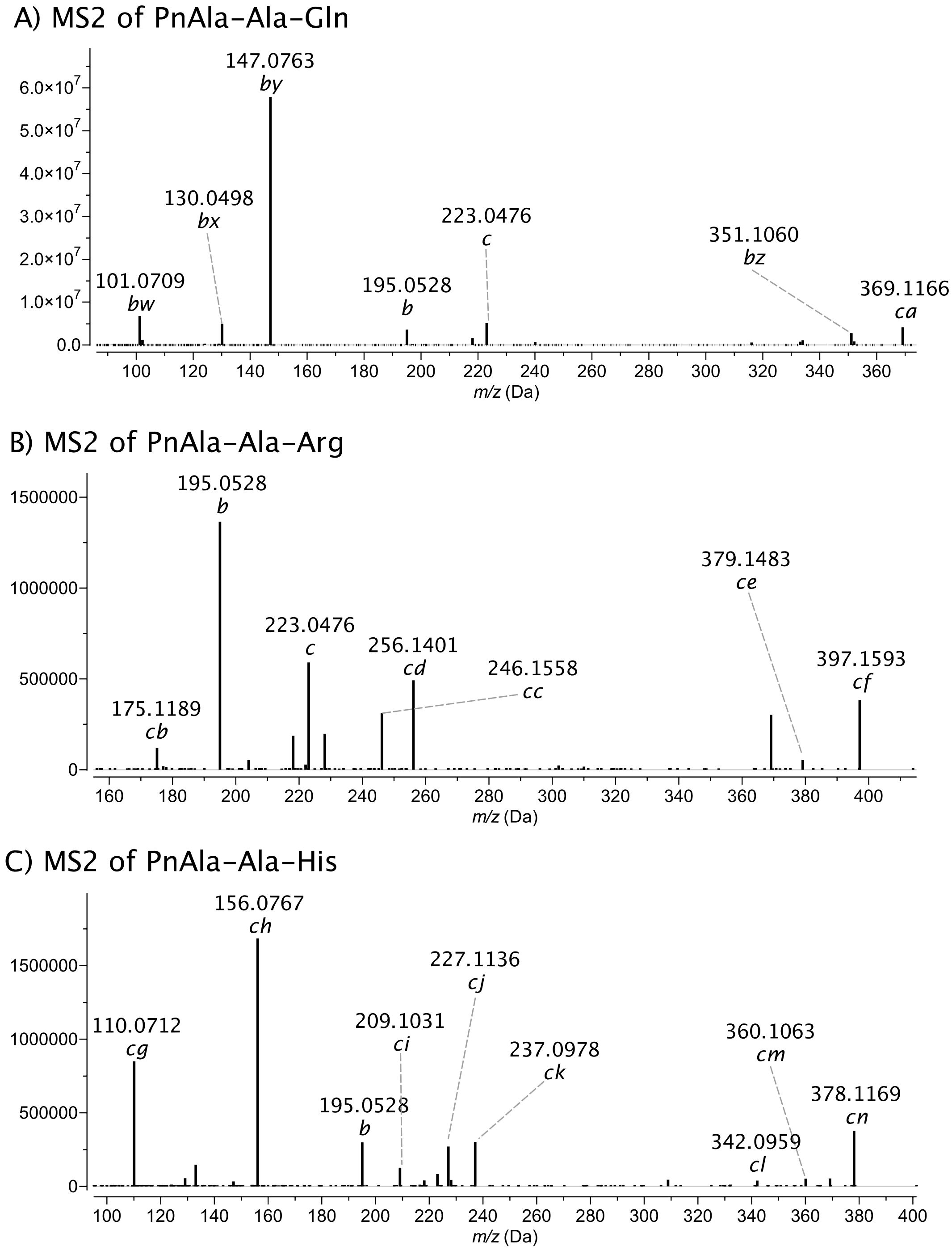

Figure S113. LC-MS/MS fragmentation of PnAla-Ala-Gln, PnAla-Ala-Arg, and PnAla-Ala-His
Refer to Table S55 for fragment ion formula, structures, and mass error

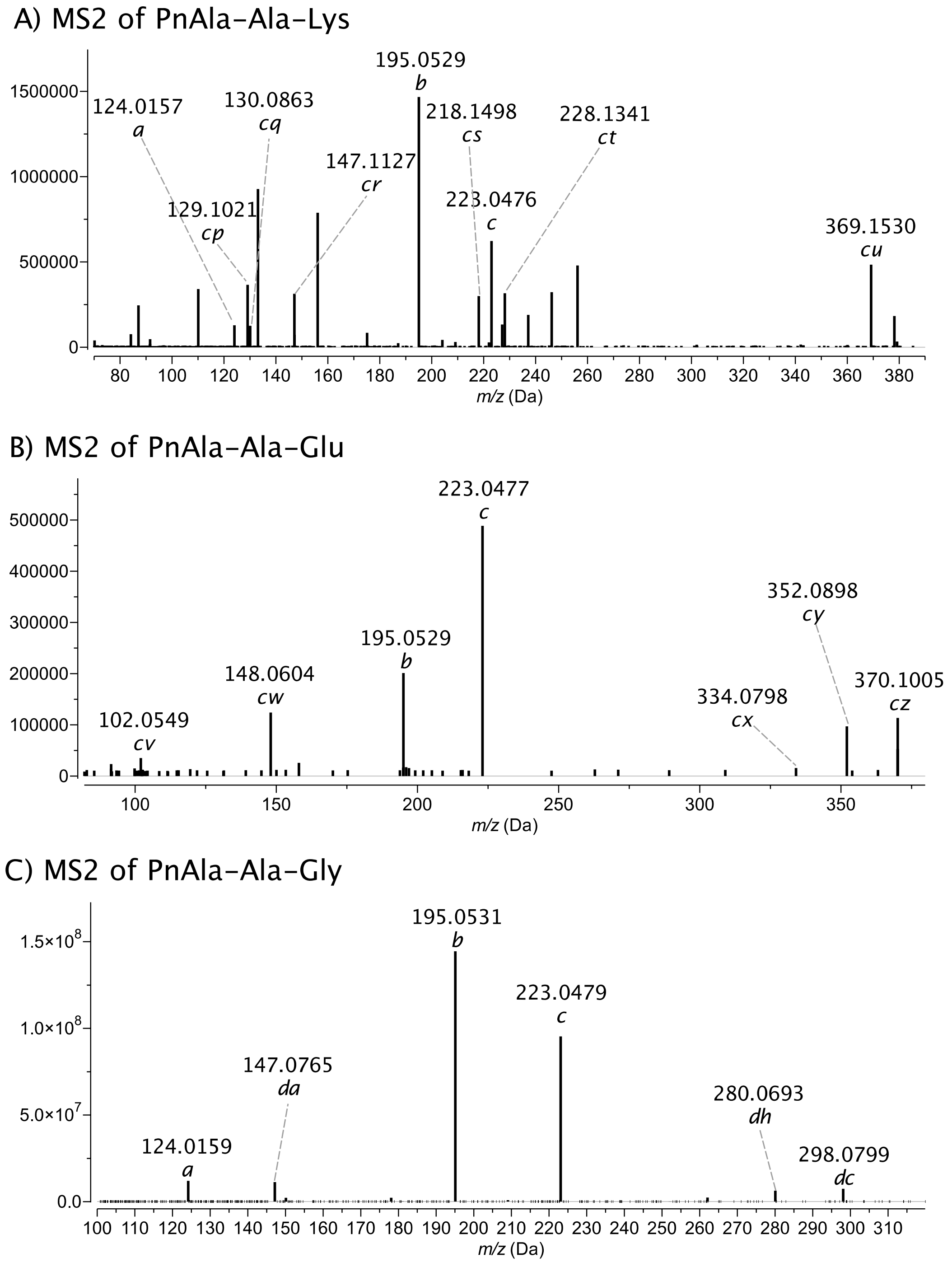

Figure S114. LC-MS/MS fragmentation of PnAla-Ala-Lys, PnAla-Ala-Glu, and PnAla-Ala-Gly

Refer to Table S56 for fragment ion formula, structures, and mass error

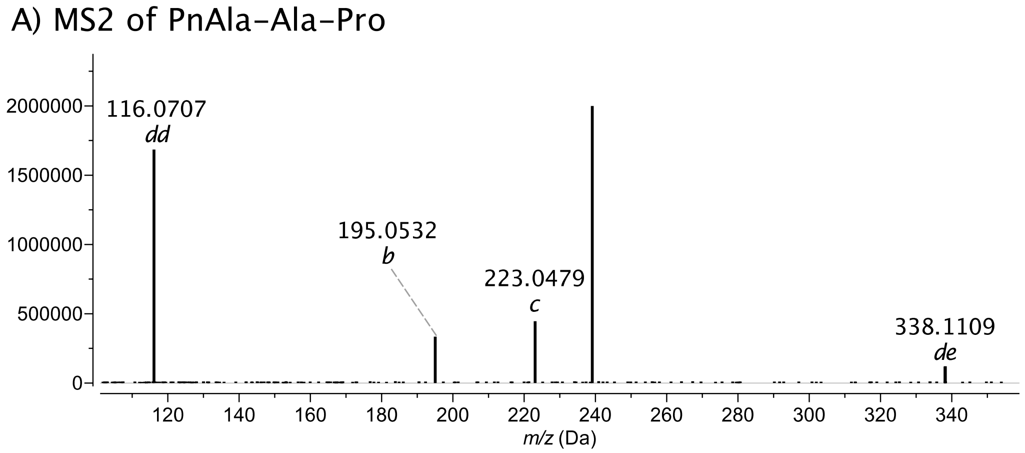

Figure S115. LC-MS/MS fragmentation of PnAla-Ala-Pro

Refer to Table S57 for fragment ion formula, structures, and mass error

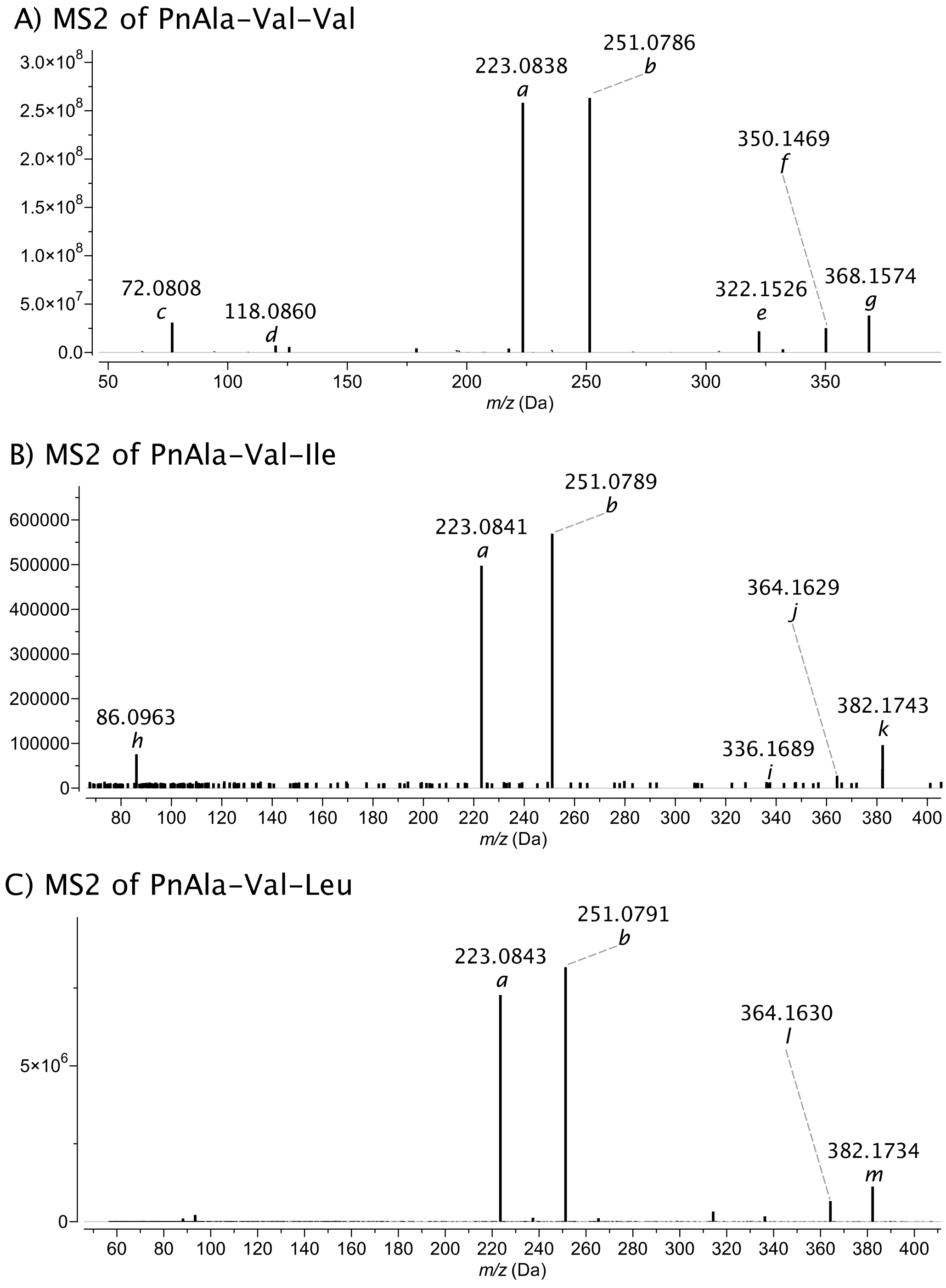

Figure S116. LC-MS/MS fragmentation of PnAla-Val-Val, PnAla-Val-Ile, and PnAla-Val-Leu

Refer to Table S58 for fragment ion formula, structures, and mass error

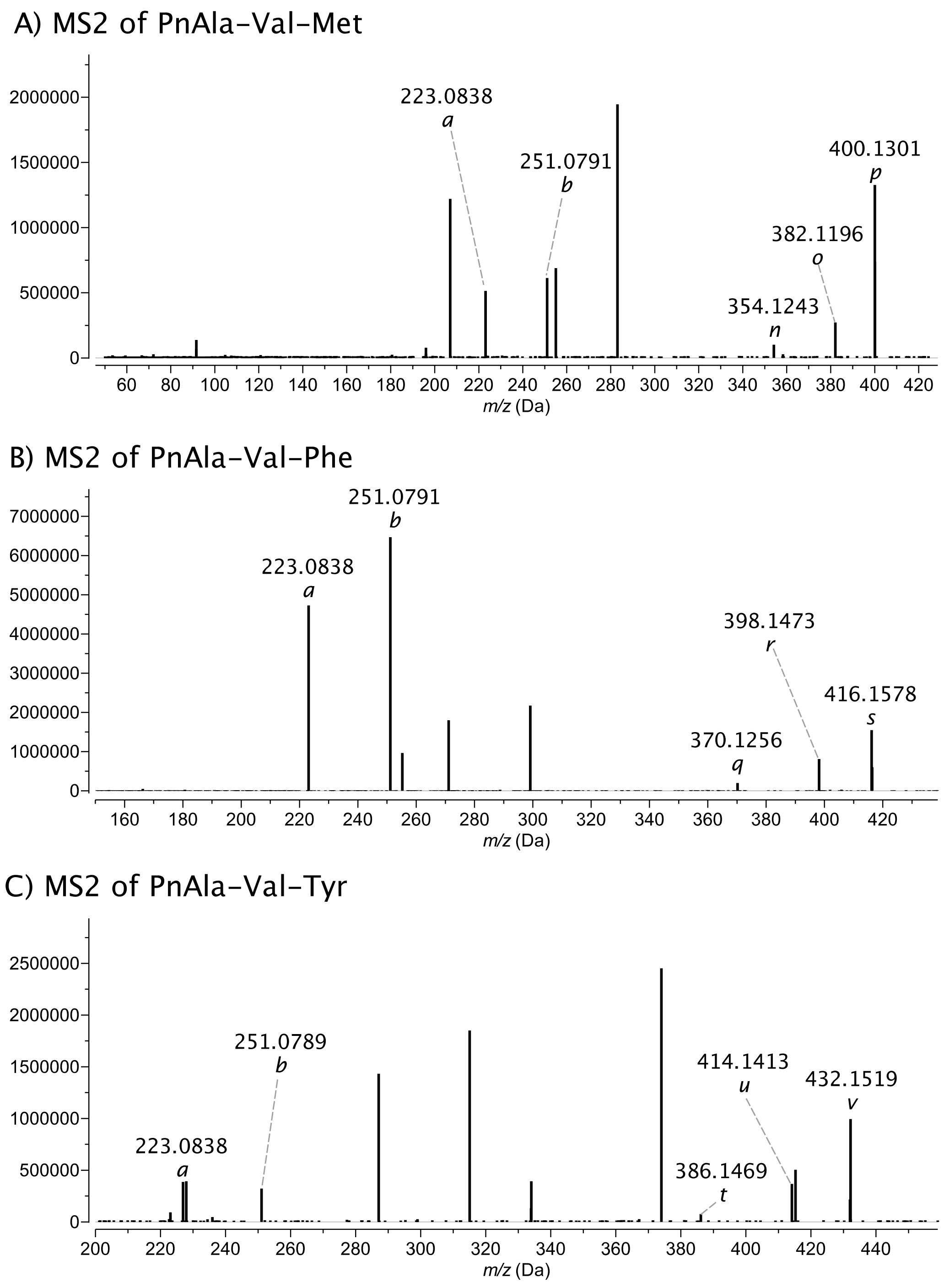

Figure S117. LC-MS/MS fragmentation of PnAla-Val-Met, PnAla-Val-Phe, and PnAla-Val-Tyr
Refer to Table S59 for fragment ion formula, structures, and mass error

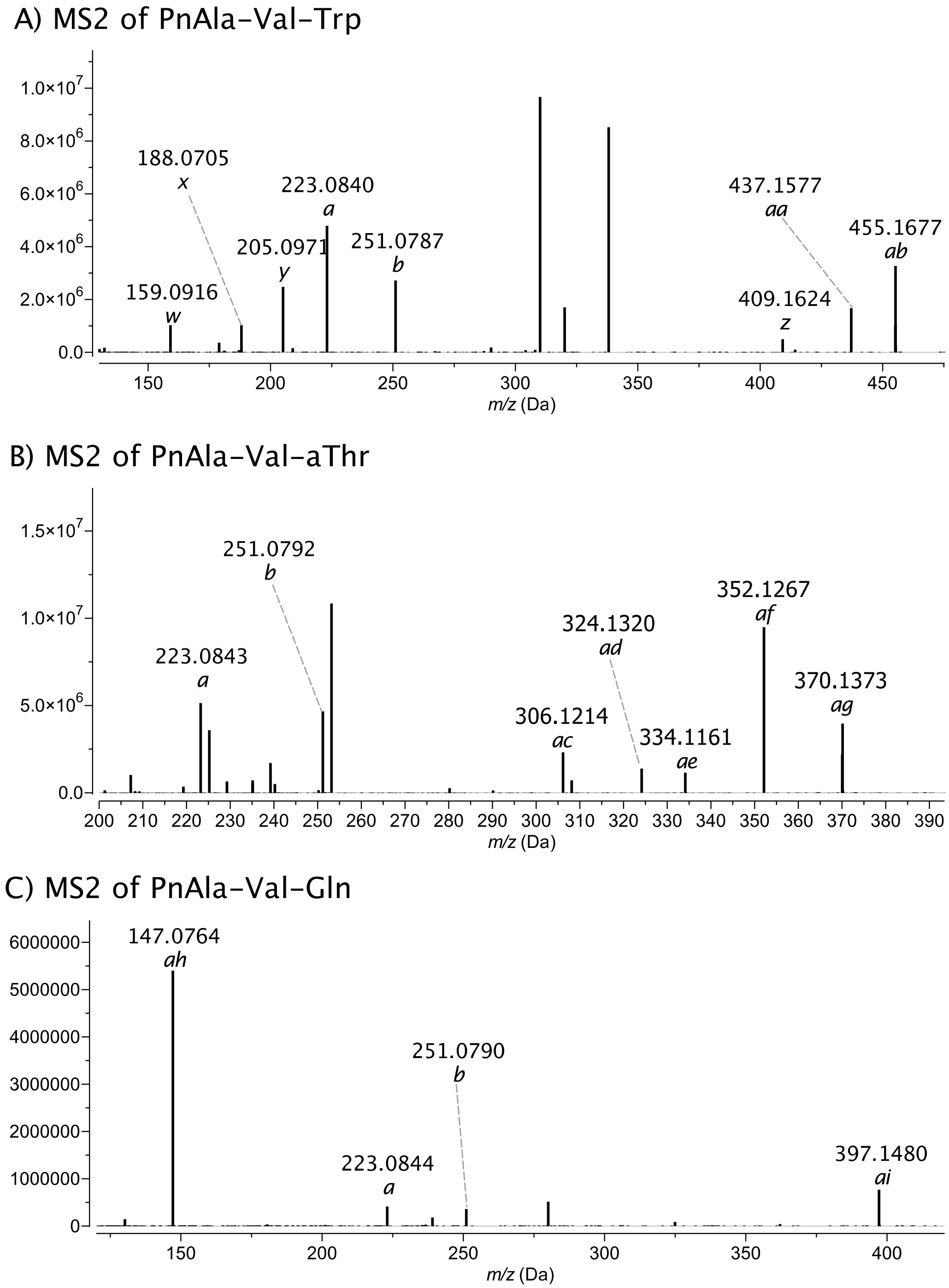

Figure S118. LC-MS/MS fragmentation of PnAla-Val-Trp, PnAla-Val-aThr, and PnAla-Val-Gln
Refer to Table S60 for fragment ion formula, structures, and mass error

A) MS2 of PnAla-Val-Pro

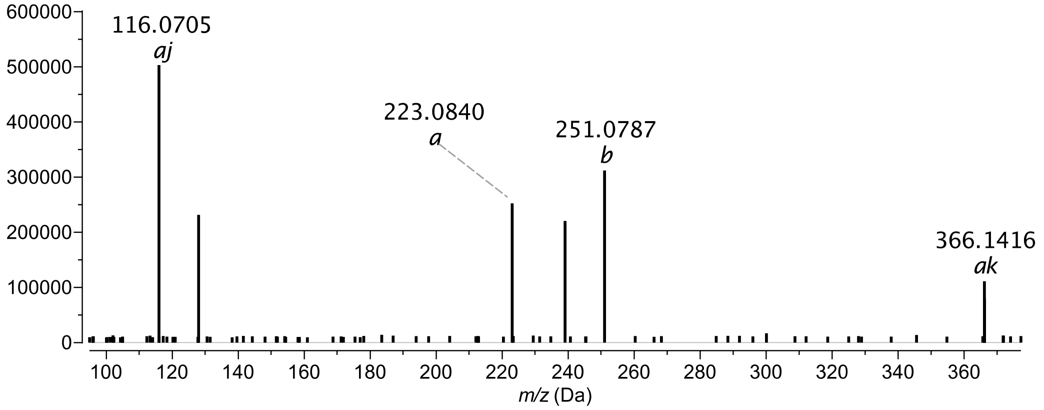

Figure S119. LC-MS/MS fragmentation of PnAla-Val-Pro

Refer to Table S61 for fragment ion formula, structures, and mass error

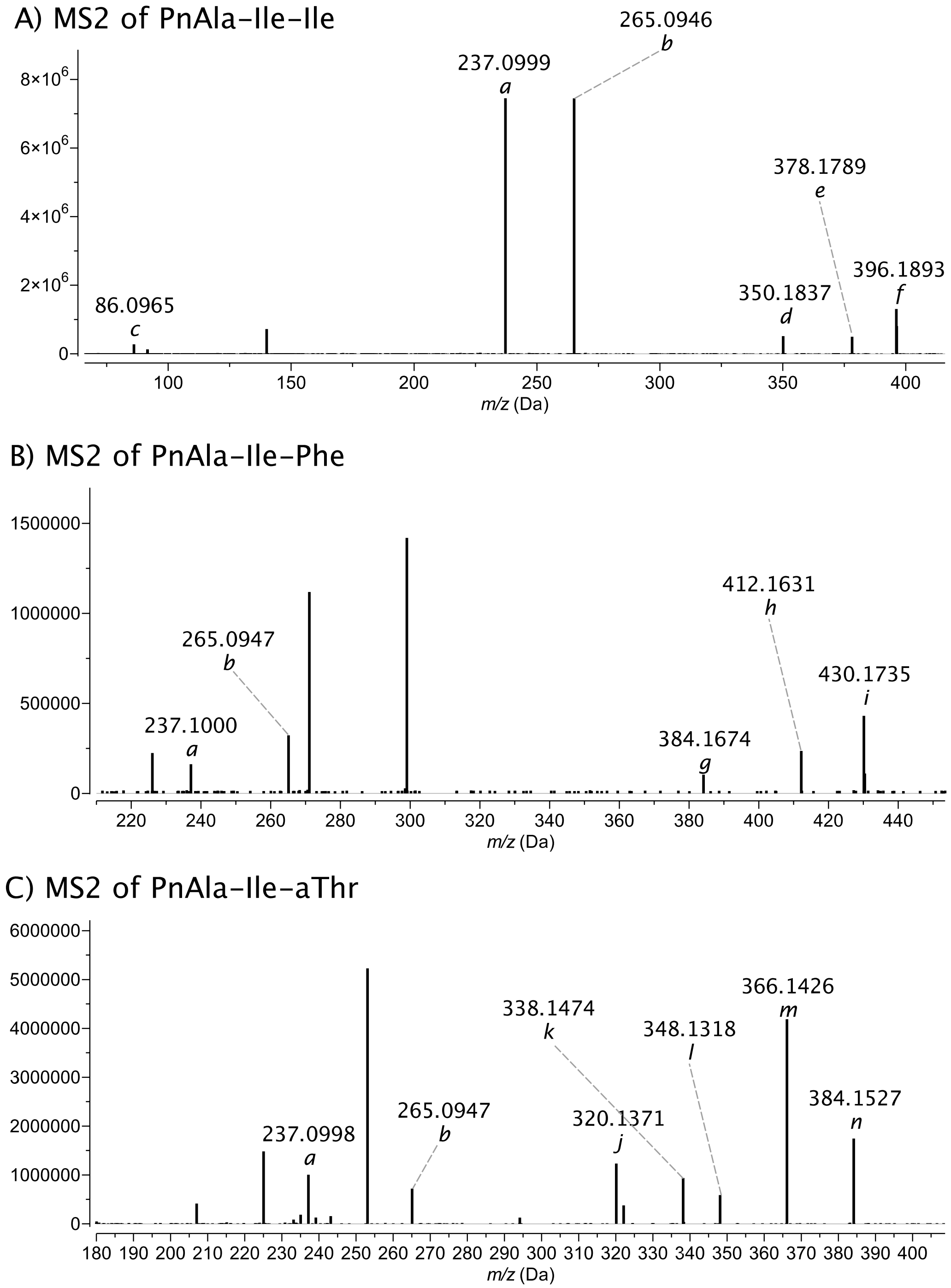

Figure S120. LC-MS/MS fragmentation of PnAla-Ile-Ile, PnAla-Ile-Phe, and PnAla-Ile-aThr

Refer to Table S62 for fragment ion formula, structures, and mass error

A) MS2 of PnAla-Leu-Leu

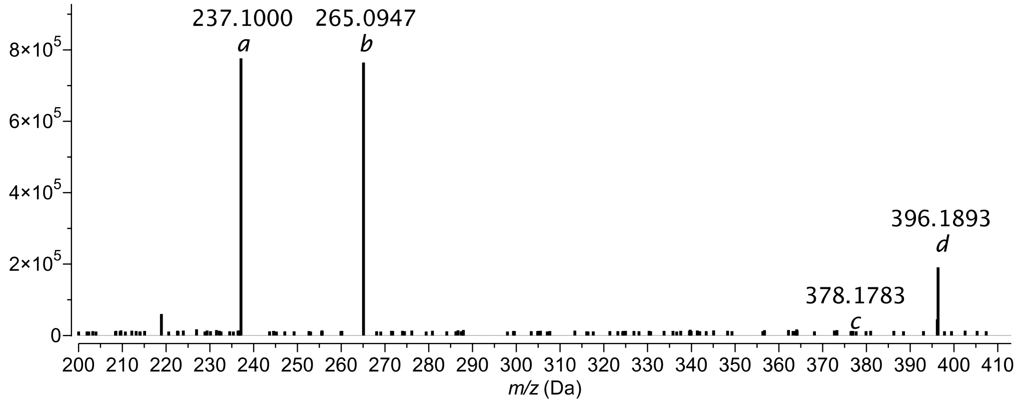

Figure S121. LC-MS/MS fragmentation of PnAla-Leu-Leu

Refer to Table S63 for fragment ion formula, structures, and mass error

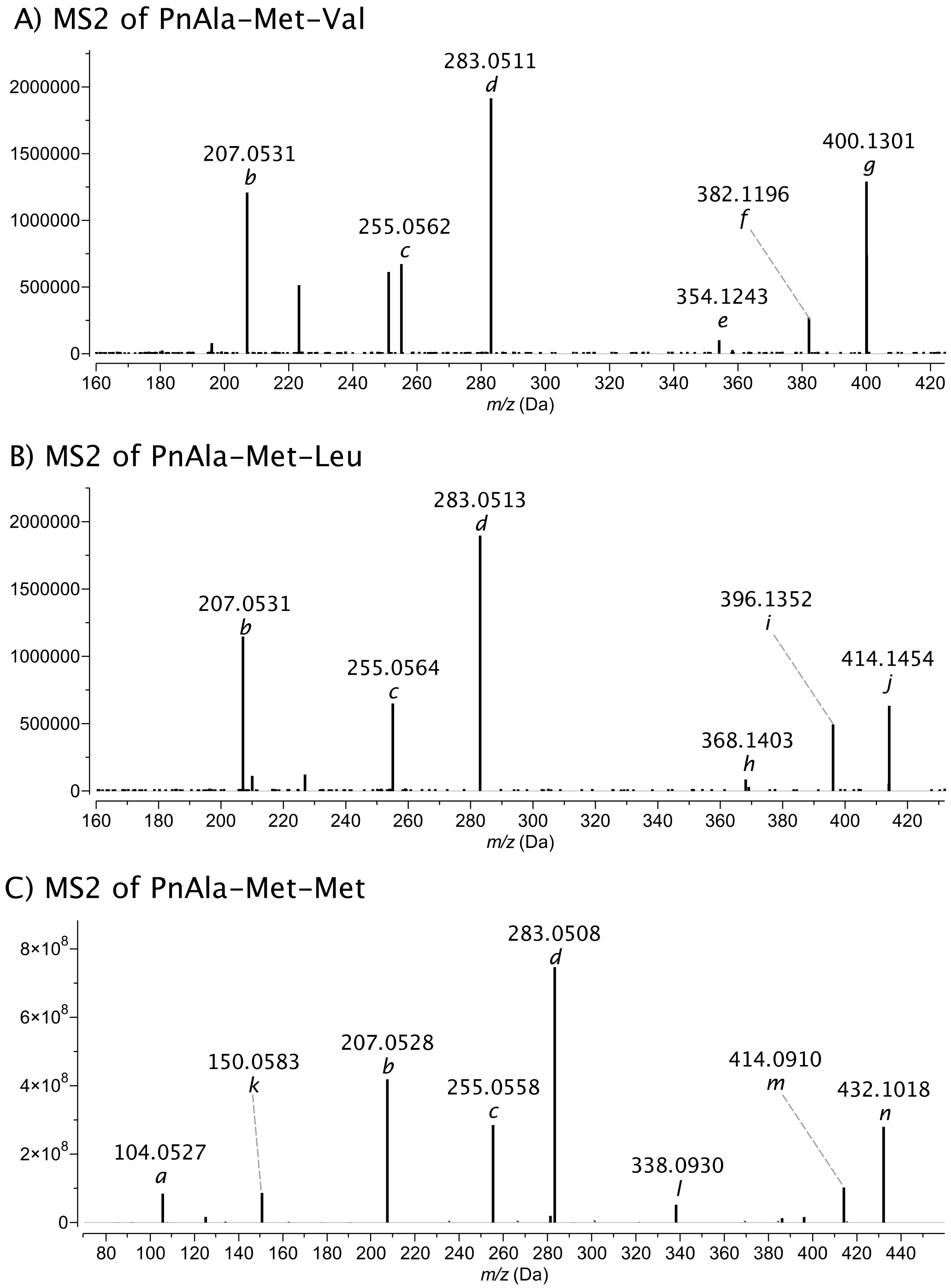

Figure S122. LC-MS/MS fragmentation of PnAla-Met-Val, PnAla-Met-Leu, and PnAla-Met-Met
Refer to Table S64 for fragment ion formula, structures, and mass error

A) MS2 of PnAla-Met-Trp

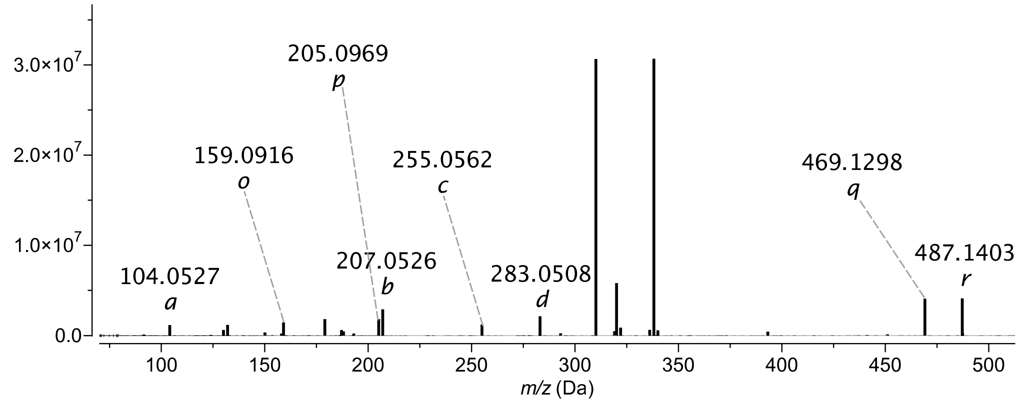

Figure S123. LC-MS/MS fragmentation of PnAla-Met-Trp

Refer to Table S65 for fragment ion formula, structures, and mass error

A) MS2 of PnAla-Phe-Val

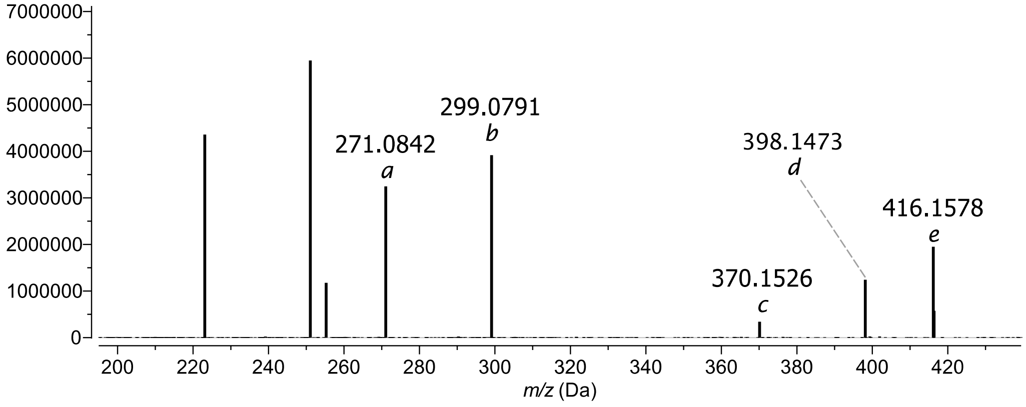

B) MS2 of PnAla-Phe-Ile

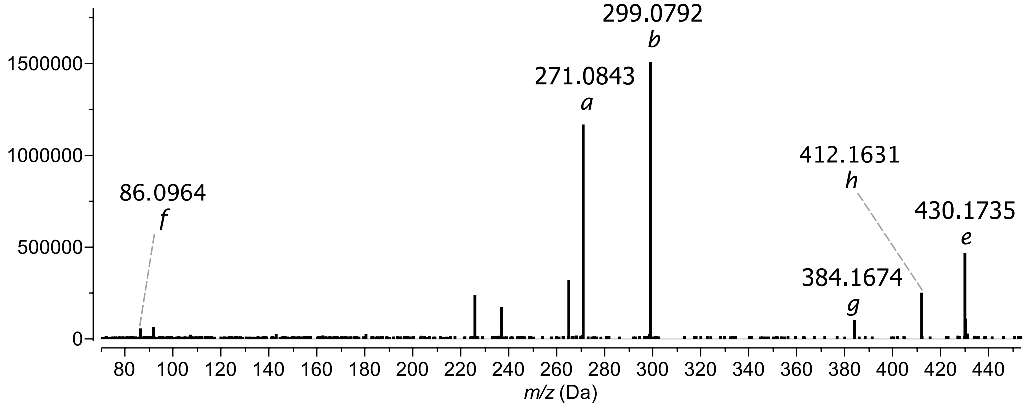

C) MS2 of PnAla-Phe-Leu

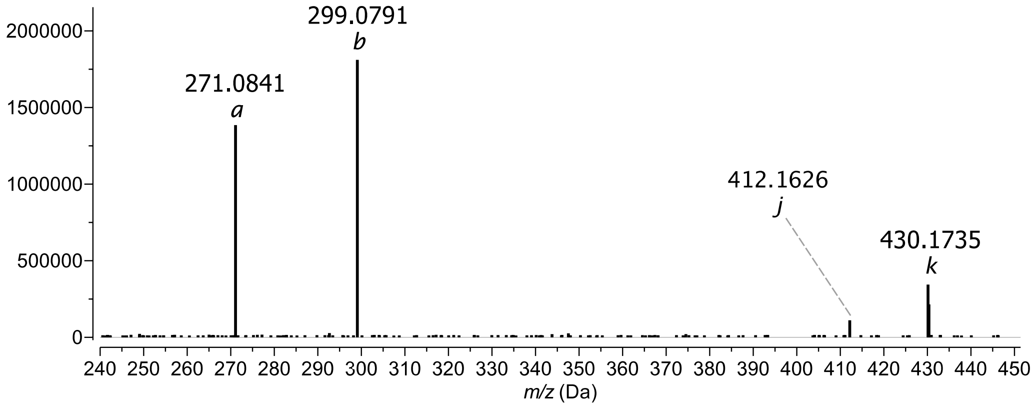

Figure S124. LC-MS/MS fragmentation of PnAla-Phe-Val, PnAla-Phe-Ile, and PnAla-Phe-Leu
Refer to Table S66 for fragment ion formula, structures, and mass error

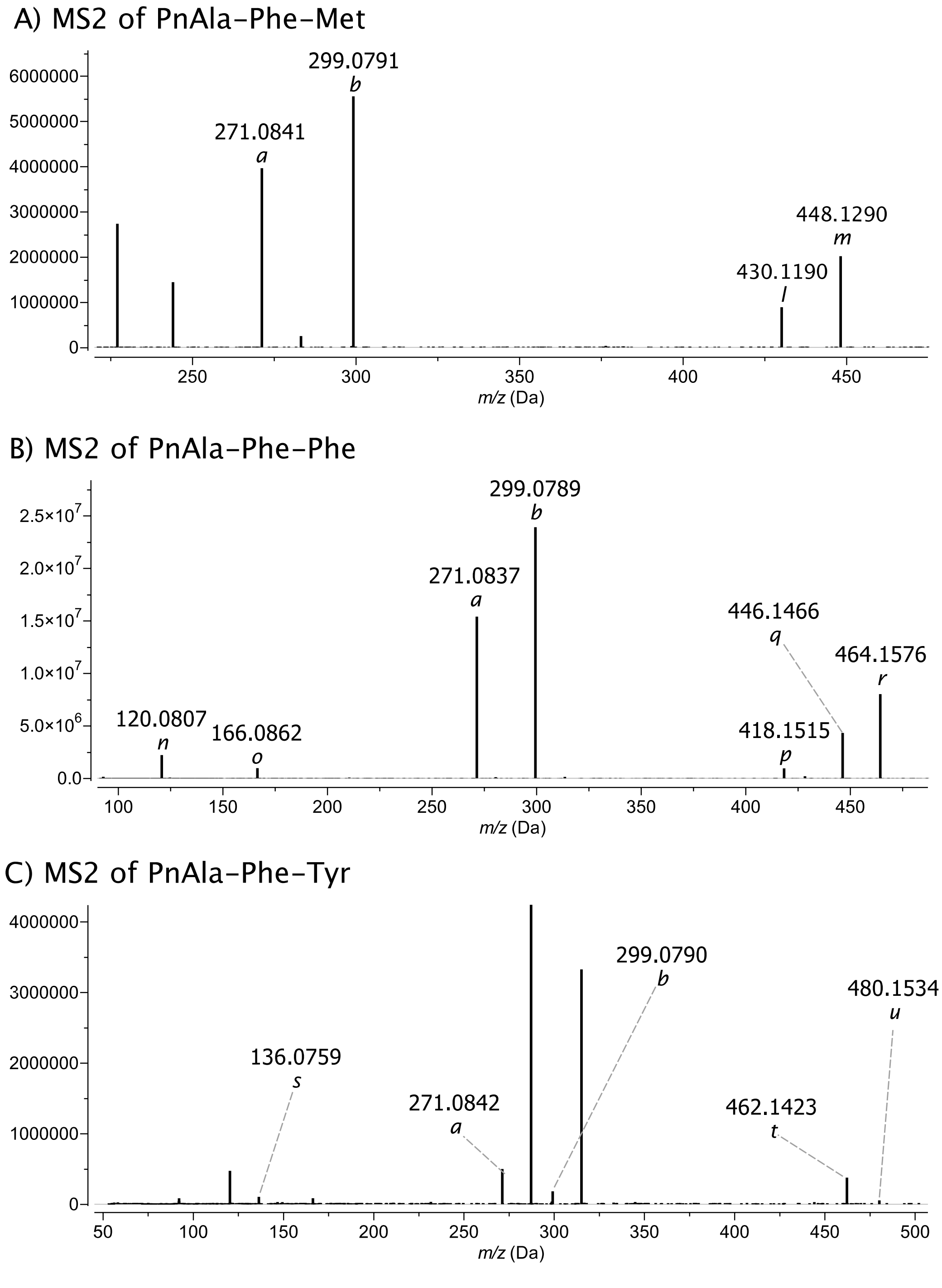

Figure S125. LC-MS/MS fragmentation of PnAla-Phe-Met, PnAla-Phe-Phe, and PnAla-Phe-Tyr

Refer to Table S67 for fragment ion formula, structures, and mass error

A) MS2 of PnAla-Phe-Trp

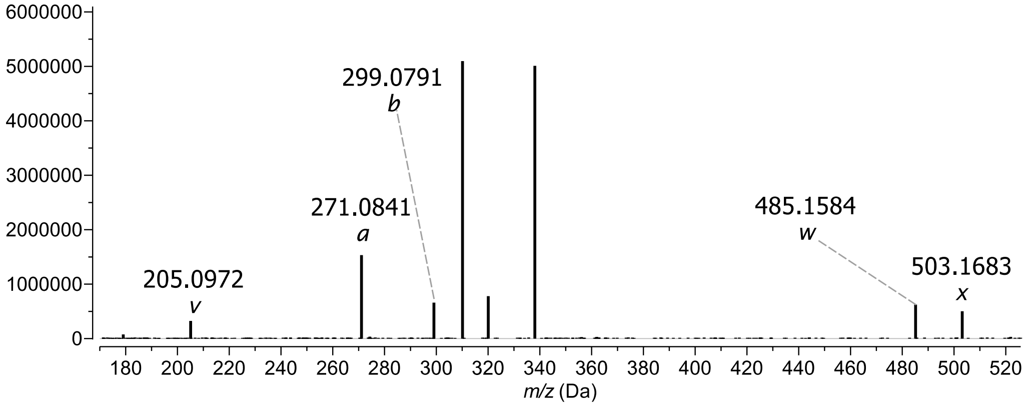

B) MS2 of PnAla-Phe-Thr

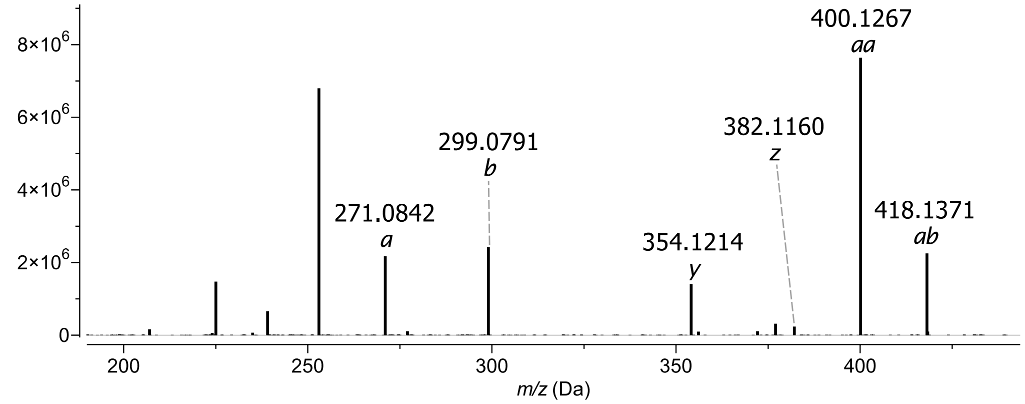

C) MS2 of PnAla-Phe-aThr

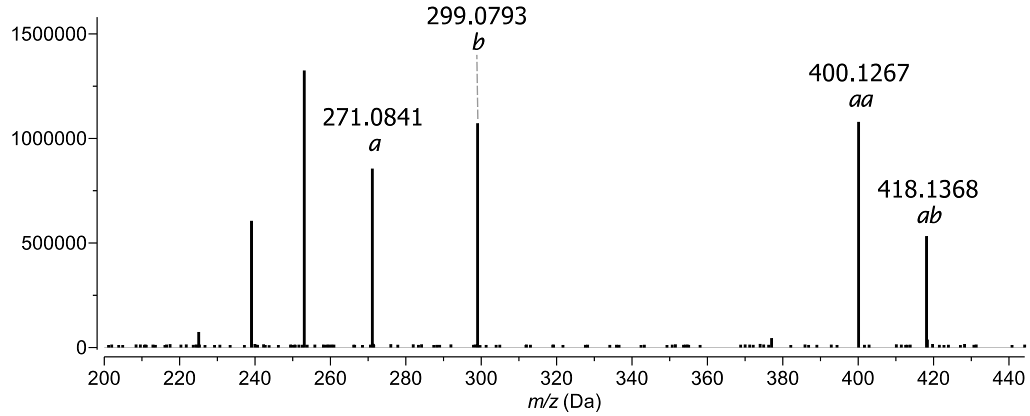

Figure S126. LC-MS/MS fragmentation of PnAla-Phe-Trp, PnAla-Phe-Thr, and PnAla-Phe-aThr

Refer to Table S68 for fragment ion formula, structures, and mass error

A) MS2 of PnAla-Tyr-Val

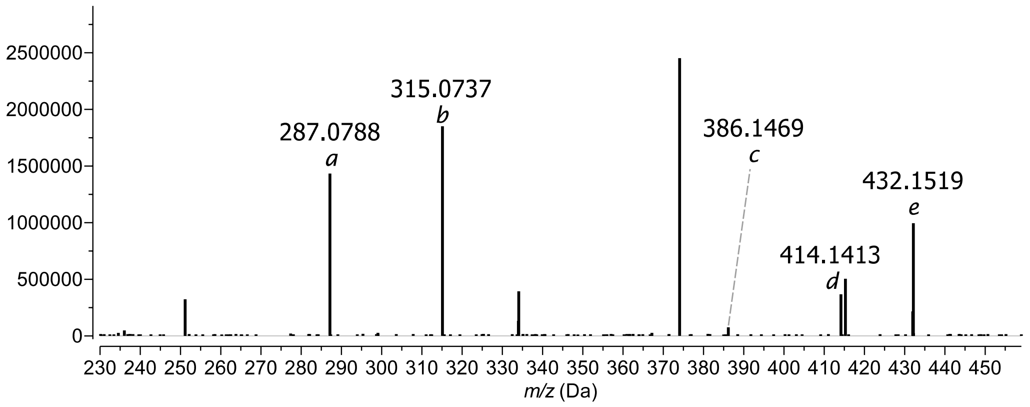

B) MS2 of PnAla-Tyr-Leu

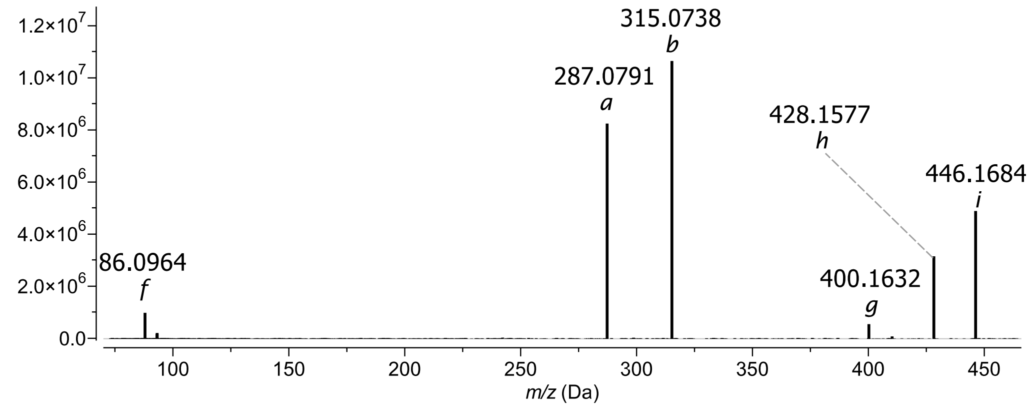

C) MS2 of PnAla-Tyr-Met

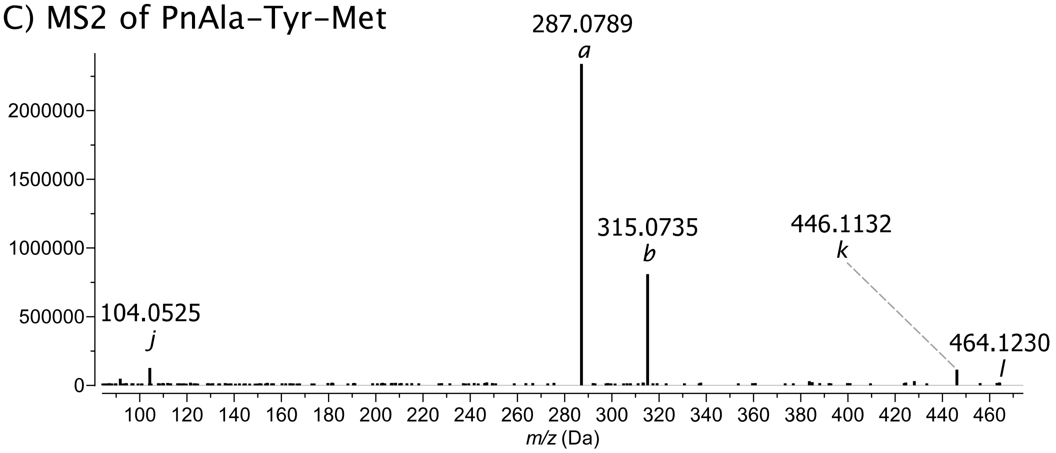

Figure S127. LC-MS/MS fragmentation of PnAla-Tyr-Val, PnAla-Tyr-Leu, and PnAla-Tyr-Met

Refer to Table S69 for fragment ion formula, structures, and mass error

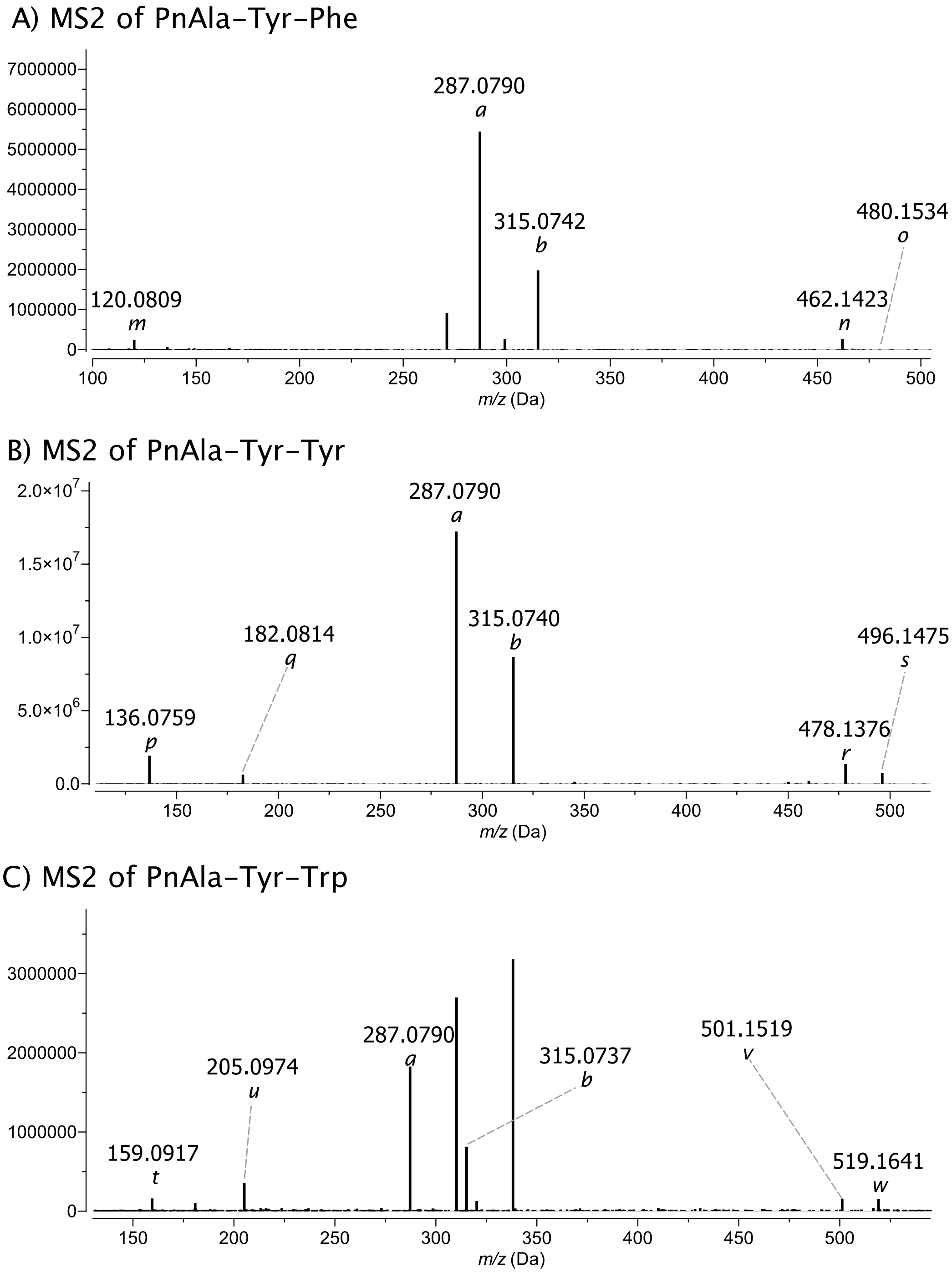

Figure S128. LC-MS/MS fragmentation of PnAla-Tyr-Phe, PnAla-Tyr-Tyr, and PnAla-Tyr-Trp

Refer to Table S70 for fragment ion formula, structures, and mass error

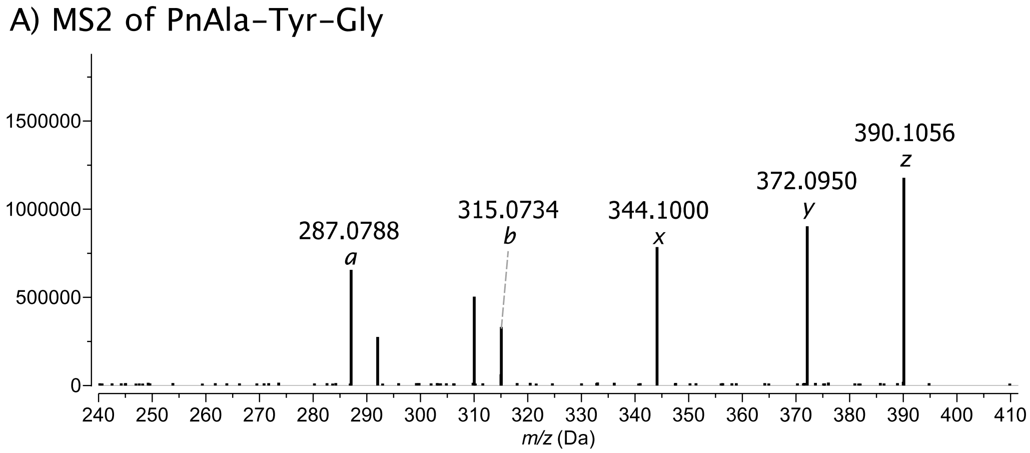

Figure S129. LC-MS/MS fragmentation of PnAla-Tyr-Gly

Refer to Table S71 for fragment ion formula, structures, and mass error

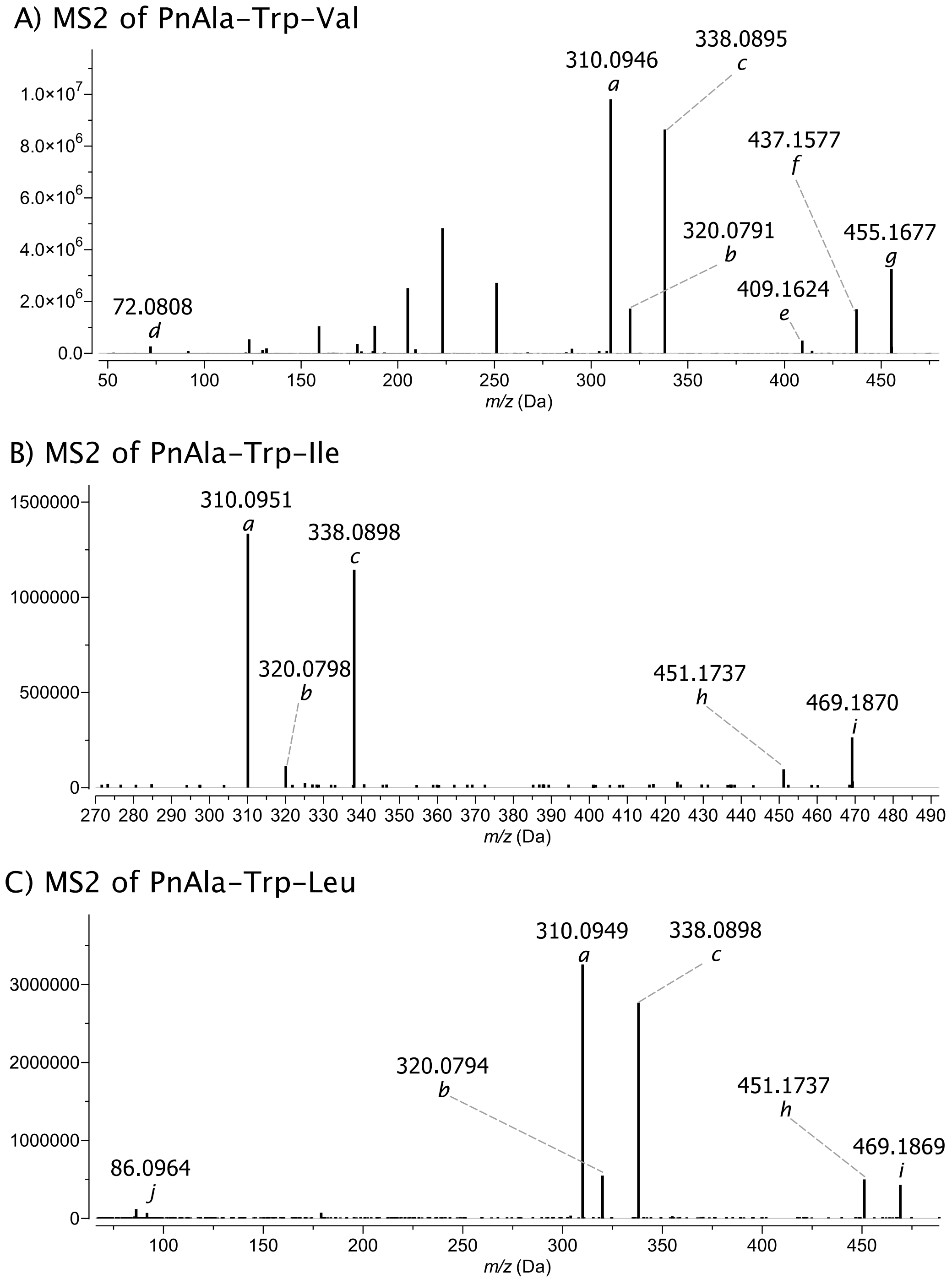

Figure S130. LC-MS/MS fragmentation of PnAla-Trp-Val, PnAla-Trp-Ile, and PnAla-Trp-Leu
Refer to Table S72 for fragment ion formula, structures, and mass error

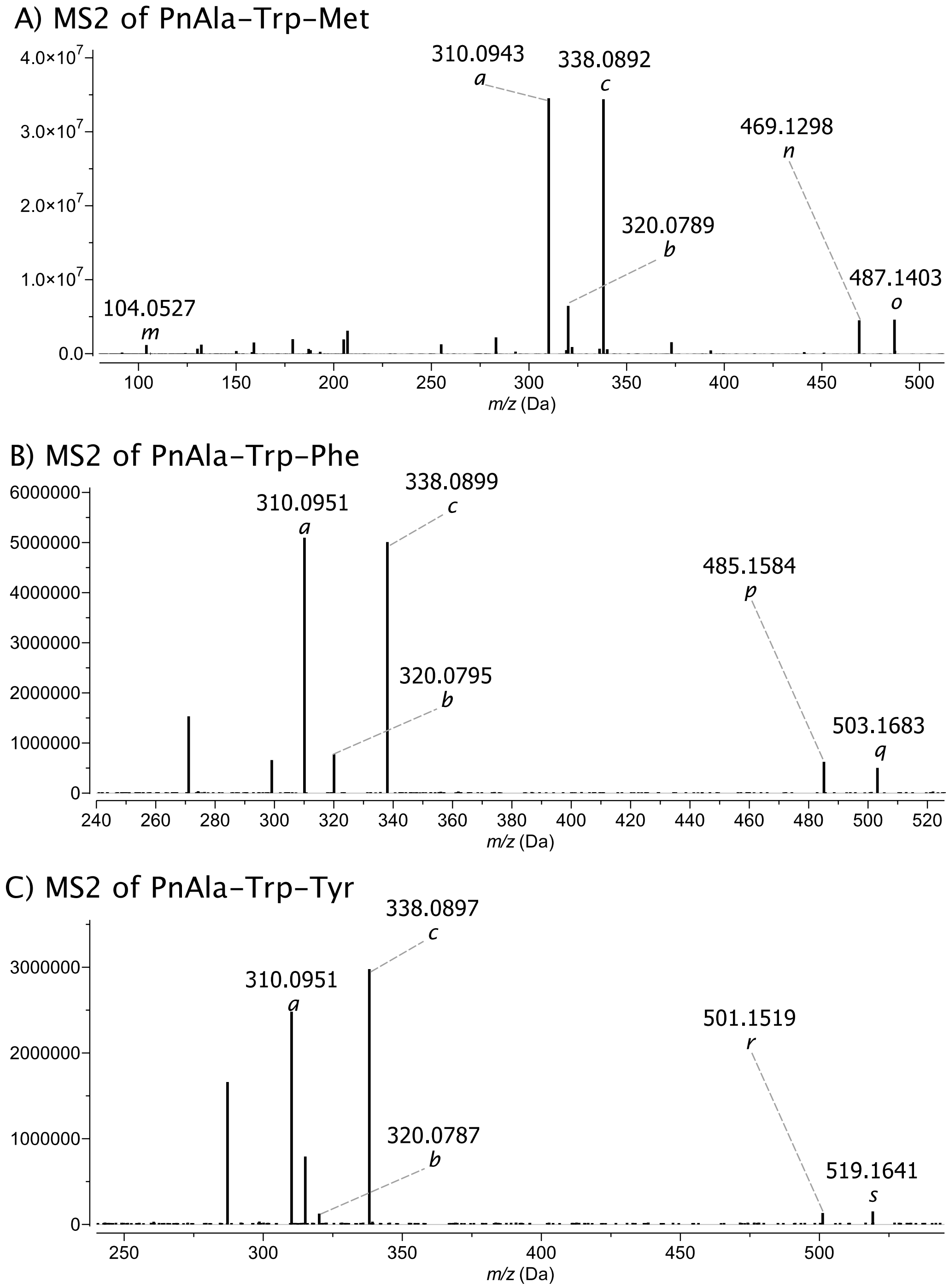

Figure S131. LC-MS/MS fragmentation of PnAla-Trp-Met, PnAla-Trp-Phe, and PnAla-Trp-Tyr
Refer to Table S73 for fragment ion formula, structures, and mass error

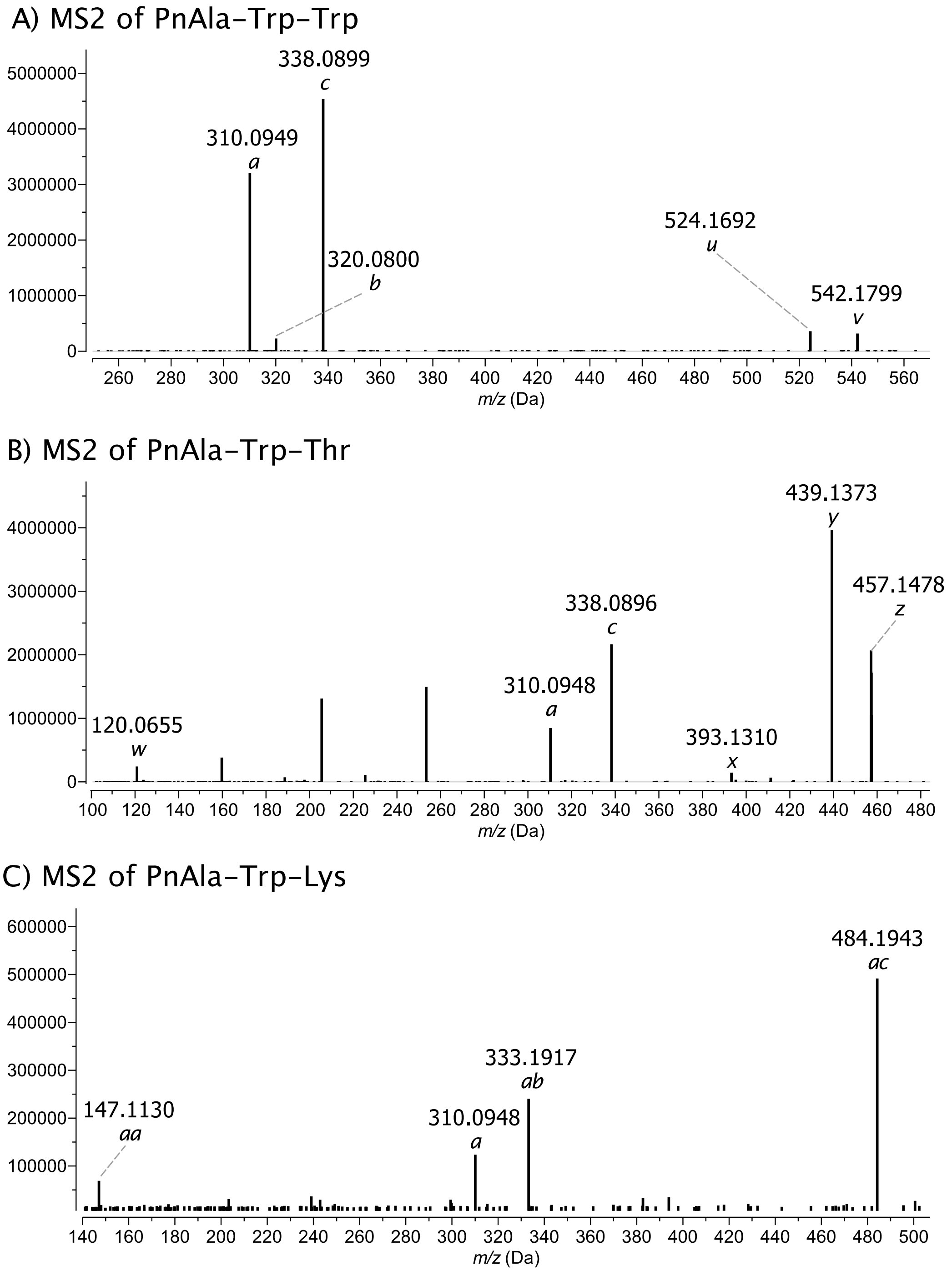

Figure S132. LC-MS/MS fragmentation of PnAla-Trp-Trp, PnAla-Trp-Thr, and PnAla-Trp-Lys

Refer to Table S74 for fragment ion formula, structures, and mass error

A) MS2 of PnAla-Trp-Gly

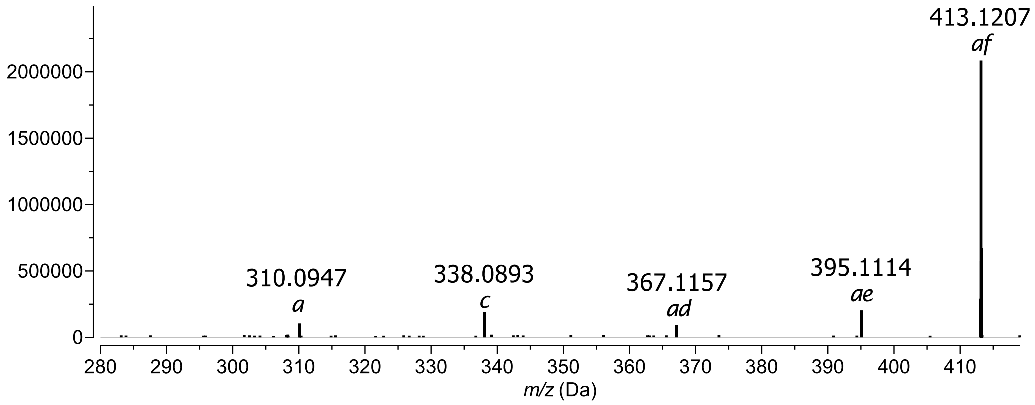

Figure S133. LC-MS/MS fragmentation of PnAla-Trp-Gly

Refer to Table S75 for fragment ion formula, structures, and mass error

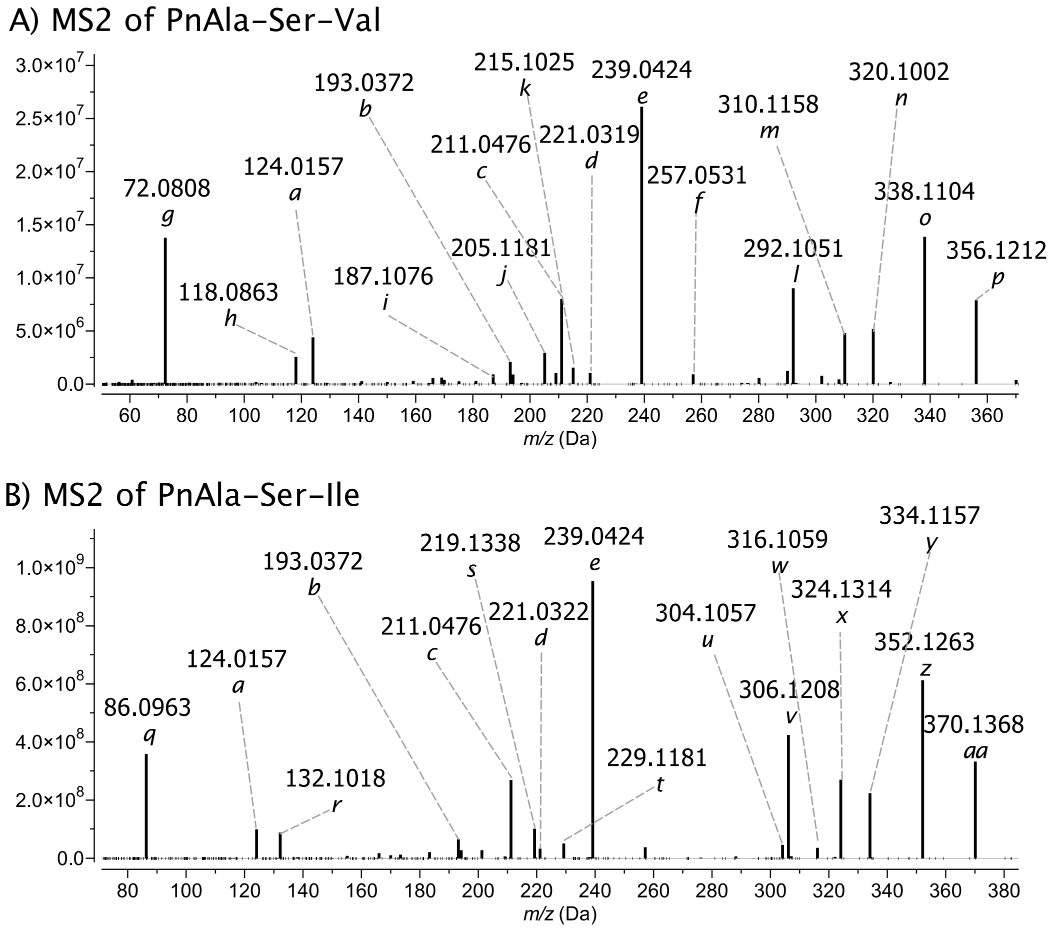

Figure S134. LC-MS/MS fragmentation of PnAla-Ser-Val and PnAla-Ser-Ile

Refer to Table S76 for fragment ion formula, structures, and mass error

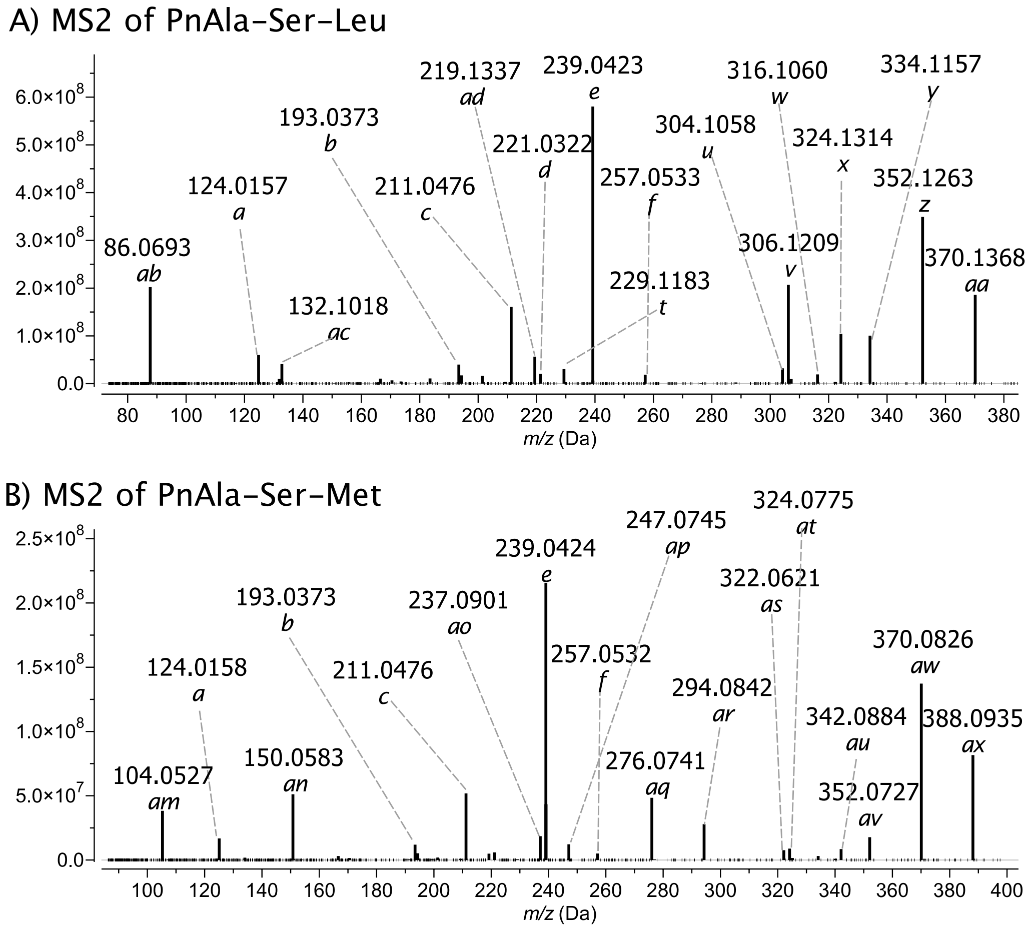

Figure S135. LC-MS/MS fragmentation of PnAla-Ser-Leu and PnAla-Ser-Met

Refer to Table S77 for fragment ion formula, structures, and mass error

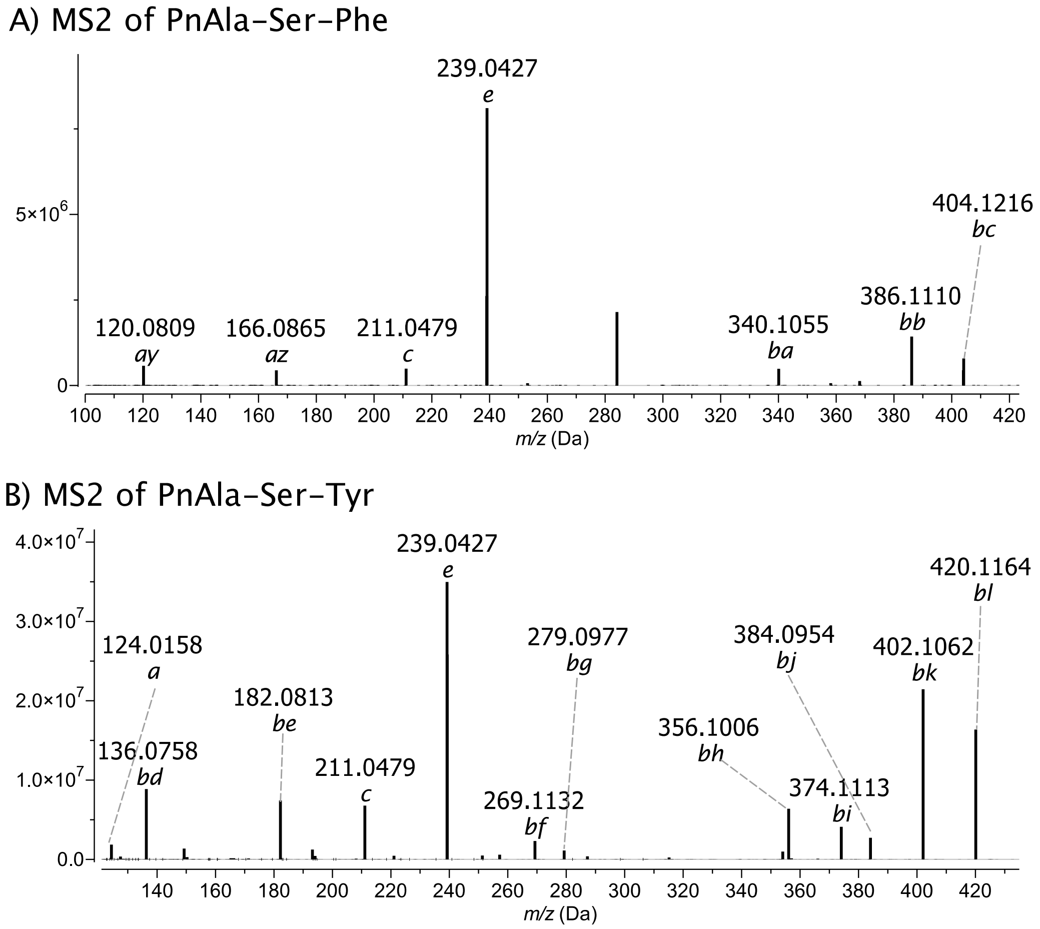

Figure S136. LC-MS/MS fragmentation of PnAla-Ser-Phe and PnAla-Ser-Tyr

Refer to Table S78 for fragment ion formula, structures, and mass error

A) MS2 of PnAla-Ser-Ser

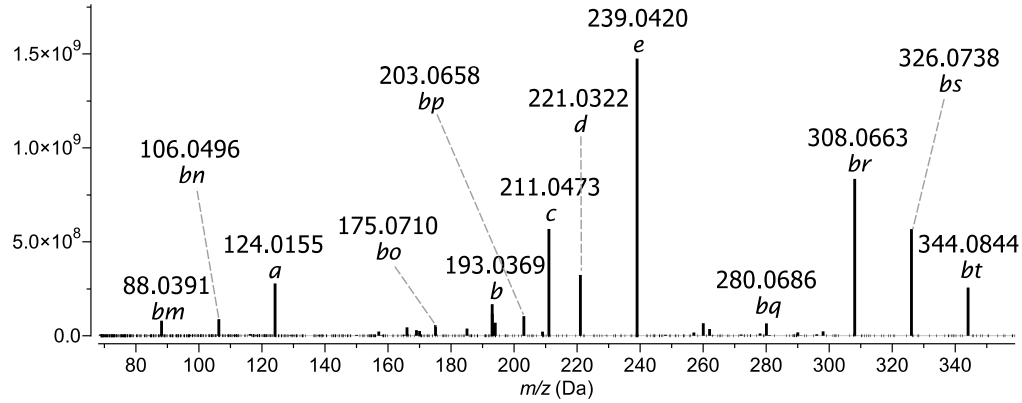

B) MS2 of PnAla-Ser-Thr

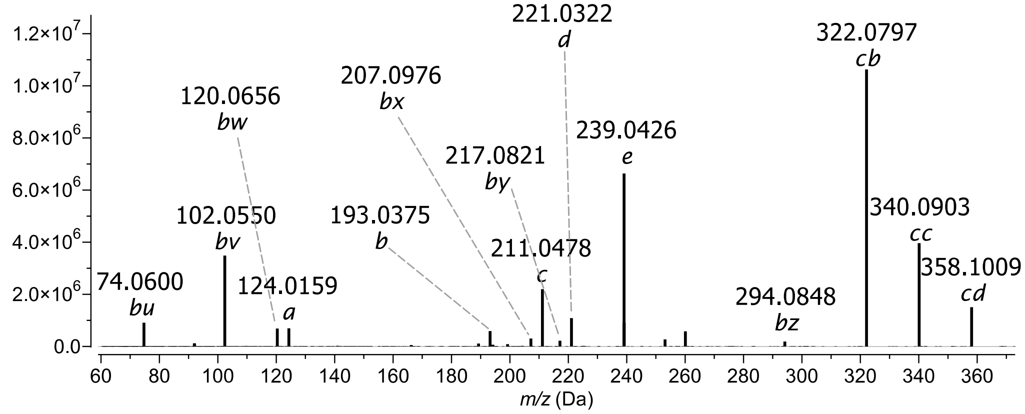

C) MS2 of PnAla-Ser-aThr

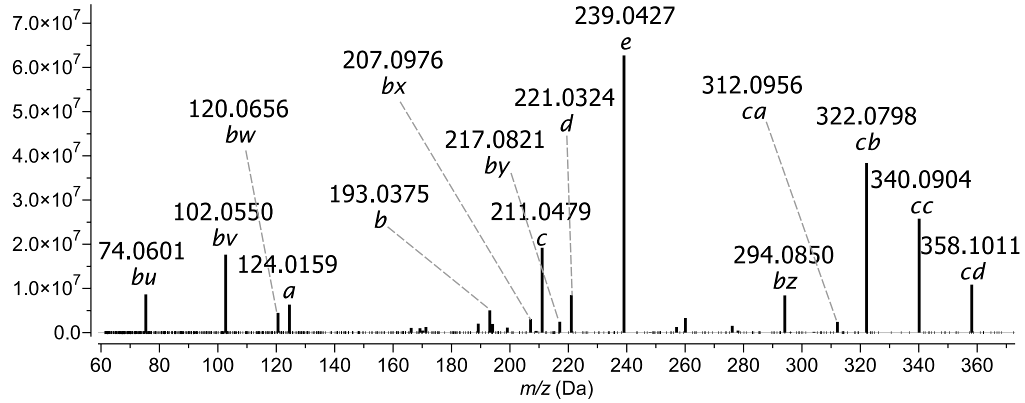

Figure S137. LC-MS/MS fragmentation of PnAla-Ser-Ser, PnAla-Ser-Thr, and PnAla-Ser-aThr

Refer to Table S79 for fragment ion formula, structures, and mass error

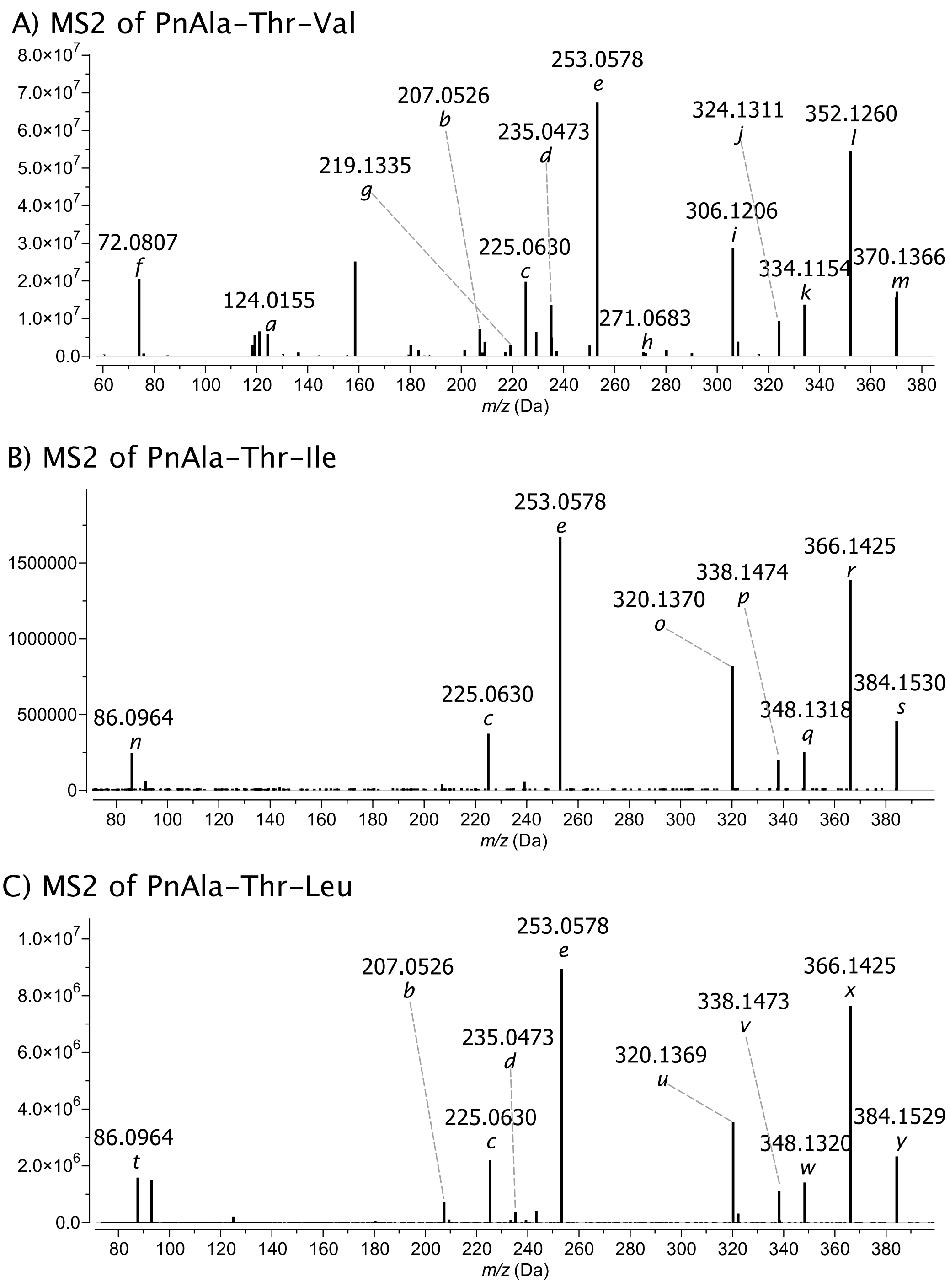

Figure S138. LC-MS/MS fragmentation of PnAla-Thr-Val, PnAla-Thr-Ile, and PnAla-Thr-Leu

Refer to Table S80 for fragment ion formula, structures, and mass error

A) MS2 of PnAla-Thr-Phe

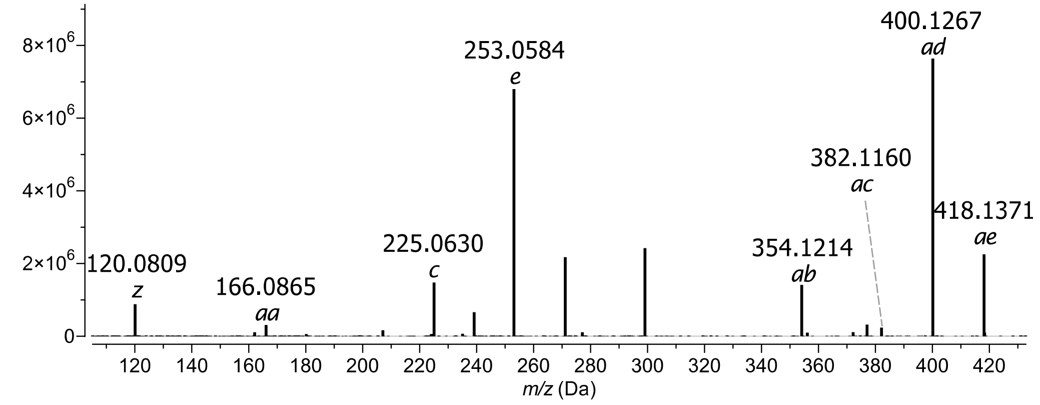

B) MS2 of PnAla-Thr-Trp

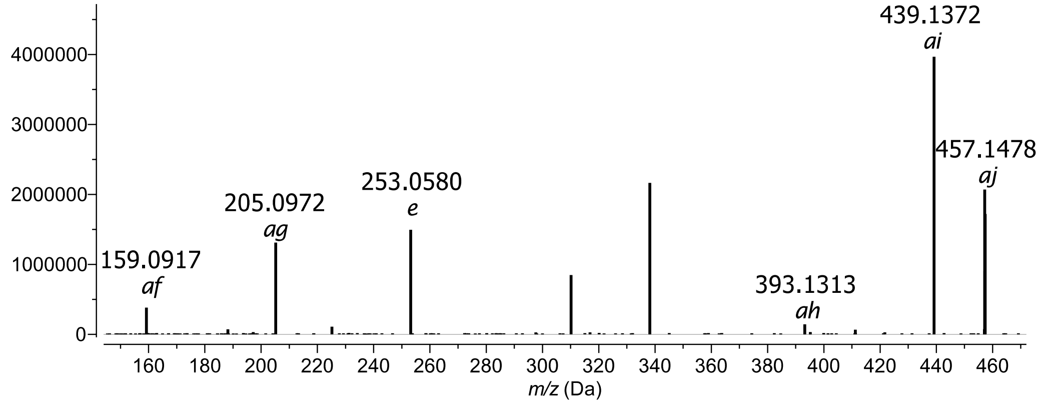

Figure S139. LC-MS/MS fragmentation of PnAla-Thr-Phe and PnAla-Thr-Trp

Refer to Table S81 for fragment ion formula, structures, and mass error

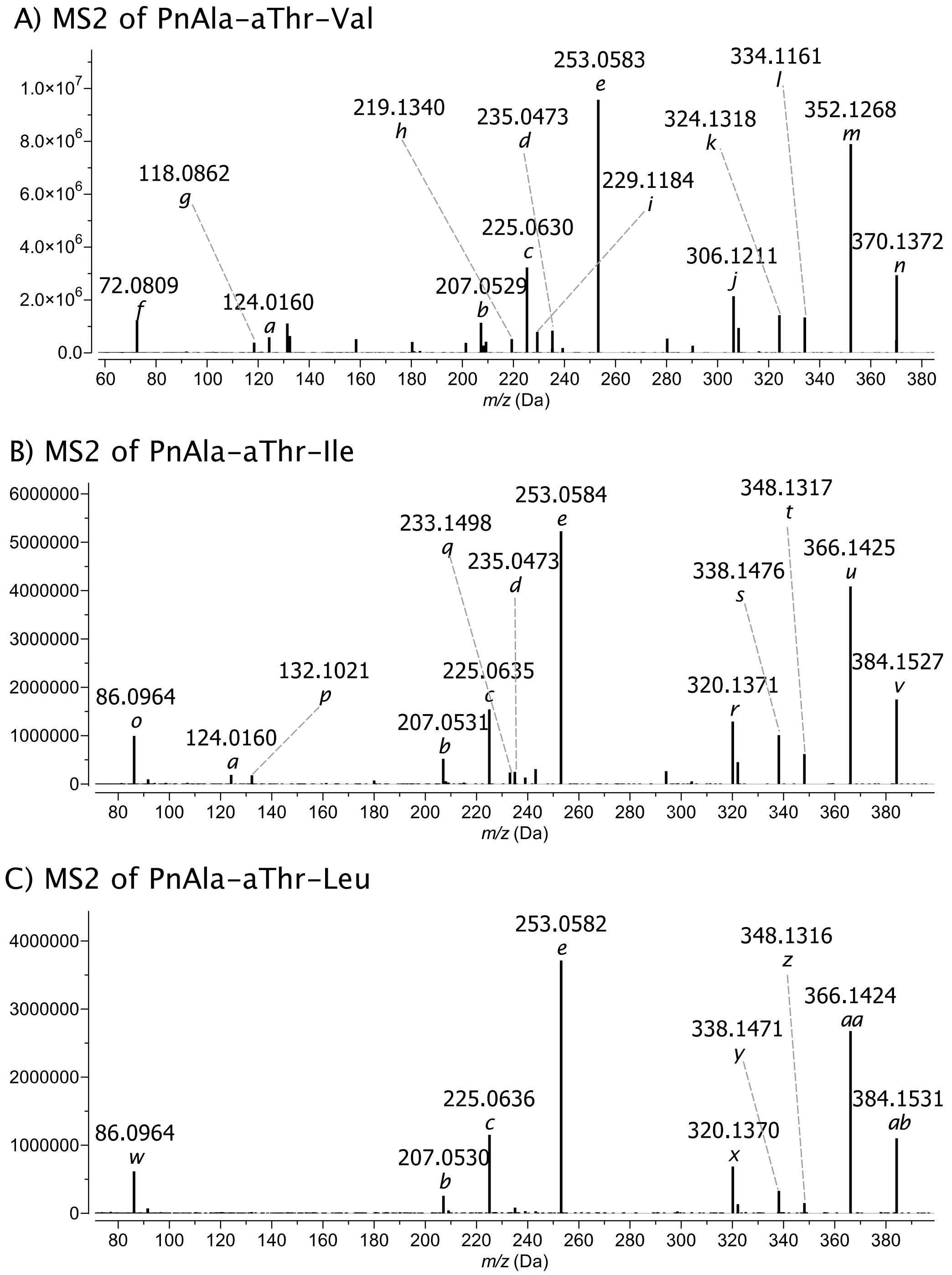

Figure S140. LC-MS/MS fragmentation of PnAla-aThr-Val, PnAla-aThr-Ile, and PnAla-aThr-Leu

Refer to Table S82 for fragment ion formula, structures, and mass error

A) MS2 of PnAla-aThr-Met

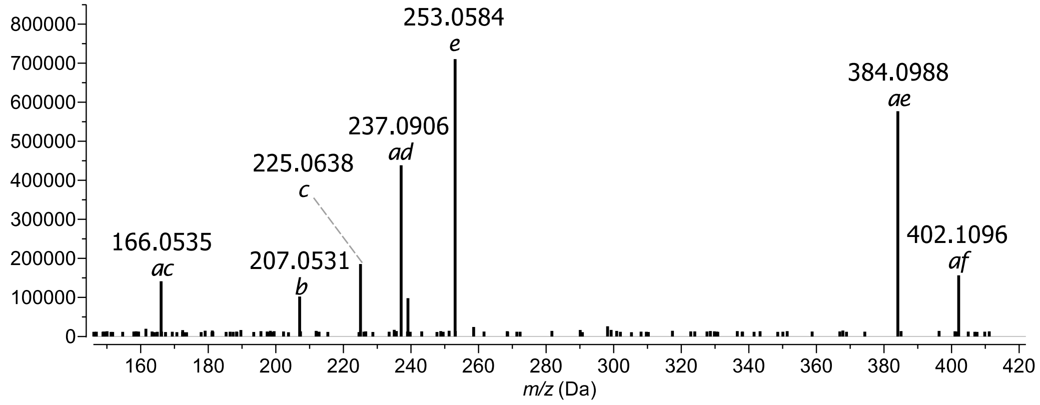

B) MS2 of PnAla-aThr-Phe

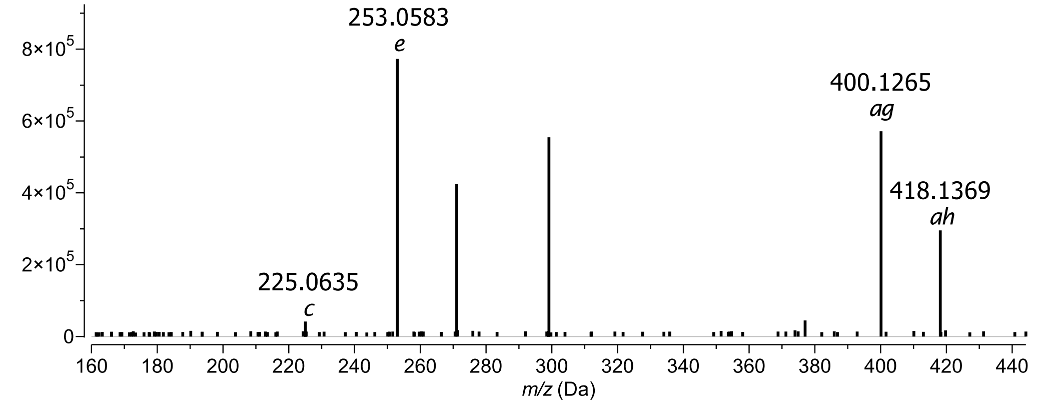

C) MS2 of PnAla-aThr-aThr

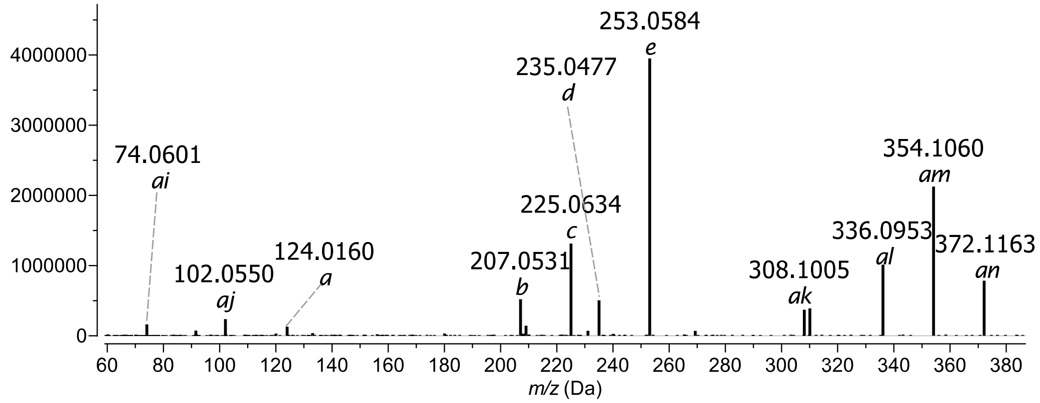

Figure S141. LC-MS/MS fragmentation of PnAla-aThr-Met, PnAla-aThr-Phe, and PnAla-aThr-aThr

Refer to Table S83 for fragment ion formula, structures, and mass error

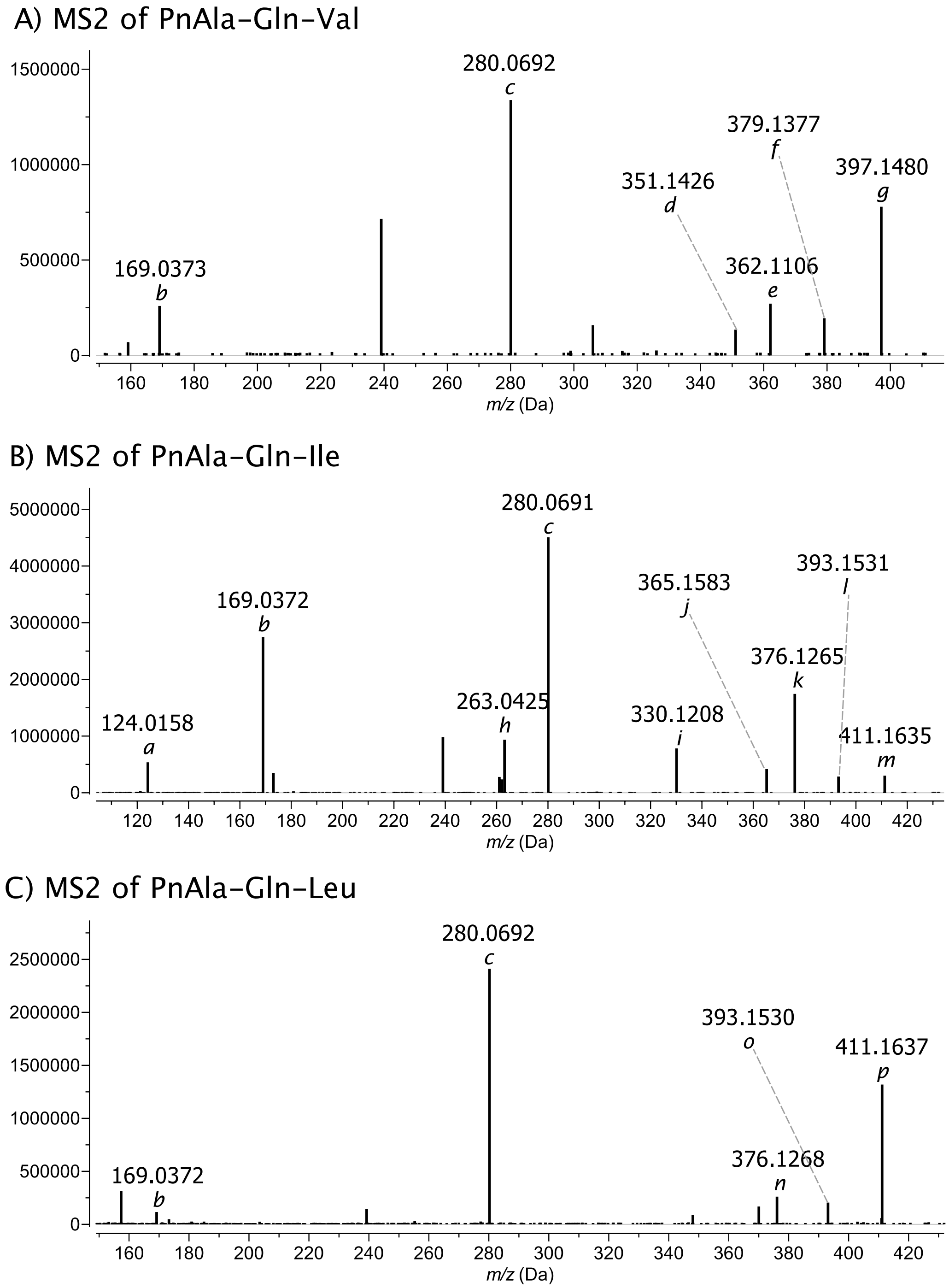

Figure S142. LC-MS/MS fragmentation of PnAla-Gln-Val, PnAla-Gln-Ile, and PnAla-Gln-Leu

Refer to Table S84 for fragment ion formula, structures, and mass error

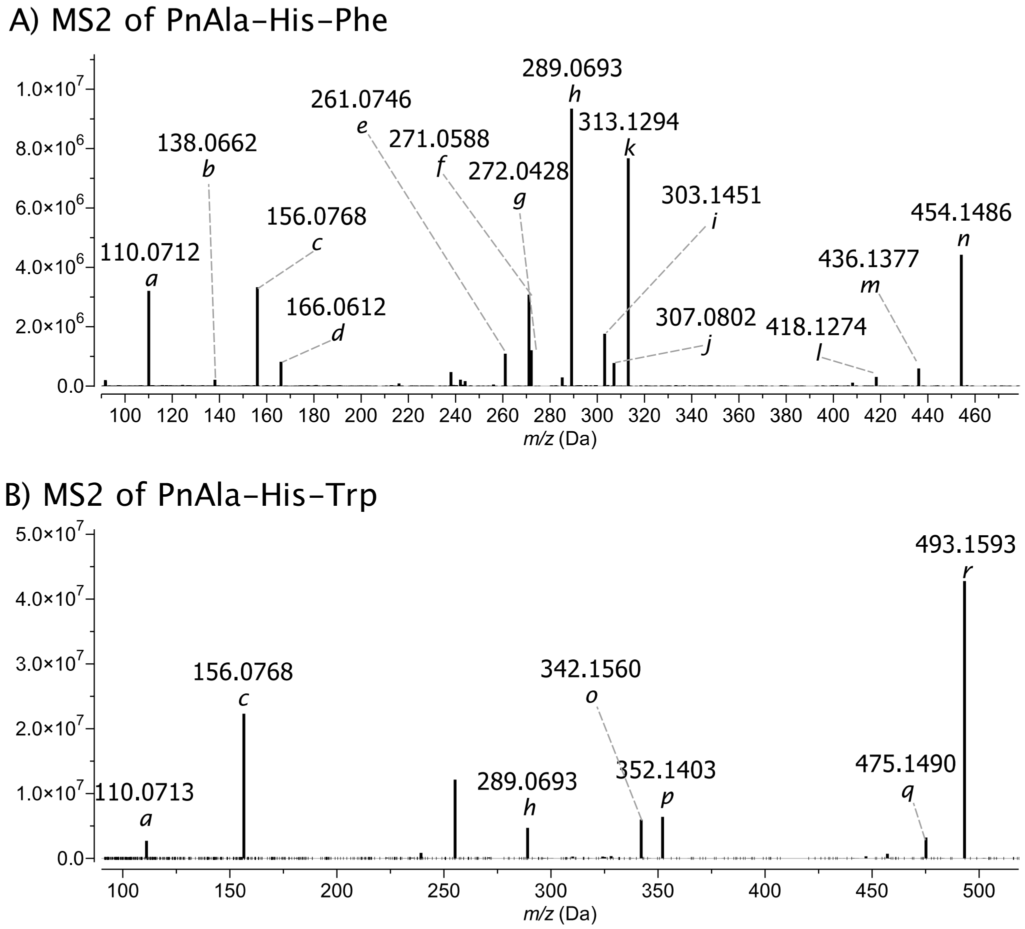

Figure S143. LC-MS/MS fragmentation of PnAla-His-Phe and PnAla-His-Trp

Refer to Table S85 for fragment ion formula, structures, and mass error

A) MS2 of PnAla-Gly-Val

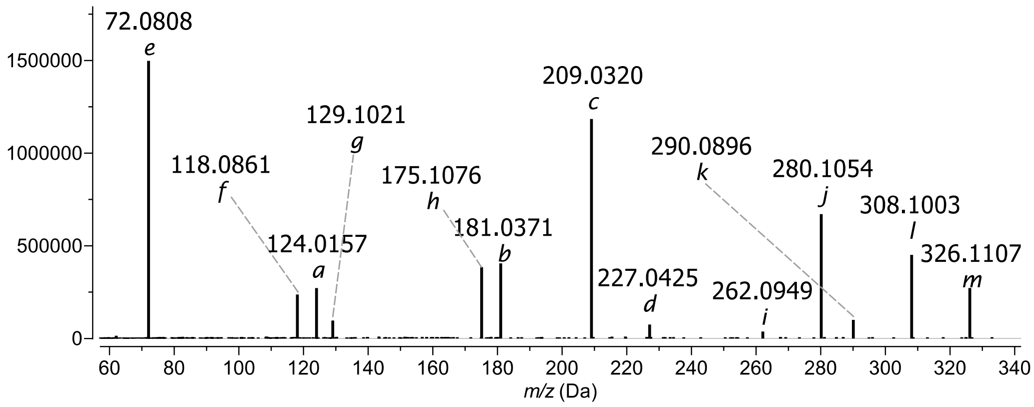

B) MS2 of PnAla-Gly-Ile

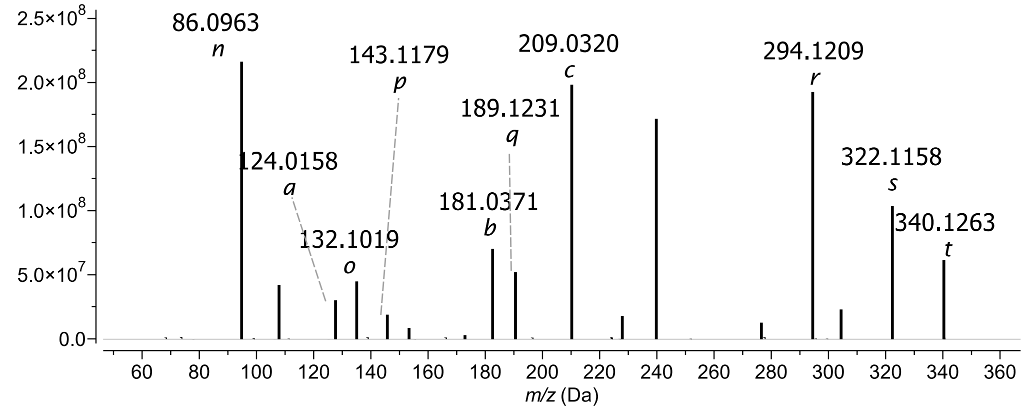

C) MS2 of PnAla-Gly-Leu

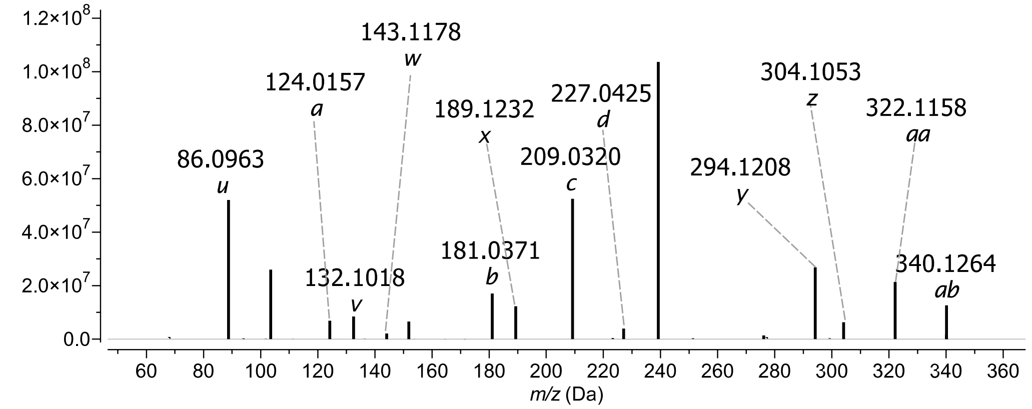

Figure S144. LC-MS/MS fragmentation of PnAla-Gly-Val, PnAla-Gly-Ile, and PnAla-Gly-Leu

Refer to Table S86 for fragment ion formula, structures, and mass error

A) MS2 of PnAla-Gly-Met

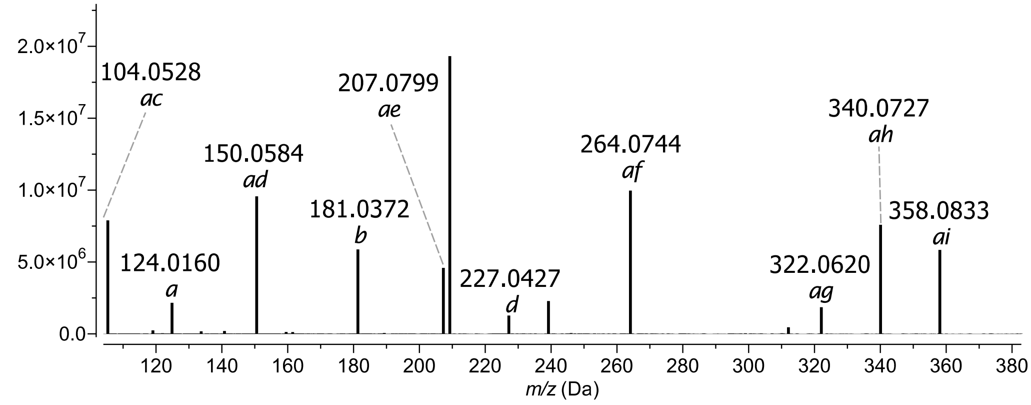

B) MS2 of PnAla-Gly-Phe

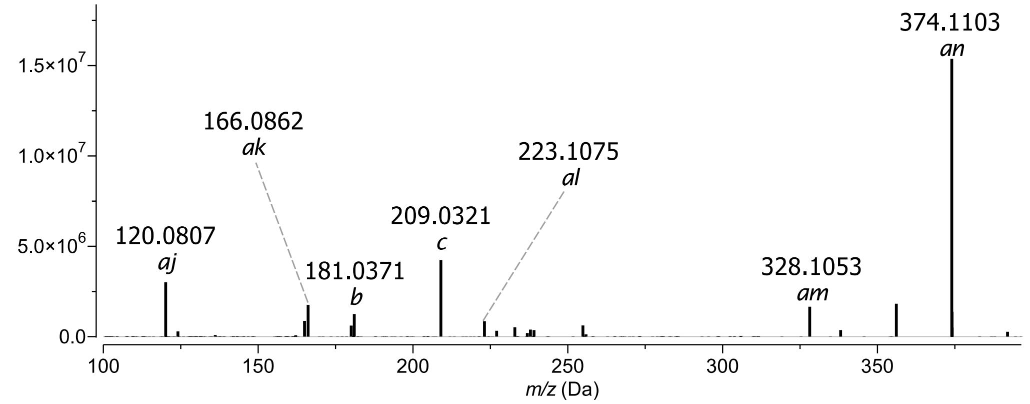

C) MS2 of PnAla-Gly-Tyr

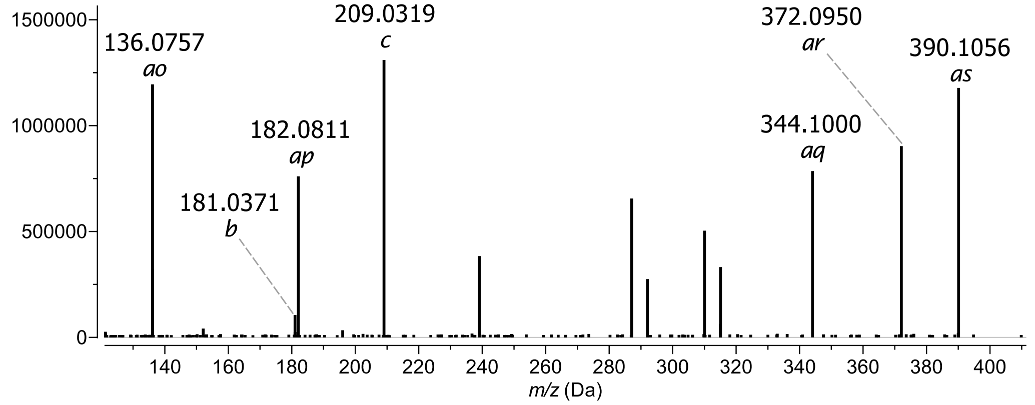

Figure S145. LC-MS/MS fragmentation of PnAla-Gly-Met, PnAla-Gly-Phe, and PnAla-Gly-Tyr

Refer to Table S87 for fragment ion formula, structures, and mass error

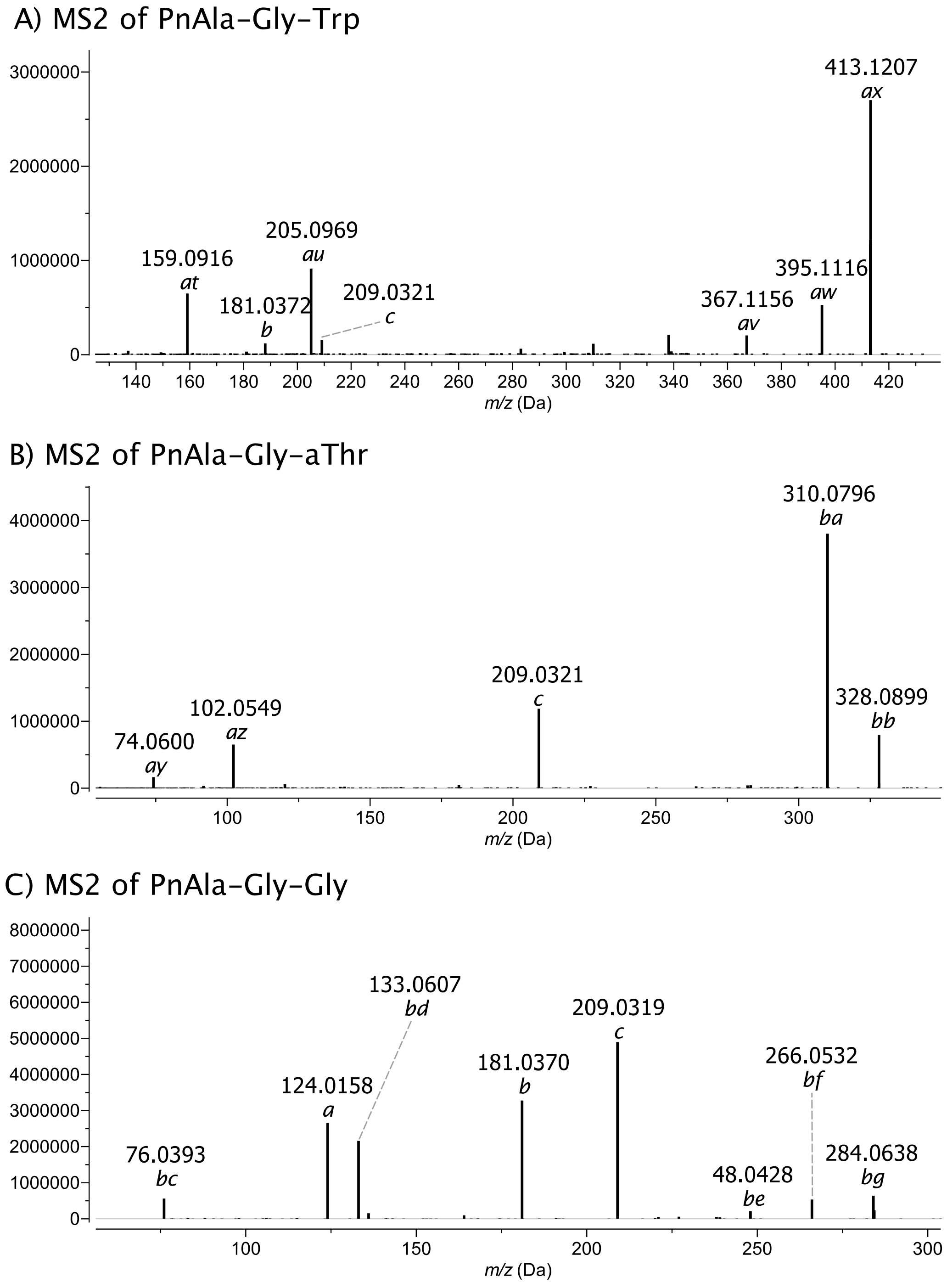

Figure S146. LC-MS/MS fragmentation of PnAla-Gly-Trp, PnAla-Gly-aThr, and PnAla-Gly-Gly

Refer to Table S88 for fragment ion formula, structures, and mass error.

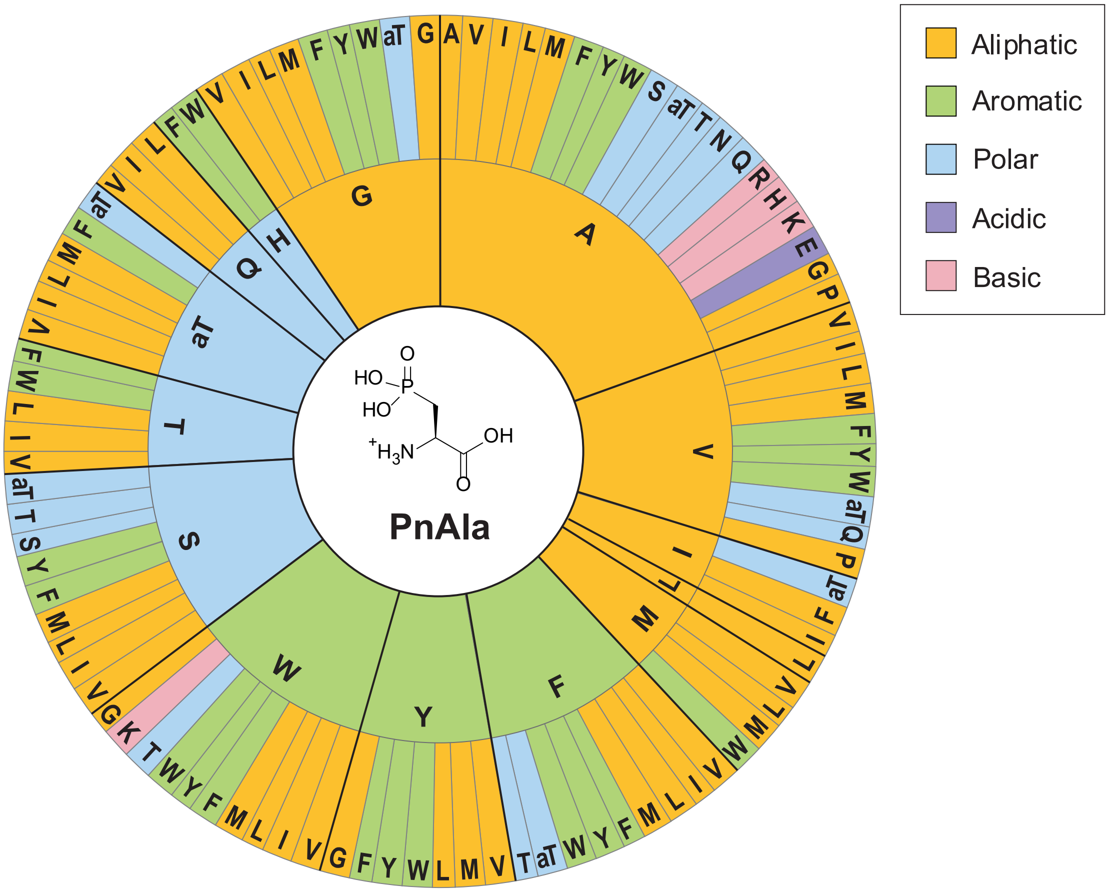

Figure S147. All N-terminal L-PnAla-containing tripeptides produced by PnaBC

A representation of all tripeptides produced by PnaBC. The sequence of each tripeptide is read from the inner circle outwards, such that PnAla-Ala-Val is represented by following the PnAla circle, the Ala inner ring, and the Val outer wedge. Amino acids are colored by their general properties.

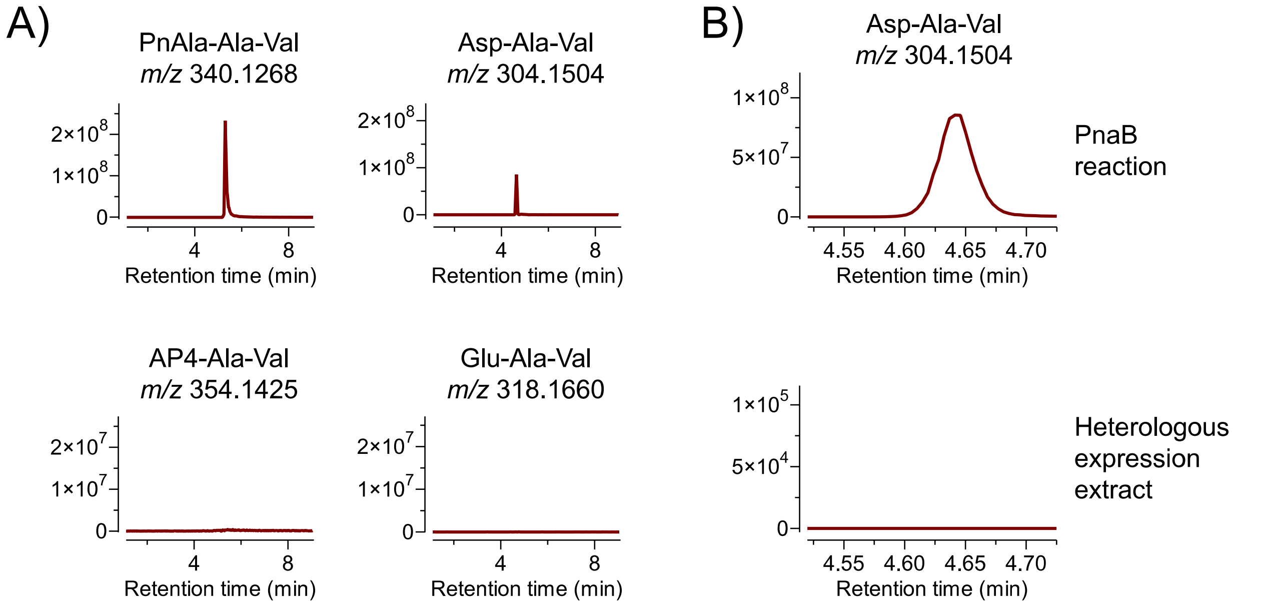

Figure S148. PnaB carboxylate specificity

To test the carboxylate specificity of PnaB, reactions were set up with ATP, Mg^{2+}, Ala-Val, and a panel of potential carboxylates: PnAla (the canonical substrate), Asp (the proteinogenic isostere), AP4 (the Pn isostere of Glu), and Glu (the proteinogenic isostere of AP4). A) LC-HRMS demonstrated that only the PnAla and Asp reactions yielded product. B) However, the corresponding EIC for Asp-Ala-Val (*m/z* 304.1504) was not observed within heterologous expression extracts (representative EIC shown).

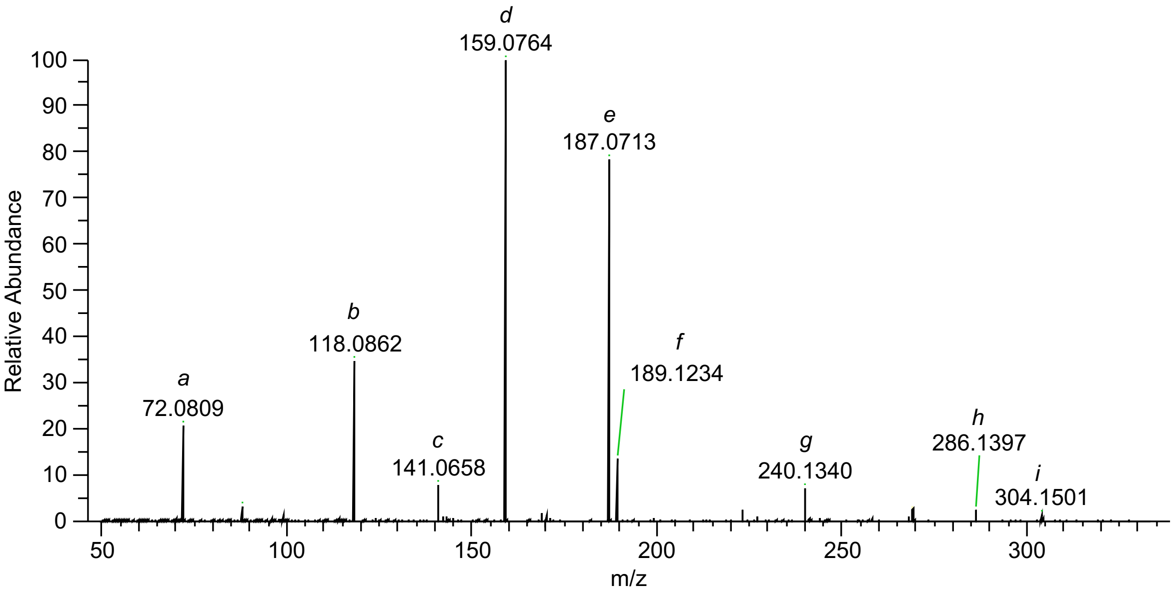

Figure S149. LC-HRMS/MS fragmentation of Asp-Ala-Val

Refer to Table S89 for fragment ion formula, structure, and mass error

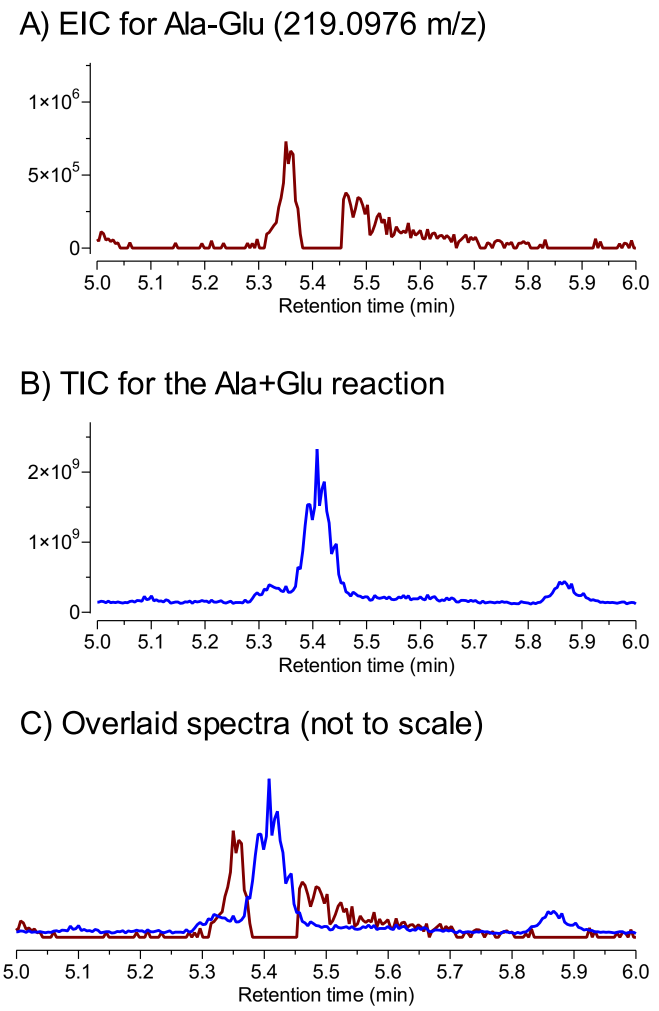

Figure S150. Impact of ion suppression on EIC spectra

A representative example of ion suppression leading to artifacts in EIC peaks. A) The EIC for Ala-Glu in the PnaC reaction containing Ala and Glu. B) The TIC for the same reaction. C) The overlay of the TIC over the Ala-Glu EIC demonstrates how ion suppression has caused a break in the EIC peak.

A) EIC for Asn-Val, 232.1292 m/z (Asn+Val reaction)

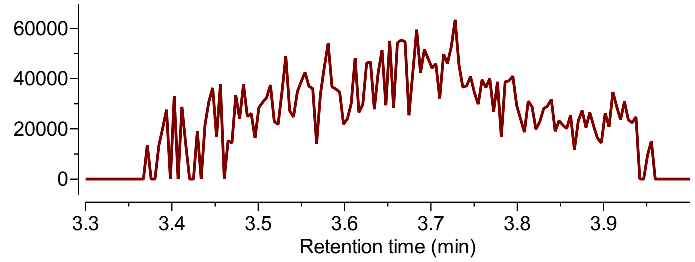

B) EIC for Asn-Val, 232.1292 m/z (Gly+Gly reaction)

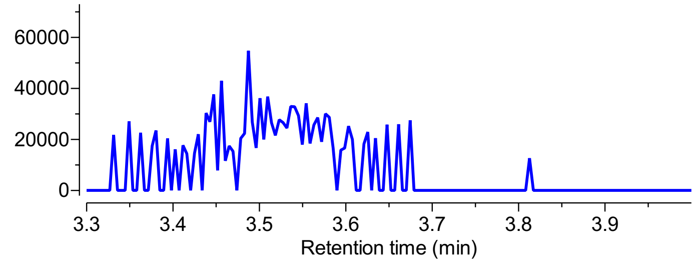

C) Spectra from 3.7-3.8 min within 5ppm of Asn-Val (Asn+Val rxn)

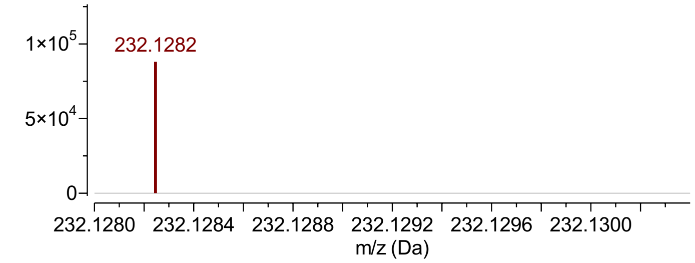

D) Spectra from 3.7-3.8 min within 5ppm of Asn-Val (Gly+Gly rxn)

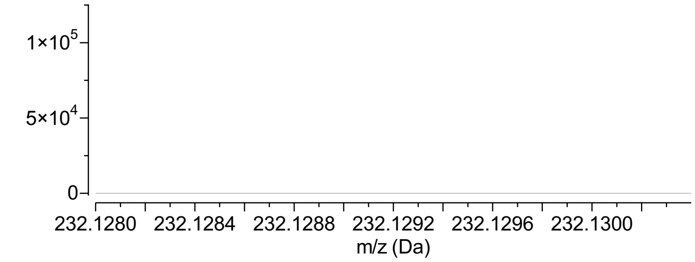

Figure S151. Background noise in EIC spectra

A representative example of background ions leading to noise in EIC spectra. A) The EIC for Asn-Val in the PnaC reaction containing Asn and Val. B) The same EIC in a PnaC reaction completely lacking Asn and Val, containing only Gly. C) The mass spectra for the window from 3.7-3.8 min in the Asn and Val reaction demonstrates a 232.1282 *m/z*, -4.31ppm from the theoretical *m/z* for protonated Asn-Val. D) The same region in the Gly reaction shows no such peak.

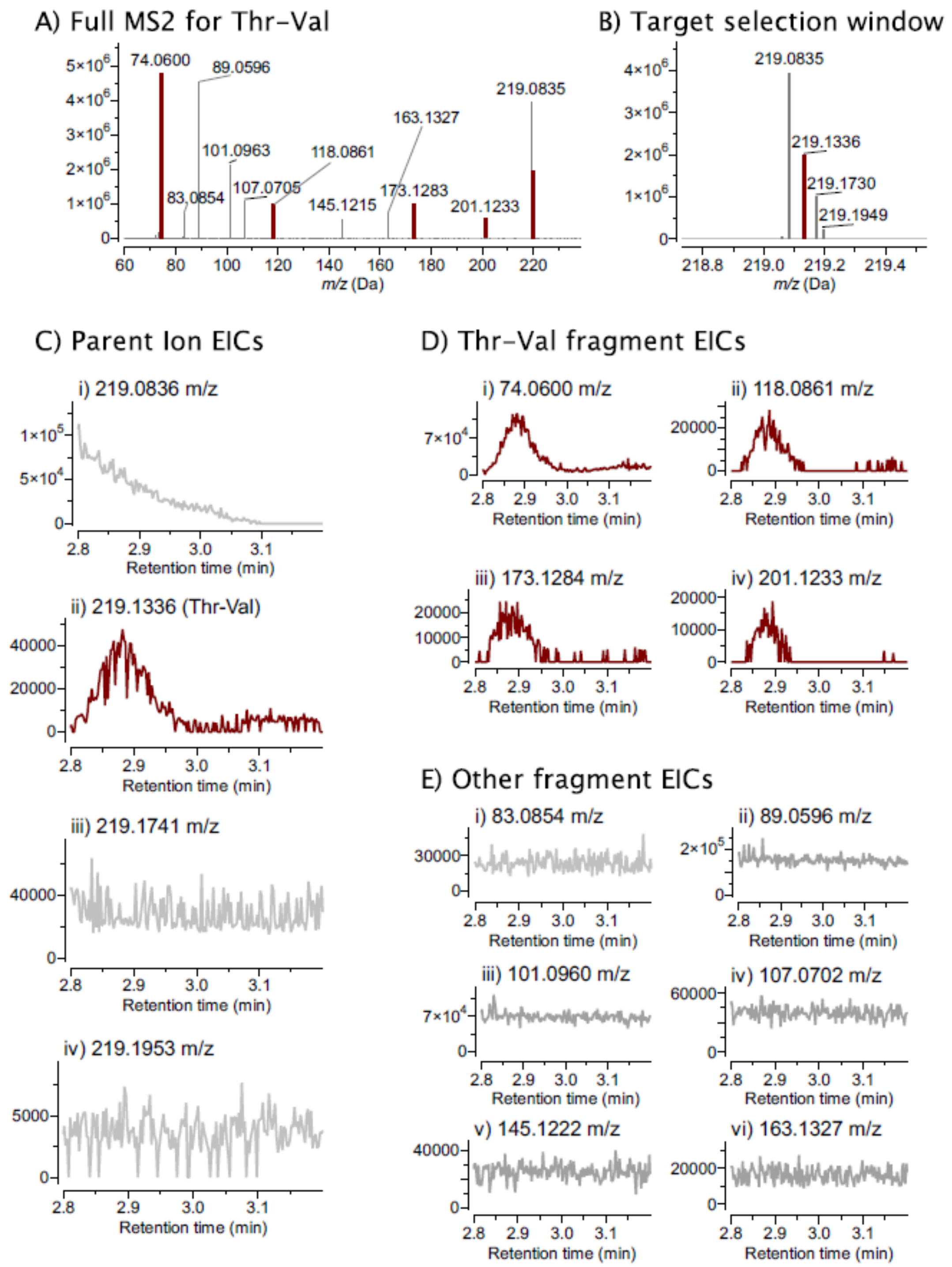

Figure S152. Contaminating parent ions in the selection window

The LC-HRMS used in this study has a minimum 0.4 *m/z* window for ion selection. A) The full MS/MS spectra for the Thr-Val species, 219.1339 *m/z*. B) Looking closer into the selection window, four parent ions can be seen. C) Only one of the four parent ions is within 5ppm of the theoretical *m/z* for protonated Thr-Val. D) The fragment ions which can be attributed to Thr-Val show similar EIC peaks. E) The other fragment ions have EICs which match the contaminating ions

SUPPLEMENTAL TABLES

Table S1. Annotation of the S-515 *pepM* neighborhood

Gene	Accession	Amino acids	NCBI annotation	Pfam annotation	CDD Annotation
orf1	WP_158820658	347	Helix-turn-helix (HTH) transcriptional regulator	Bacterial regulatory proteins, LuxR family (PF00196)	HTH superfamily (cl21459)
orf2	WP_051781700	245	2OG-Fe dioxygenase family protein	2OG-Fe dioxygenase (PF10014)	2OG-Fe_Oxy_2 superfamily (cl01794)
orf3	WP_030764880	963	FAD-binding oxidoreductase	FAD linked oxidases, C-terminal domain (PF02913)	GlcC / GlcD superfamilies
orf4	WP_030764879	252	Fumarylacetoacetate (FAA) hydrolase family protein	Fumarylacetoacetate hydrolase family (PF01557)	MhpD superfamily
orf5	WP_030764877	240	Sulfite exporter TauE/SafE family protein	Sulfite exporter TauE (PF01925)	TauE superfamily (cl21514)
pnaA	WP_051781701	410	Pyridoxal phosphate-dependent aminotransferase	Aminotransferase class I and II (PF00155)	Aspartate aminotransferase superfamily (cl18945)
pnaB	WP_030764872	431	ATP-grasp domain-containing protein	ATP-grasp domain (PF13535)	Arginosuccinate lyase superfamily (cl35175)
pnaC	WP_051781702	412	ATP-grasp domain-containing protein	ATP-grasp domain (PF13535)	Arginosuccinate lyase superfamily (cl35175)
pnaD	WP_030764868	292	Phosphoenolpyruvate mutase family protein	Phosphoenolpyruvate phosphomutase (PF13714)	[1]TIM superfamily (cl21457)
pnaT	WP_030902602	427	[2]MFS transporter	Major Facilitator Superfamily (PF07690)	MFS superfamily (cl28910)
orf11	WP_234440912	262	[3]Crp/[4]Fnr family transcriptional regulator	Crp-like HTH domain (PF13545)	Crp superfamily
orf12	WP_030764862	168	Hypothetical protein	Family of unknown function (DUF5706)	DUF5706 superfamily (cl40967)
orf13	WP_158820660	279	Hypothetical protein	Family of unknown function (DUF5545)	MSCRAMM_ClfA superfamily (cl41352)

FAA = Fumarylacetoacetate; [1]TIM = Triosphosphate Isomerase Mutase; [2]MFS = Major Facilitator Superfamily; [3]Crp = Catabolite Repressor Protein; [4]FNR = Fumarate and Nitrate Reductase; [5]MSCRAMM = Microbial Surface Components Recognizing Adhesive Matrix Molecules

Table S2. List of strains used in this study

Name	Description	Source or Reference
E. coli DH5α λ-pir	General cloning host	---
E. coli BL21(DE3)	Source of *serA* gene	Novagen
E. coli Rosetta(DE3) pLysSRARE	Protein overproduction host	Novagen
E. coli WM4489	Cloning host for fosmids; rhamnose inducible copy control	[13]
E. coli WM6029	Host for conjugation, auxotroph for 2,6-diaminopimelic acid	[14]
S. lividans 66	Heterologous host	USDA NRRL
S. coelicolor A3(2)	Source of *mdh* gene	USDA NRRL
Streptomyces sp. NRRL B-2790	Phosphonoalamide A-D producing strain	USDA NRRL
Streptomyces sp. NRRL S-515	Phosphonoalamide A-D producing strain	USDA NRRL
Streptomyces sp. NRRL S-448	Phosphonoalamide A-D producing strain	USDA NRRL
S. lividans KSJ4338	Derivative of *S. lividans* 66 ϕC31 *attB::*pKSJ553	This study
S. lividans KSJ4337	Derivative of *S. lividans* 66 ϕC31 *attB::*pKSJ554	This study
S. lividans KSJ4362	Derivative of *S. lividans* 66 ϕC31 *attB::*pKSJ588	This study
S. lividans KSJ4624	Derivative of *S. lividans* 66 ϕC31 *attB::*pKSJ595	This study
S. lividans KSJ4625	Derivative of *S. lividans* 66 ϕC31 *attB::*pKSJ596	This study

Table S3. List of plasmids used in this study

Plasmid	Description	Source
pAE4	Encodes *oriT,* ΦC31 integrase, ΦC31 *attP* for conjugation and site-specific recombination into in most *Streptomyces* chromosomes; Apr^R	[13]
pAE5	Source of aph(3')-II cassette for gene deletions; Km^R	[13]
pJK50	Dual-cos fosmid containing λattB site allowing integration with pAE4; Clm^R	[13]
pET28B	T7 based expression vector, N/C-terminal His_6, Km^R	Novagen
2-ST	pET His6 Sumo TEV LIC cloning vector (2S-T), Amp^R	Addgene
pKSJ409	Derivative of pET28B, overexpression and purification of His_6-PnaD; Km^R	This study
pKSJ441	Derivative of pET28B, overexpression and purification of malate dehydrogenase (His_6-MDH); Km^R	This study
pKSJ521	Derivative of pET28B, overexpression and purification of His_6-PnaB; Km^R	This study
pKSJ522	Derivative of pET28B, overexpression and purification of His_6-PnaC, Km^R	This study
pKSJ546	pJK50 derivative containing 5 upstream genes, *orf1-5*, and *pnaA-D* from *Streptomyces* sp. S-515; Clm^R	This study
pKSJ549	pJK50 derivative containing *orf1-5*, *pnaA-D, pnaT, orf11*, and 2 downstream genes from *Streptomyces* sp. S-515; Clm^R	This study
pKSJ553	Integration of pAE4 to the λattB site of pKSJ546; Apr^R, Clm^R	This study
pKSJ554	Integration of pAE4 to the λattB site of pKSJ549; Apr^R, Clm^R	This study
pKSJ588	Derivative of pKSJ554; cloned S-515 sequence upstream of *orf*2 replaced by the aph(3')-II cassette of pAE5; Km^R, Apr^R, Clm^R	This study
pKSJ595	Derivative of pKSJ553; cloned S-515 sequence upstream of *pnaA* replaced by the aph(3')-II cassette of pAE5; Km^R, Apr^R, Clm^R	This study
pKSJ596	Derivative of pKSJ554; cloned S-515 sequence upstream of *pnaA* replaced by the aph(3')-II cassette of pAE5; Km^R, Apr^R, Clm^R	This study
pKSJ599	Derivative of 2-ST, overexpression and purification of His_6-SUMO-PnaA; Amp^R	This study
pKSJ635	Derivative of pET28B, overexpression and purification of His_6-SerA; Km^R	This study

Km^R: Kanamycin resistant
Amp^R: Ampicillin resistant
Clm^R: Chloramphenicol resistant
Apr^R: Apramycin resistant

Table S4. Primers used in this study

**Primer**	**Sequence** (5’ to 3’)	**Purpose**
S515-pepM-screen-F	GATGAACAGCTTCGTCGCCAC	Fosmid library screening
S515-pepM-screen-R	CCCTGGTTGGCGTAGATGA	Fosmid library screening
orf1-left-Redswap-F	tccgccaccgcacggggcggcgcgggcggtggggcctcaCCTAGGtcgaggttgacatcttttgc	PCR amplification of aph-II cassette to replace genes upstream *orf1* by λ-Red recombination
orf1-left-Redswap-R	tacattatacgaagttatcaagaagcttgagctcggatcCCTAGGtcagaagaactcgtcaagaagg	
pnaA-left-Redswap-R	tggtgagggtggtgaggacggaacgggtacggcgggccgCCTAGGtcagaagaactcgtcaagaagg	PCR amplification of aph-II cassette to replace genes upstream *pnaA* by λ-Red recombination (orf1-left-Redswap-F was used as the forward primer)
pET28b-NdeI-R	CATATGGCTGCCGCGCGGCACCAGGC	Production of linearized pET28B with N-His_6 for Gibson assembly
pET28b-XhoI-F	CTCGAGCACCACCACCACCACCACTG	
2ST-F	TGCATTGGATTGGAAGTACAG	Production of linearized 2-ST with N-His_6-SUMO for Gibson assembly
2ST-R	ATTGGAAGTGGATAACGGATC	
pET28b-NHis-PnaB-F	TGCCGCGCGGCAGCCATATGtcaggtgacctccggccgctc	PCR amplification of *pnaB* for Gibson assembly cloning into pET28B
pET28b-NHis-PnaB-R	TGGTGGTGGTGGTGCTCGAGtcatcccgtcgccccgaagtcctc	
pET28b-NHis-PnaC-F	TGCCGCGCGGCAGCCATATGacccgtcacatcgccttcctg	PCR amplification of *pnaC* for Gibson assembly cloning into pET28B
pET28b-NHis-PnaC-R	TGGTGGTGGTGGTGCTCGAGtcagtgcgagtcggcggtccggac	
2ST-PnaA-F	TTATCCACTTCCAATtcacctgacatcgtccatcac	PCR amplification of *pnaA* for Gibson assembly cloning into 2-ST
2ST-PnaA-R	TTCCAATCCAATGCAatgcccgaccagcaggtcgac	
S515-pepM-F	GCCTGGTGCCGCGCGGCAGCCATATGttcaagcccgtttcgtac	PCR amplification of *pnaD* for Gibson assembly cloning into pET28B
S515-pepM-R	AGTGGTGGTGGTGGTGGTGCTCGAGtcacgcggactcgagctcctg	
pET28B-NHis-SCO4927-Fd	TGCCGCGCGGCAGCCATATGactcgcactcccgtgaacgt	PCR amplification of *mdh* for Gibson assembly cloning into pET28B
pET28B-NHis-SCO4927-Rv	TGGTGGTGGTGGTGCTCGAGtcagatgaggccgagaccgc	
pET28B-NHis-SerA-F	TGCCGCGCGGCAGCCATATGgcaaaggtatcgctggag	PCR amplification of *serA* for Gibson assembly cloning in pET28B
pET28B-NHis-SerA-R	TGGTGGTGGTGGTGCTCGAGttagtacagcagacgggcgcg	

Primers used in Gibson assembly. Lowercase represents oligonucleotides bound to the gene, and uppercase represents the oligonucleotides homologous to the vector.

Primers used in λ-Red recombination. Oligonucleotides on the 5’ side are homologous to upstream or downstream sequences of the target gene. Oligonucleotides on the 3’ side bind to aph-II cassette from pAE5.

Table S5. Ala-Ala, Gly-Ala, and Pro-Ala dipeptide fragments

Fragment				Ala-Ala		Gly-Ala		Pro-Ala	
ID	Structure	Formula	Calc *m/z*	Obs *m/z*	Δppm	Obs *m/z*	Δppm	Obs *m/z*	Δppm
a	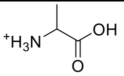	$C3H8NO2^+$	90.0550	90.0549	-0.49	90.0549	-0.67	90.0548	-2.14
b	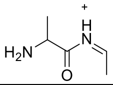	$C5H11N2O^+$	115.0866	115.0865	-0.75				
c	*b* + H2O	$C5H13N2O2^+$	133.0972	133.0971	-0.54				
d	*e* – H2O	$C6H11N2O2^+$	143.0815	143.0814	-0.40				
e	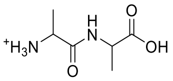	$C6H13N2O3^+$	161.0921	161.0920	-0.70				
f	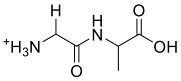	$C5H11N2O3^+$	147.0764			147.0763	-0.69		
g	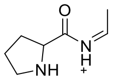	$C7H13N2O^+$	141.1022					141.1018	-2.77
h	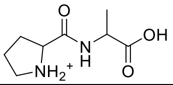	$C8H15N2O3^+$	187.1077					187.1072	-2.82

Table S6. Ala-Val, Val-Val, Leu-Val, and Ser-Val dipeptide fragments

Fragment				Ala-Val		Val-Val		Leu-Val		Ser-Val	
ID	Structure	Formula	Calc *m/z*	Obs *m/z*	Δppm	Obs *m/z*	Δppm	Obs *m/z*	Δppm	Obs *m/z*	Δppm
a	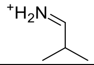	$C4H10N^+$	72.0808	72.0808	0.03	72.0808	-0.34	72.0807	-0.68	72.0808	0.16
b	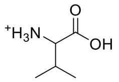	$C5H12NO2^+$	118.0863	118.0861	-1.46	118.086	-1.78			118.0861	-1.24
c	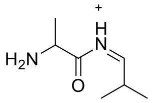	$C7H15N2O^+$	143.1179	143.1178	-0.66						
d	*e* – H2O	$C8H15N2O2^+$	171.1128	171.1126	-1.00						
e	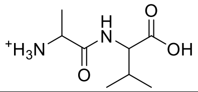	$C8H17N2O3^+$	189.1234	189.1231	-1.26						
f	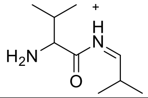	$C9H19N2O^+$	171.1492			171.1489	-1.47				
g	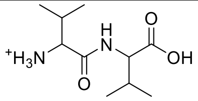	$C10H21N2O3^+$	217.1547			217.1543	-1.52				
h	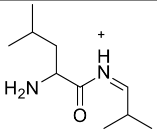	$C10H21N2O^+$	185.1648					185.1649	0.36		
i	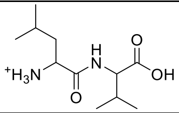	$C11H23N2O3^+$	231.1703					231.1698	-2.19		
j	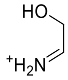	$C2H6NO^+$	60.0444							60.0447	5.77
k	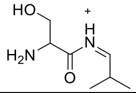	$C7H15N2O2^+$	159.1128							159.1127	-0.65
l	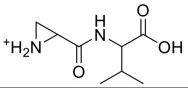	$C8H15N2O3^+$	181.1077							187.1075	-1.00
m	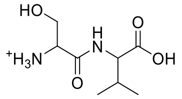	$C8H17N2O4^+$	205.1183							205.1181	-0.67

Table S7. Thr-Val, aThr-Val, Asn-Val, and Gln-Val dipeptide fragments

Fragment				Thr-Val		aThr-Val		Asn-Val		Gln-Val	
ID	Structure	Formula	Calc *m/z*	Obs *m/z*	Δppm	Obs *m/z*	Δppm	Obs *m/z*	Δppm	Obs *m/z*	Δppm
b	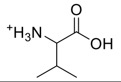	C5H12NO2$^+$	118.0863	118.0861	-1.38	118.0860	-2.26	118.0863	0.21	118.0859	-2.84
n	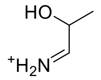	C3H8NO$^+$	74.0600	74.0600	-0.77	74.0599	-1.79				
o	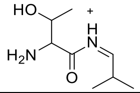	C8H17N2O2$^+$	173.1285	173.1284	-0.55	173.1282	-1.51				
p	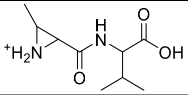	C9H17N2O3$^+$	201.1234	201.1233	-0.45	201.1230	-1.75				
q	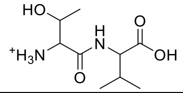	C9H19N2O4$^+$	219.1339	219.1336	-1.58	219.1333	-2.68				
r	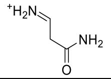	C3H7N2O$^+$	87.0553					87.0553	0.28		
s	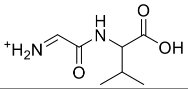	C7H13N2O3$^+$	173.0921					173.0920	-0.41		
t	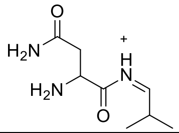	C8H16N3O2$^+$	186.1237					186.1238	0.50		
u	*v* – NH3	C9H12NO4$^+$	198.0761					198.0762	0.48		
v	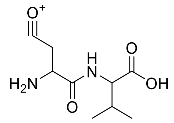	C9H15N2O4$^+$	215.1026					215.1023	-1.36		
w	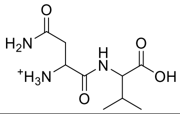	C9H18N3O4$^+$	232.1292					232.1290	-0.72		
x	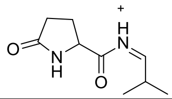	C9H15N2O2$^+$	183.1128							183.1125	-1.86
y	*aa* – H2O	C10H18N3O3$^+$	228.1343							228.1337	-2.64
z	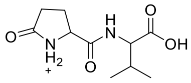	C10H17N2O4$^+$	229.1183							229.1177	-2.75
aa	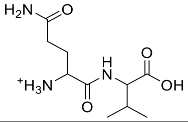	C10H20N3O4$^+$	246.1448							246.1444	-1.60

Table S8. Asp-Val, Glu-Val, Gly-Val, and Pro-Val dipeptide fragments

Fragment				Asp-Val		Glu-Val		Gly-Val		Pro-Val	
ID	Structure	Formula	Calc *m/z*	Obs *m/z*	Δppm	Obs *m/z*	Δppm	Obs *m/z*	Δppm	Obs *m/z*	Δppm
a	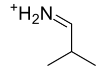	C4H10N$^+$	72.0808			72.0807	-1.62	72.0808	0.14	72.0806	-3.00
b	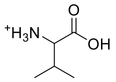	C5H12NO2$^+$	118.0863	118.0862	-0.87	118.0859	-2.88	118.0861	-1.34	118.0858	-3.53
ab	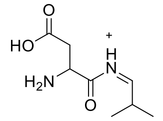	C8H15N2O3$^+$	187.1077	187.1078	0.16						
ac	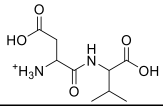	C9H17N2O5$^+$	233.1132	233.1138	2.45						
ad	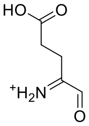	C5H8NO3$^+$	130.0499			130.0496	-1.91				
ae	*ag* – COOH	C9H14NO3$^+$	184.0968			184.0965	-2.00				
af	*ah* – NH3	C10H16NO5$^+$	230.1023			230.1017	-2.80				
ag	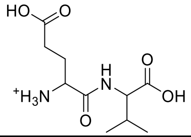	C10H19N2O5$^+$	247.1289			247.1282	-2.79				
ah	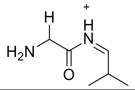	C6H13N2O$^+$	129.1022					129.1021	-1.07		
ai	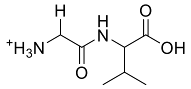	C7H15N2O3$^+$	175.1077					175.1076	-0.66		
aj	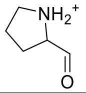	C5H10NO$^+$	100.0757							100.0754	-2.58
ak	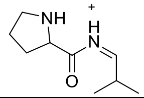	C9H17N2O$^+$	169.1335							169.1331	-2.86
al	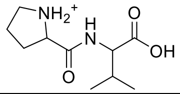	C10H19N2O3$^+$	215.139							215.1384	-2.89

Table S9. Ala-Ile, Val-Ile, Ile-Ile, and Leu-^{15}N-Ile dipeptide fragments

Fragment				Ala-Ile		Val-Ile		Ile-Ile		Leu-(^{15}N-Ile)	
ID	Structure	Formula	Calc m/z	Obs m/z	Δppm	Obs m/z	Δppm	Obs m/z	Δppm	Obs m/z	Δppm
a	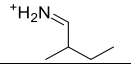	C5H12N$^+$	86.0964	86.0963	-1.35	86.0963	-1.81	86.0962	-2.42		
b	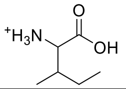	C6H14NO2$^+$	132.1019	132.1018	-0.91	132.1018	-0.63	132.1016	-2.20		
c	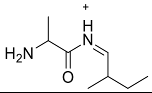	C8H17N2O$^+$	157.1335	157.1334	-0.63						
d	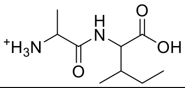	C9H19N2O3$^+$	203.1390	203.1389	-0.53						
e	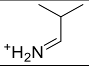	C4H10N$^+$	72.0808			72.0808	-0.35				
f	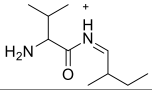	C10H21N2O$^+$	185.1648			185.1649	0.21				
g	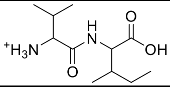	C11H23N2O3$^+$	231.1703			231.1698	-2.31				
h	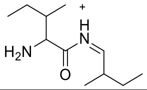	C11H23N2O$^+$	199.1805					199.1801	-1.81		
i	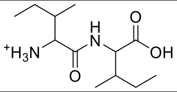	C12H25N2O3$^+$	245.1860					245.1853	-2.92		
j	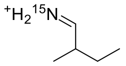	C5H12^{15}N$^+$	87.0935							87.0935	0.07
k	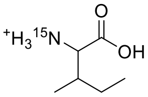	C6H14^{15}NO2$^+$	133.0989							133.0990	0.13
l	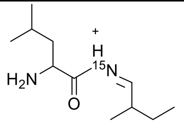	C11H23N^{15}NO$^+$	200.1775							200.1776	0.13
m	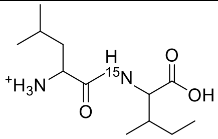	C12H25N^{15}NO3$^+$	246.1830							246.1828	-1.01

Table S10. Ser-Ile, Thr-Ile, aThr-Ile, and Gln-Ile dipeptide fragments

Fragment				Ser-Ile		Thr-Ile		aThr-Ile		Gln-Ile	
ID	Structure	Formula	Calc m/z	Obs m/z	Δppm	Obs m/z	Δppm	Obs m/z	Δppm	Obs m/z	Δppm
a	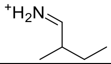	C5H12N$^+$	86.0964	86.0963	-1.71	86.0963	-1.86	86.0962	-2.07	86.0962	-2.34
b	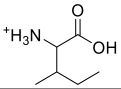	C6H14NO2$^+$	132.1019	132.1018	-1.11	132.1017	-1.19	132.1017	-1.51	132.1017	-1.23
n	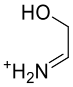	C2H6NO$^+$	60.0444	60.0447	5.56						
o	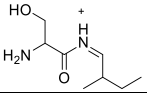	C8H17N2O2$^+$	173.1285	173.1283	-0.78						
p	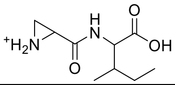	C9H17N2O3$^+$	201.1234	201.1231	-1.34						
q	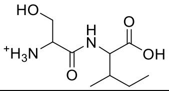	C9H19N2O4$^+$	219.1339	219.1336	-1.33						
r	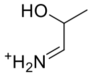	C3H8NO$^+$	74.0600			74.0599	-1.46	74.0600	-0.79		
s	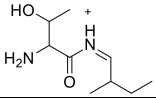	C9H19N2O2$^+$	187.1441			187.1439	-1.13	187.1439	-1.23		
t	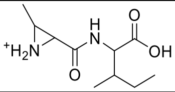	C10H19N2O3$^+$	215.139			215.1388	-1.05	215.1387	-1.58		
u	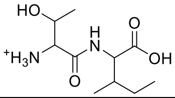	C10H21N2O4$^+$	233.1496			233.1491	-2.00	233.1491	-2.26		
v	*w* – NH3	C4H6NO$^+$	84.0444					84.0443	-1.38		
w	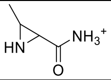	C4H9N2O$^+$	101.0709					101.0708	-0.94		
x	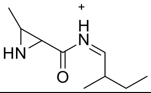	C9H17N2O$^+$	169.1335					169.1332	-1.74		
y	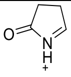	C4H6NO$^+$	84.0444							84.0443	-1.13
z	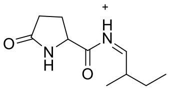	C10H17N2O2$^+$	197.1285							197.1282	-1.41
aa	*ac* – H2O	C11H20N3O3$^+$	242.1499							242.1492	-2.84
ab	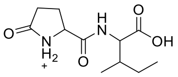	C11H19N2O4$^+$	243.1339							243.1334	-2.14
ac	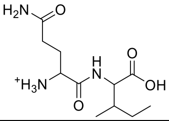	C11H22N3O4$^+$	260.1605							260.1603	-0.88

Table S11. Asp-Ile, Gly-Ile, and Pro-Ile dipeptide fragments

Fragment				Asp-Ile		Gly-Ile		Pro-Ile	
ID	Structure	Formula	Calc m/z	Obs m/z	Δppm	Obs m/z	Δppm	Obs m/z	Δppm
a	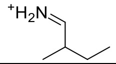	$C5H12N^{+}$	86.0964	86.0964	-0.39	86.0963	-1.32	86.0961	-3.26
b	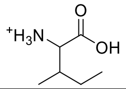	$C6H14NO2^{+}$	132.1019	132.1019	0.01	132.1018	-0.69	132.1016	-2.69
ad	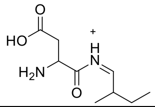	$C9H17N2O3^{+}$	201.1234	201.1235	0.81				
ae	*af* – NH3	$C10H14NO4^{+}$	212.0917	212.0919	0.95				
af	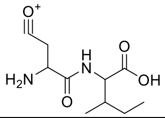	$C10H17N2O4^{+}$	229.1183	229.1181	-0.80				
ag	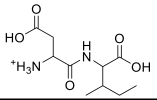	$C10H19N2O5^{+}$	247.1289	247.1284	-1.72				
ah	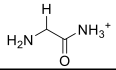	$C2H7N2O^{+}$	75.0553			75.0553	-0.11		
ai	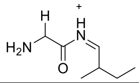	$C7H15N2O^{+}$	143.1179			143.1178	-0.53		
aj	*ak* – H2O	$C8H15N2O2^{+}$	171.1128			171.1126	-1.28		
ak	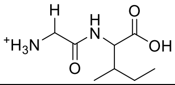	$C8H17N2O3^{+}$	189.1234			189.1232	-0.79		
al	*b* – H2O	$C6H12NO^{+}$	114.0913					114.0911	-2.41
am	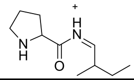	$C10H19N2O^{+}$	183.1492					183.1487	-2.71
an	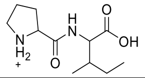	$C11H21N2O3^{+}$	229.1547					229.1540	-3.09

Table S12. Ala-Leu, Val-Leu, ^{15}N-Ile-Leu, and Leu-Leu dipeptide fragments

Fragment				Ala-Leu		Val-Leu		(^{15}N-Ile)-Leu		Leu-Leu	
ID	Structure	Formula	Calc m/z	Obs m/z	Δppm	Obs m/z	Δppm	Obs m/z	Δppm	Obs m/z	Δppm
a	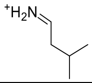	$C5H12N^+$	86.0964	86.0963	-1.44	86.0962	-2.11	86.0964	-0.34	86.0962	-2.24
b	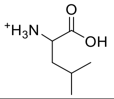	$C6H14NO2^+$	132.1019	132.1018	-0.89	132.1017	-1.27	132.1019	-0.1	132.1018	-0.87
c	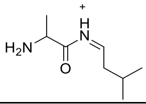	$C8H17N2O^+$	157.1335	157.1334	-0.65						
d	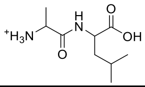	$C9H19N2O3^+$	203.139	203.1388	-0.88						
e	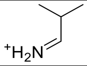	$C4H10N^+$	72.0808			72.0807	-1.39				
f	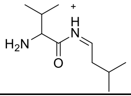	$C10H21N2O^+$	185.1648			185.1649	0.36				
g	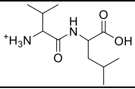	$C11H23N2O3^+$	231.1703			231.1698	-2.19				
h	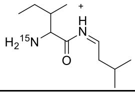	$C11H23N{*}NO^+$	200.1775					200.1776	0.13		
i	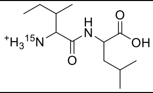	$C12H25N{*}NO3^+$	246.183					246.1828	-1.01		
j	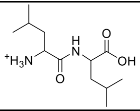	$C12H25N2O3^+$	245.186							245.1855	-1.84

Table S13. Ser-Leu, Thr-Leu, aThr-Leu, and Gln-Leu dipeptide fragments

Fragment				Ser-Leu		Thr-Leu		aThr-Leu		Gln-Leu	
ID	Structure	Formula	Calc m/z	Obs m/z	Δppm	Obs m/z	Δppm	Obs m/z	Δppm	Obs m/z	Δppm
a	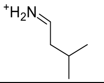	C5H12N$^+$	86.0964	86.0963	-1.55			86.0963	-2.01		
b	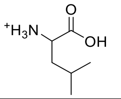	C6H14NO2$^+$	132.1019	132.1018	-0.93	132.1016	-2.23	132.1017	-1.39	132.1017	-1.88
k	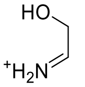	C2H6NO$^+$	60.0444	60.0447	5.72						
l	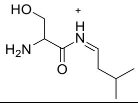	C8H17N2O2$^+$	173.1285	173.1283	-0.63						
m	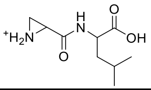	C9H17N2O3$^+$	201.1234	201.1231	-1.15						
n	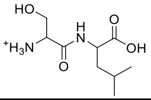	C9H19N2O4$^+$	219.1339	219.1336	-1.32						
o	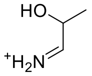	C3H8NO$^+$	74.0600			74.0599	-2.21	74.0599	-1.53		
p	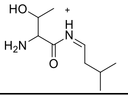	C9H19N2O2$^+$	187.1441			187.1437	-2.15	187.1439	-1.13		
q	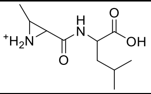	C10H19N2O3$^+$	215.139			215.1386	-1.86	215.1387	-1.60		
r	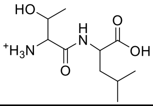	C10H21N2O4$^+$	233.1496			233.1489	-2.98	233.1493	-1.20		
s	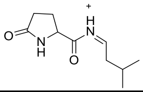	C10H17N2O2$^+$	197.1285							197.1281	-1.86
t	*w* – H2O	C11H20N3O3$^+$	242.1499							242.1490	-3.68
u	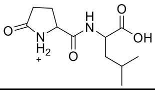	C11H19N2O4$^+$	243.1339							243.1332	-3.13
v	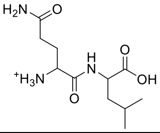	C11H22N3O4$^+$	260.1605							260.1598	-2.73

Table S14. Gly-Leu and Pro-Leu dipeptide fragments

Fragment				Gly-Leu		Pro-Leu	
ID	Structure	Formula	Calc m/z	Obs m/z	Δppm	Obs m/z	Δppm
a	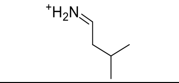	C5H12N$^+$	86.0964	86.0963	-1.48	86.0962	-2.72
b	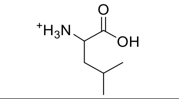	C6H14NO2$^+$	132.1019	132.1018	-1.03	132.1016	-2.17
w	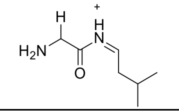	C7H15N2O$^+$	143.1179	143.1178	-0.78		
x	*z* – H2O	C8H15N2O2$^+$	171.1128	171.1126	-1.21		
y	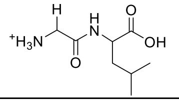	C8H17N2O3$^+$	189.1234	189.1231	-1.27		
z	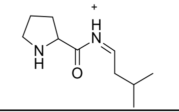	C10H19N2O$^+$	183.1492			183.1488	-2.26
aa	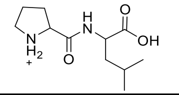	C11H21N2O3$^+$	229.1547			229.1541	-2.69

Table S15. Ala-Met, Val-Met, Leu-Met, and Met-Met dipeptide fragments

Fragment				Ala-Met		Val-Met		Leu-Met		Met-Met	
ID	Structure	Formula	Calc m/z	Obs m/z	Δppm	Obs m/z	Δppm	Obs m/z	Δppm	Obs m/z	Δppm
a	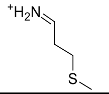	C4H10NS$^+$	104.0529	104.0527	-1.57	104.0526	-2.69	104.0527	-1.85	104.0527	-1.37
b	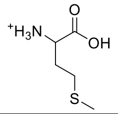	C5H12NO2S$^+$	150.0583	150.0582	-0.94	150.0580	-1.88	150.0581	-1.20	150.0582	-0.54
c	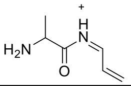	C6H11N2O$^+$	127.0866	127.0864	-1.31						
d	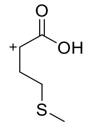	C5H9O2S$^+$	133.0318	133.0316	-1.21						
e	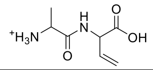	C7H13N2O3$^+$	173.0921	173.0919	-0.77						
f	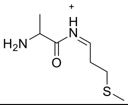	C7H15N2OS$^+$	175.0900	175.0898	-1.08						
g	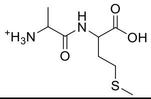	C8H17N2O3S$^+$	221.0954	221.0951	-1.52						
h	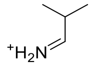	C4H10N$^+$	72.0808			72.0807	-1.32				
i	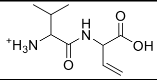	C9H17N2O3$^+$	201.1234			201.1230	-1.84				
j	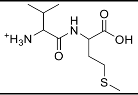	C10H21N2O3S$^+$	249.1267			249.1262	-2.14				
k	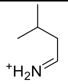	C5H12N$^+$	86.0964					86.0963	-1.80		
l	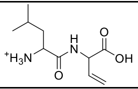	C10H19N2O3$^+$	215.139					215.1389	-0.47		
m	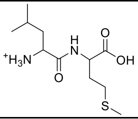	C11H23N2O3S$^+$	263.1424					263.1419	-2.04		
n	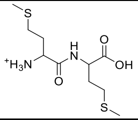	C10H21N2O3S2$^+$	281.0988							281.0985	-1.26

Table S16. Phe-Met, Tyr-Met, Ser-Met, and Thr-Met dipeptide fragments

Fragment				Phe-Met		Tyr-Met		Ser-Met		Thr-Met	
ID	Structure	Formula	Calc m/z	Obs m/z	Δppm	Obs m/z	Δppm	Obs m/z	Δppm	Obs m/z	Δppm
a	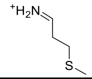	$C4H10NS^{+}$	104.0529	104.0526	-2.73	104.0526	-2.66				
b	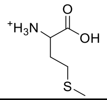	$C5H12NO2S^{+}$	150.0583	150.0581	-1.30	150.0581	-1.64	150.0583	-0.31	150.0584	0.48
o	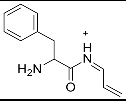	$C12H15N2O^{+}$	203.1179	203.1174	-2.3						
p	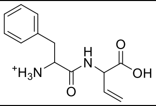	$C13H17N2O3^{+}$	249.1234	249.1232	-0.79						
q	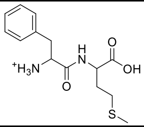	$C14H21N2O3S^{+}$	297.1267	297.1264	-1.00						
r	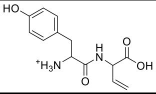	$C13H17N2O4^{+}$	265.1183			265.1177	-2.05				
s	*t* – H2O	$C14H18NO4S^{+}$	296.0951			296.0949	-0.67				
t	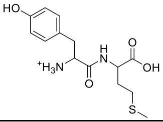	$C14H21N2O4S^{+}$	313.1217			313.1210	-1.99				
u	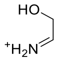	$C2H6NO^{+}$	60.0444					60.0447	5.90		
v	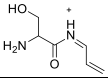	$C6H11N2O2^{+}$	143.0815					143.0815	-0.30		
w	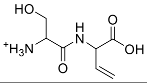	$C7H13N2O4^{+}$	189.087					189.0868	-0.85		
x	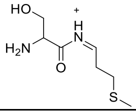	$C7H15N2O2S^{+}$	191.0849					191.0847	-0.97		
y	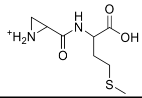	$C8H15N2O3S^{+}$	219.0798					219.0796	-0.85		
z	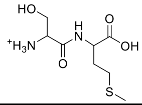	$C8H17N2O4S^{+}$	237.0904					237.0900	-1.35		
aa	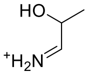	$C3H8NO^{+}$	74.0600							74.0600	-0.05
ab	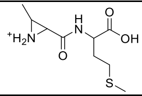	$C9H17N2O3S^{+}$	233.0941								
ac	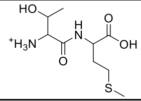	$C9H19N2O4S^{+}$	251.106							251.1054	-2.52

Table S17. aThr-Met, Gly-Met, and Pro-Met dipeptide fragments

Fragment				aThr-Met		Gly-Met		Pro-Met	
ID	Structure	Formula	Calc m/z	Obs m/z	Δppm	Obs m/z	Δppm	Obs m/z	Δppm
a	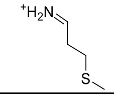	$C4H10NS^+$	104.0529			104.0526	-2.1	104.0526	-2.64
b	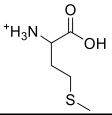	$C5H12NO2S^+$	150.0583	150.0584	0.40	150.0581	-1.53	150.0581	-1.82
aa	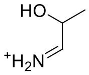	$C3H8NO^+$	74.0600	74.0600	-0.47				
ab	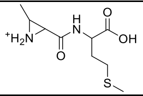	$C9H17N2O3S^+$	233.0941	233.0946	2.12				
ac	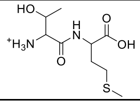	$C9H19N2O4S^+$	251.106	251.1054	-2.25				
ad	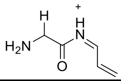	$C5H9N2O^+$	113.0709			113.0706	-2.79		
ae	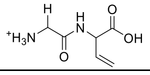	$C6H11N2O3^+$	159.0764			159.0761	-2.07		
af	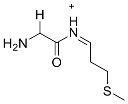	$C6H13N2OS^+$	161.0743			161.0741	-1.6		
ag	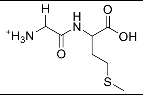	$C7H15N2O3S^+$	207.0798			207.0794	-1.82		
ah	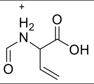	$C5H8NO3^+$	130.0499					130.0496	-1.84
ai	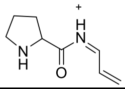	$C8H13N2O^+$	153.1022					153.1019	-2.25
aj	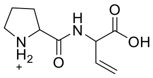	$C9H15N2O3^+$	199.1077					199.1072	-2.64
ak	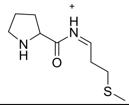	$C9H17N2OS^+$	201.1056					201.1051	-2.56
al	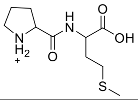	$C10H19N2O3S^+$	247.1111					247.1104	-2.65

Table S18. Ala-Phe, Val-Phe, Ile-Phe, and Leu-Phe dipeptide fragments

Fragment				Ala-Phe		Val-Phe		Ile-Phe		Leu-Phe	
ID	Structure	Formula	Calc m/z	Obs m/z	Δppm	Obs m/z	Δppm	Obs m/z	Δppm	Obs m/z	Δppm
a	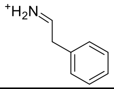	$C8H10N^+$	120.0808	120.0806	-1.20	120.0805	-2.01	120.0806	-1.37	120.0805	-2.22
b	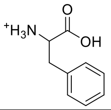	$C9H12NO2^+$	166.0863	166.0861	-1.06	166.0860	-1.68	166.0862	-0.55	166.0859	-2.09
c	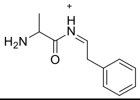	$C11H15N2O^+$	191.1179	191.1176	-1.37						
d	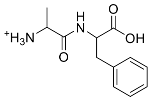	$C12H17N2O3^+$	237.1234	237.1230	-1.46						
e	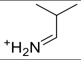	$C4H10N^+$	72.0808			72.0807	-0.99				
f	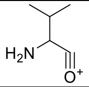	$C5H10NO^+$	100.0757			100.0754	-2.49				
g	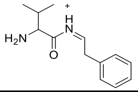	$C13H19N2O^+$	219.1492			219.1487	-2.07				
h	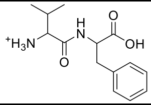	$C14H21N2O3^+$	265.1547			265.1539	-2.98				
i	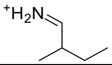	$C5H12N^+$	86.0964					86.0963	-1.16		
j	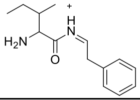	$C14H21N2O^+$	233.1648					233.1645	-1.46		
k	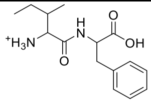	C15H23N2O3	279.1703					279.1701	-0.80		
l	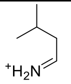	$C5H12N^+$	86.0964							86.0962	-3.13
m	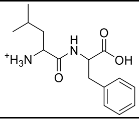	$C15H23N2O3^+$	279.1703							279.1697	-2.11

Table S19. Met-Phe, Phe-Phe, Tyr-Phe, and Ser-Phe dipeptide fragments

Fragment				Met-Phe		Phe-Phe		Tyr-Phe		Ser-Phe	
ID	Structure	Formula	Calc m/z	Obs m/z	Δppm	Obs m/z	Δppm	Obs m/z	Δppm	Obs m/z	Δppm
a	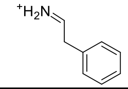	C8H10N$^+$	120.0808	120.0805	-2.30	120.0806	-1.11	120.0806	-1.07	120.0806	-1.73
b	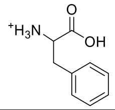	C9H12NO2$^+$	166.0863	166.0861	-0.76	166.0860	-1.73	166.0862	-0.48	166.0860	-1.54
n	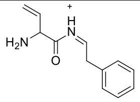	C12H15N2O$^+$	203.1179	203.1174	-2.30						
o	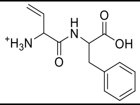	C13H17N2O3$^+$	249.1234	249.1232	-0.79						
p	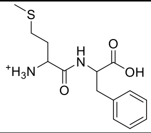	C14H21N2O3S$^+$	297.1267	297.1264	-1.00						
q	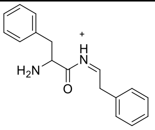	C17H19N2O$^+$	267.1492			267.1486	-2.36				
r	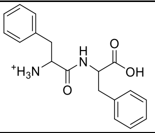	C18H21N2O3$^+$	313.1547			313.1535	-3.62				
s	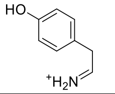	C8H10NO$^+$	136.0757					136.0756	-0.72		
t	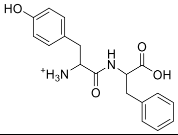	C18H21N2O4$^+$	329.1496					329.1493	-0.98		
u	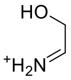	C2H6NO$^+$	60.0444							60.0447	4.81
v	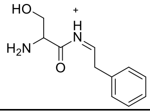	C11H15N2O2$^+$	207.1128							207.1125	-1.47
w	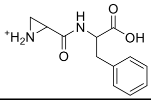	C12H15N2O3$^+$	235.1077							235.1072	-2.04
x	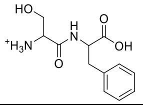	C12H17N2O4$^+$	253.1183							253.1176	-2.54

Table S20. Thr-Phe, aThr-Phe, Gly-Phe, and Pro-Phe dipeptide fragments

Fragment				Thr-Phe		aThr-Phe		Gly-Phe		Pro-Phe	
ID	Structure	Formula	Calc m/z	Obs m/z	Δppm	Obs m/z	Δppm	Obs m/z	Δppm	Obs m/z	Δppm
a	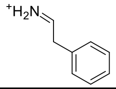	C8H10N$^+$	120.0808	120.0805	-2.50	120.0805	-2.06	120.0805	-2.54	120.0805	-2.20
b	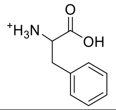	C9H12NO2$^+$	166.0863	166.0858	-2.48	166.0860	-1.31	166.0859	-2.43	166.0859	-1.95
y	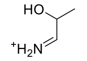	C3H8NO$^+$	74.0600	74.0599	-1.56	74.0599	-1.51				
z	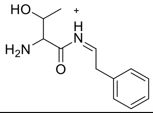	C12H17N2O2$^+$	221.1285	221.1279	-2.33						
aa	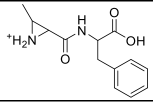	C13H17N2O3$^+$	249.1234	249.1229	-1.94	249.1230	-1.58				
ab	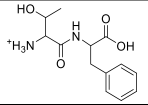	C13H19N2O4$^+$	267.1339	267.1333	-2.41	267.1334	-1.84				
ac	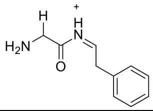	C10H13N2O$^+$	177.1022					177.1018	-2.72		
ad	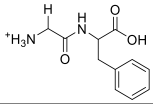	C11H15N2O3$^+$	223.1077					223.1072	-2.49		
ae	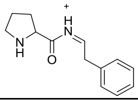	C13H17N2O$^+$	217.1335							217.1331	-2.04
af	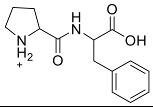	C14H19N2O3$^+$	263.1390							263.1383	-2.83

Table S21. Ala-Tyr, Val-Tyr, and Leu-Tyr dipeptide fragments

Fragment				Ala-Tyr		Val-Tyr		Leu-Tyr	
ID	Structure	Formula	Calc m/z	Obs m/z	Δppm	Obs m/z	Δppm	Obs m/z	Δppm
a	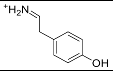	C8H10NO$^+$	136.0757	136.0756	-0.37	136.0755	-1.62	136.0754	-2.07
b	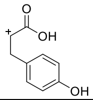	C9H9O3$^+$	165.0546	165.0545	-0.69	165.0544	-1.39		
c	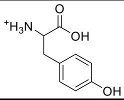	C9H12NO3$^+$	182.0812	182.0810	-0.67	182.0808	-1.81	182.0808	-2.29
d	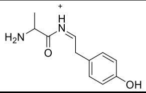	C11H15N2O2$^+$	207.1128	207.1126	-0.89				
e	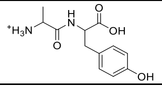	C12H17N2O4$^+$	253.1183	253.1178	-1.74				
f	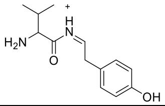	C13H19N2O2$^+$	235.1441			235.1438	-1.11		
g	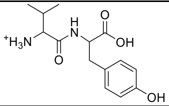	C14H21N2O4$^+$	281.1496			281.1491	-1.66		
h	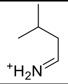	C5H12N$^+$	86.0964					86.0962	-2.81
i	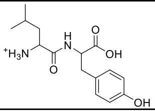	C15H23N2O4$^+$	295.1652					295.1649	-1.11

Table S22. Met-Tyr, Phe-Tyr, and Tyr-Tyr dipeptide fragments

Fragment				Met-Tyr		Phe-Tyr		Tyr-Tyr	
ID	Structure	Formula	Calc m/z	Obs m/z	Δppm	Obs m/z	Δppm	Obs m/z	Δppm
a	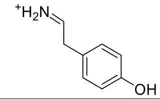	C8H10NO$^+$	136.0757	136.0755	-1.66	136.0756	-0.72	136.0756	-1.02
c	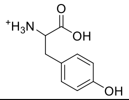	C9H12NO3$^+$	182.0812	182.0809	-1.63	182.0811	-0.39	182.0810	-0.86
j	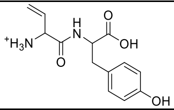	C13H17N2O4$^+$	265.1183	265.1177	-2.05				
k	*l* – NH3	C14H18NO4S$^+$	296.0951	296.0949	-0.67				
l	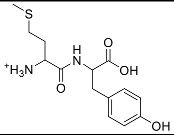	C14H21N2O4S$^+$	313.1217	313.1210	-1.99				
m	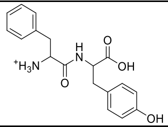	C18H21N2O4$^+$	329.1496			329.1493	-0.98		
n	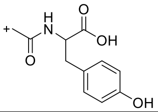	C11H12NO4$^+$	222.0761					222.0758	-1.4
o	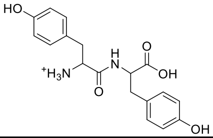	C18H21N2O5$^+$	345.1445					345.1441	-1.13

Table S23. Ser-Tyr, Gly-Tyr, and Pro-Tyr dipeptide fragments

Fragment				Ser-Tyr		Gly-Tyr		Pro-Tyr	
ID	Structure	Formula	Calc m/z	Obs m/z	Δppm	Obs m/z	Δppm	Obs m/z	Δppm
a	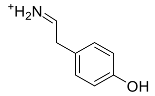	C8H10NO^{+}	136.0757			136.0755	-1.46	136.0755	-1.48
b	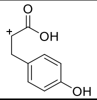	C9H9O3^{+}	165.0546			165.0544	-1.10	165.0544	-1.12
c	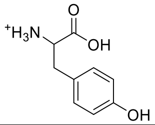	C9H12NO3^{+}	182.0812	182.0808	-1.89	182.0808	-2.10	182.0809	-1.64
p	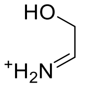	C2H6NO^{+}	60.0444	60.0447	4.41				
q	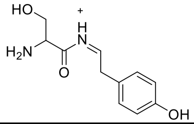	C11H15N2O3^{+}	223.1077	223.1072	-2.51				
r	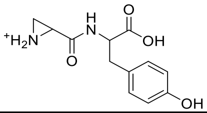	C12H15N2O4^{+}	251.1026	251.1020	-2.60				
s	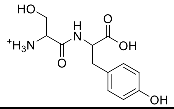	C12H17N2O5^{+}	269.1132	269.1124	-3.04				
t	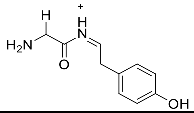	C10H13N2O2^{+}	193.0972			193.0968	-0.17		
u	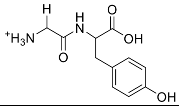	C11H15N2O4^{+}	239.1026			239.1023	-1.39		
v	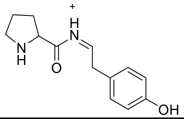	C13H17N2O2^{+}	233.1285					233.1279	-2.43
w	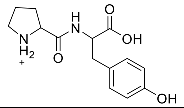	C14H19N2O4^{+}	279.1339					279.1333	-2.31

Table S24. Ala-Trp, Val-Trp, Ile-Trp, and Leu-Trp dipeptide fragments

Fragment				Ala-Trp		Val-Trp		Ile-Trp		Leu-Trp	
ID	Structure	Formula	Calc m/z	Obs m/z	Δppm	Obs m/z	Δppm	Obs m/z	Δppm	Obs m/z	Δppm
a	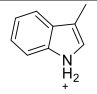	$C9H10N^+$	132.0808			132.0806	-1.26	132.0806	-1.68	132.0807	-0.84
b	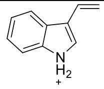	$C10H10N^+$	144.0808			144.0806	-0.89	144.0806	-0.99	144.0806	-0.94
c	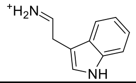	$C10H11N2^+$	159.0917			159.0915	-1.12	159.0914	-1.91	159.0914	-1.51
d	*e* – H2O	$C11H8NO^+$	170.0600					170.0597	-1.73	170.0598	-1.37
e	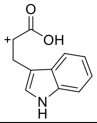	$C11H10NO2^+$	188.0706	188.0704	-0.97	188.0703	-1.69	188.0704	-0.84	188.0704	-1.13
f	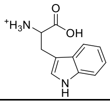	$C11H13N2O2^+$	205.0972	205.0969	-1.02	205.0969	-1.14	205.0967	-2.06	205.0968	-1.79
g	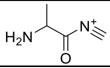	$C4H7N2O^+$	99.0553	99.0551	-1.42						
h	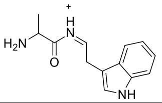	$C13H16N3O^+$	230.1288	230.1284	-1.51						
i	*i* – H2O	$C14H16N3O2^+$	258.1237	258.1234	-1.34						
j	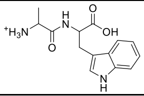	$C14H18N3O3^+$	276.1343	276.1338	-1.58						
k	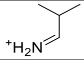	$C4H10N^+$	72.0808			72.0807	-0.50				
l	*l* – NH3	$C16H19N2O3^+$	287.1390			287.1385	-1.71				
m	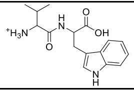	$C16H22N3O3^+$	304.1656			304.1652	-1.24				
n	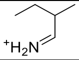	$C5H12N^+$	86.0964					86.0962	-2.07		
o	*o* – CO	$C16H21N2O2^+$	273.1598					273.1591	-2.51		
p	*q* – NH3	$C17H21N2O3^+$	301.1457					301.1541	-2.03		
q	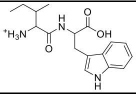	$C17H24N3O3^+$	318.1812					318.1809	-1.12		
r	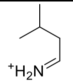	$C5H12N^+$	86.0964							86.0963	-1.76
s	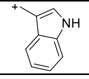	$C9H8N^+$	130.0651							130.0650	-0.84
t	*u* – NH3	$C17H21N2O3^+$	301.1547							301.1542	-1.68
u	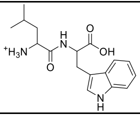	$C17H24N3O3^+$	318.1812							318.1810	-0.62

Table S25. Met-Trp, Phe-Trp, Tyr-Trp, and Trp-Trp dipeptide fragments

Fragment				Met-Trp		Phe-Trp		Tyr-Trp		Trp-Trp	
ID	Structure	Formula	Calc m/z	Obs m/z	Δppm	Obs m/z	Δppm	Obs m/z	Δppm	Obs m/z	Δppm
a	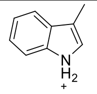	C9H10N$^+$	132.0808	132.0805	-2.36	132.0806	-1.36	132.0807	-0.83	132.0806	-1.61
b	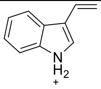	C10H10N$^+$	144.0808			144.0807	-0.61	144.0806	-0.91		
c	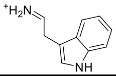	C10H11N2$^+$	159.0917	159.0914	-1.85	159.0913	-2.27	159.0914	-1.47	159.0914	-1.92
d	*e* – H2O	C11H8NO$^+$	170.0600	170.0597	-2.01					170.0597	-2.14
e	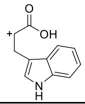	C11H10NO2$^+$	188.0706	188.0707	0.73	188.0704	-0.83	188.0705	-0.48		
f	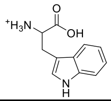	C11H13N2O2$^+$	205.0972	205.0969	-1.41	205.0968	-1.6	205.0970	-0.85		
s	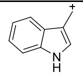	C9H8N$^+$	130.0651	130.0649	-2.12	130.0649	-1.36	130.0650	-0.70	130.0649	-1.89
v	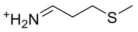	C4H10NS$^+$	104.0529	104.0526	-2.66						
w	*x* – NH3	C16H19N2O3S$^+$	319.1111	319.1102	-2.94						
x	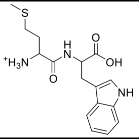	C16H22N3O3S$^+$	336.1376	336.1370	-1.83						
y	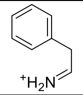	C8H10N$^+$	120.0808			120.0806	-1.49				
z	*aa* – NH3	C20H19N2O3$^+$	335.1390			335.1382	-2.38				
aa	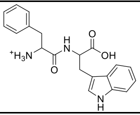	C20H22N3O3$^+$	352.1656			352.1648	-2.22				
ab	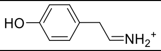	C8H10NO$^+$	136.0757					136.0755	-1.06		
ac	*ad* – NH3	C20H19N2O4$^+$	351.1339					351.1330	-2.63		
ad	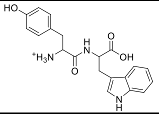	C20H22N3O4$^+$	368.1605					368.1595	-2.70		
ae	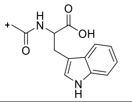	C13H13N2O3$^+$	245.0921							245.0915	-2.17
af	*ag* – NH3	C22H20N3O3$^+$	374.1499							374.1486	-3.50
ag	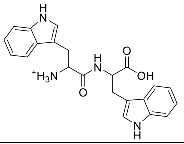	C22H23N4O3$^+$	391.1765							391.1776	3.01

Table S26. Ser-Trp, Thr-Trp, Gly-Trp, and Pro-Trp dipeptide fragments

Fragment				Ser-Trp		Thr-Trp		Gly-Trp		Pro-Trp	
ID	Structure	Formula	Calc m/z	Obs m/z	Δppm	Obs m/z	Δppm	Obs m/z	Δppm	Obs m/z	Δppm
a	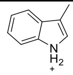	C9H10N$^+$	132.0808	132.0806	-1.70			132.0805	-2.39		
c	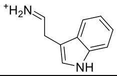	C10H11N2$^+$	159.0917	159.0913	-2.27						
e	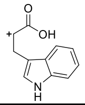	C11H10NO2$^+$	188.0706	188.0703	-1.82	188.0703	-1.39	188.0703	-1.57	188.0703	-1.83
f	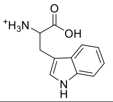	C11H13N2O2$^+$	205.0972	205.0968	-1.95	205.0969	-1.32	205.0969	-1.27	205.0969	-1.21
s	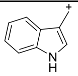	C9H8N$^+$	130.0651	130.0650	-1.35			130.0650	-1.04		
ah	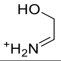	C2H6NO$^+$	60.0444	60.0447	4.74						
ai	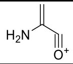	C3H4NO$^+$	70.0287	70.0287	-0.33						
aj	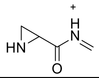	C4H7N2O2$^+$	115.0502	115.0500	-1.67						
ak	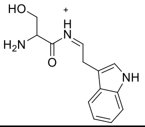	C13H16N3O2$^+$	246.1237	246.1232	-1.86						
al	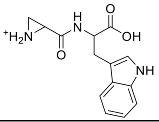	C14H16N3O3$^+$	274.1186	274.1179	-2.58						
am	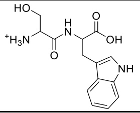	C14H18N3O4$^+$	292.1292	292.1284	-2.56						
an	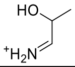	C3H8NO$^+$	74.0600			74.0599	-2.06				
ao	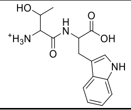	C15H20N3O4$^+$	306.1448			306.1441	-2.27				
ap	*aq* – CO – H2O	C12H14N3O$^+$	216.1137					216.1127	-4.57		
aq	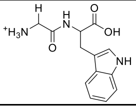	C13H17N3O3$^+$	262.1186					262.1181	-1.89		
ar	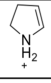	C4H8N$^+$	70.0651							70.0652	0.74
as	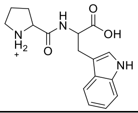	C16H20N3O3$^+$	302.1499							302.1494	-1.67

Table S27. Ala-Ser, Ser-Ser, Glu-Ser, and Gly-Ser dipeptide fragments

Fragment				Ala-Ser		Ser-Ser		Glu-Ser		Gly-Ser	
ID	Structure	Formula	Calc m/z	Obs m/z	Δppm	Obs m/z	Δppm	Obs m/z	Δppm	Obs m/z	Δppm
a	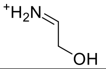	C2H6NO$^+$	60.0444	60.0447	5.89	60.0447	5.76	60.0447	4.38	60.0447	4.89
b	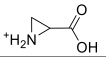	C3H6NO2$^+$	88.0393					88.0391	-2.60	88.0393	0.02
c	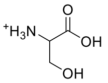	C3H8NO3$^+$	106.0499	106.0498	-0.96	106.0498	-0.97	106.0496	-2.35	106.0498	-0.25
d	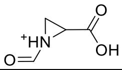	C4H6NO3$^+$	116.0342	116.0341	-0.87					116.0342	-0.15
e	*f* – H2O	C6H11N2O3$^+$	159.0764	159.0763	-0.61						
f	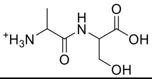	C6H13N2O4$^+$	177.0870	177.0869	-0.65						
g	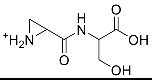	C6H11N2O4$^+$	175.0713			175.0712	-0.76				
h	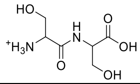	C6H13N2O5$^+$	193.0819			193.0818	-0.74				
i	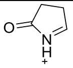	C4H6NO$^+$	84.0444					84.0442	-2.52		
j	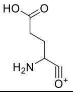	C5H8NO3$^+$	130.0499					130.0496	-2.16		
k	*l* – H2O	C8H13N2O5$^+$	217.0819					217.0814	-2.07		
l	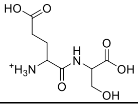	C8H15N2O6$^+$	235.0925					235.0917	-3.35		
m	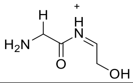	C4H9N2O2$^+$	117.0659							117.0658	-0.43
n	*o* – H2O	C5H9N2O3$^+$	145.0608							145.0607	-0.27
o	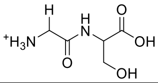	C5H11N2O4$^+$	163.0713							163.0712	-0.74

Table S28. Ala-Thr, Ser-Thr, Gly-Thr, and Pro-Thr dipeptide fragments

Fragment				Ala-Thr		Ser-Thr		Gly-Thr		Pro-Thr	
ID	Structure	Formula	Calc m/z	Obs m/z	Δppm	Obs m/z	Δppm	Obs m/z	Δppm	Obs m/z	Δppm
a	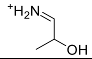	$C3H8NO^+$	74.0600	74.0600	-0.42	74.0599	-1.93	74.0600	-0.69		
b	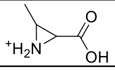	$C4H8NO2^+$	102.0550	102.0549	-0.58	102.0548	-1.61	102.0549	-0.78		
c	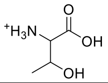	$C4H10NO3^+$	120.0655	120.0655	-0.50	120.0653	-1.74	120.0655	-0.20	120.0652	-2.54
d	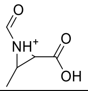	$C5H8NO3^+$	130.0499	130.0498	-0.15	130.0497	-1.48	130.0499	0.25	130.0496	-1.92
e	*f* – H2O	$C7H13N2O3^+$	173.0921	173.0921	-0.05						
f	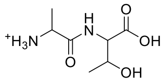	$C7H15N2O4^+$	191.1026	191.1025	-0.90						
g	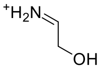	$C2H6NO^+$	60.0444			60.0447	5.07				
h	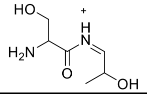	$C6H13N2O3^+$	161.0921			161.0919	-1.15				
i	*k* – H2O	$C7H13N2O4^+$	189.0870			189.0866	-1.92				
j	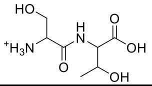	$C7H15N2O5^+$	207.0976			207.0972	-1.68				
k	*l* – H2O	$C6H11N2O3^+$	159.0764					159.0763	-0.58		
l	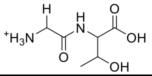	$C6H13N2O4^+$	177.0870					177.0871	0.43		
m	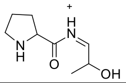	$C8H15N2O2^+$	171.1128							171.1126	-1.31
n	*o* – H2O	$C9H15N2O3^+$	199.1077							199.1073	-2.07
o	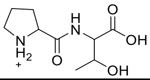	$C9H17N2O4^+$	217.1183							217.1178	-2.34

Table S29. aThr-(4-^{13}C; 2,3-D_2 Thr) dipeptide fragments

Fragment				aThr-(Thr-[4-^{13}C; 2,3-D_2])	
ID	Structure	Formula	Calc m/z	Obs m/z	Δppm
p	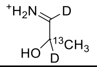	C2^{13}CH6D2NO^{+}	77.0760	77.0760	0.12
q	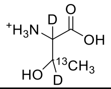	C3^{13}CH8D2NO3^{+}	123.0814	123.0814	-0.19
r	*t* – H2O	C7^{13}CH13D2N2O4^{+}	206.1185	206.1186	0.51
s	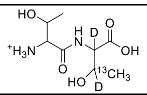	C7^{13}CH15D2N2O5^{+}	224.1291	224.1286	-2.27

Table S30. Ala-aThr, Ser-aThr, (4-^{13}C; 2,3-D_2 Thr)-aThr, and aThr-aThr dipeptide fragments

Fragment				Ala-aThr		Ser-aThr		*Thr-aThr		aThr-aThr	
ID	Structure	Formula	Calc m/z	Obs m/z	Δppm	Obs m/z	Δppm	Obs m/z	Δppm	Obs m/z	Δppm
a	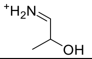	$C3H8NO^{+}$	74.0600	74.0600	-0.60			74.0601	0.24	74.0599	-1.98
b	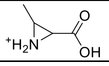	$C4H8NO2^{+}$	102.0550	102.0549	-0.72	102.0549	-0.56	102.0550	0.85		
c	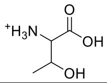	$C4H10NO3^{+}$	120.0655	120.0655	-0.40	120.0655	-0.27	120.0656	0.56	120.0653	-2.06
d	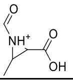	$C5H8NO3^{+}$	130.0499	130.0498	-0.28	130.0498	-0.26				
e	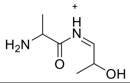	$C6H13N2O2^{+}$	145.0972	145.0971	-0.65						
f	*f* – H2O	$C7H13N2O3^{+}$	173.0921	173.0920	-0.55						
g	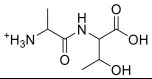	$C7H15N2O4^{+}$	191.1026	191.1025	-0.87						
h	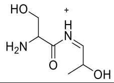	$C6H13N2O3^{+}$	161.0921			161.0920	-0.56				
i	*k* – H2O	$C7H13N2O4^{+}$	189.0870			189.0868	-1.01				
j	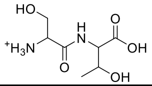	$C7H15N2O5^{+}$	207.0976			207.0973	-1.28				
k	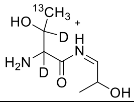	$C6^{13}CH13D2N2O3^{+}$	178.1236					178.1236	-0.32		
l	*m* – H2O	$C7^{13}C\ H13D2N2O4^{+}$	206.1185					206.1186	0.51		
m	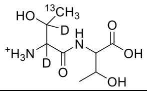	$C7^{13}C\ H15D2N2O5^{+}$	224.1291					224.1286	-2.27		
n	*o* – H2O	$C8H15N2O4^{+}$	203.1026							203.1022	-2.21
o	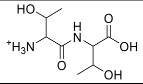	$C8H17N2O5^{+}$	221.1132							221.1127	-2.28

Table S31. Glu-aThr and Gly-aThr dipeptide fragments

Fragment				Glu-aThr		Gly-aThr	
ID	Structure	Formula	Calc m/z	Obs m/z	Δppm	Obs m/z	Δppm
a	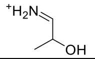	C3H8NO$^+$	74.0600	74.0599	-1.28	74.0599	-1.69
b	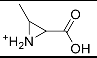	C4H8NO2$^+$	102.0550	102.0548	-1.44	102.0548	-1.32
c	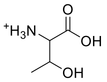	C4H10NO3$^+$	120.0655	120.0654	-1.37	120.0654	-1.40
d	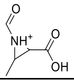	C5H8NO3$^+$	130.0499	130.0497	-1.00		
p	*q* – H2O	C9H15N2O5$^+$	231.0978	231.0973	-1.24		
q	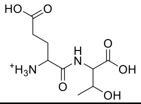	C9H17N2O6$^+$	249.1081	249.1078	-1.09		
r	*s* – H2O	C6H11N2O3$^+$	159.0764			159.0762	-1.37
s	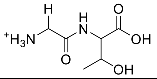	C6H13N2O4$^+$	177.0870			177.0867	-1.47

Table S32. Ala-Asn, Ser-Asn, Asp-Asn, and Pro-Asn dipeptide fragments

Fragment				Ala-Asn		Ser-Asn		Asp-Asn		Pro-Asn	
ID	Structure	Formula	Calc m/z	Obs m/z	Δppm	Obs m/z	Δppm	Obs m/z	Δppm	Obs m/z	Δppm
a	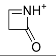	C3H4NO$^+$	70.0287					70.0288	0.27		
b	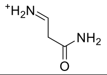	C3H7N2O$^+$	87.0553	87.0552	-1.15			87.0551	-1.60		
c	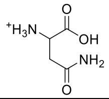	C4H9N2O3$^+$	133.0608	133.0606	-1.20	133.0605	-1.83	133.0606	-1.59	133.0605	-1.80
d	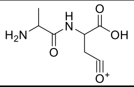	C7H11N2O4$^+$	187.0713	187.0711	-1.12						
e	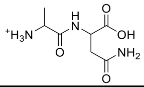	C7H14N3O4$^+$	204.0979	204.0977	-1.14						
f	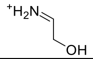	C2H6NO$^+$	60.0444			60.0447	4.85				
g	*h* – H_2O	C7H9N2O4$^+$	185.0557			185.0555	-0.98				
h	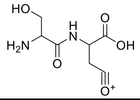	C7H11N2O5$^+$	203.0663			203.0659	-1.95				
i	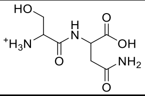	C7H14N3O5$^+$	220.0928			220.0923	-2.09				
j	*k* – NH3	C7H9N2O4$^+$	185.0557					185.0556	-0.52		
k	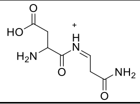	C7H12N3O4$^+$	202.0822					202.0819	-1.85		
l	*o* – (NH3$^+$H2O)	C8H9N2O5$^+$	213.0506					213.0498	-3.53		
m	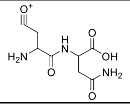	C8H12N3O5$^+$	230.0772					230.0766	-2.39		
n	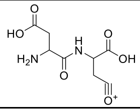	C8H11N2O6$^+$	231.0612					231.0608	-1.54		
o	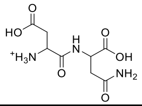	C8H14N3O6$^+$	248.0877					248.0874	-1.39		
p	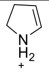	C4H8N$^+$	70.0651							70.0651	-0.09
q	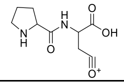	C9H13N2O4$^+$	213.0870							213.0867	-1.32
r	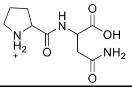	C9H16N3O4$^+$	230.1135							230.1130	-2.28

Table S33. Ala-Gln, Ser-Gln, Glu-Gln, and Pro-Gln dipeptide fragments

Fragment				Ala-Gln		Ser-Gln		Glu-Gln		Pro-Gln	
ID	Structure	Formula	Calc m/z	Obs m/z	Δppm	Obs m/z	Δppm	Obs m/z	Δppm	Obs m/z	Δppm
a	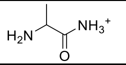	$C3H9N2O^{+}$	89.0709	89.0708	-1.35						
b	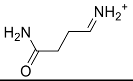	$C4H9N2O^{+}$	101.0709					101.0707	-2.13		
c	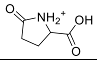	$C5H8NO3^{+}$	130.0499	130.0498	-0.70	130.0496	-2.28	130.0496	-1.81		
d	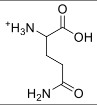	$C5H11N2O3^{+}$	147.0764	147.0762	-1.38	147.0760	-2.88	147.0760	-2.70	147.0761	-1.98
e	*f* – NH3	$C8H10NO4^{+}$	184.0604	184.0603	-0.84						
f	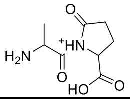	$C8H13N2O4^{+}$	201.0870	201.0867	-1.28						
g	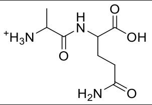	$C8H16N3O4^{+}$	218.1134	218.1133	-1.08						
h	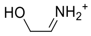	$C2H6NO^{+}$	60.0444			60.0446	3.92				
i	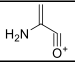	$C3H4NO^{+}$	70.0287			70.0287	-0.76				
j	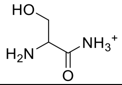	$C3H9N2O2^{+}$	105.0659			105.0656	-2.53				
k	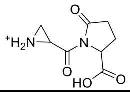	$C8H11N2O4^{+}$	199.0713			199.0708	-2.45				
l	*n* – H2O	$C8H14N3O4^{+}$	216.0979			216.0973	-2.53				
m	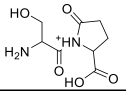	$C8H13N2O5^{+}$	217.0819			217.0814	-2.52				
n	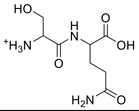	$C8H16N3O5^{+}$	234.1085			234.1080	-1.93				
o	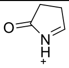	$C4H6NO^{+}$	84.0444					84.0443	-1.48		
p	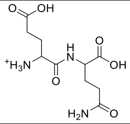	$C10H18N3O6^{+}$	276.1190					276.1183	-2.42		
q	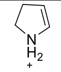	$C4H8N^{+}$	70.0651							70.0651	0.08
r	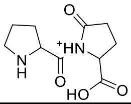	$C10H15N2O4^{+}$	227.1026							227.1022	-1.86
s	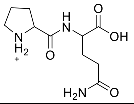	$C10H18N3O4^{+}$	244.1292							244.1286	-2.34

Table S34. Ala-Arg, Val-Arg, Ile-Arg, and Leu-Arg dipeptide fragments

Fragment				Ala-Arg		Val-Arg		Ile-Arg		Leu-Arg	
ID	Structure	Formula	Calc m/z	Obs m/z	Δppm	Obs m/z	Δppm	Obs m/z	Δppm	Obs m/z	Δppm
a	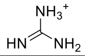	CH6N3$^+$	60.0556	60.0560	5.46	60.0559	4.24				
b	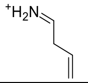	C4H8N$^+$	70.0652	70.0652	0.52	70.0651	-0.45	70.0651	-0.45	70.0651	-0.65
c	*b* + H_2O	C4H10NO$^+$	88.0757					88.0755	-2.25	88.0755	-2.52
d	*c* + H_2O	C4H12NO2$^+$	106.0863					106.0861	-1.78	106.0860	-2.14
e	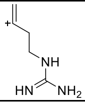	C5H10N3$^+$	112.0869								
f	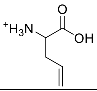	C5H10NO2$^+$	116.0706								
g	*h* – NH3	C6H12N3O2$^+$	158.0924	158.0922	-1.05						
h	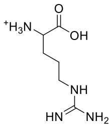	C6H15N4O2$^+$	175.1190	175.1188	-1.12	175.1187	-1.30	175.1187	-1.44	175.1186	-1.96
i	*j* – NH3	C9H17N4O3$^+$	229.1295	229.1292	-1.35						
j	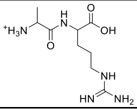	C9H20N5O3$^+$	246.1561	246.1555	-2.11						
k	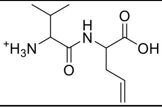	C10H19N2O3$^+$	215.1390			215.1388	-0.84				
l	*m* – H2O	C11H21N4O3$^+$	257.1608			257.1601	-2.94				
m	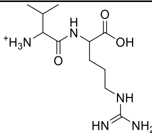	C11H24N5O3$^+$	274.1874			274.1866	-2.75				
n	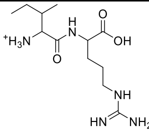	C12H26N5O3$^+$	288.2030					288.2023	-2.61		
o	*p* – NH3	C12H23N4O3$^+$	271.1765							271.1758	-2.28
p	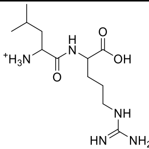	C12H26N5O3$^+$	288.2030							288.2022	-2.85

Table S35. Met-Arg, Phe-Arg, Tyr-Arg, and Trp-Arg dipeptide fragments

Fragment				Met-Arg		Phe-Arg		Tyr-Arg		Trp-Arg	
ID	Structure	Formula	Calc m/z	Obs m/z	Δppm	Obs m/z	Δppm	Obs m/z	Δppm	Obs m/z	Δppm
a	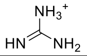	$CH6N3^+$	60.0556	60.0559	4.40	60.0559	4.89			60.0559	4.80
b	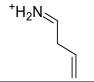	$C4H8N^+$	70.0652	70.0651	-0.25	70.0652	0.56	70.0652	0.49	70.0651	0.21
h	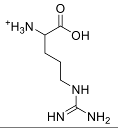	$C6H15N4O2^+$	175.1190	175.1187	-1.24	175.1189	-0.54	175.1189	-0.20	175.1188	-0.77
q	*r* – NH3	$C11H21N4O3S^+$	289.1329	289.1325	-1.38						
r	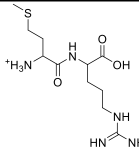	$C11H24N5O3S^+$	306.1594	306.1586	-2.65						
s	*t* – NH3	$C15H21N4O3^+$	305.1608			305.1604	-1.53				
t	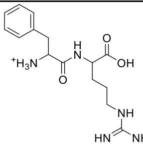	$C15H24N5O3^+$	322.1874			322.1869	-1.42				
u	*w* – NH3	$C15H21N4O4^+$	321.1557					321.1552	-1.53		
v	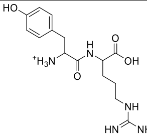	$C15H24N5O4^+$	338.1823					338.1815	-2.46		
w	*h* – H2O	$C6H13N4O^+$	157.1084							157.1082	-1.20
x	*z* – NH3	$C17H22N5O3^+$	344.1717							344.1710	-2.14
y	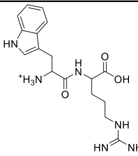	$C17H25N6O3^+$	361.1983							361.1974	-2.51

Table S36. Ser-Arg, Thr-Arg, aThr-Arg, and Gln-Arg dipeptide fragments

Fragment				Ser-Arg		Thr-Arg		aThr-Arg		Gln-Arg	
ID	Structure	Formula	Calc m/z	Obs m/z	Δppm	Obs m/z	Δppm	Obs m/z	Δppm	Obs m/z	Δppm
a	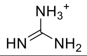	CH6N3$^+$	60.0556	60.0559	4.11	60.0559	4.47	60.0559	4.33		
b	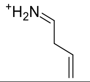	C4H8N$^+$	70.0652	70.0651	-0.76	70.0652	0.128	70.0652	1.42	70.0652	0.84
f	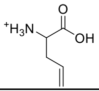	C5H10NO2$^+$	116.0706			116.0705	-0.66			116.0707	0.51
g	*h* – NH3	C6H12N3O2$^+$	158.0924	158.0920	-2.39	158.0925	-0.26				
h	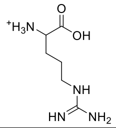	C6H15N4O2$^+$	175.1190	175.1185	-2.50	175.1190	0.48	175.119	0.39	175.1190	-0.02
z	*ab* – NH3	C9H17N4O4$^+$	245.1244	245.1237	-2.79						
aa	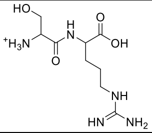	C9H18N5O5$^+$	262.1510	262.1502	-3.00						
ab	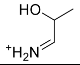	C3H8NO$^+$	74.0600			74.0601	0.44	74.0600	-0.01		
ac	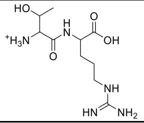	C10H22N5O4$^+$	276.1666			276.1658	-2.93	276.1659	-2.62		
ad	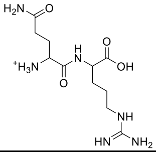	C11H23N6O4$^+$	303.1775							303.1772	-1.06

Table S37. Asp-Arg, Gly-Arg, and Pro-Arg dipeptide fragments

Fragment				Asp-Arg		Gly-Arg		Pro-Arg	
ID	Structure	Formula	Calc m/z	Obs m/z	Δppm	Obs m/z	Δppm	Obs m/z	Δppm
a	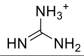	CH6N3$^+$	60.0556	60.0559	4.37	60.0559	4.03		
b	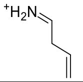	C4H8N$^+$	70.0652	70.0651	-0.06	70.0651	-0.33	70.0651	-0.76
e	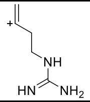	C5H10N3$^+$	112.0869			112.0867	-2.31		
h	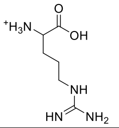	C6H15N4O2$^+$	175.1190	175.1186	-2.03	175.1187	-1.45	175.1187	-1.39
ae	*ag* – NH3	C10H17N4O5$^+$	273.1194	273.1188	-1.94				
af	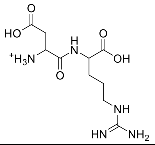	C10H20N5O5$^+$	290.1459	290.1450	-3.21				
ag	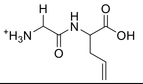	C7H13N2O3$^+$	173.0921			173.0918	-1.61		
ah	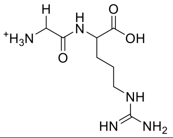	C8H18N5O3$^+$	232.1404			232.1399	-2.15		
ai	*aj* – NH3	C11H19N4O3$^+$	255.1452					255.1444	-2.99
aj	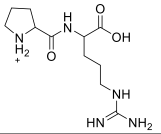	C11H22N5O3$^+$	272.1717					272.1709	-3.14

Table S38. Ala-His, Val-His, Ile-His, and Leu-His dipeptide fragments

Fragment				Ala-His		Val-His		Ile-His		Leu-His	
ID	Structure	Formula	Calc m/z	Obs m/z	Δppm	Obs m/z	Δppm	Obs m/z	Δppm	Obs m/z	Δppm
a	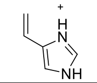	$C5H7N2^{+}$	95.0604								
b	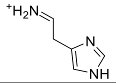	$C5H8N3^{+}$	110.0713	110.0712	-0.98	110.0710	-2.19	110.0711	-1.92	110.0710	-2.28
c	*d* – H2O	$C6H8N3O^{+}$	138.0662			138.0659	-1.76	138.0661	-0.79	138.0660	-1.43
d	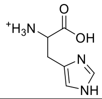	$C6H10N3O2^{+}$	156.0768	156.0766	-0.70	156.0766	-0.70	156.0767	-0.58	156.0766	-0.92
e	*c* + CO	$C7H8N3O2^{+}$	166.0611	166.0610	-0.92						
f	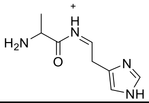	$C8H13N4O^{+}$	181.1084	181.1082	-1.20						
g	*h* – NH3	$C9H10N3O2^{+}$	192.0768	192.0765	-1.43						
h	*i* – H2O	$C9H13N4O2^{+}$	209.1033	209.1031	-1.04						
i	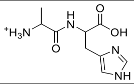	$C9H15N4O3^{+}$	227.1139	227.1135	-1.74						
j	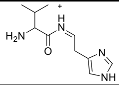	$C10H17N4O^{+}$	209.1397			209.1392	-0.70				
k	*l* – H2O	$C11H17N4O2^{+}$	237.1346			237.1341	-2.24				
l	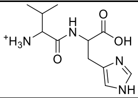	$C11H19N4O3^{+}$	255.1452			255.1445	-2.43				
m	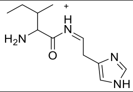	$C11H19N4O^{+}$	223.1553					223.1549	-1.78		
n	*o* – H2O	$C12H19N4O2^{+}$	251.1503					251.1498	-1.95		
o	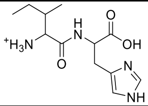	$C12H21N4O3^{+}$	269.1608					269.1602	-2.21		
p	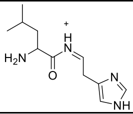	$C11H19N4O^{+}$	223.1553							223.1551	-1.07
q	*r* – H2O	$C12H19N4O2^{+}$	251.1503							251.1497	-2.04
r	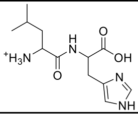	$C12H21N4O3^{+}$	269.1608							269.1602	-2.33

Table S39. Phe-His, Tyr-His, Trp-His, and Ser-His dipeptide fragments

Fragment				Phe-His		Tyr-His		Trp-His		Ser-His	
ID	Structure	Formula	Calc m/z	Obs m/z	Δppm	Obs m/z	Δppm	Obs m/z	Δppm	Obs m/z	Δppm
b	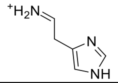	C5H8N3$^+$	110.0713	110.0711	-1.65	110.0711	-1.47	110.0711	-1.26	110.0711	-1.13
c	*d* – H2O	C6H8N3O$^+$	138.0662	138.0660	-1.50	138.0660	-1.09	138.0662	-0.19		
d	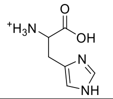	C6H10N3O2$^+$	156.0768	156.0766	-0.98	156.0766	-0.94	156.0767	-0.46	156.0766	-0.83
s	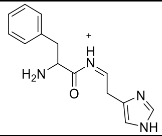	C14H17N4O$^+$	257.1397	257.1392	-1.73						
t	*u* – H2O	C15H17N4O2$^+$	285.1346	285.1341	-1.93						
u	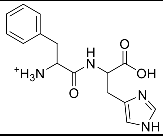	C15H19N4O3$^+$	303.1452	303.1444	-2.58						
v	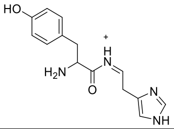	C14H17N4O2$^+$	273.1346			273.1341	-1.80				
w	*x* – H2O	C15H17N4O3$^+$	301.1295			301.1290	-1.81				
x	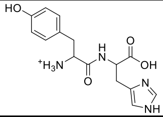	C15H19N4O4$^+$	319.1401			319.1394	-2.21				
y	*z* – H2O	C17H18N5O2$^+$	324.1455					324.1453	-0.73		
z	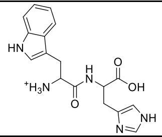	C17H20N5O3$^+$	342.1561					342.1555	-1.78		
aa	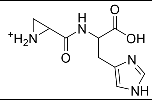	C9H13N4O3$^+$	225.0982							225.0978	-1.96
ab	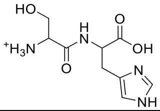	C9H15N4O4$^+$	243.1077							243.1083	-1.78

Table S40. Thr-His, aThr-His, Asp-His, and Glu-His dipeptide fragments

Fragment				Thr-His		aThr-His		Asp-His		Glu-His	
ID	Structure	Formula	Calc m/z	Obs m/z	Δppm	Obs m/z	Δppm	Obs m/z	Δppm	Obs m/z	Δppm
b	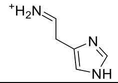	$C5H8N3^+$	110.0713	110.0711	-1.88	110.0710	-2.23	110.0711	-1.41	110.0712	-0.35
d	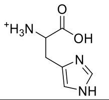	$C6H10N3O2^+$	156.0768	156.0767	-0.62	156.0766	-1.12	156.0767	-0.59	156.0768	0.13
ac	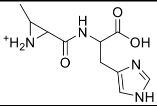	$C10H15N4O3^+$	239.1139	239.1132	-2.78	239.1133	-2.31				
ad	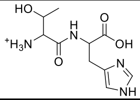	$C10H17N4O4^+$	257.1244	257.1237	-2.75	257.1238	-2.51				
ae	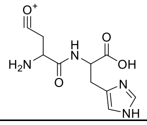	$C10H13N4O4^+$	253.0931					253.0927	-1.80		
af	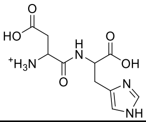	$C10H15N4O5^+$	271.1037					271.1034	-1.23		
ag	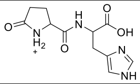	$C11H15N4O4^+$	267.1088							267.1087	-0.33
ah	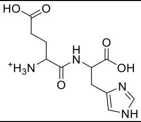	$C11H17N4O5^+$	285.1194							285.1190	-1.36

Table S41. Gly-His and Pro-His dipeptide fragments

Fragment				Gly-His		Pro-His	
ID	Structure	Formula	Calc m/z	Obs m/z	Δppm	Obs m/z	Δppm
a	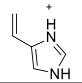	$C5H7N2^{+}$	95.0604	95.0601	-2.50		
b	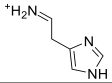	$C5H8N3^{+}$	110.0713	110.0710	-2.43	110.0711	-1.47
c	*d* – H2O	$C6H8N3O^{+}$	138.0662			138.0660	-1.50
d	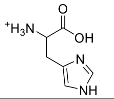	$C6H10N3O2^{+}$	156.0768			156.0766	-1.07
e	*c* + CO	$C7H8N3O2^{+}$	166.0611			166.0610	-0.60
ai	*ak* - CO	$C7H10N3O2^{+}$	168.0768	168.0764	-2.31		
aj	*al* – H2O	$C8H11N4O2^{+}$	195.0877	195.0873	-1.90		
ak	*al* – NH3	$C8H10N3O3^{+}$	196.0717	196.0712	-2.53		
al	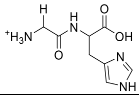	$C8H13N4O3^{+}$	213.0982	213.0978	-1.96		
am	*an* – H2O	$C11H15N4O2^{+}$	235.1190			235.1186	-1.66
an	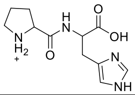	$C11H17N4O3^{+}$	253.1295			253.1289	-2.50

Table S42. Ala-Lys, Ile-Lys, Leu-Lys, and Phe-Lys dipeptide fragments

Fragment				Ala-Lys		Ile-Lys		Leu-Lys		Phe-Lys	
ID	Structure	Formula	Calc m/z	Obs m/z	Δppm	Obs m/z	Δppm	Obs m/z	Δppm	Obs m/z	Δppm
a	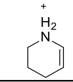	$C5H10N^+$	84.0808	84.0807	-0.87	84.0806	-1.99	84.0807	-1.06	84.0807	-1.20
b	*c* - CO	$C5H13N2^+$	101.1073	101.1072	-0.90					101.1072	-0.93
c	*e* – H2O	$C6H13N2O^+$	129.1022	129.1020	-1.49	129.1019	-2.72	129.1021	-1.37	129.1020	-2.10
d	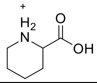	$C6H12NO2^+$	130.0863	130.0862	-0.77					130.0861	-0.85
e	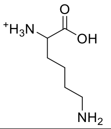	$C6H15N2O2^+$	147.1128	147.1126	-1.46	147.1125	-1.78	147.1126	-1.26	147.1126	-1.42
f	*h* – H2O	$C9H18N3O2^+$	200.1394	200.1391	-1.09						
g	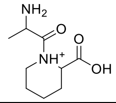	$C9H17N2O3^+$	201.1234	201.1231	-1.32						
h	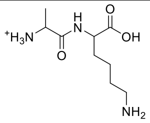	$C9H20N3O3^+$	218.1499	218.1496	-1.47						
i	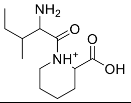	$C12H23N2O3^+$	243.1703			243.1689	-2.20				
j	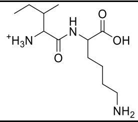	$C12H26N3O3^+$	260.1969			260.1961	-2.82				
k	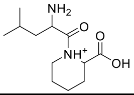	$C12H23N2O3^+$	243.1703					243.1700	-1.29		
l	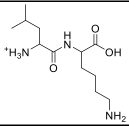	$C12H26N3O3^+$	260.1969					260.1964	-1.95		
m	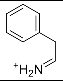	$C8H10N^+$	120.0808							120.0807	-0.87
n	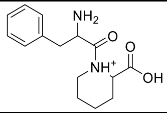	$C15H21N2O3^+$	277.1547							277.1543	-1.44
o	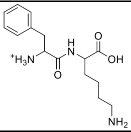	$C15H24N3O3^+$	294.1812							294.1807	-1.77

Table S43. Tyr-Lys, Trp-Lys, Ser-Lys, and Thr-Lys dipeptide fragments

Fragment				Tyr-Lys		Trp-Lys		Ser-Lys		Thr-Lys	
ID	Structure	Formula	Calc m/z	Obs m/z	Δppm	Obs m/z	Δppm	Obs m/z	Δppm	Obs m/z	Δppm
a	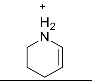	C5H10N$^+$	84.0808	84.0807	-1.22	84.0807	-1.04	84.0806	-1.54	84.0806	-1.94
b	*c* - CO	C5H13N2$^+$	101.1073	101.1072	-1.35	101.1072	-1.27	101.1072	-1.55	101.1071	-2.02
c	*e* – H2O	C6H13N2O$^+$	129.1022	129.1020	-2.16	129.1020	-2.05	129.1020	-1.84	129.1019	-2.71
d	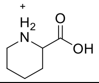	C6H12NO2$^+$	130.0863					130.0860	-1.60		
e	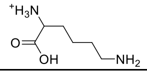	C6H15N2O2$^+$	147.1128	147.1126	-1.71	147.1126	-1.47	147.1125	-2.07	147.1125	-2.03
p	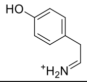	C8H10NO$^+$	136.0757	136.0756	-0.69						
q	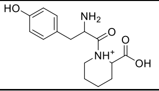	C15H21N2O4$^+$	293.1496	293.1492	-1.48						
r	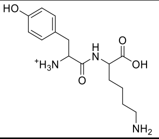	C15H24N3O4$^+$	310.1761	310.1754	-2.21						
s	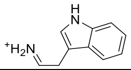	C10H11N2$^+$	159.0917			159.0916	-0.71				
t	*u* – CO	C16H22N3O2$^+$	288.1707			288.1704	-0.77				
u	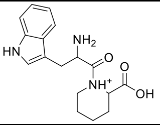	C17H22N3O3$^+$	316.1656			316.1650	-1.79				
v	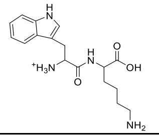	C17H25N4O3$^+$	333.1921			333.1914	-2.09				
w	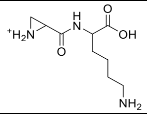	C9H18N3O3$^+$	216.1343					216.1338	-2.23		
x	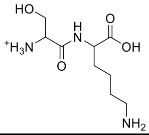	C9H20N3O4$^+$	234.1448					234.1443	-2.30		
y	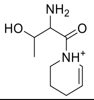	C9H17N2O2$^+$	185.1285							185.1280	-2.60
z	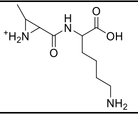	C10H20N3O3$^+$	230.1499							230.1492	-2.95
aa	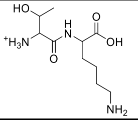	C10H22N3O4$^+$	248.1605							248.1599	-2.41

Table S44. aThr-Lys, Asn-Lys, Lys-Lys, and Asp-Lys dipeptide fragments

Fragment				aThr-Lys		Asn-Lys		Lys-Lys		Asp-Lys	
ID	Structure	Formula	Calc m/z	Obs m/z	Δppm	Obs m/z	Δppm	Obs m/z	Δppm	Obs m/z	Δppm
a	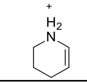	C5H10N$^+$	84.0808	84.0806	-1.80	84.0707	-0.66	84.0807	-1.00	84.0807	-1.18
b	*c* - CO	C5H13N2$^+$	101.1073	101.1072	-1.21			101.1072	-1.04		
c	*e* – H2O	C6H13N2O$^+$	129.1022	129.1019	-2.69	129.1021	-1.04	129.1020	-1.73	129.1020	-1.54
d	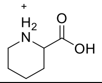	C6H12NO2$^+$	130.0863			130.0863	0.28	130.0861	-0.85		
e	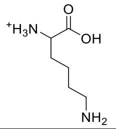	C6H15N2O2$^+$	147.1128	147.1125	-2.02	147.1127	-0.69	147.1126	-1.36	147.1126	-1.14
z	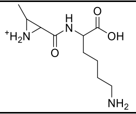	C10H20N3O3$^+$	230.1499	230.1492	-3.04						
aa	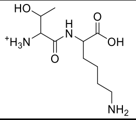	C10H22N3O4$^+$	248.1605	248.1598	-2.61						
ab	*z* – H2O	C10H18N3O2$^+$	212.1394	212.1890	-2.23						
ac	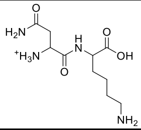	C10H21N4O4$^+$	261.1557			261.1554	-1.18				
ad	*ae* – NH3	C12H24N3O3$^+$	258.1812					258.1808	-1.48		
ae	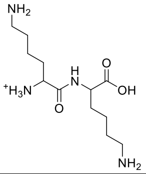	C12H27N4O3$^+$	275.2078					275.2076	-0.71		
af	*ah* – (2 H2O)	C10H16N3O3$^+$	226.1192							226.1182	-4.27
ag	*ah* – NH3	C10H17N2O5$^+$	245.1137							245.1127	-4.27
ah	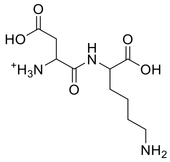	C10H20N3O5$^+$	262.1398							262.1392	-2.11

Table S45. Glu-Lys, Gly-Lys, and Pro-Lys dipeptide fragments

Fragment				Glu-Lys		Gly-Lys		Pro-Lys	
ID	Structure	Formula	Calc m/z	Obs m/z	Δppm	Obs m/z	Δppm	Obs m/z	Δppm
a	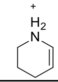	$C5H10N^+$	84.0808	84.0802	-1.32	84.0807	-1.42	84.0806	-1.54
b	*c* - CO	$C5H13N2^+$	101.1073			101.1072	-0.89	101.1072	-1.19
c	*e* – H2O	$C6H13N2O^+$	129.1022	129.1020	-1.49	129.1020	-1.57	129.1020	-2.19
d	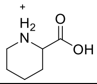	$C6H12NO2^+$	130.0863	130.0861	-1.33	130.0862	-0.72		
e	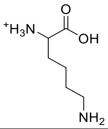	$C6H15N2O2^+$	147.1128	147.1126	-1.56	147.1127	-1.04	147.1126	-1.55
ai	*aj* – (H2O + CO)	$C9H15N2O^+$	167.1179	167.1176	-1.48				
aj	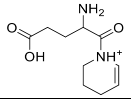	$C10H17N2O3^+$	213.1234	213.1230	-1.89				
ak	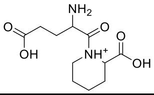	$C11H19N2O5^+$	259.1289	259.1284	-1.66				
al	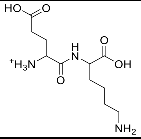	$C11H22N3O5^+$	276.1554	276.1548	-1.99				
am	*ao* – H2O	$C8H16N3O2^+$	186.1237			186.1236	-0.71		
an	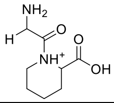	$C8H15N2O3^+$	187.1077			187.1075	-1.37		
ao	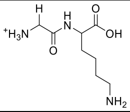	$C8H18N3O3^+$	204.1343			204.1339	-1.93		
ap	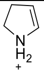	$C4H8N^+$	70.0651					70.0651	-0.12
aq	*ar* – H2O	$C11H20N3O2^+$	226.1540					226.1544	-2.47
ar	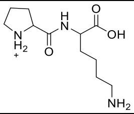	$C11H22N3O3^+$	244.1656					244.1650	-2.18

Table S46. Asp-Asp dipeptide fragments

Fragment				Asp-Asp	
ID	Structure	Formula	Calc m/z	Obs m/z	Δppm
a	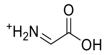	$C2H4N2O^+$	74.0237	74.0236	-1.11
b	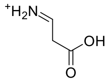	$C3H6NO2^+$	88.0393	88.0392	-1.23
c	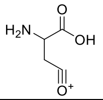	$C4H6NO3^+$	116.0342	116.0341	-0.98
d	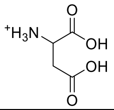	$C4H8NO4^+$	134.0448	134.0446	-1.42
e	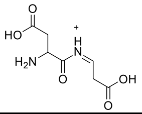	$C7H11N2O5^+$	203.0663	203.0660	-1.09
f	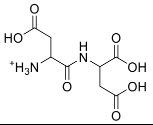	$C8H13N2O7^+$	249.0717	249.0711	-2.50

Table S47. Ala-Glu and Glu-Glu dipeptide fragments

Fragment				Ala-Glu		Glu-Glu	
ID	Structure	Formula	Calc m/z	Obs m/z	Δppm	Obs m/z	Δppm
a	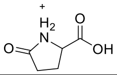	C5H8NO3^{+}	130.0499	130.0496	-1.72	130.0502	2.41
b	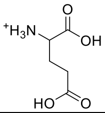	C5H10NO4^{+}	148.0604	148.0600	-2.97	148.0605	0.61
c	*d* – NH3	C8H12NO5^{+}	202.0710	202.0706	-2.22		
d	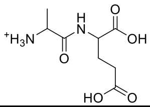	C8H15N2O5^{+}	219.0976	219.0970	-2.67		
e	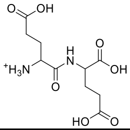	C10H17N2O7^{+}	277.1030			277.1027	-1.13

Table S48. Ala-Cys dipeptide fragments

Fragment				Ala-Cys	
ID	Structure	Formula	Calc m/z	Obs m/z	Δppm
a	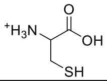	C3H8NO2S^{+}	122.0270	122.027	-0.58
b	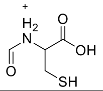 – H2O	C4H6NO2S^{+}	132.0114	132.0114	-0.29
c	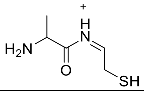	C5H11N2OS^{+}	147.0587	147.0586	-0.39
d	*e* – H2O	C6H11N2O2S^{+}	175.0536	175.0535	-0.27
e	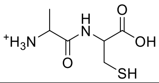	C6H13N2O3S^{+}	193.0641	193.0642	0.2

Table S49. Glu-Gly and Gly-Gly dipeptide fragments

Fragment				Glu-Gly		Gly-Gly	
ID	Structure	Formula	Calc m/z	Obs m/z	Δppm	Obs m/z	Δppm
a	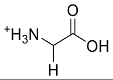	$C2H6NO2^{+}$	76.0393	76.0392	-1.93	76.0393	0.10
b	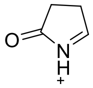	$C4H6NO^{+}$	84.0444	84.0442	-2.67		
c	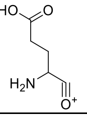	$C5H8NO3^{+}$	130.0499	130.0496	-2.32		
d	*e* – (H2O + CO)	$C6H8NO3^{+}$	142.0499	142.0496	-1.82		
e	*f* – NH3	$C7H10NO5^{+}$	188.0554	188.0550	-1.68		
f	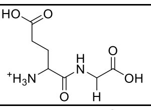	$C7H13N2O5^{+}$	205.0819	205.0817	-0.90		
g	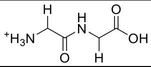	$C4H9N2O3^{+}$	133.0608			133.0608	-0.07

Table S50. aThr-Pro and His-Pro dipeptide fragments

Fragment				aThr-Pro		His-Pro	
ID	Structure	Formula	Calc m/z	Obs m/z	Δppm	Obs m/z	Δppm
a	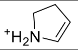	C4H8N$^+$	70.0651	70.0651	-0.06	70.0652	0.40
b	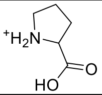	C5H10NO2$^+$	116.0706	116.0703	-2.38	116.0704	-1.35
c	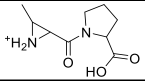	C9H15N2O3$^+$	199.1077	199.1073	-2.01		
d	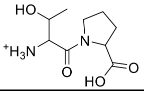	C9H17N2O4$^+$	217.1183	217.1178	-2.35		
e	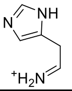	C5H8N3$^+$	110.0713			110.0711	-1.47
f	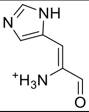	C6H8N3O$^+$	138.0662			138.0660	-1.50
g	*h* – H2O	C11H15N4O2$^+$	235.1190			235.1186	-1.66
h	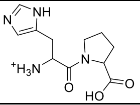	C11H17N4O3$^+$	253.1295			253.1289	-2.50

Table S51. PnAla-Ala-Ala, PnAla-Ala-Val, and PnAla-Ala-Ile tripeptide fragments

Fragment				PnAla-Ala-Ala		PnAla-Ala-Val		PnAla-Ala-Ile	
ID	Structure	Formula	Calc m/z	Obs m/z	Δppm	Obs m/z	Δppm	Obs m/z	Δppm
a	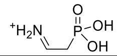	C2H7NO3P$^+$	124.0158	124.0155	-2.08	124.0155	-2.43	124.0155	-2.44
b	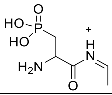	C5H12N2O4P$^+$	195.0529	195.0525	-2.00	195.0526	-1.89	195.0525	-2.05
c	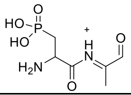	C6H12N2O5P$^+$	223.0478	223.0472	-2.84	223.0472	-2.66	223.0472	-2.66
d	*c* + H2O	C6H14N2O6P$^+$	241.0584	241.0578	-2.58	241.0577	-2.74	241.0577	-2.74
e	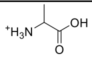	C3H8NO2$^+$	90.0550	90.0548	-1.67				
f	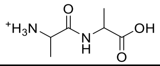	C6H13N2O3$^+$	161.0921	161.0917	-2.29				
g	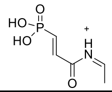	C5H9NO4P$^+$	178.0264	178.0260	-2.11				
h	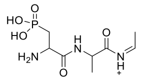	C8H17N3O5P$^+$	266.0900	266.0894	-2.33				
i	*j* – H2O	C9H15N3O5P$^+$	276.0744	276.0738	-2.09				
j	*k* – H2O	C9H17N3O6P$^+$	294.0850	294.0841	-2.86				
k	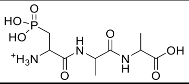	C9H19N3O7P$^+$	312.0955	312.0946	-2.84				
l	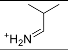	C4H10N$^+$	72.0808			72.0807	-1.11		
m	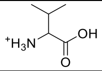	C5H12NO2$^+$	118.0863			118.0860	-2.43		
n	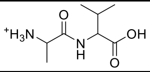	C8H17N2O3$^+$	189.1234			189.1230	-2.19		
o	*q* – H2O	C10H19N3O4P$^+$	276.1108			276.1100	-2.73		
p	*q* – NH3	C10H18N2O5P$^+$	277.0948			277.0941	-2.52		
q	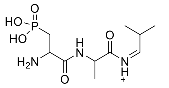	C10H21N3O5P$^+$	294.1213			294.1205	-2.79		
r	*s* – H2O	C11H19N3O5P$^+$	304.1057			304.1049	-2.51		
s	*t* – H2O	C11H21N3O6P$^+$	322.1153			322.1154	-2.58		
t	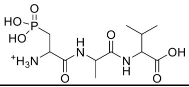	C11H23N3O7P$^+$	340.1268			340.1260	-2.37		
u	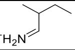	C5H12N$^+$	86.0964					86.0962	-2.55
v	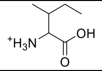	C6H14NO2$^+$	131.1019					132.1016	-2.25
w	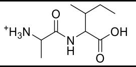	C9H19N2O3$^+$	203.1390					203.1386	-1.93
x	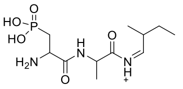	C11H23N3O5P$^+$	308.1370					308.1361	-2.74
y	*z* – H2O	C12H21N3O6P$^+$	318.1213					318.1205	-2.48
z	*aa* – H2O	C12H23N3O6P$^+$	336.1319					336.1309	-3.09
aa	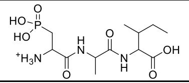	C12H25N3O7P$^+$	354.1425					354.1414	-2.86

Table S52. PnAla-Ala-Leu, PnAla-Ala-Met, and PnAla-Ala-Phe tripeptide fragments

Fragment				PnAla-Ala-Leu		PnAla-Ala-Met		PnAla-Ala-Phe	
ID	Structure	Formula	Calc m/z	Obs m/z	Δppm	Obs m/z	Δppm	Obs m/z	Δppm
a	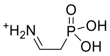	C2H7NO3P$^+$	124.0158	124.0156	-1.41	124.0156	-1.69	124.0156	-1.82
b	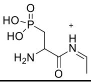	C5H12N2O4P$^+$	195.0529	195.0527	-1.23	195.0528	1.15	195.0527	-0.99
c	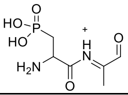	C6H12N2O5P$^+$	223.0478	223.0475	-1.62	223.0475	-1.63	223.0474	-1.73
d	*c* + H2O	C6H14N2O6P$^+$	241.0584	241.0580	-1.73				
ab	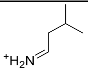	C5H12N$^+$	86.0964	86.0963	-1.76				
ac	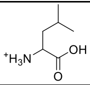	C6H14NO2$^+$	132.1019	132.1018	-0.81				
ad	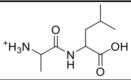	C9H19N2O3$^+$	203.1390	203.1387	-1.36				
ae	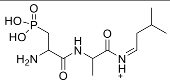	C11H23N3O5P$^+$	308.1370	308.1363	-2.09				
af	*ag* – H2O	C12H21N3O5P$^+$	318.1213	318.1209	-1.52				
ag	*ah* – H2O	C12H23N3O6P$^+$	336.1319	336.1310	-2.53				
ah	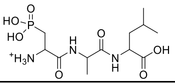	C12H25N3O7P$^+$	354.1425	354.1417	-2.13				
ai	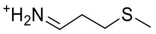	C4H10NS$^+$	104.0529			104.0527	-1.40		
aj	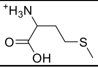	C5H12NO2S$^+$	150.0583			150.0582	-0.54		
ak	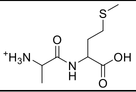	C8H17N2O3S$^+$	221.0954			221.0952	-1.31		
al	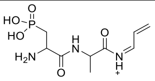	C9H17N3O5P$^+$	278.0900			278.0896	-1.65		
am	*am* – H2O	C11H19N3O5PS$^+$	336.0778			336.0772	-1.55		
an	*ao* – H2O	C11H21N3O6PS$^+$	354.0883			354.0877	-1.74		
ao	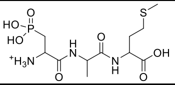	C11H23N3O7PS$^+$	372.0989			372.0985	-1.13		
ap	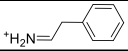	C8H10N$^+$	120.0808					120.0807	-0.79
aq	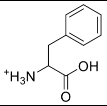	C9H12NO2$^+$	166.0863					166.0861	-0.90
ar	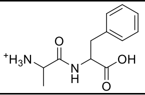	C12H17N2O3$^+$	237.1234					237.1229	-2.03
as	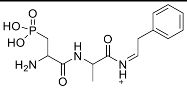	C14H21N3O5P$^+$	342.1213					342.1208	-1.51
at	*au* – H2O	C15H19N3O5P$^+$	352.1057					352.1050	-1.88
au	*av* – H2O	C15H21N3O6P$^+$	370.1163					370.1156	-1.62
av	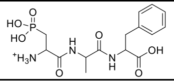	C15H23N3O7P$^+$	388.1268					388.1260	-1.98

Table S53. PnAla-Ala-Tyr, PnAla-Ala-Trp, and PnAla-Ala-Ser tripeptide fragments

Fragment				PnAla-Ala-Tyr		PnAla-Ala-Trp		PnAla-Ala-Ser	
ID	Structure	Formula	Calc m/z	Obs m/z	Δppm	Obs m/z	Δppm	Obs m/z	Δppm
a	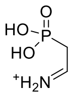	$C_2H_7NO_3P^+$	124.0158					124.0157	-0.67
b	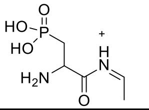	$C_5H_{12}N_2O_4P^+$	195.0529	195.0528	-0.48	195.0528	-0.72	195.0528	-0.64
c	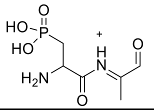	$C_6H_{12}N_2O_5P^+$	223.0478	223.0474	-1.97	223.0475	-0.17	223.0475	-1.30
aw	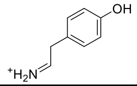	$C_8H_{10}NO^+$	136.0757	136.0757	0.41				
ax	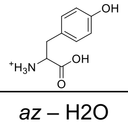	$C_9H_{12}NO_3^+$	182.0812	182.0812	0.24				
ay	*az* – H2O	$C_{15}H_{21}N_3O_7P^+$	386.1112	386.1102	-2.42				
az	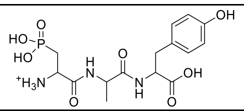	$C_{15}H_{23}N_3O_8P^+$	404.1217	404.1208	-2.39				
ba	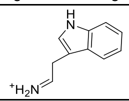	$C_{10}H_{11}N_2^+$	159.0917			159.0916	-0.19		
bb	*bc* – NH3	$C_{11}H_{10}N_2O^+$	188.0706			188.0705	-0.34		
bc	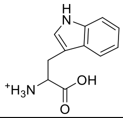	$C_{11}H_{13}N_2O_2^+$	205.0972			205.0970	-0.68		
bd	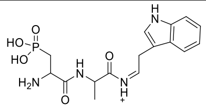	$C_{16}H_{22}N_4O_5P^+$	381.1322			381.1317	-1.28		
be	*bf* – H2O	$C_{17}H_{22}N_4O_6P^+$	409.1272			409.1267	-1.00		
bf	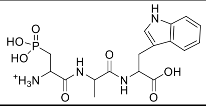	$C_{17}H_{24}N_4O_7P^+$	427.1377			427.1374	-0.68		
bg	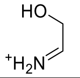	$C_2H_6NO^+$	60.0444					60.0447	5.29
bh	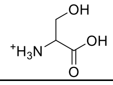	$C_3H_8NO_3^+$	106.0499					106.0498	-0.95
bi	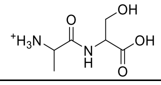	$C_6H_{13}N_2O_4^+$	177.0870					177.0869	-0.60
bj	*bk* – H2O	$C_9H_{17}N_3O_7P^+$	310.0799					310.0793	-1.81
bk	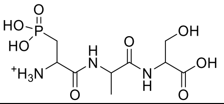	$C_9H_{19}N_3O_8P^+$	328.0904					328.0899	-1.60

Table S54. PnAla-Ala-Thr, PnAla-Ala-aThr, and PnAla-Ala-Asn tripeptide fragments

Fragment				PnAla-Ala-Thr		PnAla-Ala-aThr		PnAla-Ala-Asn	
ID	Structure	Formula	Calc m/z	Obs m/z	Δppm	Obs m/z	Δppm	Obs m/z	Δppm
a	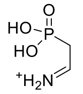	$C2H7NO3P^{+}$	124.0158	124.0157	-0.90	124.0157	-0.50		
b	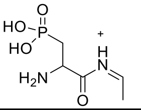	$C5H12N2O4P^{+}$	195.0529	195.0528	-0.53	195.0530	0.45	195.0528	-0.61
c	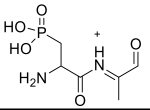	$C6H12N2O5P^{+}$	223.0478	223.0476	-1.16	223.0477	-0.58	223.0476	-1.27
bl	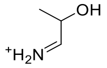	$C3H8NO^{+}$	74.0600	74.0600	-0.66	74.0601	0.19		
bm	*bn* – H2O	$C4H8NO2^{+}$	102.0550	102.0549	-0.76	102.0550	0.36		
bn	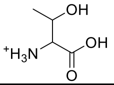	$C4H10NO3^{+}$	120.0655	120.0655	-0.49	120.0655	0.16		
bo	*bp* – H2O	$C7H13N2O3^{+}$	173.0921	173.0290	-0.30				
bp	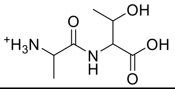	$C7H15N2O4^{+}$	191.1026	191.1025	-0.91				
bq	*br* – H2O	$C10H19N3O7P^{+}$	324.0955	324.0950	-1.45	324.0953	-0.77		
br	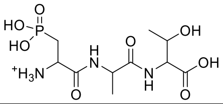	$C10H21N3O8P^{+}$	342.1061	342.1056	-1.37	342.1060	-0.17		
bs	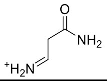	$C3H7N2O^{+}$	87.0553					87.0552	-0.75
bt	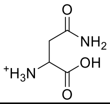	$C4H9N2O3^{+}$	133.0608					133.0606	-1.04
bu	*bv* – H2O	$C10H18N4O7P^{+}$	337.0908					337.0903	-1.50
bv	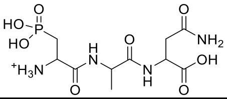	$C10H20N4O8P^{+}$	355.1013					355.1009	-1.14

Table S55. PnAla-Ala-Gln, PnAla-Ala-Arg, and PnAla-Ala-His tripeptide fragments

Fragment				PnAla-Ala-Gln		PnAla-Ala-Arg		PnAla-Ala-His	
ID	Structure	Formula	Calc m/z	Obs m/z	Δppm	Obs m/z	Δppm	Obs m/z	Δppm
b	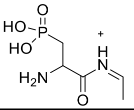	$C5H12N2O4P^+$	195.0529	195.0528	-0.54	195.0528	-0.56	195.0529	-0.35
c	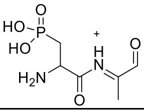	$C6H12N2O5P^+$	223.0478	223.0476	-1.18	223.0476	-1.23		
bw	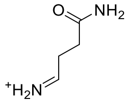	$C4H9N2O^+$	101.0709	101.0709	-0.64				
bx	*by* – NH3	$C5H8NO3^+$	130.0499	130.0498	-0.29				
by	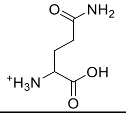	$C5H11N2O3^+$	147.0764	147.0763	-1.01				
bz	*ca* – H2O	$C11H20N4O7P^+$	351.1064	351.1060	-1.31				
ca	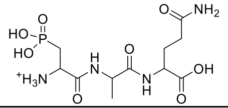	$C11H22N4O8P^+$	369.1170	369.1166	-1.15				
cb	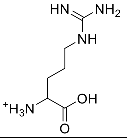	$C6H15N4O2^+$	175.1190			175.1189	-0.32		
cc	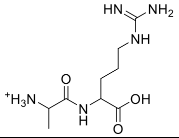	$C9H20N5O3^+$	246.1561			246.1558	-0.97		
cd	*cc* + CO – H2O	$C10H18N5O3^+$	256.1404			256.1401	-1.32		
ce	*cf* – H2O	$C12H24N6O6P^+$	379.1490			379.1483	-1.76		
cf	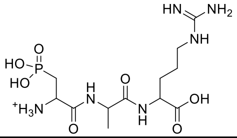	$C12H26N6O7P^+$	397.1595			397.1593	-0.50		
cg	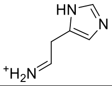	$C5H8N3^+$	110.0713					110.0712	-1.06
ch	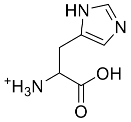	$C6H10N3O2^+$	156.0768					156.0767	-0.54
ci	*cj* – H2O	$C9H13N4O2^+$	209.1033					209.1031	-0.82
cj	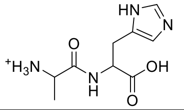	$C9H15N4O3^+$	227.1139					227.1136	-1.17
ck	*ci* + CO	$C10H13N4O3^+$	237.0982					237.0978	-1.58
cl	*cm* – H2O	$C12H17N5O5P^+$	342.0962					342.0959	-0.74
cm	*cn* – H2O	$C12H19N5O6P^+$	360.1068					360.1063	-1.36
cn	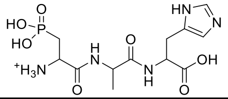	$C12H21N5O7P^+$	378.1173					378.1169	-1.15

Table S56. PnAla-Ala-Lys, PnAla-Ala-Glu, and PnAla-Ala-Gly tripeptide fragments

Fragment				PnAla-Ala-Lys		PnAla-Ala-Glu		PnAla-Ala-Gly	
ID	Structure	Formula	Calc m/z	Obs m/z	Δppm	Obs m/z	Δppm	Obs m/z	Δppm
a	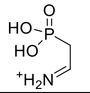	C2H7NO3P$^+$	124.0158	124.0157	-0.89			124.0159	0.74
b	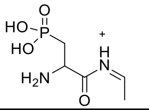	C5H12N2O4P$^+$	195.0529	195.0529	-0.21	195.0529	-0.10	195.0531	1.11
c	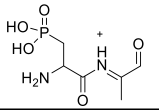	C6H12N2O5P$^+$	223.0478	223.0476	-1.21	223.0477	-0.74	223.0479	0.07
co	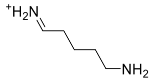	C5H10N$^+$	84.0808	84.0807	-0.57				
cp	*cr* – H2O	C6H13N2O$^+$	129.1022	129.1021	-1.22				
cq	*cr* – NH3	C6H12NO2$^+$	130.0863	130.0863	0.02				
cr	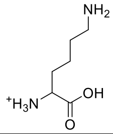	C6H15N2O2$^+$	147.1128	147.1127	-0.75				
cs	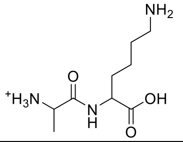	C9H20N3O3$^+$	218.1499	218.1498	-0.69				
ct	*cs* + CO – H2O	C10H18N3O3$^+$	228.1343	228.1341	-0.53				
cu	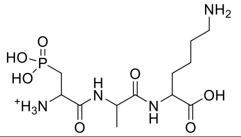	C12H26N4O7P$^+$	369.1534	369.1530	-1.11				
cv	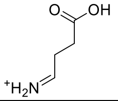	C4H8NO2$^+$	102.0550			102.0549	-0.83		
cw	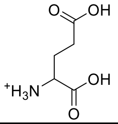	C5H10NO4$^+$	148.0604			148.0604	-0.47		
cx	*cy* – H2O	C11H17N3O7P$^+$	334.0799			334.0798	-0.28		
cy	*cz* – H2O	C11H19N3O8P$^+$	352.0904			352.0898	-1.65		
cz	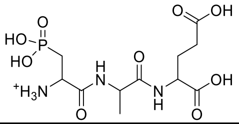	C11H21N3O9P$^+$	370.1010			370.1005	-1.25		
da	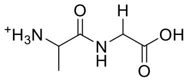	C5H11N2O3$^+$	147.0764					147.0765	0.55
db	*dc* – H2O	C8H15N3O6P$^+$	280.0693					280.0693	0.14
dc	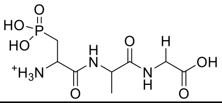	C8H17N3O7P$^+$	298.0799					298.0799	-0.02

Table S57. PnAla-Ala-Pro tripeptide fragments

Fragment				PnAla-Ala-Pro	
ID	Structure	Formula	Calc m/z	Obs m/z	Δppm
b	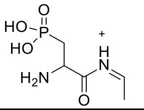	$C5H12N2O4P^+$	195.0529	195.0532	1.45
c	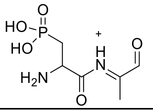	$C6H12N2O5P^+$	223.0478	223.0479	0.44
dd	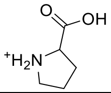	$C5H10NO2^+$	116.0706	116.0707	0.43
de	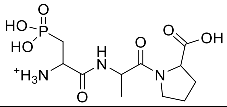	$C11H21N3O7P^+$	338.1112	338.1109	-0.92

Table S58. PnAla-Val-Val, PnAla-Val-Ile, and PnAla-Val-Leu tripeptide fragments

Fragment				PnAla-Val-Val		PnAla-Val-Ile		PnAla-Val-Leu	
ID	Structure	Formula	Calc m/z	Obs m/z	Δppm	Obs m/z	Δppm	Obs m/z	Δppm
a	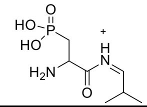	C7H16N2O4P⁺	223.0842	223.0838	-1.88	223.0841	-0.42	223.0843	0.14
b	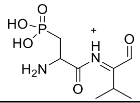	C8H16N2O5P⁺	251.0791	251.0786	-1.95	251.0789	-0.87	251.0791	0.04
c	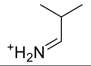	C4H10N⁺	72.0808	72.0807	-1.00				
d	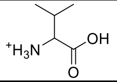	C5H12NO2⁺	118.0863	118.0860	-1.91				
e	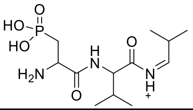	C12H25N3O5P⁺	322.1526	322.1520	-2.04				
f	*g* – H2O	C13H25N3O6P⁺	350.1476	350.1469	-1.91				
g	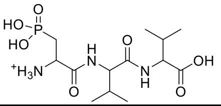	C13H27N3O7P⁺	368.1581	368.1574	-1.83				
h	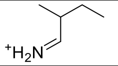	C5H12N⁺	86.0964			86.0963	-1.07		
i	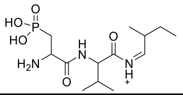	C13H27N3O5P⁺	336.1683			336.1689	1.69		
j	*k* – H2O	C14H27N3O6P⁺	364.1632			364.1629	-0.75		
k	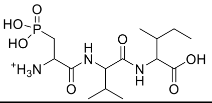	C14H29N3O7P⁺	382.1738			382.1743	1.29		
l	*m* – H2O	C14H27N3O6P⁺	364.1632					364.1630	-0.58
m	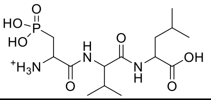	C14H29N3O7P⁺	382.1738					382.1734	-0.90

Table S59. PnAla-Val-Met, PnAla-Val-Phe, and PnAla-Val-Tyr tripeptide fragments

Fragment				PnAla-Val-Met		PnAla-Val-Phe		PnAla-Val-Tyr	
ID	Structure	Formula	Calc m/z	Obs m/z	Δppm	Obs m/z	Δppm	Obs m/z	Δppm
a	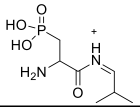	C7H16N2O4P$^+$	223.0842	223.0843	0.56	223.0842	0.12	223.0839	-1.30
b	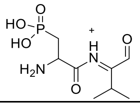	C8H16N2O5P$^+$	251.0791	251.0792	0.38	251.0791	-0.13	251.0789	-1.03
n	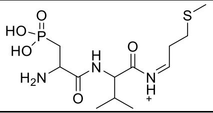	C12H25N3O5PS$^+$	354.1247	354.1243	-1.18				
o	*p* – H2O	C13H25N3O6PS$^+$	382.1196	382.1196	0.05				
p	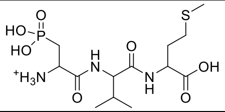	C13H27N3O7PS$^+$	400.1302	400.1301	-0.22				
q	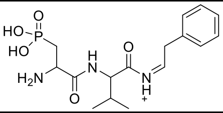	C16H25N3O5P$^+$	370.1526			370.1526	-0.10		
r	*s* – H2O	C17H25N3O6P$^+$	398.1476			398.1473	-0.65		
s	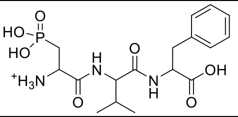	C17H27N3O6P$^+$	416.1581			416.1578	-0.84		
t	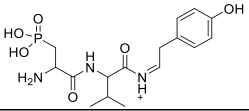	C16H25N3O6P$^+$	386.1476					386.1469	-1.56
u	*v* – H2O	C17H25N3O7P$^+$	414.1425					414.1413	-2.77
v	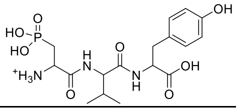	C17H27N3O8P$^+$	432.1530					432.1519	-2.72

Table S60. PnAla-Val-Trp, PnAla-Val-aThr, and PnAla-Val-Gln tripeptide fragments

Fragment				PnAla-Val-Trp		PnAla-Val-aThr		PnAla-Val-Gln	
ID	Structure	Formula	Calc m/z	Obs m/z	Δppm	Obs m/z	Δppm	Obs m/z	Δppm
a	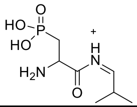	C7H16N2O4P$^+$	223.0842	223.0840	-0.85	223.0843	0.57	223.0844	0.59
b	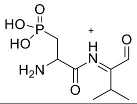	C8H16N2O5P$^+$	251.0791	251.0787	-1.87	251.0792	0.11	251.0790	-0.53
w	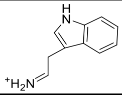	C10H11N2$^+$	159.0917	159.0916	-0.17				
x	*y* – NH3	C11H10NO2$^+$	188.0706	188.0705	-0.40				
y	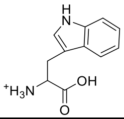	C11H13N2O2$^+$	205.0972	205.0971	-0.41				
z	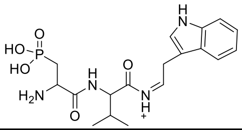	C18H26N4O5P$^+$	409.1635	409.1624	-2.79				
aa	*ab* – H2O	C19H26N4O6P$^+$	437.1585	437.1577	-1.76				
ab	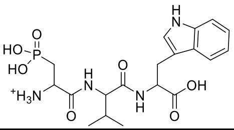	C19H28N4O7P$^+$	455.1690	455.1677	-2.81				
ac	*ad* – H2O	C11H21N3O5P$^+$	306.1213			306.1214	0.12		
ad	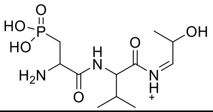	C11H23N3O6P$^+$	324.1319			324.1320	0.42		
ae	*af* – H2O	C12H21N3O6P$^+$	334.1163			334.1161	-0.52		
af	*ag* – H2O	C12H23N3O7P$^+$	352.1268			352.1267	-0.20		
ag	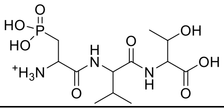	C12H25N3O8P$^+$	370.1374			370.1373	-0.34		
ah	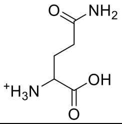	C5H11N2O3$^+$	147.0764					147.0764	0.12
ai	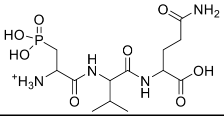	C13H26N4O8P$^+$	397.1483					397.1480	-0.61

Table S61. PnAla-Val-Pro tripeptide fragments

Fragment				PnAla-Val-Pro	
ID	Structure	Formula	Calc m/z	Obs m/z	Δppm
a	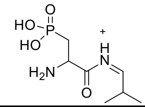	$C7H16N2O4P^{+}$	223.0842	223.0840	0.44
b	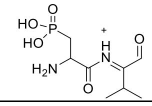	$C8H16N2O5P^{+}$	251.0791	251.0787	-0.56
aj	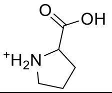	$C5H10NO2^{+}$	116.0706	116.0705	-1.23
ak	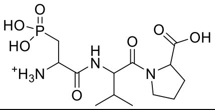	$C13H25N3O7P^{+}$	366.1425	366.1416	-2.44

Table S62. PnAla-Ile-Ile, PnAla-Ile-Phe, and PnAla-Ile-aThr tripeptide fragments

Fragment				PnAla-Ile-Ile		PnAla-Ile-Phe		PnAla-Ile-aThr	
ID	Structure	Formula	Calc m/z	Obs m/z	Δppm	Obs m/z	Δppm	Obs m/z	Δppm
a	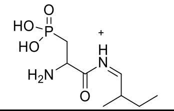	$C8H18N2O4P^{+}$	237.0999	237.0999	-0.04	237.1000	0.65	237.0998	-0.32
b	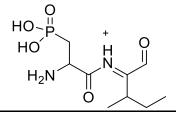	$C9H18N2O5P^{+}$	265.0948	265.0946	-0.51	265.0947	-0.29	265.0948	-0.05
c	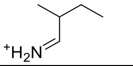	$C5H12N^{+}$	86.0964	86.0965	0.37				
d	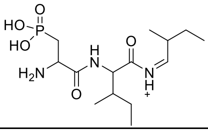	$C14H29N3O5P^{+}$	350.1839	350.1837	-0.69				
e	*f* – H2O	$C15H29N3O6P^{+}$	378.1789	378.1789	0.05				
f	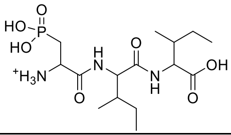	$C15H31N3O7P^{+}$	396.1894	396.1893	-0.25				
g	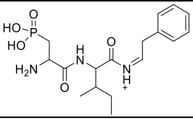	$C17H27N3O5P^{+}$	384.1683			384.1674	-2.17		
h	*i* – H2O	$C18H27N3O6P^{+}$	412.1632			412.1631	-0.20		
i	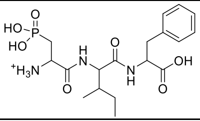	$C18H29N3O7P^{+}$	430.1738			430.1735	-0.50		
j	*k* – H2O	$C12H23N3O5P^{+}$	320.1370					320.1371	0.48
k	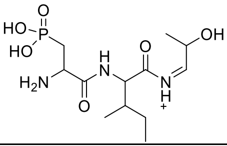	$C12H25N3O6P^{+}$	338.1478					338.1474	-0.29
l	*m* – H2O	$C13H23N3O6P^{+}$	348.1319					348.1318	-0.33
m	*n* – H2O	$C13H25N3O7P^{+}$	366.1425					366.1426	0.35
n	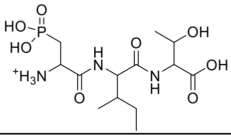	$C13H27N3O8P^{+}$	384.1530					384.1527	-0.86

Table S63. PnAla-Leu-Leu tripeptide fragments

Fragment				PnAla-Leu-Leu	
ID	Structure	Formula	Calc m/z	Obs m/z	Δppm
a	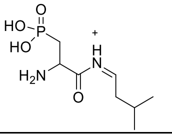	C8H18N2O4P⁺	237.0999	237.1000	0.54
b	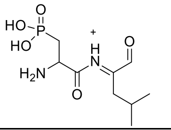	C9H18N2O5P⁺	265.0947	265.0947	-0.19
c	*d* – H2O	C15H29N3O6P⁺	378.1789	378.1783	-1.56
d	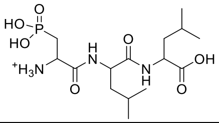	C15H31N3O7P⁺	396.1894	396.1893	-0.39

Table S64. PnAla-Met-Val, PnAla-Met-Leu, and PnAla-Met-Met tripeptide fragments

Fragment				PnAla-Met-Val		PnAla-Met-Leu		PnAla-Met-Met	
ID	Structure	Formula	Calc m/z	Obs m/z	Δppm	Obs m/z	Δppm	Obs m/z	Δppm
a	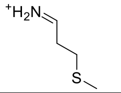	C4H10NS$^+$	104.0529					104.0527	-0.99
b	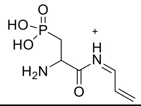	C6H12N2O4P$^+$	207.0529	207.0531	0.92	207.0531	0.63	207.0528	-0.37
c	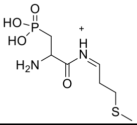	C7H16N2O4PS$^+$	255.0563	255.0562	-0.55	255.0564	0.33	255.0558	-1.81
d	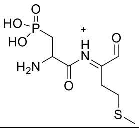	C8H16N2O5PS$^+$	283.0512	283.0511	-0.23	283.0513	2.81	283.0508	-1.53
e	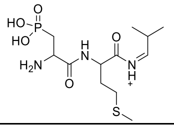	C12H25N3O5PS$^+$	354.1247	354.1243	-1.18				
f	*h* – H2O	C13H25N3O6PS$^+$	382.1196	382.1196	0.05				
g	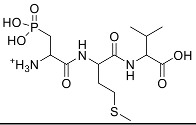	C13H27N3O7PS$^+$	400.1302	400.1301	-0.22				
h	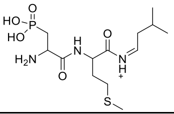	C13H27N3O5PS$^+$	368.1404			368.1403	-0.23		
i	*j* – H2O	C14H27N3O6PS$^+$	396.1353			396.1352	-0.18		
j	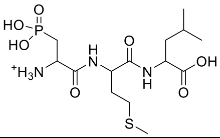	C14H29N3O7PS$^+$	414.1458			414.1454	-0.99		
k	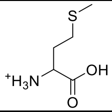	C5H12NO2S$^+$	150.0583					150.0583	-0.30
l	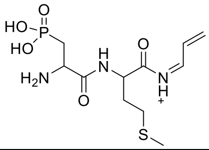	C11H21N3O5PS$^+$	338.0934					338.0930	-1.12
m	*n* – H2O	C13H25N3O6PS2$^+$	414.0917					414.0910	-1.71
n	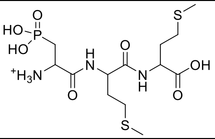	C13H27N3O7PS2$^+$	432.1023					432.1018	-1.08

Table S65. PnAla-Met-Trp tripeptide fragments

Fragment				PnAla-Met-Trp	
ID	Structure	Formula	Calc m/z	Obs m/z	Δppm
a	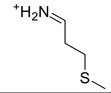	$C4H10NS^+$	104.0529	104.0527	-1.71
b	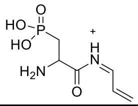	$C6H12N2O4P^+$	207.0529	207.0526	-1.44
c	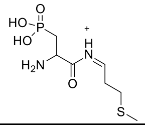	$C7H16N2O4PS^+$	255.0563	255.0557	-2.43
d	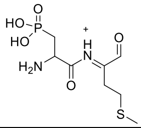	$C8H16N2O5PS^+$	283.0512	283.0508	-1.38
o	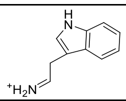	$C10H11N2^+$	159.0917	159.0916	-0.64
p	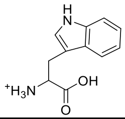	$C11H13N2O2^+$	205.0972	205.0969	-1.18
q	*r* – H2O	$C19H26N4O6PS^+$	469.1305	469.1298	-1.45
r	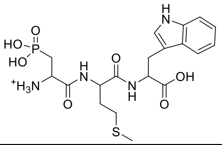	$C19H28N4O7PS^+$	487.1411	487.1403	-1.55

Table S66. PnAla-Phe-Val, PnAla-Phe-Ile, and PnAla-Phe-Leu tripeptide fragments

Fragment				PnAla-Phe-Val		PnAla-Phe-Ile		PnAla-Phe-Leu	
ID	Structure	Formula	Calc m/z	Obs m/z	Δppm	Obs m/z	Δppm	Obs m/z	Δppm
a	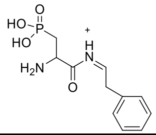	$C_{11}H_{16}N_2O_4P^+$	271.0842	271.0842	-0.01	271.0843	0.20	271.0841	-0.49
b	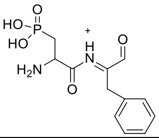	$C_{12}H_{16}N_2O_5P^+$	299.0791	299.0791	-0.13	299.0792	0.19	299.0791	-0.24
c	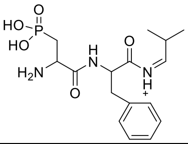	$C_{16}H_{25}N_3O_5P^+$	370.1526	370.1526	-0.10				
d	*e* – H2O	$C_{17}H_{25}N_3O_6P^+$	398.1476	398.1473	-0.65				
e	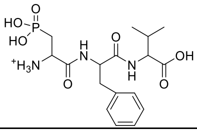	$C_{17}H_{27}N_3O_7P^+$	416.1581	416.1578	-0.84				
f	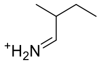	$C_5H_{12}N^+$	86.0964			86.0964	0.21		
g	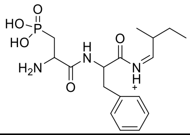	$C_{17}H_{27}N_3O_5P^+$	384.1683			384.1674	-2.17		
h	*i* – H2O	$C_{18}H_{27}N_3O_6P^+$	412.1632			412.1631	-0.20		
i	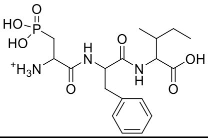	$C_{18}H_{29}N_3O_7P^+$	430.1738			430.1735	-0.50		
j	*k* – H2O	$C_{18}H_{27}N_3O_6P^+$	412.1632					412.1626	-1.51
k	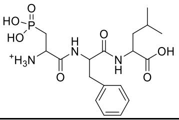	$C_{18}H_{29}N_3O_7P^+$	430.1738					430.1735	-0.68

Table S67. PnAla-Phe-Met, PnAla-Phe-Phe, and PnAla-Phe-Tyr tripeptide fragments

Fragment				PnAla-Phe-Met		PnAla-Phe-Phe		PnAla-Phe-Tyr	
ID	Structure	Formula	Calc m/z	Obs m/z	Δppm	Obs m/z	Δppm	Obs m/z	Δppm
a	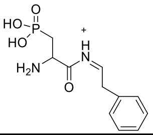	$C11H16N2O4P^+$	271.0842	271.0841	-0.38	271.0837	-1.97	271.0842	-0.17
b	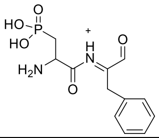	$C12H16N2O5P^+$	299.0791	299.0791	-0.13	299.0789	-0.87	299.0790	-0.38
l	*m* – H2O	$C17H25N3O6PS^+$	430.1196	430.1190	-1.38				
m	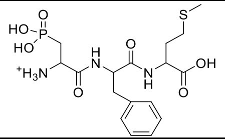	$C17H27N3O7PS^+$	448.1302	448.1290	-2.72				
n	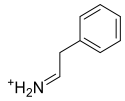	$C8H10N^+$	120.0808			120.0807	-0.24		
o	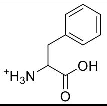	$C9H12NO2^+$	166.0863			166.0862	-0.47		
p	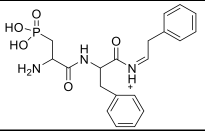	$C20H25N3O5P^+$	418.1526			418.1515	-2.62		
q	*r* – H2O	$C21H25N3O6P^+$	446.1476			446.1466	-2.16		
r	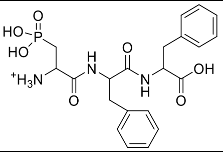	$C21H27N3O7P^+$	464.1581			464.1576	-1.10		
s	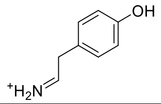	$C8H10NO^+$	136.0757					136.0759	1.45
t	*u* – H2O	$C21H25N3O7P^+$	462.1425					462.1423	-0.34
u	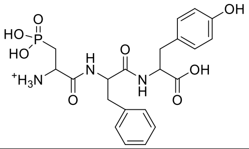	$C21H27N3O8P^+$	480.1530					480.1534	0.67

Table S68. PnAla-Phe-Trp, PnAla-Phe-Thr, and PnAla-Phe-aThr tripeptide fragments

Fragment				PnAla-Phe-Trp		PnAla-Phe-Thr		PnAla-Phe-aThr	
ID	Structure	Formula	Calc m/z	Obs m/z	Δppm	Obs m/z	Δppm	Obs m/z	Δppm
a	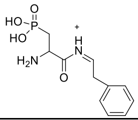	$C11H16N2O4P^+$	271.0842	271.0841	-0.26	271.0842	-0.25	271.0841	-0.46
b	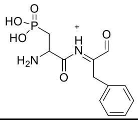	$C12H16N2O5P^+$	299.0791	299.0792	0.21	299.0791	-0.21	299.0793	0.58
v	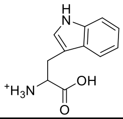	$C11H13N2O2^+$	205.0972	205.0972	0.42				
w	*x* – H2O	$C23H26N4O6P^+$	485.1585	485.1584	-0.07				
x	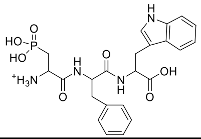	$C23H28N4O7P^+$	503.1690	503.1683	-1.34				
y	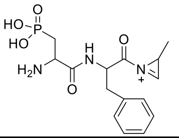	$C15H21N3O5P^+$	354.1213			354.1214	0.19		
z	*z* – H2O	$C16H21N3O6P^+$	382.1163			382.1160	-0.57		
aa	*ab* – H2O	$C16H23N3O7P^+$	400.1268			400.1267	-0.18	400.1264	-0.93
ab	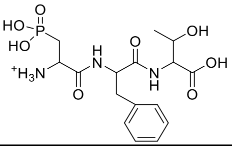	$C16H25N3O8P^+$	418.1374			418.1371	-0.60	418.1368	-1.46

Table S69. PnAla-Tyr-Val, PnAla-Tyr-Leu, and PnAla-Tyr-Met tripeptide fragments

Fragment				PnAla-Tyr-Val		PnAla-Tyr-Leu		PnAla-Tyr-Met	
ID	Structure	Formula	Calc m/z	Obs m/z	Δppm	Obs m/z	Δppm	Obs m/z	Δppm
a	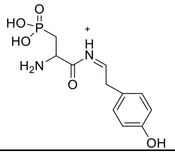	C11H16N2O5P$^+$	287.0791	287.0788	-1.32	287.0791	-0.05	287.0789	0.33
b	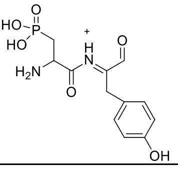	C12H16N2O6P$^+$	315.0741	315.0737	-1.00	315.0738	0.21	315.0735	-0.81
c	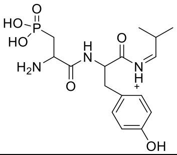	C16H25N3O6P$^+$	386.1476	386.1469	-1.56				
d	*e* – H2O	C17H25N3O7P$^+$	414.1425	414.1413	-2.77				
e	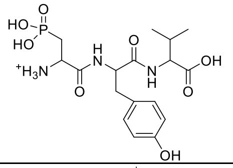	C17H27N3O8P$^+$	432.1530	432.1519	-2.72				
f	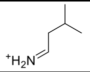	C5H12N$^+$	86.0964			86.0964	-0.33		
g		C17H26N3O6P$^+$	400.1632			400.1632	-0.03		
h	*i* – H2O	C18H27N3O7P$^+$	428.1581			428.1577	-0.86		
i	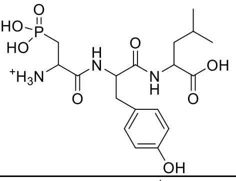	C18H29N3O8P$^+$	446.1687			446.1684	-0.57		
j	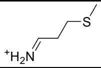	C4H10NS$^+$	104.0529					104.0525	-3.03
k	*l* – H2O	C17H25N3O7PS$^+$	446.1145					446.1132	-2.93
l	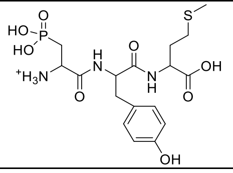	C17H27N3O8PS$^+$	464.1257					464.1230	-4.55

Table S70. PnAla-Tyr-Phe, PnAla-Tyr-Tyr, and PnAla-Tyr-Trp tripeptide fragments

Fragment				PnAla-Tyr-Phe		PnAla-Tyr-Tyr		PnAla-Tyr-Trp	
ID	Structure	Formula	Calc m/z	Obs m/z	Δppm	Obs m/z	Δppm	Obs m/z	Δppm
a	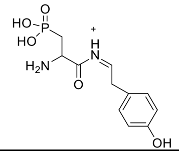	$C_{11}H_{16}N_2O_5P^+$	287.0791	287.0790	-0.30	287.0790	-0.60	287.0790	-0.58
b	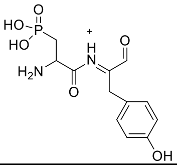	$C_{12}H_{16}N_2O_6P^+$	315.0741	315.0742	0.43	315.0740	-0.22	315.0737	-1.23
m	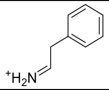	$C_8H_{10}N^+$	120.0808	120.0809	1.01				
n	*o* – H2O	$C_{21}H_{25}N_3O_7P^+$	462.1425	462.1423	-0.34				
o	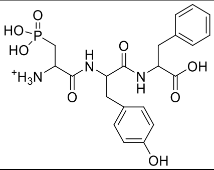	$C_{21}H_{27}N_3O_8P^+$	480.1530	480.1534	0.67				
p	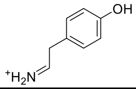	$C_8H_{10}NO^+$	136.0757			136.0759	1.22		
q	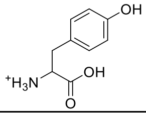	$C_9H_{12}NO_3^+$	182.0812			182.0814	1.16		
r	*s* – H2O	$C_{21}H_{25}N_3O_8P^+$	478.1374			478.1376	0.55		
s	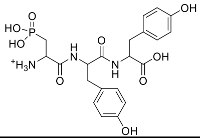	$C_{21}H_{27}N_3O_9P^+$	496.1479			496.1475	-0.94		
t	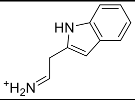	$C_{10}H_{11}N_2^+$	159.0917					159.0917	0.28
u	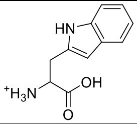	$C_{11}H_{13}N_2O_2^+$	205.0972					205.0974	0.97
v	*w* – H2O	$C_{23}H_{26}N_4O_7P^+$	501.1534					501.1519	-2.82
w	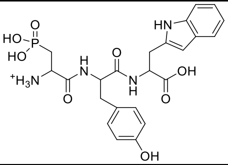	$C_{23}H_{28}N_4O_8P^+$	519.1639					519.1641	0.39

Table S71. PnAla-Tyr-Gly tripeptide fragments

Fragment				PnAla-Tyr-Gly	
ID	Structure	Formula	Calc m/z	Obs m/z	Δppm
a	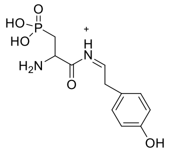	C11H16N2O5P⁺	287.0791	287.0788	-1.17
b	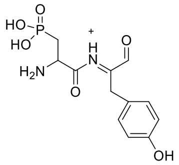	C12H16N2O6P⁺	315.0741	315.0734	-2.11
x	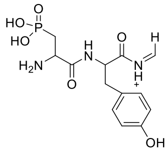	C13H19N3O6P⁺	344.1006	344.1000	-1.87
y	*z* – H2O	C14H19N3O7P⁺	372.0955	372.0950	-1.29
z	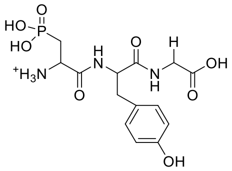	C14H21N3O8P⁺	390.1061	390.1056	-1.35

Table S72. PnAla-Trp-Val, PnAla-Trp-Ile, and PnAla-Trp-Leu tripeptide fragments

Fragment				PnAla-Trp-Val		PnAla-Trp-Ile		PnAla-Trp-Leu	
ID	Structure	Formula	Calc m/z	Obs m/z	Δppm	Obs m/z	Δppm	Obs m/z	Δppm
a	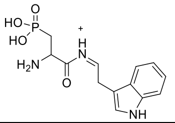	$C13H17N3O4P^+$	310.0951	310.0946	-1.79	310.0951	-0.40	310.0949	0.42
b	*c* – H2O	$C14H15N3O4P^+$	320.0795	320.0791	-1.19	320.0798	1.04	320.0794	-0.23
c	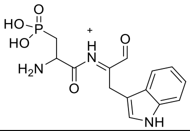	$C14H17N3O5P^+$	338.0900	338.0895	-1.52	338.0898	-0.57	338.0900	-0.09
d	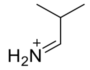	$C4H10N^+$	72.0808	72.0808	0.17				
e	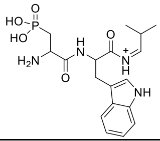	$C18H26N4O5P^+$	409.1635	409.1624	-2.79				
f	*g* – H2O	$C19H26N4O6P^+$	437.1585	437.1577	-1.76				
g	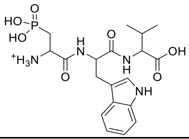	$C19H28N4O7P^+$	455.1690	455.1677	-2.81				
h	*i* – H2O	$C20H28N4O6P^+$	451.1741			451.1737	-0.89		
i	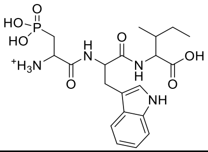	$C20H30N4O7P^+$	469.1847			469.1870	4.88		
j	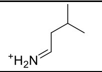	$C5H12N^+$	86.0964					86.0964	-0.08
k	*l* – H2O	$C20H28N4O6P^+$	451.1741					451.1737	-0.93
l	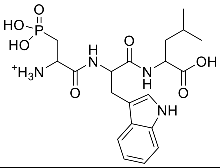	$C20H30N4O7P^+$	469.1847					469.1869	4.84

Table S73. PnAla-Trp-Met, PnAla-Trp-Phe, and PnAla-Trp-Tyr tripeptide fragments

Fragment				PnAla-Trp-Met		PnAla-Trp-Phe		PnAla-Trp-Tyr	
ID	Structure	Formula	Calc m/z	Obs m/z	Δppm	Obs m/z	Δppm	Obs m/z	Δppm
a	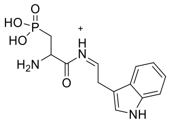	$C_{13}H_{17}N_3O_4P^+$	310.0951	310.0943	-2.59	310.0951	-0.10	310.0951	-0.07
b	*c* – H2O	$C_{14}H_{15}N_3O_4P^+$	320.0795	320.0789	-0.94	320.0795	-0.03	320.0787	-2.39
c	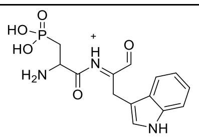	$C_{14}H_{17}N_3O_5P^+$	338.0900	338.0892	-2.49	338.0899	-0.31	338.0897	-1.06
m	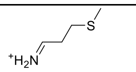	$C_4H_{10}NS^+$	104.0529	104.0527	-1.71				
n	*o* – H2O	$C_{19}H_{26}N_4O_6PS^+$	469.1305	469.1298	-1.45				
o	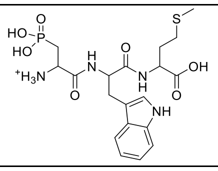	$C_{19}H_{28}N_4O_7PS^+$	487.1411	487.1403	-1.55				
p	*q* – H2O	$C_{23}H_{26}N_4O_6P^+$	485.1585			485.1584	-0.07		
q	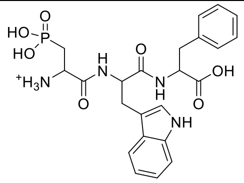	$C_{23}H_{28}N_4O_7P^+$	503.1690			503.1683	-1.34		
r	*s* – H2O	$C_{23}H_{26}N_4O_7P^+$	501.1534					501.1519	-2.82
s	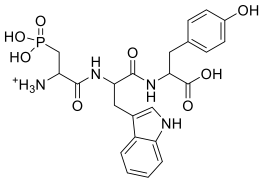	$C_{23}H_{28}N_4O_8P^+$	519.1639					519.1641	0.39

Table S74. PnAla-Trp-Trp, PnAla-Trp-Thr, and PnAla-Trp-Lys tripeptide fragments

Fragment				PnAla-Trp-Trp		PnAla-Trp-Thr		PnAla-Trp-Lys	
ID	Structure	Formula	Calc m/z	Obs m/z	Δppm	Obs m/z	Δppm	Obs m/z	Δppm
a	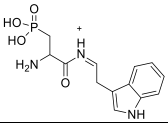	$C_{13}H_{17}N_3O_4P^+$	310.0951	310.0949	-0.60	310.0948	-0.98	310.0945	-1.85
b	*c* – H_2O	$C_{14}H_{15}N_3O_4P^+$	320.0795	320.0800	1.74				
c	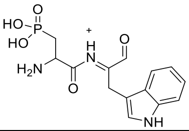	$C_{14}H_{17}N_3O_5P^+$	338.0900	338.0899	-0.53	338.0896	-1.36		
t	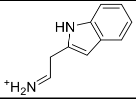	$C_{10}H_{11}N_2^+$	159.0917	159.0917	0.37				
u	*v* – H_2O	$C_{25}H_{27}N_5O_6P^+$	524.1694	524.1692	-0.25				
v	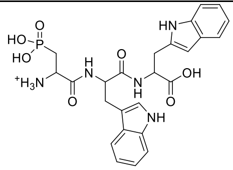	$C_{25}H_{29}N_5O_7P^+$	542.1799	542.1799	0.06				
w	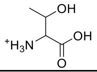	$C_4H_{10}NO_3^+$	120.0655			120.0655	0.03		
x	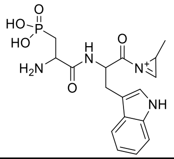	$C_{17}H_{22}N_4O_5P^+$	393.1322			393.1310	-3.21		
y	*z* – H_2O	$C_{18}H_{24}N_4O_7P^+$	439.1377			439.1373	-0.95		
z	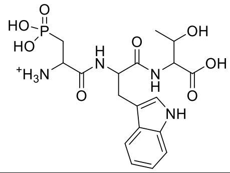	$C_{18}H_{26}N_4O_8P^+$	457.1483			457.1478	-1.01		
aa	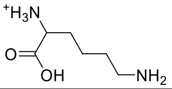	$C_6H_{15}N_2O_2^+$	147.1128					147.1130	1.02
ab	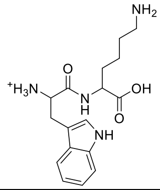	$C_{17}H_{25}N_4O_3^+$	333.1921					333.1917	-1.21
ac	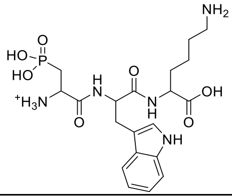	$C_{20}H_{31}N_5O_7P^+$	484.1956					484.1943	-2.63

Table S75. PnAla-Trp-Gly tripeptide fragments

Fragment				PnAla-Trp-Gly	
ID	Structure	Formula	Calc m/z	Obs m/z	Δppm
a	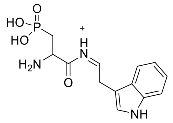	C13H17N3O4P$^+$	310.0951	310.0946	-1.52
c	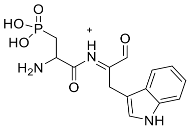	C14H17N3O5P$^+$	338.0900	338.0893	-2.09
ad	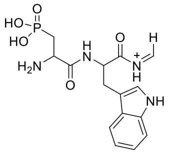	C15H20N4O5P$^+$	367.1166	367.1157	-2.35
ae	*af* – H2O	C16H20N4O6P$^+$	395.1115	395.1114	-0.26
af	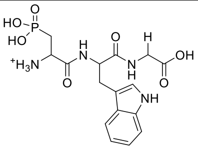	C16H22N4O7P$^+$	413.1221	413.1207	-3.23

Table S76. PnAla-Ser-Val and PnAla-Ser-Ile tripeptide fragments

Fragment				PnAla-Ser-Val		PnAla-Ser-Ile	
ID	Structure	Formula	Calc m/z	Obs m/z	Δppm	Obs m/z	Δppm
a	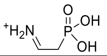	$C_2H_7NO_3P^+$	124.0158	124.0157	-0.76	124.0157	-0.67
b	*c* – H_2O	$C_5H_{10}N_2O_4P^+$	193.0373	193.0372	-0.41	193.0372	-0.6
c	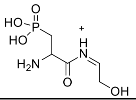	$C_5H_{12}N_2O_5P^+$	211.0478	211.0476	-1.05	211.0476	-1.02
d	*e* – H_2O	$C_6H_{10}N_2O_5P^+$	221.0322	221.0319	-1.09	221.0322	0.05
e	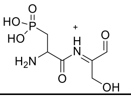	$C_6H_{12}N_2O_6P^+$	239.0428	239.0424	-1.46	239.0424	-1.65
f	*e* + H_2O	$C_6H_{14}N_2O_7P^+$	257.0533	257.0529	-1.7	257.0531	-0.71
g	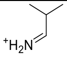	$C_4H_{10}N^+$	72.0808	72.0808	0.07		
h	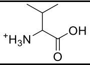	$C_5H_{12}NO_2^+$	118.0863	118.0861	-1.4		
i	*j* – H_2O	$C_8H_{15}N_2O_3^+$	187.1077	187.1076	-0.61		
j	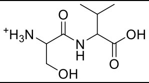	$C_8H_{17}N_2O_4^+$	205.1183	205.1181	-0.92		
k	*i* + CO	$C_9H_{15}N_2O_4^+$	215.1026	215.1025	-0.53		
l	*m* – H_2O	$C_{10}H_{19}N_3O_5P^+$	292.1057	292.1051	-1.85		
m	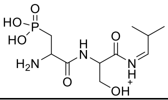	$C_{10}H_{21}N_3O_6P$	310.1163	310.1158	-1.52		
n	*o* – H_2O	$C_{11}H_{19}N_3O_6P^+$	320.1006	320.1002	-1.22		
o	*p* – H_2O	$C_{11}H_{21}N_3O_7P^+$	338.1112	338.1104	-2.16		
p	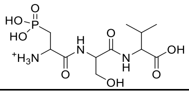	$C_{11}H_{23}N_3O_8P^+$	356.1217	356.1212	-1.56		
q	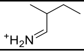	$C_5H_{12}N^+$	86.0964			86.0963	-1.16
r	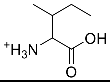	$C_6H_{14}NO_2^+$	132.1019			132.1018	-0.9
s	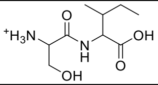	$C_9H_{19}N_2O_4^+$	219.1339			219.1338	-0.68
t	*s* + CO – H_2O	$C_{10}H_{17}N_2O_4^+$	229.1186			229.1181	-0.45
u	*v* – H_2O	$C_{11}H_{19}N_3O_5P^+$	304.1057			304.1057	0.15
v	*x* – H_2O	$C_{11}H_{21}N_3O_5P^+$	306.1213			306.1208	-1.90
w	*y* – H_2O	$C_{12}H_{19}N_3O_5P^+$	316.1057			316.1059	0.77
x	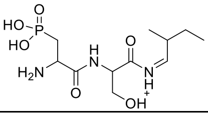	$C_{11}H_{23}N_3O_6P^+$	324.1319			324.1314	-1.56
y	*z* – H_2O	$C_{12}H_{21}N_3O_6P^+$	334.1163			334.1157	-1.57
z	*aa* – H_2O	$C_{12}H_{23}N_3O_7P^+$	352.1268			352.1263	-1.46
aa	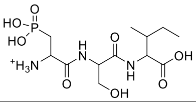	$C_{12}H_{25}N_3O_8P^+$	370.1374			370.1368	-1.43

Table S77. PnAla-Ser-Leu and PnAla-Ser-Met tripeptide fragments

Fragment				PnAla-Ser-Leu		PnAla-Ser-Met	
ID	Structure	Formula	Calc m/z	Obs m/z	Δppm	Obs m/z	Δppm
a	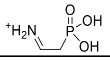	C2H7NO3P⁺	124.0158	124.0157	-1.49	124.0158	0.06
b	*c* – H2O	C5H10N2O4P⁺	193.0373	193.0373	0.07	193.0373	0.27
c	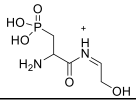	C5H12N2O5P⁺	211.0478	211.0476	-1.14	211.0476	-1.15
d	*e* – H2O	C6H10N2O5P⁺	221.0322	221.0322	0.05		
e	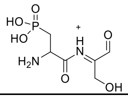	C6H12N2O6P⁺	239.0428	239.0423	-1.89	239.0424	-1.40
f	*e* + H2O	C6H14N2O7P⁺	257.0533	257.0533	-0.15	257.0532	-0.61
ab	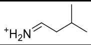	C5H12N⁺	86.0964	86.0963	-0.13		
ac	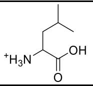	C6H14NO2⁺	132.1019	132.1018	-0.7		
ad	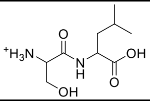	C9H19N2O4⁺	219.1339	219.1337	-1.01		
ae	*ad* + CO – H2O	C10H17N2O4⁺	229.1186	229.1183	0.18		
af	*ag* – H2	C11H19N3O5P⁺	304.1057	304.1058	0.35		
ag	*ai* – H2O	C11H21N3O5P⁺	306.1213	306.1209	-1.25		
ah	*aj* – H2O	C12H19N3O5P⁺	316.1057	316.1060	0.89		
ai	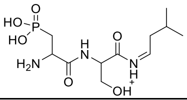	C11H23N3O6P⁺	324.1319	324.1314	-1.42		
aj	*ak* – H2O	C12H21N3O6P⁺	334.1163	334.1157	-1.68		
ak	*al* – H2O	C12H23N3O7P⁺	352.1268	352.1262	-1.79		
al	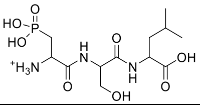	C12H25N3O8P⁺	370.1374	370.1367	-1.74		
am	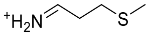	C4H10NS⁺	104.0529			104.0527	-1.16
an	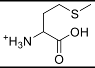	C5H12NO2S⁺	150.0583			150.0583	0.00
ao	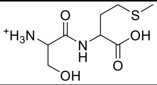	C8H17N2O4S⁺	237.0904			237.0901	-1.21
ap	*ao* + CO – H2O	C9H15N2O4S⁺	247.0747			247.0745	-0.73
aq	*ar* – H2O	C9H15N3O5P⁺	276.0744			276.0741	-1.07
ar	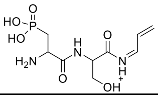	C9H17N3O6P⁺	294.0850			294.0842	-2.39
as	*at* – H2	C10H17N3O5PS⁺	322.0621			322.0621	0.09
at	*au* – H2O	C10H19N3O5PS⁺	324.0778			324.0775	-0.64
au	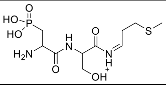	C10H21N3O6PS⁺	342.0883			342.0884	0.27
av	*aw* – H2O	C11H19N3O6PS⁺	352.0727			352.0727	0.15
aw	*ax* – H2O	C11H21N3O7PS⁺	370.0832			370.0826	-0.87
ax	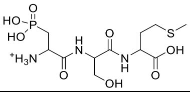	C11H23N3O8PS⁺	388.0938			388.0935	-0.85

Table S78. PnAla-Ser-Phe and PnAla-Ser-Tyr tripeptide fragments

Fragment				PnAla-Ser-Phe		PnAla-Ser-Tyr	
ID	Structure	Formula	Calc m/z	Obs m/z	Δppm	Obs m/z	Δppm
a	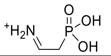	C2H7NO3P^{+}	124.0158			124.0160	1.59
b	*c* – H2O	C5H10N2O4P^{+}	193.0373			193.0376	1.73
c	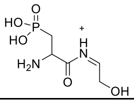	C5H12N2O5P^{+}	211.0478	211.0479		211.0479	0.27
d	*e* – H2O	C6H10N2O5P^{+}	221.0322				
e	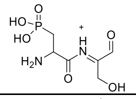	C6H12N2O6P^{+}	239.0428	239.0427	-0.41	239.0427	-0.20
ay	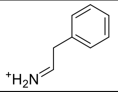	C8H10N^{+}	120.0808	120.0809	0.54		
az	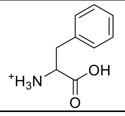	C9H12NO2^{+}	166.0863	166.0865	1.24		
ba	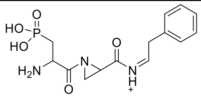	C14H19N3O5P^{+}	340.1057	340.1055	-0.48		
bb	*bc* – H2O	C15H21N3O7P^{+}	386.1109	386.1110	-0.70		
bc	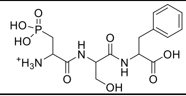	C15H23N3O8P^{+}	404.1217	404.1216	-0.31		
bd	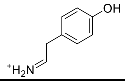	C8H10NO^{+}	136.0757			136.0758	0.75
be	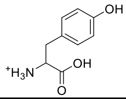	C9H12NO3^{+}	182.0812			182.0813	0.82
bf	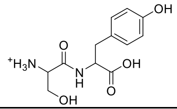	C12H17N2O5^{+}	269.1132			269.1132	0.19
bg	*bf* + CO – H2O	C13H15N2O5^{+}	279.0976			279.0977	0.44
bh	*bi* – H2O	C14H19N3O6P^{+}	356.1006			356.1006	0.07
bi	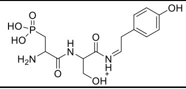	C14H21N3O7P^{+}	374.1112			374.1113	0.36
bj	*bk* – H2O	C15H19N3O7P^{+}	384.0955			384.0954	-0.28
bk	*bl* – H2O	C15H21N3O8P^{+}	402.1061			402.1062	0.38
bl	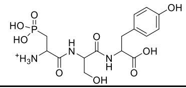	C15H23N3O9P^{+}	420.1166			420.1164	-0.5

Table S79. PnAla-Ser-Ser, PnAla-Ser-Thr, and PnAla-Ser-aThr tripeptide fragments

Fragment				PnAla-Ser-Ser		PnAla-Ser-Thr		PnAla-Ser-aThr	
ID	Structure	Formula	Calc m/z	Obs m/z	Δppm	Obs m/z	Δppm	Obs m/z	Δppm
a	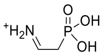	C2H7NO3P$^+$	124.0158	124.0155	-0.27	124.0159	1.00	124.0159	0.80
b	*c* – H2O	C5H10N2O4P$^+$	193.0373	193.0369	-0.37	193.0375	1.09	193.0375	1.24
c	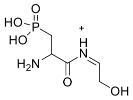	C5H12N2O5P$^+$	211.0478	211.0473	-0.55	211.0478	-0.16	211.0479	0.52
d	*e* – H2O	C6H10N2O5P$^+$	221.0322	221.0316	-0.57	221.0322	0.20	221.0324	0.79
e	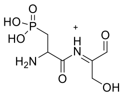	C6H12N2O6P$^+$	239.0428	239.0420	-0.77	239.0426	-0.42	239.0427	-0.14
bm	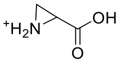	C3H6NO2$^+$	88.0393	88.0391	-0.20				
bn	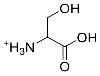	C3H8NO3$^+$	106.0499	106.0496	-0.25				
bo	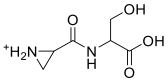	C6H11N2O4$^+$	175.0713	175.0710	-2.03				
bp	*bo* + CO	C7H11N2O5$^+$	203.0663	203.0658	-2.33				
bq	*br* – CO	C8H15N3O6P$^+$	280.0693	280.0686	-2.62				
br	*bs* – H2O	C9H15N3O7P$^+$	308.0642	308.0663	-0.92				
bs	*bt* – H2O	C9H17N3O8P$^+$	326.0748	326.0738	-0.96				
bt	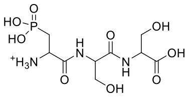	C9H19N3O9P$^+$	344.0853	344.0844	-0.93				
bu	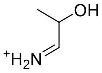	C3H8NO$^+$	74.0600			74.0600	0.01	74.0601	0.98
bv	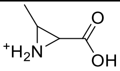	C4H8NO2$^+$	102.0550			102.0550	0.05	102.0550	0.56
bw	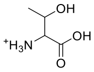	C4H10NO3$^+$	120.0655			120.0656	0.48	120.0656	0.93
bx	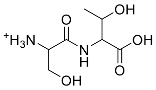	C7H15N2O5$^+$	207.0976			207.0976	0.09	207.0977	0.73
by	*bx* + CO – H2O	C8H13N2O5$^+$	217.0819			217.0819	0.11	217.0821	0.94
bz	*ca* – H2O	C9H17N3O6P$^+$	294.0850			294.0848	-0.44	294.0850	0.05
ca	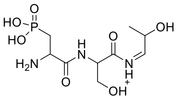	C9H19N3O7P$^+$	312.0955					312.0956	0.21
cb	*cc* – H2O	C10H17N3O7P$^+$	322.0799			322.0797	-0.51	322.0798	-0.20
cc	*cd* – H2O	C10H19N3O8P$^+$	340.0904			340.0903	-0.50	340.0904	-0.15
cd	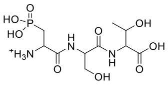	C10H21N3O9P$^+$	358.1010			358.1009	-0.16	358.1011	0.20

Table S80. PnAla-Thr-Val, PnAla-Thr-Ile, and PnAla-Thr-Leu tripeptide fragments

Fragment				PnAla-Thr-Val		PnAla-Thr-Ile		PnAla-Thr-Leu	
ID	Structure	Formula	Calc m/z	Obs m/z	Δppm	Obs m/z	Δppm	Obs m/z	Δppm
a	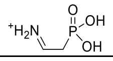	C2H7NO3P$^+$	124.0158	124.0155	-2.28				
b	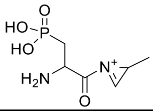	C6H12N2O4P$^+$	207.0529	207.0526	-1.75			207.0531	0.93
c	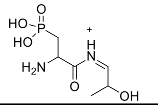	C6H14N2O5P$^+$	225.0635	225.0630	-2.33	225.0635	0.16	225.0635	0.01
d	*f* – H2O	C7H12N2O5P$^+$	235.0478	235.0473	-2.11			235.0478	-0.11
e	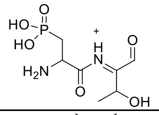	C7H14N2O6P$^+$	253.0584	253.0578	-2.48	253.0583	-0.22	253.0583	-0.51
f	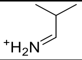	C4H10N$^+$	72.0808	72.0807	-0.99				
g	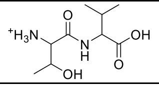	C9H19N2O4$^+$	219.1339	219.1335	-2.05				
h	*e* + H2O	C7H16N2O7P$^+$	271.0690	271.0683	-2.32				
i	*k* – H2O	C11H21N3O5P$^+$	306.1213	306.1206	-2.26				
j	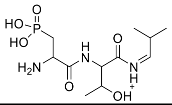	C11H23N3O6P$^+$	324.1319	324.1311	-2.36				
k	*m* – H2O	C12H21N3O6P$^+$	334.1163	334.1154	-2.43				
l	*n* – H2O	C12H23N3O7P$^+$	352.1268	352.1260	-2.37				
m	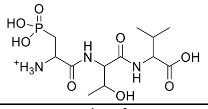	C12H25N3O8P$^+$	370.1374	370.1366	-2.05				
n	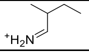	C5H12N$^+$	86.0964			86.0964	-0.14		
o	*p* – H2O	C12H23N3O5P$^+$	320.1370			320.1370	0.18		
p	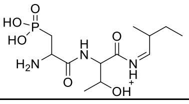	C12H25N3O6P$^+$	338.1478			338.1474	-0.32		
q	*r* – H2O	C13H23N3O6P$^+$	348.1319			348.1318	-0.15		
r	*s* – H2O	C13H25N3O7P$^+$	366.1425			366.1425	0.22		
s	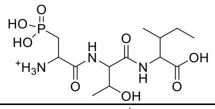	C13H27N3O8P$^+$	384.1530			384.1530	0.00		
t	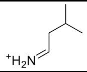	C5H12N$^+$	86.0964					86.0964	0.02
u	*v* – H2O	C12H23N3O5P$^+$	320.1370					320.1369	-0.23
v	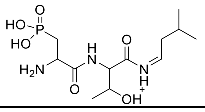	C12H25N3O6P$^+$	338.1478					338.1473	-0.64
w	*x* – H2O	C13H23N3O6P$^+$	348.1319					348.1320	0.29
x	*y* – H2O	C13H25N3O7P$^+$	366.1425					366.1425	0.18
y	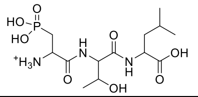	C13H27N3O8P$^+$	384.1530					384.1529	-0.22

Table S81. PnAla-Thr-Phe and PnAla-Thr-Trp tripeptide fragments

Fragment				PnAla-Thr-Phe		PnAla-Thr-Trp	
ID	Structure	Formula	Calc m/z	Obs m/z	Δppm	Obs m/z	Δppm
c	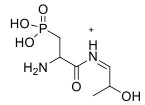	C6H14N2O5P⁺	225.0635	225.0636	0.36		
e	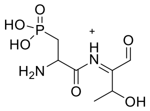	C7H14N2O6P⁺	253.0584	253.0584	0.06	253.0580	-1.46
z	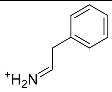	C8H10N⁺	120.0808	120.0809	-1.65		
aa	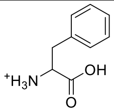	C9H12NO2⁺	166.0863	166.0865	1.38		
ab	*ad* – (H2O⁺CO)	C15H21N3O5P⁺	354.1213	354.1214	0.19		
ac	*ad* – H2O	C16H21N3O6P⁺	382.1163	382.1160	-0.57		
ad	*ae* – H2O	C16H23N3O7P⁺	400.1268	400.1267	-0.18		
ae	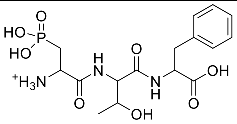	C16H25N3O8P⁺	418.1374	418.1371	-0.60		
af	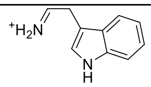	C10H11N2⁺	159.0917			159.0917	0.05
ag	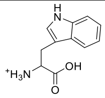	C11H13N2O2⁺	205.0972			205.0972	0.02
ah	*ai* – (H2O⁺CO)	C17H22N4O5P⁺	393.1322			393.1313	-2.48
ai	*aj* – H2O	C18H24N4O7P⁺	439.1377			439.1372	-1.07
aj	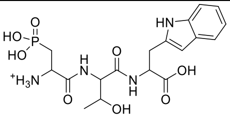	C18H26N4O8P⁺	457.1483			457.1478	-1.10

Table S82. PnAla-aThr-Val, PnAla-aThr-Ile, and PnAla-aThr-Leu tripeptide fragments

Fragment				PnAla-aThr-Val		PnAla-aThr-Ile		PnAla-aThr-Leu	
ID	Structure	Formula	Calc m/z	Obs m/z	Δppm	Obs m/z	Δppm	Obs m/z	Δppm
a	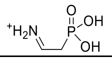	C2H7NO3P$^+$	124.0158	124.0160	1.33	124.0160	1.57		
b	*c* – H2O	C6H12N2O4P$^+$	207.0529	207.0529	-0.22	207.0531	0.92	207.0530	0.55
c	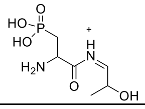	C6H14N2O5P$^+$	225.0635	225.0634	-0.43	225.0635	0.11	225.0636	0.67
d	*e* – H2O	C7H12N2O5P$^+$	235.0478	235.0478	0.00	235.0478	0.01		
e	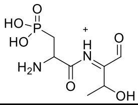	C7H14N2O6P$^+$	253.0584	253.0583	-0.41	253.0584	0.09	253.0582	-0.80
f	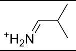	C4H10N$^+$	72.0808	72.0809	1.19				
g	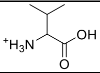	C5H12NO2$^+$	118.0863	118.0862	-0.19				
h	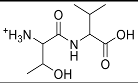	C9H19N2O4$^+$	219.1339	219.1340	0.34				
i	*h* + CO – H2O	C10H17N2O4$^+$	229.1183	229.1184	0.49				
j	*k* – H2O	C11H21N3O5P$^+$	306.1213	306.1211	-0.82				
k	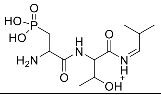	C11H23N3O6P$^+$	324.1319	324.1318	-0.25				
l	*m* – H2O	C12H21N3O6P$^+$	334.1163	334.1161	-0.38				
m	*n* – H2O	C12H23N3O7P$^+$	352.1268	352.1268	-0.14				
n	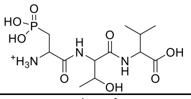	C12H25N3O8P$^+$	370.1374	370.1372	-0.42				
o	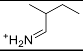	C5H12N$^+$	86.0964			86.0964	0.18		
p	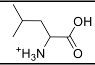	C6H14NO2$^+$	132.1019			132.1021	1.23		
q	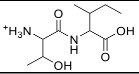	C10H21N2O4$^+$	233.1496			233.1498	0.80		
r	*s* – H2O	C12H23N3O5P$^+$	320.1370			320.1371	0.34		
s	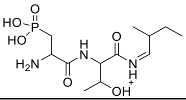	C12H25N3O6P$^+$	338.1478			338.1476	0.04		
t	*u* – H2O	C13H23N3O6P$^+$	348.1319			348.1317	-0.62		
u	*v* – H2O	C13H25N3O7P$^+$	366.1425			366.1425	0.17		
v	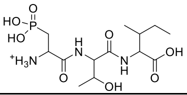	C13H27N3O8P$^+$	384.1530			384.1527	-0.91		
w	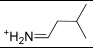	C5H12N$^+$	86.0964					86.0964	-0.10
x	*y* – H2O	C12H23N3O5P$^+$	320.1370					320.1370	-0.01
y	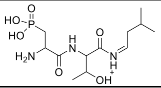	C12H25N3O6P$^+$	338.1478					338.1471	-1.37
z	*aa* – H2O	C13H23N3O6P$^+$	348.1319					348.1316	-0.85
aa	*ab* – H2O	C13H25N3O7P$^+$	366.1425					366.1424	-0.12
ab	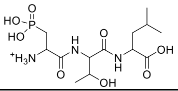	C13H27N3O8P$^+$	384.1530					384.1531	0.13

Table S83. PnAla-aThr-Met, PnAla-aThr-Phe, and PnAla-aThr-aThr tripeptide fragments

Fragment				PnAla-aThr-Met		PnAla-aThr-Phe		PnAla-aThr-aThr	
ID	Structure	Formula	Calc m/z	Obs m/z	Δppm	Obs m/z	Δppm	Obs m/z	Δppm
a	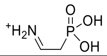	C2H7NO3P^{+}	124.0158					124.0158	0.32
b	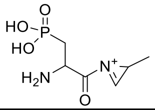	C6H12N2O4P^{+}	207.0529	207.0531	1.04			207.0531	0.92
c	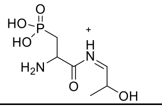	C6H14N2O5P^{+}	225.0635	225.0638	1.24	225.0635	0.18	225.0633	-0.72
d	*e* – H2O	C7H12N2O5P^{+}	235.0478					235.0477	-0.38
e	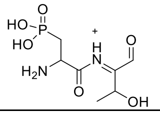	C7H14N2O6P^{+}	253.0584	253.0584	0.04	253.0583	-0.25	253.0584	-0.19
ac	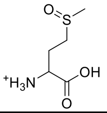	C5H12NO3S^{+}	166.0532	166.0535	1.39				
ad	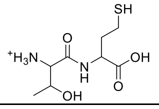	C8H17N2O4S^{+}	237.0904	237.0906	1.17				
ae	*af* – H2O	C12H23N3O7PS^{+}	384.0989	384.0988	-0.12				
af	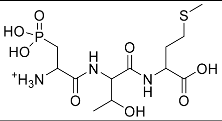	C12H25N3O8PS^{+}	402.1095	402.1096	0.35				
ag	*aj* – H2O	C16H23N3O7P^{+}	400.1268			400.1265	-0.71		
ah	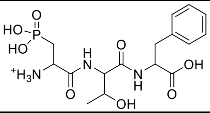	C16H25N3O8P^{+}	418.1374			418.1369	-1.05		
ai	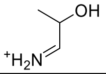	C3H8NO^{+}	74.0600					74.0601	0.86
aj	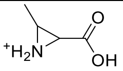	C4H8NO2^{+}	102.0550					102.0550	0.33
ak	*am* – (H2O^{+}CO)	C10H19N3O6P^{+}	308.1006					308.1005	-0.23
al	*am* – H2O	C11H19N3O7P^{+}	336.0955					336.0953	-0.52
am	*an* – H2O	C11H21N3O8P^{+}	354.1061					354.1060	-0.09
an	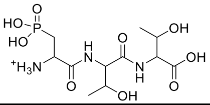	C11H23N3O9P^{+}	372.1166					372.1163	-0.98

Table S84. PnAla-Gln-Val, PnAla-Gln-Ile, and PnAla-Gln-Leu tripeptide fragments

Fragment				PnAla-Gln-Val		PnAla-Gln-Ile		PnAla-Gln-Leu	
ID	Structure	Formula	Calc m/z	Obs m/z	Δppm	Obs m/z	Δppm	Obs m/z	Δppm
a	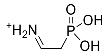	$C2H7NO3P^+$	124.0158			124.0158	0.20		
b	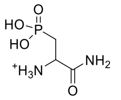	$C3H10N2O4P^+$	169.0373	169.0373	0.23	169.0372	-0.21	169.0373	0.18
c	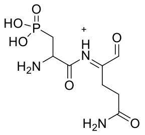	$C8H15N3O6P^+$	280.0693	280.0692	-0.52	280.0691	-0.86	280.0693	-0.10
d	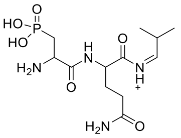	$C12H24N4O6P^+$	351.1428	351.1426	-0.47				
e	*g* – NH3	$C13H21N3O7P^+$	362.1112	362.1106	-1.62				
f	*g* – H2O	$C13H24N4O7P^+$	379.1377	379.1377	0.03				
g	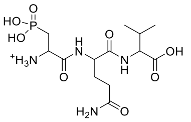	$C13H26N4O8P^+$	397.1483	397.1480	-0.68				
h	*c* – NH3	$C8H12N2O6P^+$	263.0428			263.0425	-0.87		
i	*j* – (NH3 + H2O)	$C13H21N3O5P^+$	330.1213			330.1208	-1.47		
j	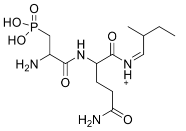	$C13H26N4O6P^+$	365.1585			365.1583	-0.33		
k	*l* – NH3	$C14H23N3O7P^+$	376.1268			376.1265	-0.86		
l	*m* – H2O	$C14H26N4O7P^+$	393.1534			393.1531	-0.55		
m	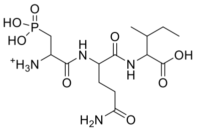	$C14H28N4O8P^+$	411.1639			411.1635	-1.10		
n	*o* – NH3	$C14H23N3O7P^+$	376.1268					376.1268	0.09
o	*p* – H2O	$C14H26N4O7P^+$	393.1534					393.1530	-1.02
p	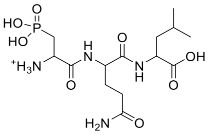	$C14H28N4O8P^+$	411.1639					411.1637	-0.44

Table S85. PnAla-His-Phe and PnAla-His-Trp tripeptide fragments

Fragment				PnAla-His-Phe		PnAla-His-Trp	
ID	Structure	Formula	Calc m/z	Obs m/z	Δppm	Obs m/z	Δppm
a	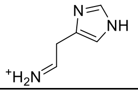	$C_5H_8N_3^+$	110.0713	110.0712	-0.25	110.0713	0.07
b	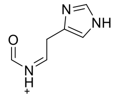	$C_6H_8N_3O^+$	138.0662	138.0662	0.44		
c	*b* + H_2O	$C_6H_{10}N_3O_2^+$	156.0768	156.0768	0.36	156.0768	0.28
d	*b* + CO	$C_7H_8N_3O_2^+$	166.0611	166.0612	0.57		
e	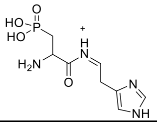	$C_8H_{14}N_4O_4P^+$	261.0744	261.0746	-0.53		
f	*h* – H_2O	$C_9H_{12}N_4O_4P^+$	271.0591	271.0588	-0.90		
g	*h* – NH_3	$C_9H_{11}N_3O_5P^+$	272.0431	272.0428	-1.16		
h	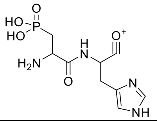	$C_9H_{14}N_4O_5P^+$	289.0696	289.0693	-1.14	289.0693	-0.04
i	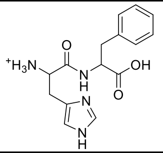	$C_{15}H_{19}N_4O_3^+$	303.1452	303.1451	-0.27		
j	*h* + H_2O	$C_9H_{16}N_4O_6P^+$	307.0802	307.0802	-0.12		
k	*i* + CO – H_2O	$C_{16}H_{17}N_4O_3^+$	313.1295	313.1294	-0.40		
l	*m* – H_2O	$C_{18}H_{21}N_5O_5P^+$	418.1275	418.1274	-0.24		
m	*n* – H_2O	$C_{18}H_{23}N_5O_6P^+$	436.1381	436.1377	-0.76		
n	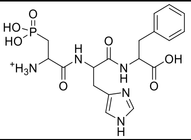	$C_{18}H_{25}N_5O_7P^+$	454.1486	454.1486	0.06		
o	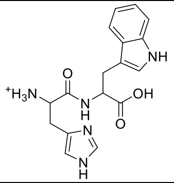	$C_{17}H_{20}N_5O_3^+$	342.1561			342.1560	-0.09
p	*o* + CO – H_2O	$C_{18}H_{18}N_5O_3^+$				352.1403	-0.07
q	*r* – H_2O	$C_{20}H_{24}N_6O_6P^+$	475.1490			475.1490	0.10
r	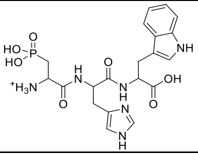	$C_{20}H_{26}N_6O_7P^+$	493.1595			493.1593	-0.39

Table S86. PnAla-Gly-Val, PnAla-Gly-Ile, and PnAla-Gly-Leu tripeptide fragments

Fragment				PnAla-Gly-Val		PnAla-Gly-Ile		PnAla-Gly-Leu	
ID	Structure	Formula	Calc m/z	Obs m/z	Δppm	Obs m/z	Δppm	Obs m/z	Δppm
a	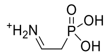	$C2H7NO3P^+$	124.0158	124.0157	-0.50	124.0158	0.26	124.0157	-0.69
b	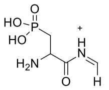	$C4H10N2O4P^+$	181.0373	181.0371	-1.01	181.0371	-1.05	181.0370	-1.23
c	*b* + CO	$C5H10N2O5P^+$	209.0322	209.0320	-0.68	209.0320	-1.04	209.0320	-0.97
d	*c* + H2O	$C5H12N2O6P^+$	227.0428	227.0425	-0.89			227.0425	-1.30
e	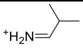	$C4H10N^+$	72.0808	72.0808	0.43				
f	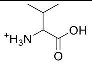	$C5H12NO2^+$	118.0863	118.0861	-0.93				
g	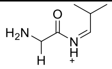	$C6H13N2O^+$	129.1022	129.1021	-1.18				
h	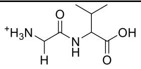	$C7H15N2O3^+$	175.1077	175.1076	-0.45				
i	*j* – H2O	$C9H17N3O4P^+$	262.0951	262.0949	-1.02				
j	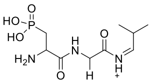	$C9H19N3O5P^+$	280.1057	280.1054	-1.14				
k	*l* – H2O	$C10H17N3O5P^+$	290.0900	290.0896	-1.47				
l	*m* – H2O	$C10H19N3O6P^+$	308.1006	308.1003	-1.02				
m	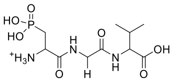	$C10H21N3O7P^+$	326.1112	326.1107	-1.36				
n	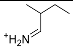	$C5H12N^+$	86.0964			86.0963	-1.22		
o	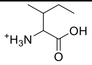	$C6H14NO2^+$	132.1019			132.1019	0.06		
p	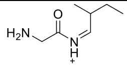	$C7H15N2O^+$	143.1179			143.1179	-0.11		
q	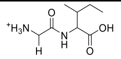	$C8H17N2O3^+$	189.1234			189.1231	-1.62		
r	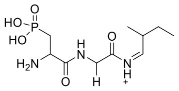	$C10H21N3O5P^+$	294.1213			294.1209	-1.54		
s	*t* – H2O	$C11H21N3O6P^+$	322.1163			322.1158	-1.44		
t	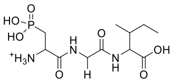	$C11H23N3O7P^+$	340.1268			340.1263	-1.47		
u	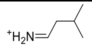	$C5H12N^+$	86.0964					86.0963	-1.31
v	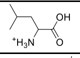	$C6H14NO2^+$	132.1019					132.1018	-0.76
w	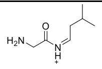	$C7H15N2O^+$	143.1179					143.1178	-0.30
x	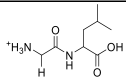	$C8H17N2O3^+$	189.1234					189.1232	-1.09
y	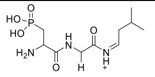	$C10H21N3O5P^+$	294.1213					294.1208	-1.70
z	*ab* – H2O	$C11H19N3O5P^+$	304.1057					304.1053	-1.41
aa	*ac* – H2O	$C11H21N3O6P^+$	322.1163					322.1158	-1.39

ab	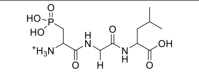	C11H23N3O7P⁺	340.1268					340.1264	-1.35

Table S87. PnAla-Gly-Met, PnAla-Gly-Phe, and PnAla-Gly-Tyr tripeptide fragments

Fragment				PnAla-Gly-Met		PnAla-Gly-Phe		PnAla-Gly-Tyr	
ID	Structure	Formula	Calc m/z	Obs m/z	Δppm	Obs m/z	Δppm	Obs m/z	Δppm
a	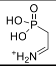	C2H7NO3P⁺	124.0158	124.0160	1.23				
b	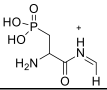	C4H10N2O4P⁺	181.0373	181.0372	-0.18	181.0371	-0.89	181.0371	0.66
c	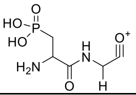	C5H10N2O5P⁺	209.0322			209.0321	-0.23	209.0319	-1.15
d	*c* + H2O	C5H12N2O6P⁺	227.0428	227.0427	-0.03				
ac	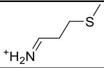	C4H10NS⁺	104.0529	104.0528	-0.20				
ad	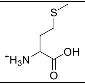	C5H12NO2S⁺	150.0583	150.0584	0.70				
ae	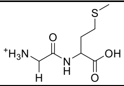	C7H15N2O3S⁺	207.0798	207.0799	0.62				
af	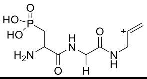	C8H15N3O5P⁺	264.0744	264.0744	0.09				
ag	*ah* – H2O	C10H17N3O5PS⁺	322.0621	322.0620	-0.31				
ah	*ai* – H2O	C10H19N3O6PS⁺	340.0727	340.0727	-0.04				
ai	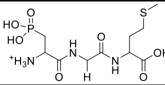	C10H21N3O7PS⁺	358.0832	358.0833	0.25				
aj	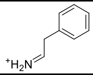	C8H10N⁺	120.0808			120.0807	-0.74		
ak	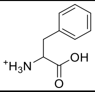	C9H12NO2⁺	166.0863			166.0862	-0.44		
al	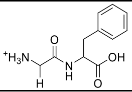	C11H15N2O3⁺	223.1077			223.1075	-0.83		
am	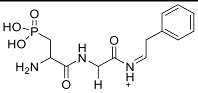	C13H19N3O5P⁺	328.1057			328.1053	-1.15		
an	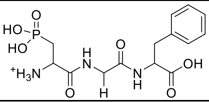	C14H21N3O7P⁺	374.1112			374.1103	-2.25		
ao	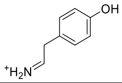	C8H10NO⁺	136.0757					136.0757	-0.17
ap	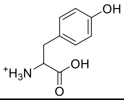	C9H12NO3⁺	182.0812					182.0811	-0.36
aq	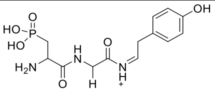	C13H19N3O6P⁺	344.1006					344.1000	-1.87
ar	*as* – H2O	C14H19N3O7P⁺	372.0955					372.0950	-1.29
as	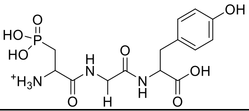	C14H21N3O8P⁺	390.1061					390.1056	-1.35

Table S88. PnAla-Gly-Trp, PnAla-Gly-aThr, and PnAla-Gly-Gly tripeptide fragments

Fragment				PnAla-Gly-Trp		PnAla-Gly-aThr		PnAla-Gly-Gly	
ID	Structure	Formula	Calc m/z	Obs m/z	Δppm	Obs m/z	Δppm	Obs m/z	Δppm
a	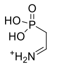	C2H7NO3P$^+$	124.0158					124.0158	-0.42
b	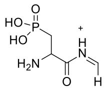	C4H10N2O4P$^+$	181.0373	181.0372	-0.33			181.0370	-1.29
c	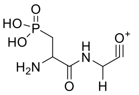	C5H10N2O5P$^+$	209.0322	209.0319	-1.20	209.0321	-0.59	209.0319	-1.12
at	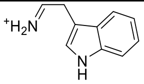	C10H11N2$^+$	159.0917	159.0916	-0.78				
au	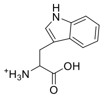	C11H13N2O2$^+$	205.0972	205.0969	-1.04				
av	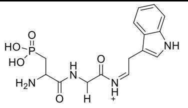	C15H20N4O5P$^+$	367.1166	367.1156	-2.63				
aw	*ax* – H2O	C16H20N4O6P$^+$	395.1115	395.1116	0.22				
ax	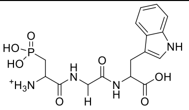	C16H22N4O7P$^+$	413.1221	413.1207	-3.38				
ay	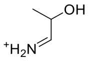	C3H8NO$^+$	74.0600			74.0600	-0.53		
az	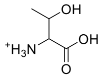	C4H8NO2$^+$	102.0550			102.0549	-0.91		
ba	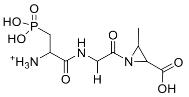	C9H17N3O7P$^+$	310.0799			310.0796	-0.86		
bb	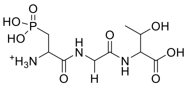	C9H19N3O8P$^+$	328.0904			328.0899	-1.75		
bc	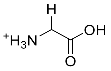	C2H6NO2$^+$	76.0393					76.0393	-0.52
bd	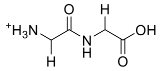	C4H9N2O3$^+$	133.0608					133.0607	-0.85
be	*bf* – H2O	C7H11N3O5P$^+$	248.0431					248.0428	-1.04
bf	*bg* – H2O	C7H13N3O6P$^+$	266.0537					266.0532	-1.63
bg	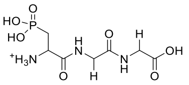	C7H15N3O7P$^+$	284.0642					284.0638	-1.35

Table S89. Asp-Ala-Val tripeptide fragments

Fragment				Asp-Ala-Val	
ID	Structure	Formula	Calc m/z	Obs m/z	Δppm
a	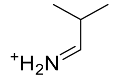	C4H10N+	72.0808	72.0809	1.04
b	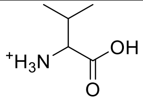	C5H12N2O2+	118.0863	118.0862	-0.54
c	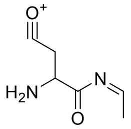	C6H9N2O2+	141.0689	141.0658	-0.10
d	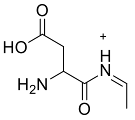	C6H11N2O3+	159.0764	159.0764	-0.12
e	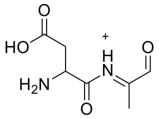	C7H11N2O4+	187.0713	187.0713	0.07
f	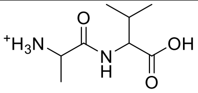	C8H17N2O3+	189.1234	189.1234	-0.05
g	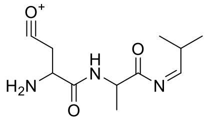	C11H18N3O3+	240.1343	240.1340	-1.10
h	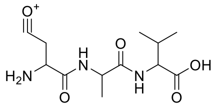	C12H20N3O5+	286.1398	286.1397	-0.31
i	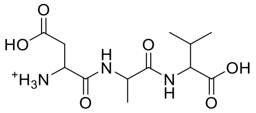	C12H22N3O6+	304.1503	304.1501	-0.79

REFERENCES

[1] P. M. Gerhardt, R. G. E.; Wood, W. A.; Krieg, N. R., *Methods for General and Molecular Bacteriology*, 2nd ed., American Society for Microbiology, **1994**.

[2] R. Y. Stanier, N. J. Palleroni, M. Doudoroff, *J Gen Microbiol* **1966**, *43*, 159-271.

[3] J. R. S. Green, J., *Molecular Cloning*, 4th ed., Cold Spring Harbor Laboratory Press, **2012**.

[4] Y. Zhang, T. M. Pham, C. Kayrouz, K. S. Ju, *J Am Chem Soc* **2022**, *144*, 9634-9644.

[5] R. R. Wick, L. M. Judd, C. L. Gorrie, K. E. Holt, *PLoS Comput Biol* **2017**, *13*, e1005595.

[6] T. Tatusova, M. DiCuccio, A. Badretdin, V. Chetvernin, E. P. Nawrocki, L. Zaslavsky, A. Lomsadze, K. D. Pruitt, M. Borodovsky, J. Ostell, *Nucleic Acids Res* **2016**, *44*, 6614-6624.

[7] a) J. Mistry, S. Chuguransky, L. Williams, M. Qureshi, G. A. Salazar, E. L. L. Sonnhammer, S. C. E. Tosatto, L. Paladin, S. Raj, L. J. Richardson, R. D. Finn, A. Bateman, *Nucleic Acids Res* **2021**, *49*, D412-D419; b) D. L. Wheeler, D. M. Church, S. Federhen, A. E. Lash, T. L. Madden, J. U. Pontius, G. D. Schuler, L. M. Schriml, E. Sequeira, T. A. Tatusova, L. Wagner, *Nucleic Acids Res* **2003**, *31*, 28-33; c) S. Lu, J. Wang, F. Chitsaz, M. K. Derbyshire, R. C. Geer, N. R. Gonzales, M. Gwadz, D. I. Hurwitz, G. H. Marchler, J. S. Song, N. Thanki, R. A. Yamashita, M. Yang, D. Zhang, C. Zheng, C. J. Lanczycki, A. Marchler-Bauer, *Nucleic Acids Res* **2020**, *48*, D265-D268.

[8] M. J. Sullivan, N. K. Petty, S. A. Beatson, *Bioinformatics* **2011**, *27*, 1009-1010.

[9] K. A. Datsenko, B. L. Wanner, *Proc Natl Acad Sci U S A* **2000**, *97*, 6640-6645.

[10] B. T. Circello, A. C. Eliot, J. H. Lee, W. A. van der Donk, W. W. Metcalf, *Chem Biol* **2010**, *17*, 402-411.

[11] Y. Zhang, L. Chen, J. A. Wilson, J. Cui, H. Roodhouse, C. Kayrouz, T. M. Pham, K. S. Ju, *J Am Chem Soc* **2022**, *144*, 9938-9948.

[12] P. Zhang, W. Chan, I. L. Ang, R. Wei, M. M. T. Lam, K. M. K. Lei, T. C. W. Poon, *Sci Rep* **2019**, *9*, 6453.

[13] A. C. Eliot, B. M. Griffin, P. M. Thomas, T. W. Johannes, N. L. Kelleher, H. Zhao, W. W. Metcalf, *Chem Biol* **2008**, *15*, 765-770.

[14] X. Yu, N. P. Price, B. S. Evans, W. W. Metcalf, *J Bacteriol* **2014**, *196*, 1768-1779.
